# COGITO: A Coarse-Grained Force Field for the Simulation of Macroscopic Properties of Triacylglycerides

**DOI:** 10.1021/acs.jctc.2c00975

**Published:** 2023-02-02

**Authors:** Robert J. Cordina, Beccy Smith, Tell Tuttle

**Affiliations:** † Mondele̅z UK R&D Ltd., P.O. Box 12, Bournville Lane, BirminghamB30 2LU, U.K.; ‡ Department of Pure and Applied Chemistry, University of Strathclyde, 295 Cathedral Street, GlasgowG1 1XL, U.K.

## Abstract

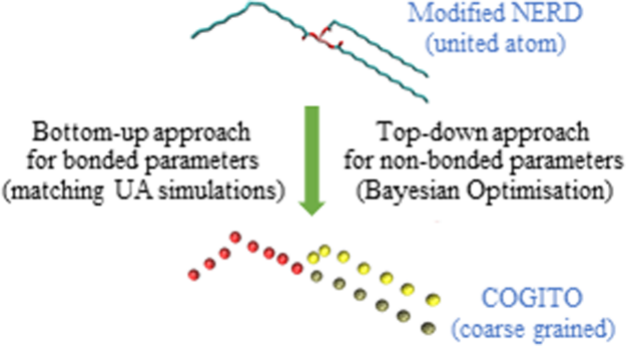

The use of molecular dynamics simulations is becoming ever more widespread; however, the application of this to pure triacylglyceride (TAG) systems is not. In this study, we are presenting the development, and validation, of a new force field (FF), which we have called the COarse-Grained Interchangeable Triacylglyceride-Optimized FF. The FF has been developed using both a bottom-up and top-down approach for different parameters, with the non-bonded parameters being optimized using a Bayesian optimization method. While the FF was developed using monounsaturated TAGs, results show that it is also suitable for fully saturated TAGs. Description of molecules which were not used during the development of the FF is carried out simply by interchanging the bead in the molecule topologies. Results show that the FF can reproduce the macroscopic properties (density and lattice parameters) of pure TAGs as both crystals and melt with high accuracy, as well as reproduce the differences in enthalpies.

## Introduction

In previous work,^1^ we described the amendment of the NERD force field (FF) to reproduce the macroscopic properties of unsaturated triacylglycerides (TAGs) accurately. This is a united-atom (UA) FF, that is, one where methine, methylene, and methyl groups are all considered as one “heavy atom” rather than two, three, or four separate atoms, respectively. This brings the number of atoms, and thus the number of bonded and non-bonded interactions, down significantly, giving a computational advantage over using an all-atom FF. Taking 1-palmitoyl-2-oleoyl-3-stearoyl-*sn*-glycerol (*sn*-POSt) as an example, this would have 64 atoms in a UA FF, but 168 atoms in an all-atom FF. If this had to be multiplied by hundreds or thousands of molecules that could be simulated in a Molecular Dynamics (MD) simulation, the advantage in using a UA FF becomes clear very quickly.

Within a coarse-grained (CG) FF, multiple atoms, such as a CH_2_CH_2_CH_2_ chain, are considered as one “superatom” (commonly referred to as a bead), which brings the number of particles down even further. Thus, the CG FF is much less computationally intensive as the number of both bonded and non-bonded terms to be calculated are reduced significantly. This allows for simulations over longer timescales or of larger systems, although it comes at a cost of some loss of resolution, with results from CG simulations potentially being less accurate. This loss of accuracy may however be outweighed by the increase in the efficiency, particularly if the property of interest can only be accessed over longer timescales or using larger systems. The accuracy largely depends on the CG resolution (i.e., the number of atoms per bead) and accurate parameterization of the CG FF, which in turn depends on proper validation of the CG FF against atomistic simulation results and/or empirical measurements.

Any FF is parameterized for molecules in a specific environment and thus only applicable within that parameterization space. We found this to be the case with one of the more widely used CG FFs, the Martini 2 FF,^2^ where this FF has been parameterized for molecules in an aqueous solution. Moreover, this version of the FF, while distinguishing between saturated and unsaturated beads, does not make a distinction between fatty acid chains of similar, but different, length. For example, a palmitic chain and a stearic chain (having, excluding the carbonyl carbon, 15 and 17 saturated carbons, respectively) are both represented by four beads of the same type.^2^ These drawbacks led us to look for a different CG FF for pure TAG systems.

The only published studies that we know, of where a CG FF for TAGs in a non-aqueous system has been developed or used, are those by the Milano group.^3−6^ The CG FF used in these studies was developed for, and validated on, symmetric, fully saturated TAGs, such as tridecanoin (10-carbon fatty acid chains), tripalmitin (16-carbon fatty acid chains), and tristearin (18-carbon fatty acid chains). While these TAGs can be found in nature, non-symmetric TAGs which contain at least one unsaturated fatty acid chain are generally more common. Even natural fats which are considered to be high in saturated fatty acids have a majority of TAGs which contain at least one unsaturated fatty acid chain. Beef fat’s unsaturated fatty acid chain content, for example, has been shown to be 33.4% of the total fatty acids.^7^ On going from hard fats to soft fats to oils, the percentage of unsaturated fatty acids increases. Thus, having a FF which can simulate the behavior of both saturated and unsaturated TAGs could be used for a wider range of applications.

In this study, we outline our efforts to develop a CG FF which can reproduce the macroscopic properties of pure TAGs (i.e., in a non-aqueous solution), be they saturated or unsaturated, accurately, building on the modified NERD UA FF.^1^ Our intention was to develop a FF which is flexible, that is, develop parameters for a set of beads which can be interchanged to build different TAGs as required—the **CO**arse-**G**rained **I**nterchangeable **T**riacylglyceride-**O**ptimized, COGITO, FF.

## Computational Methods

All simulations were carried out using GROMACS^8^ 2019.3. The FF parameters for the UA simulations were the same as described previously.^1^ All UA equilibrations were carried out using an isobaric/isothermal (NPT) ensemble, using a v-rescale thermostat and a Berendsen barostat. Constant atmospheric pressure (1.01325 bar) was maintained by using anisotropic pressure coupling, with a compressibility of 1 × 10^–5^ bar^–1^ in the *x-*, *y-*, and *z*-directions. Temperature coupling was set at 1 ps, while pressure coupling was set at 10 ps. A time-step of 2 fs was used for all UA FF equilibrations, with frames saved every 5000 steps. The cut-off scheme was set to Verlet, with the Coulomb and van der Waals (vdW) cut-off distances set to 1.1 nm. The vdW modifier was set to potential-shift, and the Coulomb type was set to Particle-Mesh Ewald (PME) (PME order = 4). In all of the UA simulation results in this paper, equilibrations were carried out on a box made up of 100 molecules (25 unit cells, with each unit cell being made up of four molecules in the case when starting from a crystalline structure, and 100 randomly placed molecules in the case of the melt equilibrations). The unit cells were obtained from Peschar et al.^9^ and van Mechelen et al.^10^ with the crystalline box being built by stacking five unit cells in each of the *a*- and *c*-directions.

**Figure 1:**
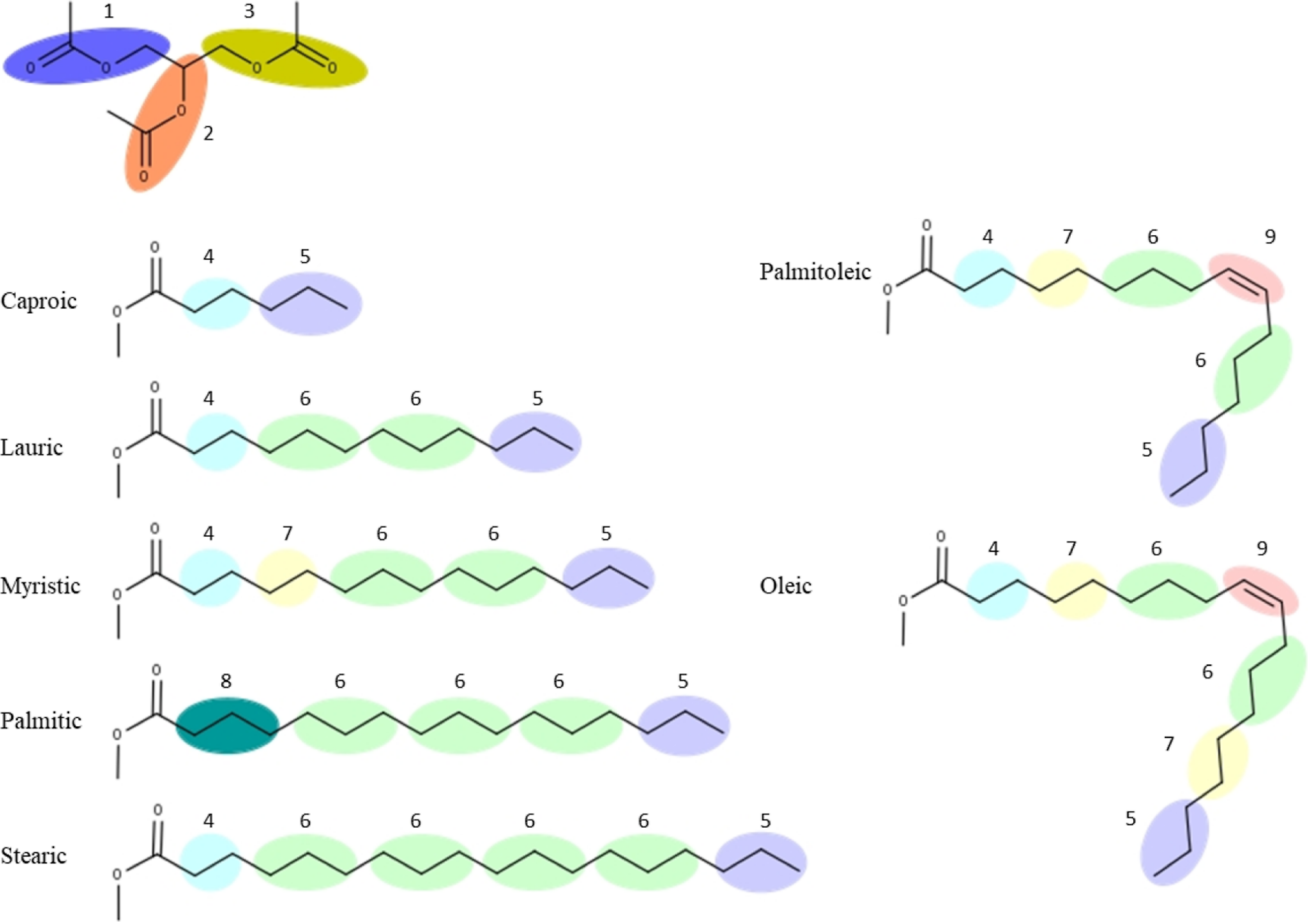
Coarse-graining of different TAGs using the newly devised CG mapping system (with implicit hydrogens not shown for clarity). 1 (dark purple): 1CH2OCO, 2 (orange): CHOCO, 3 (dark yellow): 3CH2OCO, 4 (light blue): C2H4E, 5 (light purple): C3H7T, 6 (light green): C3H6, 7 (light yellow): C2H4, 8 (dark green): C3H6E, and 9 (pink): CHCH.

All CG equilibrations were carried out using an NPT ensemble, using a time-step of 25 fs, at a range of temperatures (250–300 K) as defined in the Supporting Information (see Supporting Information 6) and a pressure of 1.01325 bar using a v-rescale thermostat and a Berendsen barostat. Anisotropic pressure coupling with a compressibility of 1 × 10^–5^ bar^–1^ in the *x*-, *y*-, and *z*-directions was used. Temperature coupling was set at 1 ps, while pressure coupling was set at 10 ps. The cut-off scheme was set to Verlet, with the Coulomb and vdW cut-off distances set to 1.1 nm. The vdW modifier was set to potential-shift and the Coulomb type was set to Particle-Mesh Ewald (PME) (PME order = 4). All equilibrations were run for 5 ns each unless specified. Frames were saved every 200 steps. All starting crystalline TAG coordinates described in this paper were mapped from crystal structures obtained by X-ray diffraction^9−12^ using Centre-of-Geometry (CoG) mapping, as per Figure 1.

Enthalpies of fusion were determined by equilibrating a pure TAG crystal system consisting of 100 molecules, using the same CG simulation settings as described, for 50 ns at its empirical melting point,^13−15^ then increasing the temperature by 150 K to ensure complete melting and allowing to equilibrate for a further 50 ns, and then cooled rapidly again to the melting temperature, followed by a further equilibration for 50 ns. The enthalpies for the crystalline and melted systems were obtained by an average enthalpy, using bootstrap statistics,^16,17^ for the last 20 ns of the crystalline and melt equilibration stages at the empirical melting temperature, with data recorded every 1000 steps. This was repeated 10 times for each system.

Enthalpies of vaporization were determined by following a similar methodology as for Martini 3;^18^ however, equilibrating a pure TAG system at the empirical vaporization temperature^19^ for 50 ns (instead of a reference temperature) and then a single molecule in a box for 50 ns, with data recorded every 1000 steps. The melt was equilibrated using an NPT ensemble, while the single molecule was equilibrated using an NVT ensemble in a box measuring 7 × 7 × 7 nm.

The enthalpies and energies were extracted using the GROMACS^8^ gmx energy program. The enthalpy of fusion (Δ*H*_fus_) was calculated by determining the difference between the average enthalpies of the melt and the crystal. The enthalpy of vaporization (Δ*H*_vap_) was determined using the equation Δ*H*_vap_ ≈ *U*_gas_ – *U*_liq_ + RT, where *U*_gas_ and *U*_liq_ are the total energies per mole of the gas and liquid phase in the NVT and NPT ensembles, respectively.^18^

## Results and Discussion

### CG Mapping

The first step in the development of this FF was to determine the CG mapping, that is, how many atoms would be grouped in the various beads making up the FF, as well as whether these would be differentiated depending on their position in the TAG. Our starting point for this was the paper by Brasiello et al.,^4^ where they had determined that grouping a glycerol carbon plus its bonded hydrogens with the ester group, as well as differentiating between the resulting beads at the glycerol *sn*-1/*sn*-3 and *sn*-2 positions, gave the best results. The only other major bead differentiation in this paper was between CH_2_CH_2_CH_2_ beads and terminal CH_2_CH_2_CH_3_ beads. Given that this study was on tridecanoin, each fatty acid chain was thus made up of 2 x CH_2_CH_2_CH_2_ beads and 1 x terminal CH_2_CH_2_CH_3_ beads (this adds up to nine carbons; however, the 10^th^ carbon making up the decanoin chain is part of the glycerol/ester bead). This study made no differentiation between a CH_2_CH_2_CH_2_ bead adjacent to the glycerol/ester bead or a CH_2_CH_2_CH_2_ bead in the middle of a fatty acid chain. In a subsequent study by Pizzirusso et al.,^6^ the CG FF was extended to tristearin and tripalmitin, where the authors again described four types of beads, a CHOCO bead, a CH_2_OCO bead, a CH_2_CH_2_CH_2_ bead, and a CH_2_CH_2_CH_3_ bead. This grouping pattern, however, has the drawback that chain lengths that differ by less than three carbon atoms, such as a 16-carbon palmitic and 18-carbon stearic chain, cannot be differentiated accurately.

Given our requirements to make this FF suitable for unsaturated TAGs with differing chain lengths, we decided to devise a new CG mapping system for the hydrocarbon chains. Also, given the partial atomic charges used in the modified NERD FF, summing up these partial charges for a CHOCO or CH_2_OCO bead results in an overall partial charge of −0.05, with any adjacent hydrocarbon bead having a partial charge of +0.05. We thus decided that any such beads would have these partial charges and would not be neutral as per the Brasiello et al.^4^ and Pizzirusso et al.^6^ papers. We thus devised a CG mapping, as listed in Table 1 and shown in Figure 1.

**Table 1:** CG Mapping of TAG Beads

bead name	description	weight (rmm)	partial charge
1CH2OCO	*sn*-1 glycerol carbon plus ester group	58.036	–0.05
2CHOCO	*sn*-2 glycerol carbon plus ester group	57.028	–0.05
3CH2OCO	*sn*-3 glycerol carbon plus ester group	58.036	–0.05
CHCH	CH=CH alkene bead	26.037	0
C2H4E	CH_2_CH_2_ bead adjacent to a CHOCO/CH2OCO bead	28.053	+0.05
C2H4	CH_2_CH_2_ bead not adjacent to a CHOCO/CH2OCO bead	28.053	0
C3H6E	CH_2_CH_2_CH_2_ bead adjacent to a CHOCO/CH2OCO bead	42.080	+0.05
C3H6	CH_2_CH_2_CH_2_ bead not adjacent to a CHOCO/CH2OCO bead	42.080	0
C3H7T	terminal CH_2_CH_2_CH_3_ bead	43.088	0

An increase in the number of a FF bead types does not result in an increase in computational time as this depends on the number of beads present in the simulation box, which determines how many bonded and non-bonded terms need to be calculated. Having more beads defined, however, should lead to a more refined FF which can thus describe and differentiate between molecules better. Using the beads as described above, we were thus able to map a number of different fatty acids of different lengths and level of saturation, as shown in Figure 1. As can be seen in the figure, the use of two and three-carbon beads is necessary to have the correct number of carbons in a fatty acid chain, as well as giving a lot of flexibility in building different TAGs. Other CG mapping schemes could have been devised, such as having a (CH_2_)_4_ bead or a CH=CHCH_2_ bead. Having such beads would translate into having fewer beads per fatty acid chain and therefore making the FF more computationally efficient. However, we had to strike a balance between resolution and efficiency, and, given the importance of the alkene structure in unsaturated TAGs, having the alkene carbons in a bead with another carbon would result in losing too much resolution between *cis* and *trans* alkene configurations.

### Analysis of UA Simulation Data

Given that this new FF is based on the modified NERD FF,^1^ a number of simulations were carried out using the latter FF, with part of the data obtained (distances, angles, and dihedrals) being used to develop the COGITO FF, while experimental data were used to validate it. The UA simulations were carried out on three TAGs, namely, *sn*-POSt, 1,3-dipalmitoyl-2-oleoyl-*sn*-glycerol (*sn*-POP), and 1,3-distearoyl-2-oleoyl-*sn*-glycerol (*sn*-StOSt). These three TAGs were chosen as the crystalline structures of their β_2_ and β_1_ polymorphs are known,^9,10^ and are widely found in the confectionery industry. Both the crystalline and melt equilibrations were carried out at 280, 300, 325, 350, 375, 400, and 410 K for 50 ns, using the settings as described in the Computational Methods section. A custom script was written in Python 3 to evaluate the desired measurements (see Supporting Information 1). The coordinates of every atom for the final 10 ns of a 50 ns equilibration trajectory were extracted to ensure that the captured data were for a well-equilibrated system, and thus, any averages taken are more accurate. The data extraction was carried out using the MDTraj^20^ (version 1.9.4) Python module. These UA atom coordinates were then used to determine the CoG coordinates of each mapped CG bead, which in turn were used to calculate the bead distances, angles, and torsion angles using the relevant standard Euclidean geometry equations.

### Choice of FF Functions and Parameterization of the COGITO CG FF

As with any FF, the COGITO FF is made up of two parts; the bonded and non-bonded potential functions describing the FF potential energy and the parameters used in these functions.^21^ A simple harmonic potential was chosen for the bond-stretch term, while a cosine-based (GROMOS-96) potential was chosen for the angle-bending terms. Similar to other CG FFs, such as Martini^2,18^ and that developed by the Milano group,^3−6^ the torsion angle (or dihedral) potential was not included in the COGITO FF. This decision was arrived at after an in-depth analysis of the torsion angle distribution data from the UA equilibrations, as the rotation energy barrier was small in all cases (see Supporting Information 2), the inclusion of a torsional angle potential had no meaningful effect on the simulated structures and, given the very flexible nature of the fatty acids chains as well as having a number of the 3-body reference angles making up the 4-body dihedrals close to 180°, instead resulted in unstable simulations, as described by Souza et al. and Bulacu et al.^18,22^ With respect to the non-bonded potential terms, a standard Coulomb potential was chosen for electrostatic interactions, while a Lennard-Jones (LJ) function was chosen for the repulsive and dispersion potential terms. To calculate the LJ function between two different beads, the Lorentz–Berthelot mixing rules^23,24^ were employed. The standard Coulomb potential was chosen as no reaction-field is used, as per our previous study.^1^ Non-bonded interactions between atoms which are one bond away were excluded. The COGITO FF potential is given in eq 1.
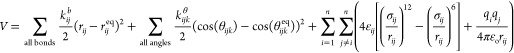


Equation 1. Potential energy equation used for the COGITO FF. The force constants for the bond between beads *i*–*j* and the angle between bonded beads *i*-*j*-*k* are represented by *k*_*ij*_^*b*^ and *k*_*ijk*_^θ^, respectively. ε_*ij*_ and σ_*ij*_ are the non-bonded LJ parameters. The distance between any two beads *i* and *j* is given by *r*_*i*j_ and the angle of bonded beads *i*-*j*-*k* is given by θ_*ijk*_, while *r*_*ij*_^eq^ and θ_*ijk*_^eq^ are the equilibrium distance and angle between two and three bonded beads, respectively. *q*_*i*_ and *q*_*j*_ are the partial charges of beads *i* and *j*.

The bond-stretching equilibrium distances were determined by first extracting all bond distances for all molecules for the last 10 ns of the UA equilibrations and generating plots of frequency versus distance. The CG equilibrium values for all distances were then varied until a plot of frequency versus distance of any given specific bond system of the CG-equilibrated system overlapped the UA results plot as closely as possible (see Supporting Information 3). In all cases, this meant matching the mean of the plot as the distribution of the CG-equilibrated distances was wider (and correspondingly the maximum was lower), due to a smoothing of the potential as a result of the coarse-graining. The bond-stretching force constants were set by grouping the bond pairs by the standard deviation of the UA simulation frequency plots and then assigning a value based on similar systems found in published work.^2,4^ All of this was carried out based on the results of the crystalline UA simulations of both the β_2_ and β_1_ polymorphs of *sn*-POP, *sn*-POSt, and *sn*-StOSt. The final chosen bond-stretching parameters for the FF are given in Table 2.

**Table 2:** Bond-Stretching Parameters for the COGITO FF

bead *i*	bead *j*	*r*_ij_^eq^(nm)	*k*_ij_^b^(kJ mol^–1^nm^–2^)
1CH2OCO	2CHOCO	0.470	4000
1CH2OCO	C2H4E	0.300	5000
1CH2OCO	C3H6E	0.365	5000
2CHOCO	3CH2OCO	0.350	4000
2CHOCO	C2H4E	0.300	5000
2CHOCO	C3H6E	0.360	5000
3CH2OCO	C2H4E	0.290	5000
3CH2OCO	C3H6E	0.350	5000
CHCH	C3H6	0.340	3000
C2H4E	C2H4	0.280	3000
C2H4E	C3H6	0.335	3000
C2H4	C3H6	0.345	3000
C2H4	C3H7T	0.345	3000
C3H6E	C3H6	0.400	3000
C3H6	C3H6	0.400	3000
C3H6	C3H7T	0.400	3000

The equilibrium CG bond angles were set by determining the bond angles of the CG-mapped crystals, as obtained from the literature,^9,10^ while the angle force constants were set using a similar methodology as described for the bond distance force constants (Table 3).

**Table 3:** Angle-Vibration Parameters for the COGITO FF

bead *i*	bead *j*	bead *k*	θ_*ijk*_^eq^(°)	*k*_*ijk*_^θ^(kJ mol^–1^)
1CH2OCO	2CHOCO	3CH2OCO	65	120
1CH2OCO	2CHOCO	C2H4E	147	100
1CH2OCO	2CHOCO	C3H6E	140	100
1CH2OCO	C2H4E	C2H4	166	50
1CH2OCO	C2H4E	C3H6	171	50
1CH2OCO	C3H6E	C3H6	157	50
2CHOCO	1CH2OCO	C2H4E	144	100
2CHOCO	1CH2OCO	C3H6E	145	100
2CHOCO	3CH2OCO	C2H4E	126	35
2CHOCO	3CH2OCO	C3H6E	131	35
2CHOCO	C2H4E	C2H4	168	50
2CHOCO	C2H4E	C3H6	172	50
2CHOCO	C3H6E	C3H6	158	50
3CH2OCO	2CHOCO	C2H4E	117	35
3CH2OCO	2CHOCO	C3H6E	118	35
3CH2OCO	C2H4E	C2H4	97	50
3CH2OCO	C2H4E	C3H6	105	50
3CH2OCO	C3H6E	C3H6	118	50
CHCH	C3H6	C2H4	159	35
C2H4E	C2H4	C3H6	174	35
C2H4E	C3H6	C3H6	164	35
C2H4	C3H6	C3H6	166	35
C3H6E	C3H6	C3H6	165	35
C3H6	CHCH	C3H6	101	35
C3H6	C2H4	C3H7T	178	35
C3H6	C3H6	C3H6	163	35
C3H6	C3H6	C3H7T	165	35

As shown in Table 1, the COGITO FF comprises nine different beads. In a LJ potential term, two parameters need to be assigned, σ and ε, that is, the distance at which the potential energy between two particles is 0 and the depth of the potential well, respectively. This amounts to a total of 18 parameters. Given that any bead can interact with any other bead, and the use of Lorentz–Berthelot rules^23,24^ to calculate the LJ potential of any bead pair, the LJ parameters for any single bead cannot be parameterized in isolation. Given this we decided to implement a Bayesian optimization (BO) approach, whereby all 18 parameters were varied and tested with each iteration. This approach has been taken before for the parameterization of new FFs, such as by McDonagh et al.^25^ and Sestito et al.^26^ however, to the best of our knowledge, not with so many different beads.

The BO was carried out using a top-down approach, that is, the cost (or deviation from the reference value) of each iteration was calculated with respect to empirical values. In this case, the empirical values were taken from known macroscopic values (densities and lattice parameters) of crystalline (in both β_2_ and β_1_ polymorphs) and melt *sn*-POSt, *sn*-POP, and *sn*-StOSt^9,27^ for a total of nine systems. The cost function was calculated as given in eq 2. This ad hoc equation was designed to (a) normalize all the values obtained from the MD simulations with respect to their reference empirical value, (b) avoid any individual cost value to be less than 1 by multiplying by 10, and (c) make sure that all costs were cumulative by squaring the result, and thus avoiding any negative individual cost values. Squaring of the cost term also makes it harmonic, that is, larger deviations from the reference values result in a quadratic increase in cost. This value was then multiplied by a weighting factor, which we determined for each metric according to the priority we gave to each. A list of the weightings and reference values is given in Supporting Information 4.
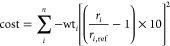
Equation 2: The cost function used for the BO. *i* = a given metric, *n* = total number of metrics, *wt* = assigned weight to any given metric, *r* = metric obtained from MD simulation, and *r*_*ref*_ = empirical value.

The BO was started by running 500 training iterations (i.e., 500 × 9 simulations) using random values within set limits for each LJ parameter, after which the 500 × 18 LJ parameters were used as the inputs to the BO, while the cost values were used as the output. The limits for each LJ parameter were based on the size of the bead (with the σ range being at higher values for larger beads) and the expected interaction of each bead (with the neutral aliphatic beads having a lower range for ε) (see Supporting Information 5 for more information). The density values were calculated over the last 3 ns of each simulation, while the *a*, *b*, and *c* dimensions and β angle of the simulation box were taken from the last frame of the simulation.

**Figure 2:**
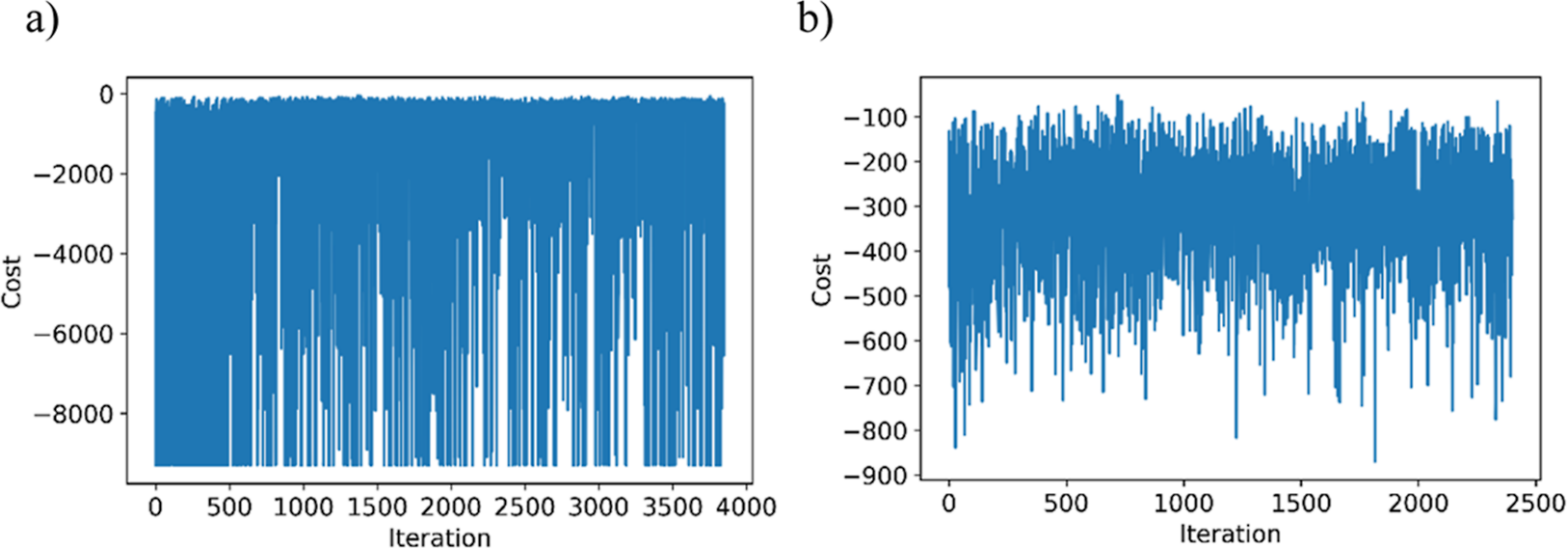
Plots of objective cost per iteration for all iterations carried out during the BO of the LJ parameters: (a) all iterations and (b) filtered for the iterations in which all simulations were completed.

The BO was then iterated through several thousand iterations to attempt to reduce the cost through optimization of the LJ parameters. This did not perform as well as expected, with a number of simulations crashing before completion of the 5 ns simulation time due to the automated choice of the LJ parameters, leading to a highly negative cost value (Figure 2a). Filtering for those simulations which did go to completion reduces the number of iterations from 3850 to 2401; however, the objective cost still did not show any convergence (Figure 2b).

Given this, the optimized LJ parameters were determined by filtering the results for (a) iterations which ran to completion, and (b) a cost which was below a specified value, and averaging the resulting parameter values. This filtering ensured that only the range of tested LJ parameters which gave the best results were analyzed, giving the parameters listed in Table 4.

**Table 4:** LJ Non-bonded Parameters for the COGITO FF^a^

bead name	σ (nm)	ε (kJ mol^–1^)
1CH2OCO	0.431	4.661
2CHOCO	0.442	4.781
3CH2OCO	0.434	4.374
CHCH	0.415	2.584
C2H4E	0.408	2.598
C2H4	0.412	2.384
C3H6E	0.386	2.800
C3H6	0.465	3.188
C3H7T	0.426	2.689

a Bayesian optimization of the non-bonded parameters.

### Parameter Validation Simulations

Having obtained a set of parameters for the COGITO FF based on the UA simulations using the NERD FF, XRD measurements, and optimization of the LJ potential parameters using BO by comparing against a number of physical properties, this parameter set was used to reproduce the macroscopic properties of different polymorphs of three pure TAGs.

**Figure 3:**
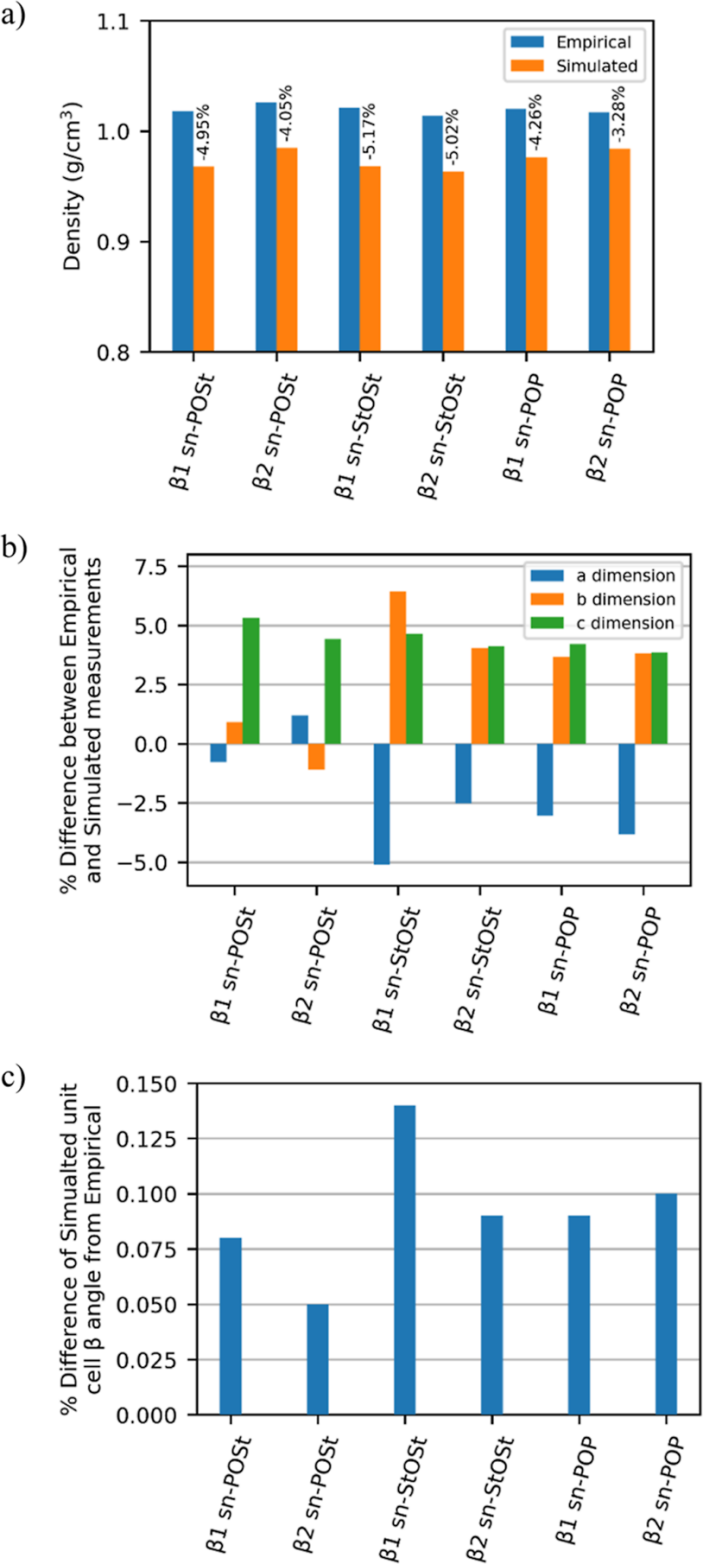
(a) Plot of empirical and simulated crystal densities of the β_1_ and β_2_ polymorphs of *sn*-POSt, *sn*-StOSt, and *sn*-POP. The values on top of the simulated column results show the relative % difference from empirical. (b) Plot showing relative % difference in unit cell dimensions, and (c) plot showing relative % difference in the unit cell β angle for the same six crystals.

Comparing the equilibration results obtained using the COGITO FF, built using the settings in Tables 2, 3, and 4, with empirical, the simulated results matched very well with empirical. Simulated densities varied from empirical by less than 6% in all six cases (Figure 3a), while the unit cell dimensions varied from empirical by less than 7% (Figure 3b). Moreover, the α and γ angles of the unit cells were reproduced at 90°, while the β angle varied minimally, with all unit cells varying by less 0.15% (Figure 3c). A full set of results is given in Supporting Information 6.

To test the validity of the non-bonded parameters, the radial distribution function (RDF) of all the possible bead combinations in both the melt and the crystalline states of *sn*-POSt (as a molecule which contains all nine bead types) were compared, giving a total of 90 different RDFs. The RDFs were calculated using the GROMACS^8^ gmx rdf program, where the atoms in the UA simulations were grouped by their center-of-geometry to correspond to the respective CG beads. The UA and CG RDFs overlap closely for both the melt and the crystalline structures (see Supporting Information 7), confirming the similar behavior in the UA and CG FFs.

### Testing the Reliability and Stability of the COGITO CG FF with Increasing Time-Steps

In general, the time-step used for a simulation should be about one-tenth of the time of the shortest period of motion.^28^ In an atomistic simulation, this is usually the C–H bond stretch, which is approximately 10 fs. This means that the time-step used should be of 1 fs. In the case of CG FFs, the weight of each bead is much heavier than a hydrogen atom, and hence, the period of motion will be much longer. This thus allows for the time-step used to be much longer, making the FF even more efficient. There is however still a balance to be struck. A shorter time-step gives more accurate results but requires a longer computation time to reach a specific target simulation time. A longer time-step gives greater efficiency, at the possible expense of accuracy. Choosing a time-step which is too long can lead to instabilities in the simulation.^28^ The limit of this newly developed FF was thus tested to determine the best accuracy-efficiency balance by equilibrating 100 molecules of β_2_*sn*-POSt crystal and *sn*-POSt melt at 280, 300, 325, and 350 K, using time-steps of 2, 10, 20, 30, 40, and 50 fs, for a duration of 50 ns. The simulations using the 40 and 50 fs time-steps all crashed immediately and were thus discarded. Some equilibrations using a 30 fs time-step also crashed, while all simulations using a 25 fs time-step, or shorter, all completed successfully. No significant difference was seen in the simulated density results going from a 2 fs time-step to a 25 fs time-step (Table 5). The complete results for robustness testing can be found in Supporting Information 8.

**Table 5:** Simulated Densities (g/cm^3^) of *sn*-POSt Using Different Equilibration Temperatures and Time-Steps for Robustness Testing of the COGITO FF

		time-step (fs)			
phase	temperature (*K*)	2	10	20	25
*sn*-POSt melt	280	0.925	0.924	0.925	0.925
	300	0.914	0.914	0.914	0.914
	325	0.900	0.900	0.901	0.901
	350	0.887	0.887	0.887	0.887
β_2_*sn*-POSt	280	0.971	0.972	0.972	0.971
	300	0.963	0.963	0.964	0.965
	325	0.949	0.951	0.953	0.951
	350	0.939	0.940	0.940	0.940

### Extending the FF to Other TAGs

The FF with the optimized parameters was then extended to other TAGs by using the same parameterized beads but changing the various topologies to allow the simulation of these TAGs, namely 1,2,3-trimyristoyl-*sn*-glycerol (*sn*-MMM, or trimyristin), 1,2,3-trioleoyl-*sn*-glycerol (*sn*-OOO, or triolein), 1,2,3-tripalmitoyl-*sn*-glycerol (*sn*-PPP, or tripalmitin), 1,2,3-tristearoyl-*sn*-glycerol (*sn*-StStSt, or tristearin), 1,3-dimyristoyl-2-oleoyl-*sn*-glycerol (*sn*-MOM), and 1-stearoyl-2-oleoyl-3-arachidoyl-*sn*-glycerol (*sn*-StOA). The various TAGs were mapped and are shown in Figure 1. Arachidic acid, ignoring the glycerol-ester bead, was mapped as -C2H4E–C2H4–C3H6–C3H6–C3H6–C3H6–C3H7T. These TAGs were chosen as empirical macroscopic data were available for all of them,^9,10,27^ thus making validation possible, as well as including a range of different fatty acid chain lengths (from 14 carbons for myristic acid to 20 carbons for arachidic acid) and including a mixture of fully saturated (such as *sn*-PPP), monounsaturated (such as *sn*-MOM), and polyunsaturated TAGs (such as *sn*-OOO).

**Figure 4:**
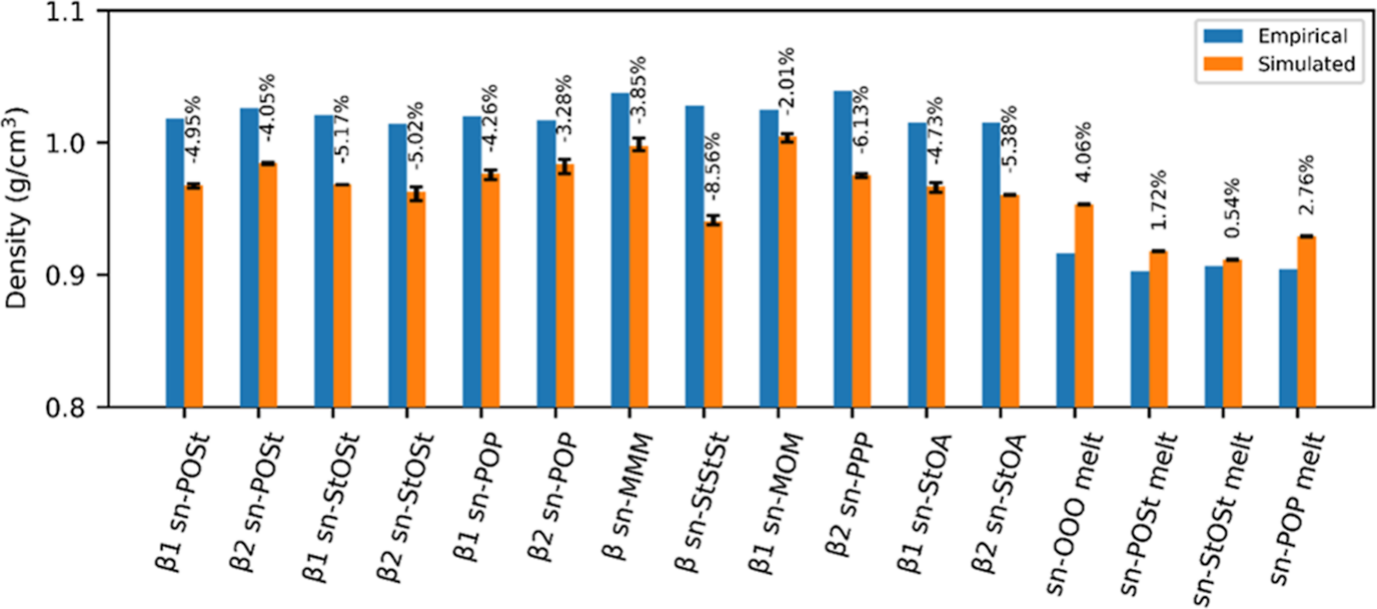
Plot of empirical and simulated densities for various TAGs in crystalline and melt phases. The values on top of the simulated column results show the relative % difference from empirical. Error bars = min/max density from three runs (where no error bars are shown, these are not available).

The empirical and simulated densities are compared in Figure 4. In all cases, the simulated densities differed from empirical by less than 6%, and the lower densities of the melt, compared to the crystalline phases of *sn*-POSt, *sn*-POP, and *sn*-StOSt, were reproduced. The unit cell dimensions and angles of all six other crystals (β *sn*-MMM, β *sn*-PPP, β *sn*-StStSt, β_1_*sn*-MOM, β_1_*sn*-StOA, and β_2_*sn*-StOA) were also reproduced, varying by less than 7% from empirical (see Supporting Information 6 for a full set of results). These results confirmed that the FF beads, as parameterized, are interchangeable, that is, the FF can predict the properties of molecules which were not used in the parameterization exercise accurately by simply building a new topology to represent a new molecule.

**Figure 5:**
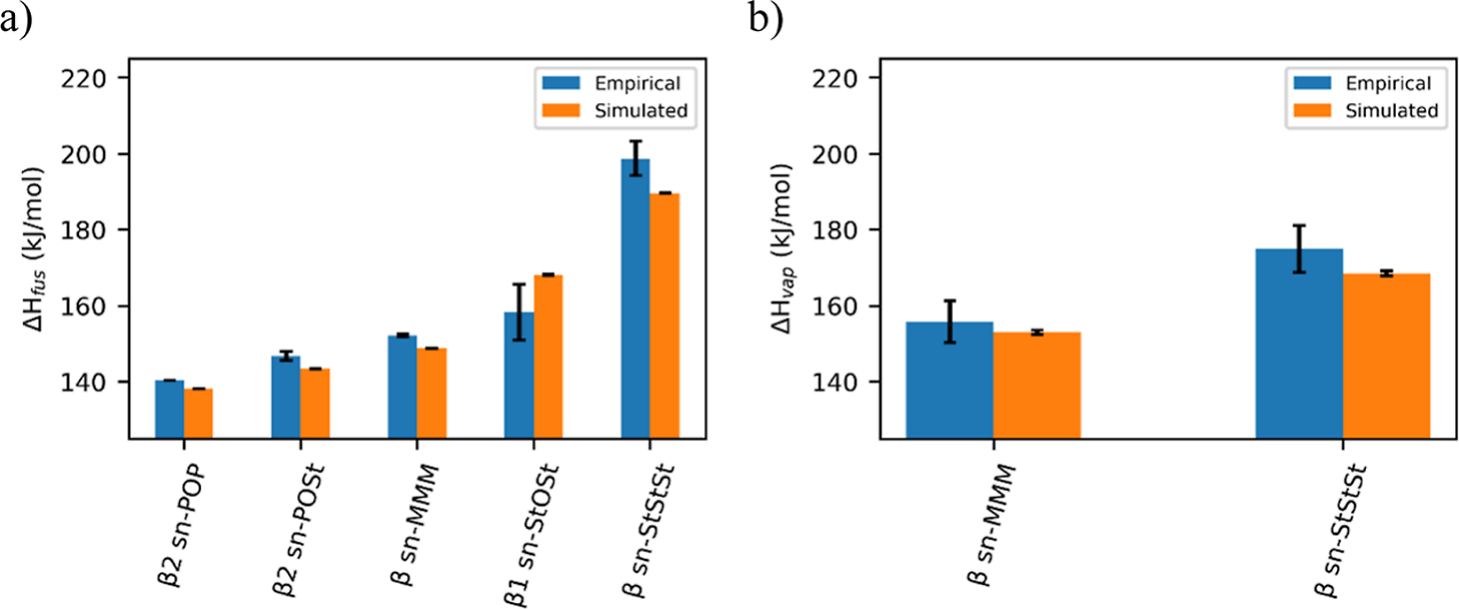
(a) Plot of empirical^31−35^ and simulated enthalpy of fusion (Δ*H*_fus_). Simulated values have been multiplied by 3.5 for better comparison. (b) Plot of empirical^19^ and simulated enthalpy of vaporization (Δ*H*_vap_). Simulated values have been divided by 1.5 for better comparison.

Comparing other, more dispersed empirical measurements for a selection of TAGs show good enthalpy trend reproduction, if offset by a factor (see Supporting Information 9 for individual run and aggregated data; the results shown in Figure 5 are from the aggregated data of 10 different runs for each TAG). From the results obtained, simulated Δ*H*_fus_ values are approximately 3.5 times lower than empirical, while simulated Δ*H*_vap_ values are approximately 1.5 times higher than empirical.^19^ This is expected given that the FF has not been parameterized against enthalpies, as is also found with other FFs. Martini 3 reports consistently low enthalpies of vaporization values,^18^ while Tsuchiya et al.^29^ and Jayaraman and Maginn^30^ struggle to replicate enthalpy of fusion trends for *n*-alkanes (using the PCFF FF) and those of different polymorphs of an ionic liquid (using the CMJJ FF), respectively.

## Conclusions

In this study, we have presented the COGITO FF, a newly parameterized FF for both saturated and unsaturated TAGs. We have developed this FF based on both empirical data and atomistic MD simulations. The CG simulations using the newly developed COGITO FF have been shown to reproduce the macroscopic properties (crystalline and melted density, and crystal shape and dimensions) of various TAGs accurately. The FF has also been shown to be flexible, giving good results for TAGs which were not used for the development of the FF but which were built from the defined building-block beads by interchanging the order in which they are placed when defining the TAG topology.

## Data Availability

PURE Dataset.zip can be found at 10.15129/9fd77c80-c43e-4f18-9cfc-420df756a36d consisting of GROMACS-compatible FF files, all topology files for all TAGs, starting configurations, Python script for BO and simulation analysis, associated csv files used as inputs in the Python script, all plots used in paper and SI, GROMACS system minimization parameter file, and example GROMACS system equilibration parameter file.

## Supporting Information Available

Python script to extract UA-bonded distances, angles, and dihedrals for *sn*-POSt; plots of potential energy (kJ/mol) versus torsion angle for four-body bead systems, using Centre of Geometries determined from the UA simulations; plots comparing UA (CG-mapped) bond distances with CG bond distances; Bayesian optimization metric reference values, weightings, and equilibration temperatures; chosen LJ parameter ranges for the Bayesian optimization; full set of simulated macroscopic properties of all TAGs; RDFs for all possible bead-type combinations for the melt and crystal of *sn*-POSt; simulated densities (g/cm^3^) of sn-POSt using different equilibration temperatures and time-steps for robustness testing of the COGITO FF; and simulated enthalpies of fusion and vaporization of TAGs (PDF)
