# Supplementary material for: COGITO: A Coarse-Grained Force Field for the Simulation of Macroscopic Properties of Triacylglycerides
Source: J Chem Theory Comput. 2023 Feb 2;19(4):1333–41. doi: 10.1021/acs.jctc.2c00975 (PMC9979597; doi:10.1021/acs.jctc.2c00975)
Supplement: Supplementary file 1 — ct2c00975_si_001.pdf [file ct2c00975_si_001.pdf]

Supporting Information

COGITO: A Coarse-Grained Force Field for the Simulation of Macroscopic Properties of Triacylglycerides

*Robert J. Cordina, Beccy Smith and Tell Tuttle**

[1] Mondelēz UK R&D Ltd., PO Box 12, Bournville Lane, Birmingham B30 2LU, UK.

[2] Department of Pure and Applied Chemistry, University of Strathclyde, 295 Cathedral Street, Glasgow G1 1XL, UK.

*tell.tuttle@strath.ac.uk

SI.1 *Python* s*cript to extract UA bonded distances, angles and dihedrals for* sn*-POSt*

```
#!/usr/bin/env python3
# -*- coding: utf-8 -*-


import mdtraj as md
import numpy as np
import time

start_time = time.time()

TAG = 'POS'
num_atoms = 6100

temperatures = np.array(['280_K', '300_K', '325_K', '350_K', '375_K', '400_K', '410_K'])
structure = np.array(['crystal', 'random'])
from_frame = 4000
num_frames = 1001
num_mols = 100

#############################################################
## DEFINING BEAD NAMES, BEAD ATOM INDICES AND ATOM WEIGHTS ##
#############################################################

# defining bead numbers (names) for POS with 4 atoms - format is last two digits are bead name, and prior digits are
the molecule number
bead_nums_4 = np.array([
111,     121,     131,     211,    221,      231,     311,     321,     331,     411,     421,     431,     511,
         521,     531,     611,     621,     631,     711,     721,     731,     811,     821,     831,     911,
         921,     931,     1011,    1021,    1031,    1111,    1121,    1131,    1211,    1221,    1231,    1311,
         1321,    1331,    1411,    1421,    1431,    1511,    1521,    1531,    1611,    1621,    1631,    1711,
         1721,    1731,    1811,    1821,    1831,    1911,    1921,    1931,    2011,    2021,    2031,    2111,
         2121,    2131,    2211,    2221,    2231,    2311,    2321,    2331,    2411,    2421,    2431,    2511,
         2521,    2531,    2611,    2621,    2631,    2711,    2721,    2731,    2811,    2821,    2831,    2911,
         2921,    2931,    3011,    3021,    3031,    3111,    3121,    3131,    3211,    3221,    3231,    3311,
         3321,    3331,    3411,    3421,    3431,    3511,    3521,    3531,    3611,    3621,    3631,    3711,
         3721,    3731,    3811,    3821,    3831,    3911,    3921,    3931,    4011,    4021,    4031,    4111,
         4121,    4131,    4211,    4221,    4231,    4311,    4321,    4331,    4411,    4421,    4431,    4511,
         4521,    4531,    4611,    4621,    4631,    4711,    4721,    4731,    4811,    4821,    4831,    4911,
         4921,    4931,    5011,    5021,    5031,    5111,    5121,    5131,    5211,    5221,    5231,    5311,
         5321,    5331,    5411,    5421,    5431,    5511,    5521,    5531,    5611,    5621,    5631,    5711,
         5721,    5731,    5811,    5821,    5831,    5911,    5921,    5931,    6011,    6021,    6031,    6111,
         6121,    6131,    6211,    6221,    6231,    6311,    6321,    6331,    6411,    6421,    6431,    6511,
         6521,    6531,    6611,    6621,    6631,    6711,    6721,    6731,    6811,    6821,    6831,    6911,
         6921,    6931,    7011,    7021,    7031,    7111,    7121,    7131,    7211,    7221,    7231,    7311,
         7321,    7331,    7411,    7421,    7431,    7511,    7521,    7531,    7611,    7621,    7631,    7711,
         7721,    7731,    7811,    7821,    7831,    7911,    7921,    7931,    8011,    8021,    8031,    8111,
         8121,    8131,    8211,    8221,    8231,    8311,    8321,    8331,    8411,    8421,    8431,    8511,
         8521,    8531,    8611,    8621,    8631,    8711,    8721,    8731,    8811,    8821,    8831,    8911,
         8921,    8931,    9011,    9021,    9031,    9111,    9121,    9131,    9211,    9221,    9231,    9311,
         9321,    9331,    9411,    9421,    9431,    9511,    9521,    9531,    9611,    9621,    9631,    9711,
         9721,    9731,    9811,    9821,    9831,    9911,    9921,    9931,    10011,   10021,   10031])

# defining bead numbers (names) for POS with 3 atoms - format is last two digits are bead name, and prior digits are
the molecule number
bead_nums_3 = np.array([
112,  113,  114,  115,  116,  124,  126,  128,  133,  134,  135,  136,  137,  212,  213,  214,  215,  216,  224,  226,
228,  233,  234,  235,  236,  237,  312,  313,  314,  315,  316,  324,  326,  328,  333,  334,  335,  336,  337,  412,
413,  414,  415,  416,  424,  426,  428,  433,  434,  435,  436,  437,  512,  513,  514,  515,  516,  524,  526,  528,
533,  534,  535,  536,  537,  612,  613,  614,  615,  616,  624,  626,  628,  633,  634,  635,  636,  637,  712,  713,
714,  715,  716,  724,  726,  728,  733,  734,  735,  736,  737,  812,  813,  814,  815,  816,  824,  826,  828,  833,
834,  835,  836,  837,  912,  913,  914,  915,  916,  924,  926,  928,  933,  934,  935,  936,  937,  1012,  1013,
1014,  1015,  1016,  1024,  1026,  1028,  1033,  1034,  1035,  1036,  1037,  1112,  1113,  1114,  1115,  1116,  1124,
1126,  1128,  1133,  1134,  1135,  1136,  1137,  1212,  1213,  1214,  1215,  1216,  1224,  1226,  1228,  1233,  1234,
1235,  1236,  1237,  1312,  1313,  1314,  1315,  1316,  1324,  1326,  1328,  1333,  1334,  1335,  1336,  1337,  1412,
1413,  1414,  1415,  1416,  1424,  1426,  1428,  1433,  1434,  1435,  1436,  1437,  1512,  1513,  1514,  1515,  1516,
1524,  1526,  1528,  1533,  1534,  1535,  1536,  1537,  1612,  1613,  1614,  1615,  1616,  1624,  1626,  1628,  1633,
1634,  1635,  1636,  1637,  1712,  1713,  1714,  1715,  1716,  1724,  1726,  1728,  1733,  1734,  1735,  1736,  1737,
1812,  1813,  1814,  1815,  1816,  1824,  1826,  1828,  1833,  1834,  1835,  1836,  1837,  1912,  1913,  1914,  1915,
1916,  1924,  1926,  1928,  1933,  1934,  1935,  1936,  1937,  2012,  2013,  2014,  2015,  2016,  2024,  2026,  2028,
2033,  2034,  2035,  2036,  2037,  2112,  2113,  2114,  2115,  2116,  2124,  2126,  2128,  2133,  2134,  2135,  2136,
2137,  2212,  2213,  2214,  2215,  2216,  2224,  2226,  2228,  2233,  2234,  2235,  2236,  2237,  2312,  2313,  2314,
2315,  2316,  2324,  2326,  2328,  2333,  2334,  2335,  2336,  2337,  2412,  2413,  2414,  2415,  2416,  2424,  2426,
2428,  2433,  2434,  2435,  2436,  2437,  2512,  2513,  2514,  2515,  2516,  2524,  2526,  2528,  2533,  2534,  2535,
2536,  2537,  2612,  2613,  2614,  2615,  2616,  2624,  2626,  2628,  2633,  2634,  2635,  2636,  2637,  2712,  2713,
2714,  2715,  2716,  2724,  2726,  2728,  2733,  2734,  2735,  2736,  2737,  2812,  2813,  2814,  2815,  2816,  2824,
2826,  2828,  2833,  2834,  2835,  2836,  2837,  2912,  2913,  2914,  2915,  2916,  2924,  2926,  2928,  2933,  2934,
2935,  2936,  2937,  3012,  3013,  3014,  3015,  3016,  3024,  3026,  3028,  3033,  3034,  3035,  3036,  3037,  3112,
3113,  3114,  3115,  3116,  3124,  3126,  3128,  3133,  3134,  3135,  3136,  3137,  3212,  3213,  3214,  3215,  3216,
```

```
3224,  3226,  3228,  3233,  3234,  3235,  3236,  3237,  3312,  3313,  3314,  3315,  3316,  3324,  3326,  3328,  3333,
3334,  3335,  3336,  3337,  3412,  3413,  3414,  3415,  3416,  3424,  3426,  3428,  3433,  3434,  3435,  3436,  3437,
3512,  3513,  3514,  3515,  3516,  3524,  3526,  3528,  3533,  3534,  3535,  3536,  3537,  3612,  3613,  3614,  3615,
3616,  3624,  3626,  3628,  3633,  3634,  3635,  3636,  3637,  3712,  3713,  3714,  3715,  3716,  3724,  3726,  3728,
3733,  3734,  3735,  3736,  3737,  3812,  3813,  3814,  3815,  3816,  3824,  3826,  3828,  3833,  3834,  3835,  3836,
3837,  3912,  3913,  3914,  3915,  3916,  3924,  3926,  3928,  3933,  3934,  3935,  3936,  3937,  4012,  4013,  4014,
4015,  4016,  4024,  4026,  4028,  4033,  4034,  4035,  4036,  4037,  4112,  4113,  4114,  4115,  4116,  4124,  4126,
4128,  4133,  4134,  4135,  4136,  4137,  4212,  4213,  4214,  4215,  4216,  4224,  4226,  4228,  4233,  4234,  4235,
4236,  4237,  4312,  4313,  4314,  4315,  4316,  4324,  4326,  4328,  4333,  4334,  4335,  4336,  4337,  4412,  4413,
4414,  4415,  4416,  4424,  4426,  4428,  4433,  4434,  4435,  4436,  4437,  4512,  4513,  4514,  4515,  4516,  4524,
4526,  4528,  4533,  4534,  4535,  4536,  4537,  4612,  4613,  4614,  4615,  4616,  4624,  4626,  4628,  4633,  4634,
4635,  4636,  4637,  4712,  4713,  4714,  4715,  4716,  4724,  4726,  4728,  4733,  4734,  4735,  4736,  4737,  4812,
4813,  4814,  4815,  4816,  4824,  4826,  4828,  4833,  4834,  4835,  4836,  4837,  4912,  4913,  4914,  4915,  4916,
4924,  4926,  4928,  4933,  4934,  4935,  4936,  4937,  5012,  5013,  5014,  5015,  5016,  5024,  5026,  5028,  5033,
5034,  5035,  5036,  5037,  5112,  5113,  5114,  5115,  5116,  5124,  5126,  5128,  5133,  5134,  5135,  5136,  5137,
5212,  5213,  5214,  5215,  5216,  5224,  5226,  5228,  5233,  5234,  5235,  5236,  5237,  5312,  5313,  5314,  5315,
5316,  5324,  5326,  5328,  5333,  5334,  5335,  5336,  5337,  5412,  5413,  5414,  5415,  5416,  5424,  5426,  5428,
5433,  5434,  5435,  5436,  5437,  5512,  5513,  5514,  5515,  5516,  5524,  5526,  5528,  5533,  5534,  5535,  5536,
5537,  5612,  5613,  5614,  5615,  5616,  5624,  5626,  5628,  5633,  5634,  5635,  5636,  5637,  5712,  5713,  5714,
5715,  5716,  5724,  5726,  5728,  5733,  5734,  5735,  5736,  5737,  5812,  5813,  5814,  5815,  5816,  5824,  5826,
5828,  5833,  5834,  5835,  5836,  5837,  5912,  5913,  5914,  5915,  5916,  5924,  5926,  5928,  5933,  5934,  5935,
5936,  5937,  6012,  6013,  6014,  6015,  6016,  6024,  6026,  6028,  6033,  6034,  6035,  6036,  6037,  6112,  6113,
6114,  6115,  6116,  6124,  6126,  6128,  6133,  6134,  6135,  6136,  6137,  6212,  6213,  6214,  6215,  6216,  6224,
6226,  6228,  6233,  6234,  6235,  6236,  6237,  6312,  6313,  6314,  6315,  6316,  6324,  6326,  6328,  6333,  6334,
6335,  6336,  6337,  6412,  6413,  6414,  6415,  6416,  6424,  6426,  6428,  6433,  6434,  6435,  6436,  6437,  6512,
6513,  6514,  6515,  6516,  6524,  6526,  6528,  6533,  6534,  6535,  6536,  6537,  6612,  6613,  6614,  6615,  6616,
6624,  6626,  6628,  6633,  6634,  6635,  6636,  6637,  6712,  6713,  6714,  6715,  6716,  6724,  6726,  6728,  6733,
6734,  6735,  6736,  6737,  6812,  6813,  6814,  6815,  6816,  6824,  6826,  6828,  6833,  6834,  6835,  6836,  6837,
6912,  6913,  6914,  6915,  6916,  6924,  6926,  6928,  6933,  6934,  6935,  6936,  6937,  7012,  7013,  7014,  7015,
7016,  7024,  7026,  7028,  7033,  7034,  7035,  7036,  7037,  7112,  7113,  7114,  7115,  7116,  7124,  7126,  7128,
7133,  7134,  7135,  7136,  7137,  7212,  7213,  7214,  7215,  7216,  7224,  7226,  7228,  7233,  7234,  7235,  7236,
7237,  7312,  7313,  7314,  7315,  7316,  7324,  7326,  7328,  7333,  7334,  7335,  7336,  7337,  7412,  7413,  7414,
7415,  7416,  7424,  7426,  7428,  7433,  7434,  7435,  7436,  7437,  7512,  7513,  7514,  7515,  7516,  7524,  7526,
7528,  7533,  7534,  7535,  7536,  7537,  7612,  7613,  7614,  7615,  7616,  7624,  7626,  7628,  7633,  7634,  7635,
7636,  7637,  7712,  7713,  7714,  7715,  7716,  7724,  7726,  7728,  7733,  7734,  7735,  7736,  7737,  7812,  7813,
7814,  7815,  7816,  7824,  7826,  7828,  7833,  7834,  7835,  7836,  7837,  7912,  7913,  7914,  7915,  7916,  7924,
7926,  7928,  7933,  7934,  7935,  7936,  7937,  8012,  8013,  8014,  8015,  8016,  8024,  8026,  8028,  8033,  8034,
8035,  8036,  8037,  8112,  8113,  8114,  8115,  8116,  8124,  8126,  8128,  8133,  8134,  8135,  8136,  8137,  8212,
8213,  8214,  8215,  8216,  8224,  8226,  8228,  8233,  8234,  8235,  8236,  8237,  8312,  8313,  8314,  8315,  8316,
8324,  8326,  8328,  8333,  8334,  8335,  8336,  8337,  8412,  8413,  8414,  8415,  8416,  8424,  8426,  8428,  8433,
8434,  8435,  8436,  8437,  8512,  8513,  8514,  8515,  8516,  8524,  8526,  8528,  8533,  8534,  8535,  8536,  8537,
8612,  8613,  8614,  8615,  8616,  8624,  8626,  8628,  8633,  8634,  8635,  8636,  8637,  8712,  8713,  8714,  8715,
8716,  8724,  8726,  8728,  8733,  8734,  8735,  8736,  8737,  8812,  8813,  8814,  8815,  8816,  8824,  8826,  8828,
8833,  8834,  8835,  8836,  8837,  8912,  8913,  8914,  8915,  8916,  8924,  8926,  8928,  8933,  8934,  8935,  8936,
8937,  9012,  9013,  9014,  9015,  9016,  9024,  9026,  9028,  9033,  9034,  9035,  9036,  9037,  9112,  9113,  9114,
9115,  9116,  9124,  9126,  9128,  9133,  9134,  9135,  9136,  9137,  9212,  9213,  9214,  9215,  9216,  9224,  9226,
9228,  9233,  9234,  9235,  9236,  9237,  9312,  9313,  9314,  9315,  9316,  9324,  9326,  9328,  9333,  9334,  9335,
9336,  9337,  9412,  9413,  9414,  9415,  9416,  9424,  9426,  9428,  9433,  9434,  9435,  9436,  9437,  9512,  9513,
9514,  9515,  9516,  9524,  9526,  9528,  9533,  9534,  9535,  9536,  9537,  9612,  9613,  9614,  9615,  9616,  9624,
9626,  9628,  9633,  9634,  9635,  9636,  9637,  9712,  9713,  9714,  9715,  9716,  9724,  9726,  9728,  9733,  9734,
9735,  9736,  9737,  9812,  9813,  9814,  9815,  9816,  9824,  9826,  9828,  9833,  9834,  9835,  9836,  9837,  9912,
9913,  9914,  9915,  9916,  9924,  9926,  9928,  9933,  9934,  9935,  9936,  9937,  10012,  10013,  10014,  10015,
10016,  10024,  10026,  10028,  10033,  10034,  10035,  10036,  10037  ])

# defining bead numbers (names) for POS with 2 atoms - format is last two digits are bead name, and prior digits are
the molecule number
bead_nums_2 = np.array([
122,  123,  125,  127,  132,  222,  223,  225,  227,  232,  322,  323,  325,  327,  332,  422,  423,  425,  427,  432,
522,  523,  525,  527,  532,  622,  623,  625,  627,  632,  722,  723,  725,  727,  732,  822,  823,  825,  827,  832,
922,  923,  925,  927,  932,  1022,  1023,  1025,  1027,  1032,  1122,  1123,  1125,  1127,  1132,  1222,  1223,  1225,
1227,  1232,  1322,  1323,  1325,  1327,  1332,  1422,  1423,  1425,  1427,  1432,  1522,  1523,  1525,  1527,  1532,
1622,  1623,  1625,  1627,  1632,  1722,  1723,  1725,  1727,  1732,  1822,  1823,  1825,  1827,  1832,  1922,  1923,
1925,  1927,  1932,  2022,  2023,  2025,  2027,  2032,  2122,  2123,  2125,  2127,  2132,  2222,  2223,  2225,  2227,
2232,  2322,  2323,  2325,  2327,  2332,  2422,  2423,  2425,  2427,  2432,  2522,  2523,  2525,  2527,  2532,  2622,
2623,  2625,  2627,  2632,  2722,  2723,  2725,  2727,  2732,  2822,  2823,  2825,  2827,  2832,  2922,  2923,  2925,
2927,  2932,  3022,  3023,  3025,  3027,  3032,  3122,  3123,  3125,  3127,  3132,  3222,  3223,  3225,  3227,  3232,
3322,  3323,  3325,  3327,  3332,  3422,  3423,  3425,  3427,  3432,  3522,  3523,  3525,  3527,  3532,  3622,  3623,
3625,  3627,  3632,  3722,  3723,  3725,  3727,  3732,  3822,  3823,  3825,  3827,  3832,  3922,  3923,  3925,  3927,
3932,  4022,  4023,  4025,  4027,  4032,  4122,  4123,  4125,  4127,  4132,  4222,  4223,  4225,  4227,  4232,  4322,
4323,  4325,  4327,  4332,  4422,  4423,  4425,  4427,  4432,  4522,  4523,  4525,  4527,  4532,  4622,  4623,  4625,
4627,  4632,  4722,  4723,  4725,  4727,  4732,  4822,  4823,  4825,  4827,  4832,  4922,  4923,  4925,  4927,  4932,
5022,  5023,  5025,  5027,  5032,  5122,  5123,  5125,  5127,  5132,  5222,  5223,  5225,  5227,  5232,  5322,  5323,
5325,  5327,  5332,  5422,  5423,  5425,  5427,  5432,  5522,  5523,  5525,  5527,  5532,  5622,  5623,  5625,  5627,
5632,  5722,  5723,  5725,  5727,  5732,  5822,  5823,  5825,  5827,  5832,  5922,  5923,  5925,  5927,  5932,  6022,
6023,  6025,  6027,  6032,  6122,  6123,  6125,  6127,  6132,  6222,  6223,  6225,  6227,  6232,  6322,  6323,  6325,
6327,  6332,  6422,  6423,  6425,  6427,  6432,  6522,  6523,  6525,  6527,  6532,  6622,  6623,  6625,  6627,  6632,
6722,  6723,  6725,  6727,  6732,  6822,  6823,  6825,  6827,  6832,  6922,  6923,  6925,  6927,  6932,  7022,  7023,
7025,  7027,  7032,  7122,  7123,  7125,  7127,  7132,  7222,  7223,  7225,  7227,  7232,  7322,  7323,  7325,  7327,
7332,  7422,  7423,  7425,  7427,  7432,  7522,  7523,  7525,  7527,  7532,  7622,  7623,  7625,  7627,  7632,  7722,
7723,  7725,  7727,  7732,  7822,  7823,  7825,  7827,  7832,  7922,  7923,  7925,  7927,  7932,  8022,  8023,  8025,
8027,  8032,  8122,  8123,  8125,  8127,  8132,  8222,  8223,  8225,  8227,  8232,  8322,  8323,  8325,  8327,  8332,
8422,  8423,  8425,  8427,  8432,  8522,  8523,  8525,  8527,  8532,  8622,  8623,  8625,  8627,  8632,  8722,  8723,
8725,  8727,  8732,  8822,  8823,  8825,  8827,  8832,  8922,  8923,  8925,  8927,  8932,  9022,  9023,  9025,  9027,
9032,  9122,  9123,  9125,  9127,  9132,  9222,  9223,  9225,  9227,  9232,  9322,  9323,  9325,  9327,  9332,  9422,
9423,  9425,  9427,  9432,  9522,  9523,  9525,  9527,  9532,  9622,  9623,  9625,  9627,  9632,  9722,  9723,  9725,
```

```
9727,  9732,  9822,  9823,  9825,  9827,  9832,  9922,  9923,  9925,  9927,  9932,  10022,  10023,  10025,  10027,
10032 ])

# defining Pythom atom indices for each bead as defined in bead_nums_4 with 4 atoms per bead
bead_nums_atoms_4 = np.array([
[0,1,2,3],   [5,6,7,8],   [10,11,12,13],   [61,62,63,64],   [66,67,68,69],   [71,72,73,74],   [122,123,124,125],
[127,128,129,130],  [132,133,134,135],  [183,184,185,186],  [188,189,190,191],  [193,194,195,196],  [244,245,246,247],
[249,250,251,252],  [254,255,256,257],  [305,306,307,308],  [310,311,312,313],  [315,316,317,318],  [366,367,368,369],
[371,372,373,374],  [376,377,378,379],  [427,428,429,430],  [432,433,434,435],  [437,438,439,440],  [488,489,490,491],
[493,494,495,496],  [498,499,500,501],  [549,550,551,552],  [554,555,556,557],  [559,560,561,562],  [610,611,612,613],
[615,616,617,618],  [620,621,622,623],  [671,672,673,674],  [676,677,678,679],  [681,682,683,684],  [732,733,734,735],
[737,738,739,740],  [742,743,744,745],  [793,794,795,796],  [798,799,800,801],  [803,804,805,806],  [854,855,856,857],
[859,860,861,862],  [864,865,866,867],  [915,916,917,918],  [920,921,922,923],  [925,926,927,928],  [976,977,978,979],
[981,982,983,984],   [986,987,988,989],   [1037,1038,1039,1040],   [1042,1043,1044,1045],   [1047,1048,1049,1050],
[1098,1099,1100,1101],  [1103,1104,1105,1106],  [1108,1109,1110,1111],  [1159,1160,1161,1162],  [1164,1165,1166,1167],
[1169,1170,1171,1172],  [1220,1221,1222,1223],  [1225,1226,1227,1228],  [1230,1231,1232,1233],  [1281,1282,1283,1284],
[1286,1287,1288,1289],  [1291,1292,1293,1294],  [1342,1343,1344,1345],  [1347,1348,1349,1350],  [1352,1353,1354,1355],
[1403,1404,1405,1406],  [1408,1409,1410,1411],  [1413,1414,1415,1416],  [1464,1465,1466,1467],  [1469,1470,1471,1472],
[1474,1475,1476,1477],  [1525,1526,1527,1528],  [1530,1531,1532,1533],  [1535,1536,1537,1538],  [1586,1587,1588,1589],
[1591,1592,1593,1594],  [1596,1597,1598,1599],  [1647,1648,1649,1650],  [1652,1653,1654,1655],  [1657,1658,1659,1660],
[1708,1709,1710,1711],  [1713,1714,1715,1716],  [1718,1719,1720,1721],  [1769,1770,1771,1772],  [1774,1775,1776,1777],
[1779,1780,1781,1782],  [1830,1831,1832,1833],  [1835,1836,1837,1838],  [1840,1841,1842,1843],  [1891,1892,1893,1894],
[1896,1897,1898,1899],  [1901,1902,1903,1904],  [1952,1953,1954,1955],  [1957,1958,1959,1960],  [1962,1963,1964,1965],
[2013,2014,2015,2016],  [2018,2019,2020,2021],  [2023,2024,2025,2026],  [2074,2075,2076,2077],  [2079,2080,2081,2082],
[2084,2085,2086,2087],  [2135,2136,2137,2138],  [2140,2141,2142,2143],  [2145,2146,2147,2148],  [2196,2197,2198,2199],
[2201,2202,2203,2204],  [2206,2207,2208,2209],  [2257,2258,2259,2260],  [2262,2263,2264,2265],  [2267,2268,2269,2270],
[2318,2319,2320,2321],  [2323,2324,2325,2326],  [2328,2329,2330,2331],  [2379,2380,2381,2382],  [2384,2385,2386,2387],
[2389,2390,2391,2392],  [2440,2441,2442,2443],  [2445,2446,2447,2448],  [2450,2451,2452,2453],  [2501,2502,2503,2504],
[2506,2507,2508,2509],  [2511,2512,2513,2514],  [2562,2563,2564,2565],  [2567,2568,2569,2570],  [2572,2573,2574,2575],
[2623,2624,2625,2626],  [2628,2629,2630,2631],  [2633,2634,2635,2636],  [2684,2685,2686,2687],  [2689,2690,2691,2692],
[2694,2695,2696,2697],  [2745,2746,2747,2748],  [2750,2751,2752,2753],  [2755,2756,2757,2758],  [2806,2807,2808,2809],
[2811,2812,2813,2814],  [2816,2817,2818,2819],  [2867,2868,2869,2870],  [2872,2873,2874,2875],  [2877,2878,2879,2880],
[2928,2929,2930,2931],  [2933,2934,2935,2936],  [2938,2939,2940,2941],  [2989,2990,2991,2992],  [2994,2995,2996,2997],
[2999,3000,3001,3002],  [3050,3051,3052,3053],  [3055,3056,3057,3058],  [3060,3061,3062,3063],  [3111,3112,3113,3114],
[3116,3117,3118,3119],  [3121,3122,3123,3124],  [3172,3173,3174,3175],  [3177,3178,3179,3180],  [3182,3183,3184,3185],
[3233,3234,3235,3236],  [3238,3239,3240,3241],  [3243,3244,3245,3246],  [3294,3295,3296,3297],  [3299,3300,3301,3302],
[3304,3305,3306,3307],  [3355,3356,3357,3358],  [3360,3361,3362,3363],  [3365,3366,3367,3368],  [3416,3417,3418,3419],
[3421,3422,3423,3424],  [3426,3427,3428,3429],  [3477,3478,3479,3480],  [3482,3483,3484,3485],  [3487,3488,3489,3490],
[3538,3539,3540,3541],  [3543,3544,3545,3546],  [3548,3549,3550,3551],  [3599,3600,3601,3602],  [3604,3605,3606,3607],
[3609,3610,3611,3612],  [3660,3661,3662,3663],  [3665,3666,3667,3668],  [3670,3671,3672,3673],  [3721,3722,3723,3724],
[3726,3727,3728,3729],  [3731,3732,3733,3734],  [3782,3783,3784,3785],  [3787,3788,3789,3790],  [3792,3793,3794,3795],
[3843,3844,3845,3846],  [3848,3849,3850,3851],  [3853,3854,3855,3856],  [3904,3905,3906,3907],  [3909,3910,3911,3912],
[3914,3915,3916,3917],  [3965,3966,3967,3968],  [3970,3971,3972,3973],  [3975,3976,3977,3978],  [4026,4027,4028,4029],
[4031,4032,4033,4034],  [4036,4037,4038,4039],  [4087,4088,4089,4090],  [4092,4093,4094,4095],  [4097,4098,4099,4100],
[4148,4149,4150,4151],  [4153,4154,4155,4156],  [4158,4159,4160,4161],  [4209,4210,4211,4212],  [4214,4215,4216,4217],
[4219,4220,4221,4222],  [4270,4271,4272,4273],  [4275,4276,4277,4278],  [4280,4281,4282,4283],  [4331,4332,4333,4334],
[4336,4337,4338,4339],  [4341,4342,4343,4344],  [4392,4393,4394,4395],  [4397,4398,4399,4400],  [4402,4403,4404,4405],
[4453,4454,4455,4456],  [4458,4459,4460,4461],  [4463,4464,4465,4466],  [4514,4515,4516,4517],  [4519,4520,4521,4522],
[4524,4525,4526,4527],  [4575,4576,4577,4578],  [4580,4581,4582,4583],  [4585,4586,4587,4588],  [4636,4637,4638,4639],
[4641,4642,4643,4644],  [4646,4647,4648,4649],  [4697,4698,4699,4700],  [4702,4703,4704,4705],  [4707,4708,4709,4710],
[4758,4759,4760,4761],  [4763,4764,4765,4766],  [4768,4769,4770,4771],  [4819,4820,4821,4822],  [4824,4825,4826,4827],
[4829,4830,4831,4832],  [4880,4881,4882,4883],  [4885,4886,4887,4888],  [4890,4891,4892,4893],  [4941,4942,4943,4944],
[4946,4947,4948,4949],  [4951,4952,4953,4954],  [5002,5003,5004,5005],  [5007,5008,5009,5010],  [5012,5013,5014,5015],
[5063,5064,5065,5066],  [5068,5069,5070,5071],  [5073,5074,5075,5076],  [5124,5125,5126,5127],  [5129,5130,5131,5132],
[5134,5135,5136,5137],  [5185,5186,5187,5188],  [5190,5191,5192,5193],  [5195,5196,5197,5198],  [5246,5247,5248,5249],
[5251,5252,5253,5254],  [5256,5257,5258,5259],  [5307,5308,5309,5310],  [5312,5313,5314,5315],  [5317,5318,5319,5320],
[5368,5369,5370,5371],  [5373,5374,5375,5376],  [5378,5379,5380,5381],  [5429,5430,5431,5432],  [5434,5435,5436,5437],
[5439,5440,5441,5442],  [5490,5491,5492,5493],  [5495,5496,5497,5498],  [5500,5501,5502,5503],  [5551,5552,5553,5554],
[5556,5557,5558,5559],  [5561,5562,5563,5564],  [5612,5613,5614,5615],  [5617,5618,5619,5620],  [5622,5623,5624,5625],
[5673,5674,5675,5676],  [5678,5679,5680,5681],  [5683,5684,5685,5686],  [5734,5735,5736,5737],  [5739,5740,5741,5742],
[5744,5745,5746,5747],  [5795,5796,5797,5798],  [5800,5801,5802,5803],  [5805,5806,5807,5808],  [5856,5857,5858,5859],
[5861,5862,5863,5864],  [5866,5867,5868,5869],  [5917,5918,5919,5920],  [5922,5923,5924,5925],  [5927,5928,5929,5930],
[5978,5979,5980,5981],  [5983,5984,5985,5986],  [5988,5989,5990,5991],  [6039,6040,6041,6042],  [6044,6045,6046,6047],
[6049,6050,6051,6052] ])

# defining Pythom atom indices for each bead as defined in bead_nums_3 with 3 atoms per bead
bead_nums_atoms_3 = np.array([
[4,15,16],  [17,18,19],  [20,21,22],  [23,24,25],  [26,27,28],  [32,33,34],  [37,38,39],  [42,43,44],  [46,47,48],
[49,50,51],  [52,53,54],  [55,56,57],  [58,59,60],  [65,76,77],  [78,79,80],  [81,82,83],  [84,85,86],  [87,88,89],
[93,94,95],   [98,99,100],   [103,104,105],   [107,108,109],   [110,111,112],   [113,114,115],   [116,117,118],
[119,120,121],   [126,137,138],   [139,140,141],   [142,143,144],   [145,146,147],   [148,149,150],   [154,155,156],
[159,160,161],   [164,165,166],   [168,169,170],   [171,172,173],   [174,175,176],   [177,178,179],   [180,181,182],
[187,198,199],   [200,201,202],   [203,204,205],   [206,207,208],   [209,210,211],   [215,216,217],   [220,221,222],
[225,226,227],   [229,230,231],   [232,233,234],   [235,236,237],   [238,239,240],   [241,242,243],   [248,259,260],
[261,262,263],   [264,265,266],   [267,268,269],   [270,271,272],   [276,277,278],   [281,282,283],   [286,287,288],
[290,291,292],   [293,294,295],   [296,297,298],   [299,300,301],   [302,303,304],   [309,320,321],   [322,323,324],
[325,326,327],   [328,329,330],   [331,332,333],   [337,338,339],   [342,343,344],   [347,348,349],   [351,352,353],
[354,355,356],   [357,358,359],   [360,361,362],   [363,364,365],   [370,381,382],   [383,384,385],   [386,387,388],
[389,390,391],   [392,393,394],   [398,399,400],   [403,404,405],   [408,409,410],   [412,413,414],   [415,416,417],
[418,419,420],   [421,422,423],   [424,425,426],   [431,442,443],   [444,445,446],   [447,448,449],   [450,451,452],
[453,454,455],   [459,460,461],   [464,465,466],   [469,470,471],   [473,474,475],   [476,477,478],   [479,480,481],
[482,483,484],   [485,486,487],   [492,503,504],   [505,506,507],   [508,509,510],   [511,512,513],   [514,515,516],
[520,521,522],   [525,526,527],   [530,531,532],   [534,535,536],   [537,538,539],   [540,541,542],   [543,544,545],
[546,547,548],   [553,564,565],   [566,567,568],   [569,570,571],   [572,573,574],   [575,576,577],   [581,582,583],
[586,587,588],   [591,592,593],   [595,596,597],   [598,599,600],   [601,602,603],   [604,605,606],   [607,608,609],
```

[614,625,626], [627,628,629], [630,631,632], [633,634,635], [636,637,638], [642,643,644], [647,648,649],
[652,653,654], [656,657,658], [659,660,661], [662,663,664], [665,666,667], [668,669,670], [675,686,687],
[688,689,690], [691,692,693], [694,695,696], [697,698,699], [703,704,705], [708,709,710], [713,714,715],
[717,718,719], [720,721,722], [723,724,725], [726,727,728], [729,730,731], [736,747,748], [749,750,751],
[752,753,754], [755,756,757], [758,759,760], [764,765,766], [769,770,771], [774,775,776], [778,779,780],
[781,782,783], [784,785,786], [787,788,789], [790,791,792], [797,808,809], [810,811,812], [813,814,815],
[816,817,818], [819,820,821], [825,826,827], [830,831,832], [835,836,837], [839,840,841], [842,843,844],
[845,846,847], [848,849,850], [851,852,853], [858,869,870], [871,872,873], [874,875,876], [877,878,879],
[880,881,882], [886,887,888], [891,892,893], [896,897,898], [900,901,902], [903,904,905], [906,907,908],
[909,910,911], [912,913,914], [919,930,931], [932,933,934], [935,936,937], [938,939,940], [941,942,943],
[947,948,949], [952,953,954], [957,958,959], [961,962,963], [964,965,966], [967,968,969], [970,971,972],
[973,974,975], [980,991,992], [993,994,995], [996,997,998], [999,1000,1001], [1002,1003,1004], [1008,1009,1010],
[1013,1014,1015], [1018,1019,1020], [1022,1023,1024], [1025,1026,1027], [1028,1029,1030], [1031,1032,1033],
[1034,1035,1036], [1041,1052,1053], [1054,1055,1056], [1057,1058,1059], [1060,1061,1062], [1063,1064,1065],
[1069,1070,1071], [1074,1075,1076], [1079,1080,1081], [1083,1084,1085], [1086,1087,1088], [1089,1090,1091],
[1092,1093,1094], [1095,1096,1097], [1102,1113,1114], [1115,1116,1117], [1118,1119,1120], [1121,1122,1123],
[1124,1125,1126], [1130,1131,1132], [1135,1136,1137], [1140,1141,1142], [1144,1145,1146], [1147,1148,1149],
[1150,1151,1152], [1153,1154,1155], [1156,1157,1158], [1163,1174,1175], [1176,1177,1178], [1179,1180,1181],
[1182,1183,1184], [1185,1186,1187], [1191,1192,1193], [1196,1197,1198], [1201,1202,1203], [1205,1206,1207],
[1208,1209,1210], [1211,1212,1213], [1214,1215,1216], [1217,1218,1219], [1224,1235,1236], [1237,1238,1239],
[1240,1241,1242], [1243,1244,1245], [1246,1247,1248], [1252,1253,1254], [1257,1258,1259], [1262,1263,1264],
[1266,1267,1268], [1269,1270,1271], [1272,1273,1274], [1275,1276,1277], [1278,1279,1280], [1285,1296,1297],
[1298,1299,1300], [1301,1302,1303], [1304,1305,1306], [1307,1308,1309], [1313,1314,1315], [1318,1319,1320],
[1323,1324,1325], [1327,1328,1329], [1330,1331,1332], [1333,1334,1335], [1336,1337,1338], [1339,1340,1341],
[1346,1357,1358], [1359,1360,1361], [1362,1363,1364], [1365,1366,1367], [1368,1369,1370], [1374,1375,1376],
[1379,1380,1381], [1384,1385,1386], [1388,1389,1390], [1391,1392,1393], [1394,1395,1396], [1397,1398,1399],
[1400,1401,1402], [1407,1418,1419], [1420,1421,1422], [1423,1424,1425], [1426,1427,1428], [1429,1430,1431],
[1435,1436,1437], [1440,1441,1442], [1445,1446,1447], [1449,1450,1451], [1452,1453,1454], [1455,1456,1457],
[1458,1459,1460], [1461,1462,1463], [1468,1479,1480], [1481,1482,1483], [1484,1485,1486], [1487,1488,1489],
[1490,1491,1492], [1496,1497,1498], [1501,1502,1503], [1506,1507,1508], [1510,1511,1512], [1513,1514,1515],
[1516,1517,1518], [1519,1520,1521], [1522,1523,1524], [1529,1540,1541], [1542,1543,1544], [1545,1546,1547],
[1548,1549,1550], [1551,1552,1553], [1557,1558,1559], [1562,1563,1564], [1567,1568,1569], [1571,1572,1573],
[1574,1575,1576], [1577,1578,1579], [1580,1581,1582], [1583,1584,1585], [1590,1601,1602], [1603,1604,1605],
[1606,1607,1608], [1609,1610,1611], [1612,1613,1614], [1618,1619,1620], [1623,1624,1625], [1628,1629,1630],
[1632,1633,1634], [1635,1636,1637], [1638,1639,1640], [1641,1642,1643], [1644,1645,1646], [1651,1662,1663],
[1664,1665,1666], [1667,1668,1669], [1670,1671,1672], [1673,1674,1675], [1679,1680,1681], [1684,1685,1686],
[1689,1690,1691], [1693,1694,1695], [1696,1697,1698], [1699,1700,1701], [1702,1703,1704], [1705,1706,1707],
[1712,1723,1724], [1725,1726,1727], [1728,1729,1730], [1731,1732,1733], [1734,1735,1736], [1740,1741,1742],
[1745,1746,1747], [1750,1751,1752], [1754,1755,1756], [1757,1758,1759], [1760,1761,1762], [1763,1764,1765],
[1766,1767,1768], [1773,1784,1785], [1786,1787,1788], [1789,1790,1791], [1792,1793,1794], [1795,1796,1797],
[1801,1802,1803], [1806,1807,1808], [1811,1812,1813], [1815,1816,1817], [1818,1819,1820], [1821,1822,1823],
[1824,1825,1826], [1827,1828,1829], [1834,1845,1846], [1847,1848,1849], [1850,1851,1852], [1853,1854,1855],
[1856,1857,1858], [1862,1863,1864], [1867,1868,1869], [1872,1873,1874], [1876,1877,1878], [1879,1880,1881],
[1882,1883,1884], [1885,1886,1887], [1888,1889,1890], [1895,1906,1907], [1908,1909,1910], [1911,1912,1913],
[1914,1915,1916], [1917,1918,1919], [1923,1924,1925], [1928,1929,1930], [1933,1934,1935], [1937,1938,1939],
[1940,1941,1942], [1943,1944,1945], [1946,1947,1948], [1949,1950,1951], [1956,1967,1968], [1969,1970,1971],
[1972,1973,1974], [1975,1976,1977], [1978,1979,1980], [1984,1985,1986], [1989,1990,1991], [1994,1995,1996],
[1998,1999,2000], [2001,2002,2003], [2004,2005,2006], [2007,2008,2009], [2010,2011,2012], [2017,2028,2029],
[2030,2031,2032], [2033,2034,2035], [2036,2037,2038], [2039,2040,2041], [2045,2046,2047], [2050,2051,2052],
[2055,2056,2057], [2059,2060,2061], [2062,2063,2064], [2065,2066,2067], [2068,2069,2070], [2071,2072,2073],
[2078,2089,2090], [2091,2092,2093], [2094,2095,2096], [2097,2098,2099], [2100,2101,2102], [2106,2107,2108],
[2111,2112,2113], [2116,2117,2118], [2120,2121,2122], [2123,2124,2125], [2126,2127,2128], [2129,2130,2131],
[2132,2133,2134], [2139,2150,2151], [2152,2153,2154], [2155,2156,2157], [2158,2159,2160], [2161,2162,2163],
[2167,2168,2169], [2172,2173,2174], [2177,2178,2179], [2181,2182,2183], [2184,2185,2186], [2187,2188,2189],
[2190,2191,2192], [2193,2194,2195], [2200,2211,2212], [2213,2214,2215], [2216,2217,2218], [2219,2220,2221],
[2222,2223,2224], [2228,2229,2230], [2233,2234,2235], [2238,2239,2240], [2242,2243,2244], [2245,2246,2247],
[2248,2249,2250], [2251,2252,2253], [2254,2255,2256], [2261,2272,2273], [2274,2275,2276], [2277,2278,2279],
[2280,2281,2282], [2283,2284,2285], [2289,2290,2291], [2294,2295,2296], [2299,2300,2301], [2303,2304,2305],
[2306,2307,2308], [2309,2310,2311], [2312,2313,2314], [2315,2316,2317], [2322,2333,2334], [2335,2336,2337],
[2338,2339,2340], [2341,2342,2343], [2344,2345,2346], [2350,2351,2352], [2355,2356,2357], [2360,2361,2362],
[2364,2365,2366], [2367,2368,2369], [2370,2371,2372], [2373,2374,2375], [2376,2377,2378], [2383,2394,2395],
[2396,2397,2398], [2399,2400,2401], [2402,2403,2404], [2405,2406,2407], [2411,2412,2413], [2416,2417,2418],
[2421,2422,2423], [2425,2426,2427], [2428,2429,2430], [2431,2432,2433], [2434,2435,2436], [2437,2438,2439],
[2444,2455,2456], [2457,2458,2459], [2460,2461,2462], [2463,2464,2465], [2466,2467,2468], [2472,2473,2474],
[2477,2478,2479], [2482,2483,2484], [2486,2487,2488], [2489,2490,2491], [2492,2493,2494], [2495,2496,2497],
[2498,2499,2500], [2505,2516,2517], [2518,2519,2520], [2521,2522,2523], [2524,2525,2526], [2527,2528,2529],
[2533,2534,2535], [2538,2539,2540], [2543,2544,2545], [2547,2548,2549], [2550,2551,2552], [2553,2554,2555],
[2556,2557,2558], [2559,2560,2561], [2566,2577,2578], [2579,2580,2581], [2582,2583,2584], [2585,2586,2587],
[2588,2589,2590], [2594,2595,2596], [2599,2600,2601], [2604,2605,2606], [2608,2609,2610], [2611,2612,2613],
[2614,2615,2616], [2617,2618,2619], [2620,2621,2622], [2627,2638,2639], [2640,2641,2642], [2643,2644,2645],
[2646,2647,2648], [2649,2650,2651], [2655,2656,2657], [2660,2661,2662], [2665,2666,2667], [2669,2670,2671],
[2672,2673,2674], [2675,2676,2677], [2678,2679,2680], [2681,2682,2683], [2688,2699,2700], [2701,2702,2703],
[2704,2705,2706], [2707,2708,2709], [2710,2711,2712], [2716,2717,2718], [2721,2722,2723], [2726,2727,2728],
[2730,2731,2732], [2733,2734,2735], [2736,2737,2738], [2739,2740,2741], [2742,2743,2744], [2749,2760,2761],
[2762,2763,2764], [2765,2766,2767], [2768,2769,2770], [2771,2772,2773], [2777,2778,2779], [2782,2783,2784],
[2787,2788,2789], [2791,2792,2793], [2794,2795,2796], [2797,2798,2799], [2800,2801,2802], [2803,2804,2805],
[2810,2821,2822], [2823,2824,2825], [2826,2827,2828], [2829,2830,2831], [2832,2833,2834], [2838,2839,2840],
[2843,2844,2845], [2848,2849,2850], [2852,2853,2854], [2855,2856,2857], [2858,2859,2860], [2861,2862,2863],
[2864,2865,2866], [2871,2882,2883], [2884,2885,2886], [2887,2888,2889], [2890,2891,2892], [2893,2894,2895],
[2899,2900,2901], [2904,2905,2906], [2909,2910,2911], [2913,2914,2915], [2916,2917,2918], [2919,2920,2921],
[2922,2923,2924], [2925,2926,2927], [2932,2943,2944], [2945,2946,2947], [2948,2949,2950], [2951,2952,2953],
[2954,2955,2956], [2960,2961,2962], [2965,2966,2967], [2970,2971,2972], [2974,2975,2976], [2977,2978,2979],
[2980,2981,2982], [2983,2984,2985], [2986,2987,2988], [2993,3004,3005], [3006,3007,3008], [3009,3010,3011],
[3012,3013,3014], [3015,3016,3017], [3021,3022,3023], [3026,3027,3028], [3031,3032,3033], [3035,3036,3037],
[3038,3039,3040], [3041,3042,3043], [3044,3045,3046], [3047,3048,3049], [3054,3065,3066], [3067,3068,3069],

[3070,3071,3072], [3073,3074,3075], [3076,3077,3078], [3082,3083,3084], [3087,3088,3089], [3092,3093,3094],
[3096,3097,3098], [3099,3100,3101], [3102,3103,3104], [3105,3106,3107], [3108,3109,3110], [3115,3126,3127],
[3128,3129,3130], [3131,3132,3133], [3134,3135,3136], [3137,3138,3139], [3143,3144,3145], [3148,3149,3150],
[3153,3154,3155], [3157,3158,3159], [3160,3161,3162], [3163,3164,3165], [3166,3167,3168], [3169,3170,3171],
[3176,3187,3188], [3189,3190,3191], [3192,3193,3194], [3195,3196,3197], [3198,3199,3200], [3204,3205,3206],
[3209,3210,3211], [3214,3215,3216], [3218,3219,3220], [3221,3222,3223], [3224,3225,3226], [3227,3228,3229],
[3230,3231,3232], [3237,3248,3249], [3250,3251,3252], [3253,3254,3255], [3256,3257,3258], [3259,3260,3261],
[3265,3266,3267], [3270,3271,3272], [3275,3276,3277], [3279,3280,3281], [3282,3283,3284], [3285,3286,3287],
[3288,3289,3290], [3291,3292,3293], [3298,3309,3310], [3311,3312,3313], [3314,3315,3316], [3317,3318,3319],
[3320,3321,3322], [3326,3327,3328], [3331,3332,3333], [3336,3337,3338], [3340,3341,3342], [3343,3344,3345],
[3346,3347,3348], [3349,3350,3351], [3352,3353,3354], [3359,3370,3371], [3372,3373,3374], [3375,3376,3377],
[3378,3379,3380], [3381,3382,3383], [3387,3388,3389], [3392,3393,3394], [3397,3398,3399], [3401,3402,3403],
[3404,3405,3406], [3407,3408,3409], [3410,3411,3412], [3413,3414,3415], [3420,3431,3432], [3433,3434,3435],
[3436,3437,3438], [3439,3440,3441], [3442,3443,3444], [3448,3449,3450], [3453,3454,3455], [3458,3459,3460],
[3462,3463,3464], [3465,3466,3467], [3468,3469,3470], [3471,3472,3473], [3474,3475,3476], [3481,3492,3493],
[3494,3495,3496], [3497,3498,3499], [3500,3501,3502], [3503,3504,3505], [3509,3510,3511], [3514,3515,3516],
[3519,3520,3521], [3523,3524,3525], [3526,3527,3528], [3529,3530,3531], [3532,3533,3534], [3535,3536,3537],
[3542,3553,3554], [3555,3556,3557], [3558,3559,3560], [3561,3562,3563], [3564,3565,3566], [3570,3571,3572],
[3575,3576,3577], [3580,3581,3582], [3584,3585,3586], [3587,3588,3589], [3590,3591,3592], [3593,3594,3595],
[3596,3597,3598], [3603,3614,3615], [3616,3617,3618], [3619,3620,3621], [3622,3623,3624], [3625,3626,3627],
[3631,3632,3633], [3636,3637,3638], [3641,3642,3643], [3645,3646,3647], [3648,3649,3650], [3651,3652,3653],
[3654,3655,3656], [3657,3658,3659], [3664,3675,3676], [3677,3678,3679], [3680,3681,3682], [3683,3684,3685],
[3686,3687,3688], [3692,3693,3694], [3697,3698,3699], [3702,3703,3704], [3706,3707,3708], [3709,3710,3711],
[3712,3713,3714], [3715,3716,3717], [3718,3719,3720], [3725,3736,3737], [3738,3739,3740], [3741,3742,3743],
[3744,3745,3746], [3747,3748,3749], [3753,3754,3755], [3758,3759,3760], [3763,3764,3765], [3767,3768,3769],
[3770,3771,3772], [3773,3774,3775], [3776,3777,3778], [3779,3780,3781], [3786,3797,3798], [3799,3800,3801],
[3802,3803,3804], [3805,3806,3807], [3808,3809,3810], [3814,3815,3816], [3819,3820,3821], [3824,3825,3826],
[3828,3829,3830], [3831,3832,3833], [3834,3835,3836], [3837,3838,3839], [3840,3841,3842], [3847,3858,3859],
[3860,3861,3862], [3863,3864,3865], [3866,3867,3868], [3869,3870,3871], [3875,3876,3877], [3880,3881,3882],
[3885,3886,3887], [3889,3890,3891], [3892,3893,3894], [3895,3896,3897], [3898,3899,3900], [3901,3902,3903],
[3908,3919,3920], [3921,3922,3923], [3924,3925,3926], [3927,3928,3929], [3930,3931,3932], [3936,3937,3938],
[3941,3942,3943], [3946,3947,3948], [3950,3951,3952], [3953,3954,3955], [3956,3957,3958], [3959,3960,3961],
[3962,3963,3964], [3969,3980,3981], [3982,3983,3984], [3985,3986,3987], [3988,3989,3990], [3991,3992,3993],
[3997,3998,3999], [4002,4003,4004], [4007,4008,4009], [4011,4012,4013], [4014,4015,4016], [4017,4018,4019],
[4020,4021,4022], [4023,4024,4025], [4030,4041,4042], [4043,4044,4045], [4046,4047,4048], [4049,4050,4051],
[4052,4053,4054], [4058,4059,4060], [4063,4064,4065], [4068,4069,4070], [4072,4073,4074], [4075,4076,4077],
[4078,4079,4080], [4081,4082,4083], [4084,4085,4086], [4091,4102,4103], [4104,4105,4106], [4107,4108,4109],
[4110,4111,4112], [4113,4114,4115], [4119,4120,4121], [4124,4125,4126], [4129,4130,4131], [4133,4134,4135],
[4136,4137,4138], [4139,4140,4141], [4142,4143,4144], [4145,4146,4147], [4152,4163,4164], [4165,4166,4167],
[4168,4169,4170], [4171,4172,4173], [4174,4175,4176], [4180,4181,4182], [4185,4186,4187], [4190,4191,4192],
[4194,4195,4196], [4197,4198,4199], [4200,4201,4202], [4203,4204,4205], [4206,4207,4208], [4213,4224,4225],
[4226,4227,4228], [4229,4230,4231], [4232,4233,4234], [4235,4236,4237], [4241,4242,4243], [4246,4247,4248],
[4251,4252,4253], [4255,4256,4257], [4258,4259,4260], [4261,4262,4263], [4264,4265,4266], [4267,4268,4269],
[4274,4285,4286], [4287,4288,4289], [4290,4291,4292], [4293,4294,4295], [4296,4297,4298], [4302,4303,4304],
[4307,4308,4309], [4312,4313,4314], [4316,4317,4318], [4319,4320,4321], [4322,4323,4324], [4325,4326,4327],
[4328,4329,4330], [4335,4346,4347], [4348,4349,4350], [4351,4352,4353], [4354,4355,4356], [4357,4358,4359],
[4363,4364,4365], [4368,4369,4370], [4373,4374,4375], [4377,4378,4379], [4380,4381,4382], [4383,4384,4385],
[4386,4387,4388], [4389,4390,4391], [4396,4407,4408], [4409,4410,4411], [4412,4413,4414], [4415,4416,4417],
[4418,4419,4420], [4424,4425,4426], [4429,4430,4431], [4434,4435,4436], [4438,4439,4440], [4441,4442,4443],
[4444,4445,4446], [4447,4448,4449], [4450,4451,4452], [4457,4468,4469], [4470,4471,4472], [4473,4474,4475],
[4476,4477,4478], [4479,4480,4481], [4485,4486,4487], [4490,4491,4492], [4495,4496,4497], [4499,4500,4501],
[4502,4503,4504], [4505,4506,4507], [4508,4509,4510], [4511,4512,4513], [4518,4529,4530], [4531,4532,4533],
[4534,4535,4536], [4537,4538,4539], [4540,4541,4542], [4546,4547,4548], [4551,4552,4553], [4556,4557,4558],
[4560,4561,4562], [4563,4564,4565], [4566,4567,4568], [4569,4570,4571], [4572,4573,4574], [4579,4590,4591],
[4592,4593,4594], [4595,4596,4597], [4598,4599,4600], [4601,4602,4603], [4607,4608,4609], [4612,4613,4614],
[4617,4618,4619], [4621,4622,4623], [4624,4625,4626], [4627,4628,4629], [4630,4631,4632], [4633,4634,4635],
[4640,4651,4652], [4653,4654,4655], [4656,4657,4658], [4659,4660,4661], [4662,4663,4664], [4668,4669,4670],
[4673,4674,4675], [4678,4679,4680], [4682,4683,4684], [4685,4686,4687], [4688,4689,4690], [4691,4692,4693],
[4694,4695,4696], [4701,4712,4713], [4714,4715,4716], [4717,4718,4719], [4720,4721,4722], [4723,4724,4725],
[4729,4730,4731], [4734,4735,4736], [4739,4740,4741], [4743,4744,4745], [4746,4747,4748], [4749,4750,4751],
[4752,4753,4754], [4755,4756,4757], [4762,4773,4774], [4775,4776,4777], [4778,4779,4780], [4781,4782,4783],
[4784,4785,4786], [4790,4791,4792], [4795,4796,4797], [4800,4801,4802], [4804,4805,4806], [4807,4808,4809],
[4810,4811,4812], [4813,4814,4815], [4816,4817,4818], [4823,4834,4835], [4836,4837,4838], [4839,4840,4841],
[4842,4843,4844], [4845,4846,4847], [4851,4852,4853], [4856,4857,4858], [4861,4862,4863], [4865,4866,4867],
[4868,4869,4870], [4871,4872,4873], [4874,4875,4876], [4877,4878,4879], [4884,4895,4896], [4897,4898,4899],
[4900,4901,4902], [4903,4904,4905], [4906,4907,4908], [4912,4913,4914], [4917,4918,4919], [4922,4923,4924],
[4926,4927,4928], [4929,4930,4931], [4932,4933,4934], [4935,4936,4937], [4938,4939,4940], [4945,4956,4957],
[4958,4959,4960], [4961,4962,4963], [4964,4965,4966], [4967,4968,4969], [4973,4974,4975], [4978,4979,4980],
[4983,4984,4985], [4987,4988,4989], [4990,4991,4992], [4993,4994,4995], [4996,4997,4998], [4999,5000,5001],
[5006,5017,5018], [5019,5020,5021], [5022,5023,5024], [5025,5026,5027], [5028,5029,5030], [5034,5035,5036],
[5039,5040,5041], [5044,5045,5046], [5048,5049,5050], [5051,5052,5053], [5054,5055,5056], [5057,5058,5059],
[5060,5061,5062], [5067,5078,5079], [5080,5081,5082], [5083,5084,5085], [5086,5087,5088], [5089,5090,5091],
[5095,5096,5097], [5100,5101,5102], [5105,5106,5107], [5109,5110,5111], [5112,5113,5114], [5115,5116,5117],
[5118,5119,5120], [5121,5122,5123], [5128,5139,5140], [5141,5142,5143], [5144,5145,5146], [5147,5148,5149],
[5150,5151,5152], [5156,5157,5158], [5161,5162,5163], [5166,5167,5168], [5170,5171,5172], [5173,5174,5175],
[5176,5177,5178], [5179,5180,5181], [5182,5183,5184], [5189,5200,5201], [5202,5203,5204], [5205,5206,5207],
[5208,5209,5210], [5211,5212,5213], [5217,5218,5219], [5222,5223,5224], [5227,5228,5229], [5231,5232,5233],
[5234,5235,5236], [5237,5238,5239], [5240,5241,5242], [5243,5244,5245], [5250,5261,5262], [5263,5264,5265],
[5266,5267,5268], [5269,5270,5271], [5272,5273,5274], [5278,5279,5280], [5283,5284,5285], [5288,5289,5290],
[5292,5293,5294], [5295,5296,5297], [5298,5299,5300], [5301,5302,5303], [5304,5305,5306], [5311,5322,5323],
[5324,5325,5326], [5327,5328,5329], [5330,5331,5332], [5333,5334,5335], [5339,5340,5341], [5344,5345,5346],
[5349,5350,5351], [5353,5354,5355], [5356,5357,5358], [5359,5360,5361], [5362,5363,5364], [5365,5366,5367],
[5372,5383,5384], [5385,5386,5387], [5388,5389,5390], [5391,5392,5393], [5394,5395,5396], [5400,5401,5402],
[5405,5406,5407], [5410,5411,5412], [5414,5415,5416], [5417,5418,5419], [5420,5421,5422], [5423,5424,5425],
[5426,5427,5428], [5433,5444,5445], [5446,5447,5448], [5449,5450,5451], [5452,5453,5454], [5455,5456,5457],

```
[5461,5462,5463],   [5466,5467,5468],   [5471,5472,5473],   [5475,5476,5477],   [5478,5479,5480],   [5481,5482,5483],
[5484,5485,5486],   [5487,5488,5489],   [5494,5505,5506],   [5507,5508,5509],   [5510,5511,5512],   [5513,5514,5515],
[5516,5517,5518],   [5522,5523,5524],   [5527,5528,5529],   [5532,5533,5534],   [5536,5537,5538],   [5539,5540,5541],
[5542,5543,5544],   [5545,5546,5547],   [5548,5549,5550],   [5555,5566,5567],   [5568,5569,5570],   [5571,5572,5573],
[5574,5575,5576],   [5577,5578,5579],   [5583,5584,5585],   [5588,5589,5590],   [5593,5594,5595],   [5597,5598,5599],
[5600,5601,5602],   [5603,5604,5605],   [5606,5607,5608],   [5609,5610,5611],   [5616,5627,5628],   [5629,5630,5631],
[5632,5633,5634],   [5635,5636,5637],   [5638,5639,5640],   [5644,5645,5646],   [5649,5650,5651],   [5654,5655,5656],
[5658,5659,5660],   [5661,5662,5663],   [5664,5665,5666],   [5667,5668,5669],   [5670,5671,5672],   [5677,5688,5689],
[5690,5691,5692],   [5693,5694,5695],   [5696,5697,5698],   [5699,5700,5701],   [5705,5706,5707],   [5710,5711,5712],
[5715,5716,5717],   [5719,5720,5721],   [5722,5723,5724],   [5725,5726,5727],   [5728,5729,5730],   [5731,5732,5733],
[5738,5749,5750],   [5751,5752,5753],   [5754,5755,5756],   [5757,5758,5759],   [5760,5761,5762],   [5766,5767,5768],
[5771,5772,5773],   [5776,5777,5778],   [5780,5781,5782],   [5783,5784,5785],   [5786,5787,5788],   [5789,5790,5791],
[5792,5793,5794],   [5799,5810,5811],   [5812,5813,5814],   [5815,5816,5817],   [5818,5819,5820],   [5821,5822,5823],
[5827,5828,5829],   [5832,5833,5834],   [5837,5838,5839],   [5841,5842,5843],   [5844,5845,5846],   [5847,5848,5849],
[5850,5851,5852],   [5853,5854,5855],   [5860,5871,5872],   [5873,5874,5875],   [5876,5877,5878],   [5879,5880,5881],
[5882,5883,5884],   [5888,5889,5890],   [5893,5894,5895],   [5898,5899,5900],   [5902,5903,5904],   [5905,5906,5907],
[5908,5909,5910],   [5911,5912,5913],   [5914,5915,5916],   [5921,5932,5933],   [5934,5935,5936],   [5937,5938,5939],
[5940,5941,5942],   [5943,5944,5945],   [5949,5950,5951],   [5954,5955,5956],   [5959,5960,5961],   [5963,5964,5965],
[5966,5967,5968],   [5969,5970,5971],   [5972,5973,5974],   [5975,5976,5977],   [5982,5993,5994],   [5995,5996,5997],
[5998,5999,6000],   [6001,6002,6003],   [6004,6005,6006],   [6010,6011,6012],   [6015,6016,6017],   [6020,6021,6022],
[6024,6025,6026],   [6027,6028,6029],   [6030,6031,6032],   [6033,6034,6035],   [6036,6037,6038],   [6043,6054,6055],
[6056,6057,6058],   [6059,6060,6061],   [6062,6063,6064],   [6065,6066,6067],   [6071,6072,6073],   [6076,6077,6078],
[6081,6082,6083],  [6085,6086,6087],  [6088,6089,6090],  [6091,6092,6093],  [6094,6095,6096],  [6097,6098,6099]  ])

# defining Pythom atom indices for each bead as defined in bead_nums_2 with 2 atoms per bead
bead_nums_atoms_2 = np.array([
[9,29],  [30,31],  [35,36],  [40,41],  [14,45],  [70,90],  [91,92],  [96,97],  [101,102],  [75,106],  [131,151],
[152,153],  [157,158],  [162,163],  [136,167],  [192,212],  [213,214],  [218,219],  [223,224],  [197,228],  [253,273],
[274,275],  [279,280],  [284,285],  [258,289],  [314,334],  [335,336],  [340,341],  [345,346],  [319,350],  [375,395],
[396,397],  [401,402],  [406,407],  [380,411],  [436,456],  [457,458],  [462,463],  [467,468],  [441,472],  [497,517],
[518,519],  [523,524],  [528,529],  [502,533],  [558,578],  [579,580],  [584,585],  [589,590],  [563,594],  [619,639],
[640,641],  [645,646],  [650,651],  [624,655],  [680,700],  [701,702],  [706,707],  [711,712],  [685,716],  [741,761],
[762,763],  [767,768],  [772,773],  [746,777],  [802,822],  [823,824],  [828,829],  [833,834],  [807,838],  [863,883],
[884,885],  [889,890],  [894,895],  [868,899],  [924,944],  [945,946],  [950,951],  [955,956],  [929,960],  [985,1005],
[1006,1007],   [1011,1012],   [1016,1017],   [990,1021],   [1046,1066],   [1067,1068],   [1072,1073],   [1077,1078],
[1051,1082],   [1107,1127],   [1128,1129],   [1133,1134],   [1138,1139],   [1112,1143],   [1168,1188],   [1189,1190],
[1194,1195],   [1199,1200],   [1173,1204],   [1229,1249],   [1250,1251],   [1255,1256],   [1260,1261],   [1234,1265],
[1290,1310],   [1311,1312],   [1316,1317],   [1321,1322],   [1295,1326],   [1351,1371],   [1372,1373],   [1377,1378],
[1382,1383],   [1356,1387],   [1412,1432],   [1433,1434],   [1438,1439],   [1443,1444],   [1417,1448],   [1473,1493],
[1494,1495],   [1499,1500],   [1504,1505],   [1478,1509],   [1534,1554],   [1555,1556],   [1560,1561],   [1565,1566],
[1539,1570],   [1595,1615],   [1616,1617],   [1621,1622],   [1626,1627],   [1600,1631],   [1656,1676],   [1677,1678],
[1682,1683],   [1687,1688],   [1661,1692],   [1717,1737],   [1738,1739],   [1743,1744],   [1748,1749],   [1722,1753],
[1778,1798],   [1799,1800],   [1804,1805],   [1809,1810],   [1783,1814],   [1839,1859],   [1860,1861],   [1865,1866],
[1870,1871],   [1844,1875],   [1900,1920],   [1921,1922],   [1926,1927],   [1931,1932],   [1905,1936],   [1961,1981],
[1982,1983],   [1987,1988],   [1992,1993],   [1966,1997],   [2022,2042],   [2043,2044],   [2048,2049],   [2053,2054],
[2027,2058],   [2083,2103],   [2104,2105],   [2109,2110],   [2114,2115],   [2088,2119],   [2144,2164],   [2165,2166],
[2170,2171],   [2175,2176],   [2149,2180],   [2205,2225],   [2226,2227],   [2231,2232],   [2236,2237],   [2210,2241],
[2266,2286],   [2287,2288],   [2292,2293],   [2297,2298],   [2271,2302],   [2327,2347],   [2348,2349],   [2353,2354],
[2358,2359],   [2332,2363],   [2388,2408],   [2409,2410],   [2414,2415],   [2419,2420],   [2393,2424],   [2449,2469],
[2470,2471],   [2475,2476],   [2480,2481],   [2454,2485],   [2510,2530],   [2531,2532],   [2536,2537],   [2541,2542],
[2515,2546],   [2571,2591],   [2592,2593],   [2597,2598],   [2602,2603],   [2576,2607],   [2632,2652],   [2653,2654],
[2658,2659],   [2663,2664],   [2637,2668],   [2693,2713],   [2714,2715],   [2719,2720],   [2724,2725],   [2698,2729],
[2754,2774],   [2775,2776],   [2780,2781],   [2785,2786],   [2759,2790],   [2815,2835],   [2836,2837],   [2841,2842],
[2846,2847],   [2820,2851],   [2876,2896],   [2897,2898],   [2902,2903],   [2907,2908],   [2881,2912],   [2937,2957],
[2958,2959],   [2963,2964],   [2968,2969],   [2942,2973],   [2998,3018],   [3019,3020],   [3024,3025],   [3029,3030],
[3003,3034],   [3059,3079],   [3080,3081],   [3085,3086],   [3090,3091],   [3064,3095],   [3120,3140],   [3141,3142],
[3146,3147],   [3151,3152],   [3125,3156],   [3181,3201],   [3202,3203],   [3207,3208],   [3212,3213],   [3186,3217],
[3242,3262],   [3263,3264],   [3268,3269],   [3273,3274],   [3247,3278],   [3303,3323],   [3324,3325],   [3329,3330],
[3334,3335],   [3308,3339],   [3364,3384],   [3385,3386],   [3390,3391],   [3395,3396],   [3369,3400],   [3425,3445],
[3446,3447],   [3451,3452],   [3456,3457],   [3430,3461],   [3486,3506],   [3507,3508],   [3512,3513],   [3517,3518],
[3491,3522],   [3547,3567],   [3568,3569],   [3573,3574],   [3578,3579],   [3552,3583],   [3608,3628],   [3629,3630],
[3634,3635],   [3639,3640],   [3613,3644],   [3669,3689],   [3690,3691],   [3695,3696],   [3700,3701],   [3674,3705],
[3730,3750],   [3751,3752],   [3756,3757],   [3761,3762],   [3735,3766],   [3791,3811],   [3812,3813],   [3817,3818],
[3822,3823],   [3796,3827],   [3852,3872],   [3873,3874],   [3878,3879],   [3883,3884],   [3857,3888],   [3913,3933],
[3934,3935],   [3939,3940],   [3944,3945],   [3918,3949],   [3974,3994],   [3995,3996],   [4000,4001],   [4005,4006],
[3979,4010],   [4035,4055],   [4056,4057],   [4061,4062],   [4066,4067],   [4040,4071],   [4096,4116],   [4117,4118],
[4122,4123],   [4127,4128],   [4101,4132],   [4157,4177],   [4178,4179],   [4183,4184],   [4188,4189],   [4162,4193],
[4218,4238],   [4239,4240],   [4244,4245],   [4249,4250],   [4223,4254],   [4279,4299],   [4300,4301],   [4305,4306],
[4310,4311],   [4284,4315],   [4340,4360],   [4361,4362],   [4366,4367],   [4371,4372],   [4345,4376],   [4401,4421],
[4422,4423],   [4427,4428],   [4432,4433],   [4406,4437],   [4462,4482],   [4483,4484],   [4488,4489],   [4493,4494],
[4467,4498],   [4523,4543],   [4544,4545],   [4549,4550],   [4554,4555],   [4528,4559],   [4584,4604],   [4605,4606],
[4610,4611],   [4615,4616],   [4589,4620],   [4645,4665],   [4666,4667],   [4671,4672],   [4676,4677],   [4650,4681],
[4706,4726],   [4727,4728],   [4732,4733],   [4737,4738],   [4711,4742],   [4767,4787],   [4788,4789],   [4793,4794],
[4798,4799],   [4772,4803],   [4828,4848],   [4849,4850],   [4854,4855],   [4859,4860],   [4833,4864],   [4889,4909],
[4910,4911],   [4915,4916],   [4920,4921],   [4894,4925],   [4950,4970],   [4971,4972],   [4976,4977],   [4981,4982],
[4955,4986],   [5011,5031],   [5032,5033],   [5037,5038],   [5042,5043],   [5016,5047],   [5072,5092],   [5093,5094],
[5098,5099],   [5103,5104],   [5077,5108],   [5133,5153],   [5154,5155],   [5159,5160],   [5164,5165],   [5138,5169],
[5194,5214],   [5215,5216],   [5220,5221],   [5225,5226],   [5199,5230],   [5255,5275],   [5276,5277],   [5281,5282],
[5286,5287],   [5260,5291],   [5316,5336],   [5337,5338],   [5342,5343],   [5347,5348],   [5321,5352],   [5377,5397],
[5398,5399],   [5403,5404],   [5408,5409],   [5382,5413],   [5438,5458],   [5459,5460],   [5464,5465],   [5469,5470],
[5443,5474],   [5499,5519],   [5520,5521],   [5525,5526],   [5530,5531],   [5504,5535],   [5560,5580],   [5581,5582],
[5586,5587],   [5591,5592],   [5565,5596],   [5621,5641],   [5642,5643],   [5647,5648],   [5652,5653],   [5626,5657],
[5682,5702],   [5703,5704],   [5708,5709],   [5713,5714],   [5687,5718],   [5743,5763],   [5764,5765],   [5769,5770],
[5774,5775],   [5748,5779],   [5804,5824],   [5825,5826],   [5830,5831],   [5835,5836],   [5809,5840],   [5865,5885],
[5886,5887],   [5891,5892],   [5896,5897],   [5870,5901],   [5926,5946],   [5947,5948],   [5952,5953],   [5957,5958],
```

```
[5931,5962],   [5987,6007],   [6008,6009],   [6013,6014],   [6018,6019],   [5992,6023],   [6048,6068],   [6069,6070],
[6074,6075],  [6079,6080],  [6053,6084] ])


# LIST OF BEAD CONNECTIONS TO CALCULATE BEAD "BOND" DISTANCES AND CALCULATIONS

bead_connections_list = np.array([
[111,112],  [112,113],  [113,114],  [114,115],  [115,116],  [111,121],  [121,122],  [122,123],  [123,124],  [124,125],
[125,126],  [126,127],  [127,128],  [121,131],  [131,132],  [132,133],  [133,134],  [134,135],  [135,136],  [136,137],
[211,212],  [212,213],  [213,214],  [214,215],  [215,216],  [211,221],  [221,222],  [222,223],  [223,224],  [224,225],
[225,226],  [226,227],  [227,228],  [221,231],  [231,232],  [232,233],  [233,234],  [234,235],  [235,236],  [236,237],
[311,312],  [312,313],  [313,314],  [314,315],  [315,316],  [311,321],  [321,322],  [322,323],  [323,324],  [324,325],
[325,326],  [326,327],  [327,328],  [321,331],  [331,332],  [332,333],  [333,334],  [334,335],  [335,336],  [336,337],
[411,412],  [412,413],  [413,414],  [414,415],  [415,416],  [411,421],  [421,422],  [422,423],  [423,424],  [424,425],
[425,426],  [426,427],  [427,428],  [421,431],  [431,432],  [432,433],  [433,434],  [434,435],  [435,436],  [436,437],
[511,512],  [512,513],  [513,514],  [514,515],  [515,516],  [511,521],  [521,522],  [522,523],  [523,524],  [524,525],
[525,526],  [526,527],  [527,528],  [521,531],  [531,532],  [532,533],  [533,534],  [534,535],  [535,536],  [536,537],
[611,612],  [612,613],  [613,614],  [614,615],  [615,616],  [611,621],  [621,622],  [622,623],  [623,624],  [624,625],
[625,626],  [626,627],  [627,628],  [621,631],  [631,632],  [632,633],  [633,634],  [634,635],  [635,636],  [636,637],
[711,712],  [712,713],  [713,714],  [714,715],  [715,716],  [711,721],  [721,722],  [722,723],  [723,724],  [724,725],
[725,726],  [726,727],  [727,728],  [721,731],  [731,732],  [732,733],  [733,734],  [734,735],  [735,736],  [736,737],
[811,812],  [812,813],  [813,814],  [814,815],  [815,816],  [811,821],  [821,822],  [822,823],  [823,824],  [824,825],
[825,826],  [826,827],  [827,828],  [821,831],  [831,832],  [832,833],  [833,834],  [834,835],  [835,836],  [836,837],
[911,912],  [912,913],  [913,914],  [914,915],  [915,916],  [911,921],  [921,922],  [922,923],  [923,924],  [924,925],
[925,926],  [926,927],  [927,928],  [921,931],  [931,932],  [932,933],  [933,934],  [934,935],  [935,936],  [936,937],
[1011,1012],   [1012,1013],   [1013,1014],   [1014,1015],   [1015,1016],   [1011,1021],   [1021,1022],   [1022,1023],
[1023,1024],   [1024,1025],   [1025,1026],   [1026,1027],   [1027,1028],   [1021,1031],   [1031,1032],   [1032,1033],
[1033,1034],   [1034,1035],   [1035,1036],   [1036,1037],   [1111,1112],   [1112,1113],   [1113,1114],   [1114,1115],
[1115,1116],   [1111,1121],   [1121,1122],   [1122,1123],   [1123,1124],   [1124,1125],   [1125,1126],   [1126,1127],
[1127,1128],   [1121,1131],   [1131,1132],   [1132,1133],   [1133,1134],   [1134,1135],   [1135,1136],   [1136,1137],
[1211,1212],   [1212,1213],   [1213,1214],   [1214,1215],   [1215,1216],   [1211,1221],   [1221,1222],   [1222,1223],
[1223,1224],   [1224,1225],   [1225,1226],   [1226,1227],   [1227,1228],   [1221,1231],   [1231,1232],   [1232,1233],
[1233,1234],   [1234,1235],   [1235,1236],   [1236,1237],   [1311,1312],   [1312,1313],   [1313,1314],   [1314,1315],
[1315,1316],   [1311,1321],   [1321,1322],   [1322,1323],   [1323,1324],   [1324,1325],   [1325,1326],   [1326,1327],
[1327,1328],   [1321,1331],   [1331,1332],   [1332,1333],   [1333,1334],   [1334,1335],   [1335,1336],   [1336,1337],
[1411,1412],   [1412,1413],   [1413,1414],   [1414,1415],   [1415,1416],   [1411,1421],   [1421,1422],   [1422,1423],
[1423,1424],   [1424,1425],   [1425,1426],   [1426,1427],   [1427,1428],   [1421,1431],   [1431,1432],   [1432,1433],
[1433,1434],   [1434,1435],   [1435,1436],   [1436,1437],   [1511,1512],   [1512,1513],   [1513,1514],   [1514,1515],
[1515,1516],   [1511,1521],   [1521,1522],   [1522,1523],   [1523,1524],   [1524,1525],   [1525,1526],   [1526,1527],
[1527,1528],   [1521,1531],   [1531,1532],   [1532,1533],   [1533,1534],   [1534,1535],   [1535,1536],   [1536,1537],
[1611,1612],   [1612,1613],   [1613,1614],   [1614,1615],   [1615,1616],   [1611,1621],   [1621,1622],   [1622,1623],
[1623,1624],   [1624,1625],   [1625,1626],   [1626,1627],   [1627,1628],   [1621,1631],   [1631,1632],   [1632,1633],
[1633,1634],   [1634,1635],   [1635,1636],   [1636,1637],   [1711,1712],   [1712,1713],   [1713,1714],   [1714,1715],
[1715,1716],   [1711,1721],   [1721,1722],   [1722,1723],   [1723,1724],   [1724,1725],   [1725,1726],   [1726,1727],
[1727,1728],   [1721,1731],   [1731,1732],   [1732,1733],   [1733,1734],   [1734,1735],   [1735,1736],   [1736,1737],
[1811,1812],   [1812,1813],   [1813,1814],   [1814,1815],   [1815,1816],   [1811,1821],   [1821,1822],   [1822,1823],
[1823,1824],   [1824,1825],   [1825,1826],   [1826,1827],   [1827,1828],   [1821,1831],   [1831,1832],   [1832,1833],
[1833,1834],   [1834,1835],   [1835,1836],   [1836,1837],   [1911,1912],   [1912,1913],   [1913,1914],   [1914,1915],
[1915,1916],   [1911,1921],   [1921,1922],   [1922,1923],   [1923,1924],   [1924,1925],   [1925,1926],   [1926,1927],
[1927,1928],   [1921,1931],   [1931,1932],   [1932,1933],   [1933,1934],   [1934,1935],   [1935,1936],   [1936,1937],
[2011,2012],   [2012,2013],   [2013,2014],   [2014,2015],   [2015,2016],   [2011,2021],   [2021,2022],   [2022,2023],
[2023,2024],   [2024,2025],   [2025,2026],   [2026,2027],   [2027,2028],   [2021,2031],   [2031,2032],   [2032,2033],
[2033,2034],   [2034,2035],   [2035,2036],   [2036,2037],   [2111,2112],   [2112,2113],   [2113,2114],   [2114,2115],
[2115,2116],   [2111,2121],   [2121,2122],   [2122,2123],   [2123,2124],   [2124,2125],   [2125,2126],   [2126,2127],
[2127,2128],   [2121,2131],   [2131,2132],   [2132,2133],   [2133,2134],   [2134,2135],   [2135,2136],   [2136,2137],
[2211,2212],   [2212,2213],   [2213,2214],   [2214,2215],   [2215,2216],   [2211,2221],   [2221,2222],   [2222,2223],
[2223,2224],   [2224,2225],   [2225,2226],   [2226,2227],   [2227,2228],   [2221,2231],   [2231,2232],   [2232,2233],
[2233,2234],   [2234,2235],   [2235,2236],   [2236,2237],   [2311,2312],   [2312,2313],   [2313,2314],   [2314,2315],
[2315,2316],   [2311,2321],   [2321,2322],   [2322,2323],   [2323,2324],   [2324,2325],   [2325,2326],   [2326,2327],
[2327,2328],   [2321,2331],   [2331,2332],   [2332,2333],   [2333,2334],   [2334,2335],   [2335,2336],   [2336,2337],
[2411,2412],   [2412,2413],   [2413,2414],   [2414,2415],   [2415,2416],   [2411,2421],   [2421,2422],   [2422,2423],
[2423,2424],   [2424,2425],   [2425,2426],   [2426,2427],   [2427,2428],   [2421,2431],   [2431,2432],   [2432,2433],
[2433,2434],   [2434,2435],   [2435,2436],   [2436,2437],   [2511,2512],   [2512,2513],   [2513,2514],   [2514,2515],
[2515,2516],   [2511,2521],   [2521,2522],   [2522,2523],   [2523,2524],   [2524,2525],   [2525,2526],   [2526,2527],
[2527,2528],   [2521,2531],   [2531,2532],   [2532,2533],   [2533,2534],   [2534,2535],   [2535,2536],   [2536,2537],
[2611,2612],   [2612,2613],   [2613,2614],   [2614,2615],   [2615,2616],   [2611,2621],   [2621,2622],   [2622,2623],
[2623,2624],   [2624,2625],   [2625,2626],   [2626,2627],   [2627,2628],   [2621,2631],   [2631,2632],   [2632,2633],
[2633,2634],   [2634,2635],   [2635,2636],   [2636,2637],   [2711,2712],   [2712,2713],   [2713,2714],   [2714,2715],
[2715,2716],   [2711,2721],   [2721,2722],   [2722,2723],   [2723,2724],   [2724,2725],   [2725,2726],   [2726,2727],
[2727,2728],   [2721,2731],   [2731,2732],   [2732,2733],   [2733,2734],   [2734,2735],   [2735,2736],   [2736,2737],
[2811,2812],   [2812,2813],   [2813,2814],   [2814,2815],   [2815,2816],   [2811,2821],   [2821,2822],   [2822,2823],
[2823,2824],   [2824,2825],   [2825,2826],   [2826,2827],   [2827,2828],   [2821,2831],   [2831,2832],   [2832,2833],
[2833,2834],   [2834,2835],   [2835,2836],   [2836,2837],   [2911,2912],   [2912,2913],   [2913,2914],   [2914,2915],
[2915,2916],   [2911,2921],   [2921,2922],   [2922,2923],   [2923,2924],   [2924,2925],   [2925,2926],   [2926,2927],
[2927,2928],   [2921,2931],   [2931,2932],   [2932,2933],   [2933,2934],   [2934,2935],   [2935,2936],   [2936,2937],
[3011,3012],   [3012,3013],   [3013,3014],   [3014,3015],   [3015,3016],   [3011,3021],   [3021,3022],   [3022,3023],
[3023,3024],   [3024,3025],   [3025,3026],   [3026,3027],   [3027,3028],   [3021,3031],   [3031,3032],   [3032,3033],
[3033,3034],   [3034,3035],   [3035,3036],   [3036,3037],   [3111,3112],   [3112,3113],   [3113,3114],   [3114,3115],
[3115,3116],   [3111,3121],   [3121,3122],   [3122,3123],   [3123,3124],   [3124,3125],   [3125,3126],   [3126,3127],
[3127,3128],   [3121,3131],   [3131,3132],   [3132,3133],   [3133,3134],   [3134,3135],   [3135,3136],   [3136,3137],
[3211,3212],   [3212,3213],   [3213,3214],   [3214,3215],   [3215,3216],   [3211,3221],   [3221,3222],   [3222,3223],
[3223,3224],   [3224,3225],   [3225,3226],   [3226,3227],   [3227,3228],   [3221,3231],   [3231,3232],   [3232,3233],
[3233,3234],   [3234,3235],   [3235,3236],   [3236,3237],   [3311,3312],   [3312,3313],   [3313,3314],   [3314,3315],
[3315,3316],   [3311,3321],   [3321,3322],   [3322,3323],   [3323,3324],   [3324,3325],   [3325,3326],   [3326,3327],
[3327,3328],   [3321,3331],   [3331,3332],   [3332,3333],   [3333,3334],   [3334,3335],   [3335,3336],   [3336,3337],
```

[3411,3412], [3412,3413], [3413,3414], [3414,3415], [3415,3416], [3411,3421], [3421,3422], [3422,3423],
[3423,3424], [3424,3425], [3425,3426], [3426,3427], [3427,3428], [3421,3431], [3431,3432], [3432,3433],
[3433,3434], [3434,3435], [3435,3436], [3436,3437], [3511,3512], [3512,3513], [3513,3514], [3514,3515],
[3515,3516], [3511,3521], [3521,3522], [3522,3523], [3523,3524], [3524,3525], [3525,3526], [3526,3527],
[3527,3528], [3521,3531], [3531,3532], [3532,3533], [3533,3534], [3534,3535], [3535,3536], [3536,3537],
[3611,3612], [3612,3613], [3613,3614], [3614,3615], [3615,3616], [3611,3621], [3621,3622], [3622,3623],
[3623,3624], [3624,3625], [3625,3626], [3626,3627], [3627,3628], [3621,3631], [3631,3632], [3632,3633],
[3633,3634], [3634,3635], [3635,3636], [3636,3637], [3711,3712], [3712,3713], [3713,3714], [3714,3715],
[3715,3716], [3711,3721], [3721,3722], [3722,3723], [3723,3724], [3724,3725], [3725,3726], [3726,3727],
[3727,3728], [3721,3731], [3731,3732], [3732,3733], [3733,3734], [3734,3735], [3735,3736], [3736,3737],
[3811,3812], [3812,3813], [3813,3814], [3814,3815], [3815,3816], [3811,3821], [3821,3822], [3822,3823],
[3823,3824], [3824,3825], [3825,3826], [3826,3827], [3827,3828], [3821,3831], [3831,3832], [3832,3833],
[3833,3834], [3834,3835], [3835,3836], [3836,3837], [3911,3912], [3912,3913], [3913,3914], [3914,3915],
[3915,3916], [3911,3921], [3921,3922], [3922,3923], [3923,3924], [3924,3925], [3925,3926], [3926,3927],
[3927,3928], [3921,3931], [3931,3932], [3932,3933], [3933,3934], [3934,3935], [3935,3936], [3936,3937],
[4011,4012], [4012,4013], [4013,4014], [4014,4015], [4015,4016], [4011,4021], [4021,4022], [4022,4023],
[4023,4024], [4024,4025], [4025,4026], [4026,4027], [4027,4028], [4021,4031], [4031,4032], [4032,4033],
[4033,4034], [4034,4035], [4035,4036], [4036,4037], [4111,4112], [4112,4113], [4113,4114], [4114,4115],
[4115,4116], [4111,4121], [4121,4122], [4122,4123], [4123,4124], [4124,4125], [4125,4126], [4126,4127],
[4127,4128], [4121,4131], [4131,4132], [4132,4133], [4133,4134], [4134,4135], [4135,4136], [4136,4137],
[4211,4212], [4212,4213], [4213,4214], [4214,4215], [4215,4216], [4211,4221], [4221,4222], [4222,4223],
[4223,4224], [4224,4225], [4225,4226], [4226,4227], [4227,4228], [4221,4231], [4231,4232], [4232,4233],
[4233,4234], [4234,4235], [4235,4236], [4236,4237], [4311,4312], [4312,4313], [4313,4314], [4314,4315],
[4315,4316], [4311,4321], [4321,4322], [4322,4323], [4323,4324], [4324,4325], [4325,4326], [4326,4327],
[4327,4328], [4321,4331], [4331,4332], [4332,4333], [4333,4334], [4334,4335], [4335,4336], [4336,4337],
[4411,4412], [4412,4413], [4413,4414], [4414,4415], [4415,4416], [4411,4421], [4421,4422], [4422,4423],
[4423,4424], [4424,4425], [4425,4426], [4426,4427], [4427,4428], [4421,4431], [4431,4432], [4432,4433],
[4433,4434], [4434,4435], [4435,4436], [4436,4437], [4511,4512], [4512,4513], [4513,4514], [4514,4515],
[4515,4516], [4511,4521], [4521,4522], [4522,4523], [4523,4524], [4524,4525], [4525,4526], [4526,4527],
[4527,4528], [4521,4531], [4531,4532], [4532,4533], [4533,4534], [4534,4535], [4535,4536], [4536,4537],
[4611,4612], [4612,4613], [4613,4614], [4614,4615], [4615,4616], [4611,4621], [4621,4622], [4622,4623],
[4623,4624], [4624,4625], [4625,4626], [4626,4627], [4627,4628], [4621,4631], [4631,4632], [4632,4633],
[4633,4634], [4634,4635], [4635,4636], [4636,4637], [4711,4712], [4712,4713], [4713,4714], [4714,4715],
[4715,4716], [4711,4721], [4721,4722], [4722,4723], [4723,4724], [4724,4725], [4725,4726], [4726,4727],
[4727,4728], [4721,4731], [4731,4732], [4732,4733], [4733,4734], [4734,4735], [4735,4736], [4736,4737],
[4811,4812], [4812,4813], [4813,4814], [4814,4815], [4815,4816], [4811,4821], [4821,4822], [4822,4823],
[4823,4824], [4824,4825], [4825,4826], [4826,4827], [4827,4828], [4821,4831], [4831,4832], [4832,4833],
[4833,4834], [4834,4835], [4835,4836], [4836,4837], [4911,4912], [4912,4913], [4913,4914], [4914,4915],
[4915,4916], [4911,4921], [4921,4922], [4922,4923], [4923,4924], [4924,4925], [4925,4926], [4926,4927],
[4927,4928], [4921,4931], [4931,4932], [4932,4933], [4933,4934], [4934,4935], [4935,4936], [4936,4937],
[5011,5012], [5012,5013], [5013,5014], [5014,5015], [5015,5016], [5011,5021], [5021,5022], [5022,5023],
[5023,5024], [5024,5025], [5025,5026], [5026,5027], [5027,5028], [5021,5031], [5031,5032], [5032,5033],
[5033,5034], [5034,5035], [5035,5036], [5036,5037], [5111,5112], [5112,5113], [5113,5114], [5114,5115],
[5115,5116], [5111,5121], [5121,5122], [5122,5123], [5123,5124], [5124,5125], [5125,5126], [5126,5127],
[5127,5128], [5121,5131], [5131,5132], [5132,5133], [5133,5134], [5134,5135], [5135,5136], [5136,5137],
[5211,5212], [5212,5213], [5213,5214], [5214,5215], [5215,5216], [5211,5221], [5221,5222], [5222,5223],
[5223,5224], [5224,5225], [5225,5226], [5226,5227], [5227,5228], [5221,5231], [5231,5232], [5232,5233],
[5233,5234], [5234,5235], [5235,5236], [5236,5237], [5311,5312], [5312,5313], [5313,5314], [5314,5315],
[5315,5316], [5311,5321], [5321,5322], [5322,5323], [5323,5324], [5324,5325], [5325,5326], [5326,5327],
[5327,5328], [5321,5331], [5331,5332], [5332,5333], [5333,5334], [5334,5335], [5335,5336], [5336,5337],
[5411,5412], [5412,5413], [5413,5414], [5414,5415], [5415,5416], [5411,5421], [5421,5422], [5422,5423],
[5423,5424], [5424,5425], [5425,5426], [5426,5427], [5427,5428], [5421,5431], [5431,5432], [5432,5433],
[5433,5434], [5434,5435], [5435,5436], [5436,5437], [5511,5512], [5512,5513], [5513,5514], [5514,5515],
[5515,5516], [5511,5521], [5521,5522], [5522,5523], [5523,5524], [5524,5525], [5525,5526], [5526,5527],
[5527,5528], [5521,5531], [5531,5532], [5532,5533], [5533,5534], [5534,5535], [5535,5536], [5536,5537],
[5611,5612], [5612,5613], [5613,5614], [5614,5615], [5615,5616], [5611,5621], [5621,5622], [5622,5623],
[5623,5624], [5624,5625], [5625,5626], [5626,5627], [5627,5628], [5621,5631], [5631,5632], [5632,5633],
[5633,5634], [5634,5635], [5635,5636], [5636,5637], [5711,5712], [5712,5713], [5713,5714], [5714,5715],
[5715,5716], [5711,5721], [5721,5722], [5722,5723], [5723,5724], [5724,5725], [5725,5726], [5726,5727],
[5727,5728], [5721,5731], [5731,5732], [5732,5733], [5733,5734], [5734,5735], [5735,5736], [5736,5737],
[5811,5812], [5812,5813], [5813,5814], [5814,5815], [5815,5816], [5811,5821], [5821,5822], [5822,5823],
[5823,5824], [5824,5825], [5825,5826], [5826,5827], [5827,5828], [5821,5831], [5831,5832], [5832,5833],
[5833,5834], [5834,5835], [5835,5836], [5836,5837], [5911,5912], [5912,5913], [5913,5914], [5914,5915],
[5915,5916], [5911,5921], [5921,5922], [5922,5923], [5923,5924], [5924,5925], [5925,5926], [5926,5927],
[5927,5928], [5921,5931], [5931,5932], [5932,5933], [5933,5934], [5934,5935], [5935,5936], [5936,5937],
[6011,6012], [6012,6013], [6013,6014], [6014,6015], [6015,6016], [6011,6021], [6021,6022], [6022,6023],
[6023,6024], [6024,6025], [6025,6026], [6026,6027], [6027,6028], [6021,6031], [6031,6032], [6032,6033],
[6033,6034], [6034,6035], [6035,6036], [6036,6037], [6111,6112], [6112,6113], [6113,6114], [6114,6115],
[6115,6116], [6111,6121], [6121,6122], [6122,6123], [6123,6124], [6124,6125], [6125,6126], [6126,6127],
[6127,6128], [6121,6131], [6131,6132], [6132,6133], [6133,6134], [6134,6135], [6135,6136], [6136,6137],
[6211,6212], [6212,6213], [6213,6214], [6214,6215], [6215,6216], [6211,6221], [6221,6222], [6222,6223],
[6223,6224], [6224,6225], [6225,6226], [6226,6227], [6227,6228], [6221,6231], [6231,6232], [6232,6233],
[6233,6234], [6234,6235], [6235,6236], [6236,6237], [6311,6312], [6312,6313], [6313,6314], [6314,6315],
[6315,6316], [6311,6321], [6321,6322], [6322,6323], [6323,6324], [6324,6325], [6325,6326], [6326,6327],
[6327,6328], [6321,6331], [6331,6332], [6332,6333], [6333,6334], [6334,6335], [6335,6336], [6336,6337],
[6411,6412], [6412,6413], [6413,6414], [6414,6415], [6415,6416], [6411,6421], [6421,6422], [6422,6423],
[6423,6424], [6424,6425], [6425,6426], [6426,6427], [6427,6428], [6421,6431], [6431,6432], [6432,6433],
[6433,6434], [6434,6435], [6435,6436], [6436,6437], [6511,6512], [6512,6513], [6513,6514], [6514,6515],
[6515,6516], [6511,6521], [6521,6522], [6522,6523], [6523,6524], [6524,6525], [6525,6526], [6526,6527],
[6527,6528], [6521,6531], [6531,6532], [6532,6533], [6533,6534], [6534,6535], [6535,6536], [6536,6537],
[6611,6612], [6612,6613], [6613,6614], [6614,6615], [6615,6616], [6611,6621], [6621,6622], [6622,6623],
[6623,6624], [6624,6625], [6625,6626], [6626,6627], [6627,6628], [6621,6631], [6631,6632], [6632,6633],
[6633,6634], [6634,6635], [6635,6636], [6636,6637], [6711,6712], [6712,6713], [6713,6714], [6714,6715],
[6715,6716], [6711,6721], [6721,6722], [6722,6723], [6723,6724], [6724,6725], [6725,6726], [6726,6727],
[6727,6728], [6721,6731], [6731,6732], [6732,6733], [6733,6734], [6734,6735], [6735,6736], [6736,6737],

```
[6811,6812],   [6812,6813],   [6813,6814],   [6814,6815],   [6815,6816],   [6811,6821],   [6821,6822],   [6822,6823],
[6823,6824],   [6824,6825],   [6825,6826],   [6826,6827],   [6827,6828],   [6821,6831],   [6831,6832],   [6832,6833],
[6833,6834],   [6834,6835],   [6835,6836],   [6836,6837],   [6911,6912],   [6912,6913],   [6913,6914],   [6914,6915],
[6915,6916],   [6911,6921],   [6921,6922],   [6922,6923],   [6923,6924],   [6924,6925],   [6925,6926],   [6926,6927],
[6927,6928],   [6921,6931],   [6931,6932],   [6932,6933],   [6933,6934],   [6934,6935],   [6935,6936],   [6936,6937],
[7011,7012],   [7012,7013],   [7013,7014],   [7014,7015],   [7015,7016],   [7011,7021],   [7021,7022],   [7022,7023],
[7023,7024],   [7024,7025],   [7025,7026],   [7026,7027],   [7027,7028],   [7021,7031],   [7031,7032],   [7032,7033],
[7033,7034],   [7034,7035],   [7035,7036],   [7036,7037],   [7111,7112],   [7112,7113],   [7113,7114],   [7114,7115],
[7115,7116],   [7111,7121],   [7121,7122],   [7122,7123],   [7123,7124],   [7124,7125],   [7125,7126],   [7126,7127],
[7127,7128],   [7121,7131],   [7131,7132],   [7132,7133],   [7133,7134],   [7134,7135],   [7135,7136],   [7136,7137],
[7211,7212],   [7212,7213],   [7213,7214],   [7214,7215],   [7215,7216],   [7211,7221],   [7221,7222],   [7222,7223],
[7223,7224],   [7224,7225],   [7225,7226],   [7226,7227],   [7227,7228],   [7221,7231],   [7231,7232],   [7232,7233],
[7233,7234],   [7234,7235],   [7235,7236],   [7236,7237],   [7311,7312],   [7312,7313],   [7313,7314],   [7314,7315],
[7315,7316],   [7311,7321],   [7321,7322],   [7322,7323],   [7323,7324],   [7324,7325],   [7325,7326],   [7326,7327],
[7327,7328],   [7321,7331],   [7331,7332],   [7332,7333],   [7333,7334],   [7334,7335],   [7335,7336],   [7336,7337],
[7411,7412],   [7412,7413],   [7413,7414],   [7414,7415],   [7415,7416],   [7411,7421],   [7421,7422],   [7422,7423],
[7423,7424],   [7424,7425],   [7425,7426],   [7426,7427],   [7427,7428],   [7421,7431],   [7431,7432],   [7432,7433],
[7433,7434],   [7434,7435],   [7435,7436],   [7436,7437],   [7511,7512],   [7512,7513],   [7513,7514],   [7514,7515],
[7515,7516],   [7511,7521],   [7521,7522],   [7522,7523],   [7523,7524],   [7524,7525],   [7525,7526],   [7526,7527],
[7527,7528],   [7521,7531],   [7531,7532],   [7532,7533],   [7533,7534],   [7534,7535],   [7535,7536],   [7536,7537],
[7611,7612],   [7612,7613],   [7613,7614],   [7614,7615],   [7615,7616],   [7611,7621],   [7621,7622],   [7622,7623],
[7623,7624],   [7624,7625],   [7625,7626],   [7626,7627],   [7627,7628],   [7621,7631],   [7631,7632],   [7632,7633],
[7633,7634],   [7634,7635],   [7635,7636],   [7636,7637],   [7711,7712],   [7712,7713],   [7713,7714],   [7714,7715],
[7715,7716],   [7711,7721],   [7721,7722],   [7722,7723],   [7723,7724],   [7724,7725],   [7725,7726],   [7726,7727],
[7727,7728],   [7721,7731],   [7731,7732],   [7732,7733],   [7733,7734],   [7734,7735],   [7735,7736],   [7736,7737],
[7811,7812],   [7812,7813],   [7813,7814],   [7814,7815],   [7815,7816],   [7811,7821],   [7821,7822],   [7822,7823],
[7823,7824],   [7824,7825],   [7825,7826],   [7826,7827],   [7827,7828],   [7821,7831],   [7831,7832],   [7832,7833],
[7833,7834],   [7834,7835],   [7835,7836],   [7836,7837],   [7911,7912],   [7912,7913],   [7913,7914],   [7914,7915],
[7915,7916],   [7911,7921],   [7921,7922],   [7922,7923],   [7923,7924],   [7924,7925],   [7925,7926],   [7926,7927],
[7927,7928],   [7921,7931],   [7931,7932],   [7932,7933],   [7933,7934],   [7934,7935],   [7935,7936],   [7936,7937],
[8011,8012],   [8012,8013],   [8013,8014],   [8014,8015],   [8015,8016],   [8011,8021],   [8021,8022],   [8022,8023],
[8023,8024],   [8024,8025],   [8025,8026],   [8026,8027],   [8027,8028],   [8021,8031],   [8031,8032],   [8032,8033],
[8033,8034],   [8034,8035],   [8035,8036],   [8036,8037],   [8111,8112],   [8112,8113],   [8113,8114],   [8114,8115],
[8115,8116],   [8111,8121],   [8121,8122],   [8122,8123],   [8123,8124],   [8124,8125],   [8125,8126],   [8126,8127],
[8127,8128],   [8121,8131],   [8131,8132],   [8132,8133],   [8133,8134],   [8134,8135],   [8135,8136],   [8136,8137],
[8211,8212],   [8212,8213],   [8213,8214],   [8214,8215],   [8215,8216],   [8211,8221],   [8221,8222],   [8222,8223],
[8223,8224],   [8224,8225],   [8225,8226],   [8226,8227],   [8227,8228],   [8221,8231],   [8231,8232],   [8232,8233],
[8233,8234],   [8234,8235],   [8235,8236],   [8236,8237],   [8311,8312],   [8312,8313],   [8313,8314],   [8314,8315],
[8315,8316],   [8311,8321],   [8321,8322],   [8322,8323],   [8323,8324],   [8324,8325],   [8325,8326],   [8326,8327],
[8327,8328],   [8321,8331],   [8331,8332],   [8332,8333],   [8333,8334],   [8334,8335],   [8335,8336],   [8336,8337],
[8411,8412],   [8412,8413],   [8413,8414],   [8414,8415],   [8415,8416],   [8411,8421],   [8421,8422],   [8422,8423],
[8423,8424],   [8424,8425],   [8425,8426],   [8426,8427],   [8427,8428],   [8421,8431],   [8431,8432],   [8432,8433],
[8433,8434],   [8434,8435],   [8435,8436],   [8436,8437],   [8511,8512],   [8512,8513],   [8513,8514],   [8514,8515],
[8515,8516],   [8511,8521],   [8521,8522],   [8522,8523],   [8523,8524],   [8524,8525],   [8525,8526],   [8526,8527],
[8527,8528],   [8521,8531],   [8531,8532],   [8532,8533],   [8533,8534],   [8534,8535],   [8535,8536],   [8536,8537],
[8611,8612],   [8612,8613],   [8613,8614],   [8614,8615],   [8615,8616],   [8611,8621],   [8621,8622],   [8622,8623],
[8623,8624],   [8624,8625],   [8625,8626],   [8626,8627],   [8627,8628],   [8621,8631],   [8631,8632],   [8632,8633],
[8633,8634],   [8634,8635],   [8635,8636],   [8636,8637],   [8711,8712],   [8712,8713],   [8713,8714],   [8714,8715],
[8715,8716],   [8711,8721],   [8721,8722],   [8722,8723],   [8723,8724],   [8724,8725],   [8725,8726],   [8726,8727],
[8727,8728],   [8721,8731],   [8731,8732],   [8732,8733],   [8733,8734],   [8734,8735],   [8735,8736],   [8736,8737],
[8811,8812],   [8812,8813],   [8813,8814],   [8814,8815],   [8815,8816],   [8811,8821],   [8821,8822],   [8822,8823],
[8823,8824],   [8824,8825],   [8825,8826],   [8826,8827],   [8827,8828],   [8821,8831],   [8831,8832],   [8832,8833],
[8833,8834],   [8834,8835],   [8835,8836],   [8836,8837],   [8911,8912],   [8912,8913],   [8913,8914],   [8914,8915],
[8915,8916],   [8911,8921],   [8921,8922],   [8922,8923],   [8923,8924],   [8924,8925],   [8925,8926],   [8926,8927],
[8927,8928],   [8921,8931],   [8931,8932],   [8932,8933],   [8933,8934],   [8934,8935],   [8935,8936],   [8936,8937],
[9011,9012],   [9012,9013],   [9013,9014],   [9014,9015],   [9015,9016],   [9011,9021],   [9021,9022],   [9022,9023],
[9023,9024],   [9024,9025],   [9025,9026],   [9026,9027],   [9027,9028],   [9021,9031],   [9031,9032],   [9032,9033],
[9033,9034],   [9034,9035],   [9035,9036],   [9036,9037],   [9111,9112],   [9112,9113],   [9113,9114],   [9114,9115],
[9115,9116],   [9111,9121],   [9121,9122],   [9122,9123],   [9123,9124],   [9124,9125],   [9125,9126],   [9126,9127],
[9127,9128],   [9121,9131],   [9131,9132],   [9132,9133],   [9133,9134],   [9134,9135],   [9135,9136],   [9136,9137],
[9211,9212],   [9212,9213],   [9213,9214],   [9214,9215],   [9215,9216],   [9211,9221],   [9221,9222],   [9222,9223],
[9223,9224],   [9224,9225],   [9225,9226],   [9226,9227],   [9227,9228],   [9221,9231],   [9231,9232],   [9232,9233],
[9233,9234],   [9234,9235],   [9235,9236],   [9236,9237],   [9311,9312],   [9312,9313],   [9313,9314],   [9314,9315],
[9315,9316],   [9311,9321],   [9321,9322],   [9322,9323],   [9323,9324],   [9324,9325],   [9325,9326],   [9326,9327],
[9327,9328],   [9321,9331],   [9331,9332],   [9332,9333],   [9333,9334],   [9334,9335],   [9335,9336],   [9336,9337],
[9411,9412],   [9412,9413],   [9413,9414],   [9414,9415],   [9415,9416],   [9411,9421],   [9421,9422],   [9422,9423],
[9423,9424],   [9424,9425],   [9425,9426],   [9426,9427],   [9427,9428],   [9421,9431],   [9431,9432],   [9432,9433],
[9433,9434],   [9434,9435],   [9435,9436],   [9436,9437],   [9511,9512],   [9512,9513],   [9513,9514],   [9514,9515],
[9515,9516],   [9511,9521],   [9521,9522],   [9522,9523],   [9523,9524],   [9524,9525],   [9525,9526],   [9526,9527],
[9527,9528],   [9521,9531],   [9531,9532],   [9532,9533],   [9533,9534],   [9534,9535],   [9535,9536],   [9536,9537],
[9611,9612],   [9612,9613],   [9613,9614],   [9614,9615],   [9615,9616],   [9611,9621],   [9621,9622],   [9622,9623],
[9623,9624],   [9624,9625],   [9625,9626],   [9626,9627],   [9627,9628],   [9621,9631],   [9631,9632],   [9632,9633],
[9633,9634],   [9634,9635],   [9635,9636],   [9636,9637],   [9711,9712],   [9712,9713],   [9713,9714],   [9714,9715],
[9715,9716],   [9711,9721],   [9721,9722],   [9722,9723],   [9723,9724],   [9724,9725],   [9725,9726],   [9726,9727],
[9727,9728],   [9721,9731],   [9731,9732],   [9732,9733],   [9733,9734],   [9734,9735],   [9735,9736],   [9736,9737],
[9811,9812],   [9812,9813],   [9813,9814],   [9814,9815],   [9815,9816],   [9811,9821],   [9821,9822],   [9822,9823],
[9823,9824],   [9824,9825],   [9825,9826],   [9826,9827],   [9827,9828],   [9821,9831],   [9831,9832],   [9832,9833],
[9833,9834],   [9834,9835],   [9835,9836],   [9836,9837],   [9911,9912],   [9912,9913],   [9913,9914],   [9914,9915],
[9915,9916],   [9911,9921],   [9921,9922],   [9922,9923],   [9923,9924],   [9924,9925],   [9925,9926],   [9926,9927],
[9927,9928],   [9921,9931],   [9931,9932],   [9932,9933],   [9933,9934],   [9934,9935],   [9935,9936],   [9936,9937],
[10011,10012],   [10012,10013],   [10013,10014],   [10014,10015],   [10015,10016],   [10011,10021],   [10021,10022],
[10022,10023],   [10023,10024],   [10024,10025],   [10025,10026],   [10026,10027],   [10027,10028],   [10021,10031],
[10031,10032],  [10032,10033],  [10033,10034],  [10034,10035],  [10035,10036],  [10036,10037] ])

### CALCULATE BEAD DISTANCES AND STATISTICS ###
```

```
# Array giving all the bind distances that need to be combined for statistical purposes
distance_combination_indices = np.array([

[0,20,40,60,80,100,120,140,160,180,200,220,240,260,280,300,320,340,360,380,400,420,440,460,480,500,520,540,560,580,60
0,620,640,660,680,700,720,740,760,780,800,820,840,860,880,900,920,940,960,980,1000,1020,1040,1060,1080,1100,1120,1140
,1160,1180,1200,1220,1240,1260,1280,1300,1320,1340,1360,1380,1400,1420,1440,1460,1480,1500,1520,1540,1560,1580,1600,1
620,1640,1660,1680,1700,1720,1740,1760,1780,1800,1820,1840,1860,1880,1900,1920,1940,1960,1980],

[1,21,41,61,81,101,121,141,161,181,201,221,241,261,281,301,321,341,361,381,401,421,441,461,481,501,521,541,561,581,60
1,621,641,661,681,701,721,741,761,781,801,821,841,861,881,901,921,941,961,981,1001,1021,1041,1061,1081,1101,1121,1141
,1161,1181,1201,1221,1241,1261,1281,1301,1321,1341,1361,1381,1401,1421,1441,1461,1481,1501,1521,1541,1561,1581,1601,1
621,1641,1661,1681,1701,1721,1741,1761,1781,1801,1821,1841,1861,1881,1901,1921,1941,1961,1981],

[2,22,42,62,82,102,122,142,162,182,202,222,242,262,282,302,322,342,362,382,402,422,442,462,482,502,522,542,562,582,60
2,622,642,662,682,702,722,742,762,782,802,822,842,862,882,902,922,942,962,982,1002,1022,1042,1062,1082,1102,1122,1142
,1162,1182,1202,1222,1242,1262,1282,1302,1322,1342,1362,1382,1402,1422,1442,1462,1482,1502,1522,1542,1562,1582,1602,1
622,1642,1662,1682,1702,1722,1742,1762,1782,1802,1822,1842,1862,1882,1902,1922,1942,1962,1982],

[3,23,43,63,83,103,123,143,163,183,203,223,243,263,283,303,323,343,363,383,403,423,443,463,483,503,523,543,563,583,60
3,623,643,663,683,703,723,743,763,783,803,823,843,863,883,903,923,943,963,983,1003,1023,1043,1063,1083,1103,1123,1143
,1163,1183,1203,1223,1243,1263,1283,1303,1323,1343,1363,1383,1403,1423,1443,1463,1483,1503,1523,1543,1563,1583,1603,1
623,1643,1663,1683,1703,1723,1743,1763,1783,1803,1823,1843,1863,1883,1903,1923,1943,1963,1983],

[4,24,44,64,84,104,124,144,164,184,204,224,244,264,284,304,324,344,364,384,404,424,444,464,484,504,524,544,564,584,60
4,624,644,664,684,704,724,744,764,784,804,824,844,864,884,904,924,944,964,984,1004,1024,1044,1064,1084,1104,1124,1144
,1164,1184,1204,1224,1244,1264,1284,1304,1324,1344,1364,1384,1404,1424,1444,1464,1484,1504,1524,1544,1564,1584,1604,1
624,1644,1664,1684,1704,1724,1744,1764,1784,1804,1824,1844,1864,1884,1904,1924,1944,1964,1984],

[5,25,45,65,85,105,125,145,165,185,205,225,245,265,285,305,325,345,365,385,405,425,445,465,485,505,525,545,565,585,60
5,625,645,665,685,705,725,745,765,785,805,825,845,865,885,905,925,945,965,985,1005,1025,1045,1065,1085,1105,1125,1145
,1165,1185,1205,1225,1245,1265,1285,1305,1325,1345,1365,1385,1405,1425,1445,1465,1485,1505,1525,1545,1565,1585,1605,1
625,1645,1665,1685,1705,1725,1745,1765,1785,1805,1825,1845,1865,1885,1905,1925,1945,1965,1985],

[6,26,46,66,86,106,126,146,166,186,206,226,246,266,286,306,326,346,366,386,406,426,446,466,486,506,526,546,566,586,60
6,626,646,666,686,706,726,746,766,786,806,826,846,866,886,906,926,946,966,986,1006,1026,1046,1066,1086,1106,1126,1146
,1166,1186,1206,1226,1246,1266,1286,1306,1326,1346,1366,1386,1406,1426,1446,1466,1486,1506,1526,1546,1566,1586,1606,1
626,1646,1666,1686,1706,1726,1746,1766,1786,1806,1826,1846,1866,1886,1906,1926,1946,1966,1986],

[7,27,47,67,87,107,127,147,167,187,207,227,247,267,287,307,327,347,367,387,407,427,447,467,487,507,527,547,567,587,60
7,627,647,667,687,707,727,747,767,787,807,827,847,867,887,907,927,947,967,987,1007,1027,1047,1067,1087,1107,1127,1147
,1167,1187,1207,1227,1247,1267,1287,1307,1327,1347,1367,1387,1407,1427,1447,1467,1487,1507,1527,1547,1567,1587,1607,1
627,1647,1667,1687,1707,1727,1747,1767,1787,1807,1827,1847,1867,1887,1907,1927,1947,1967,1987],

[8,28,48,68,88,108,128,148,168,188,208,228,248,268,288,308,328,348,368,388,408,428,448,468,488,508,528,548,568,588,60
8,628,648,668,688,708,728,748,768,788,808,828,848,868,888,908,928,948,968,988,1008,1028,1048,1068,1088,1108,1128,1148
,1168,1188,1208,1228,1248,1268,1288,1308,1328,1348,1368,1388,1408,1428,1448,1468,1488,1508,1528,1548,1568,1588,1608,1
628,1648,1668,1688,1708,1728,1748,1768,1788,1808,1828,1848,1868,1888,1908,1928,1948,1968,1988],

[9,29,49,69,89,109,129,149,169,189,209,229,249,269,289,309,329,349,369,389,409,429,449,469,489,509,529,549,569,589,60
9,629,649,669,689,709,729,749,769,789,809,829,849,869,889,909,929,949,969,989,1009,1029,1049,1069,1089,1109,1129,1149
,1169,1189,1209,1229,1249,1269,1289,1309,1329,1349,1369,1389,1409,1429,1449,1469,1489,1509,1529,1549,1569,1589,1609,1
629,1649,1669,1689,1709,1729,1749,1769,1789,1809,1829,1849,1869,1889,1909,1929,1949,1969,1989],

[10,30,50,70,90,110,130,150,170,190,210,230,250,270,290,310,330,350,370,390,410,430,450,470,490,510,530,550,570,590,6
10,630,650,670,690,710,730,750,770,790,810,830,850,870,890,910,930,950,970,990,1010,1030,1050,1070,1090,1110,1130,115
0,1170,1190,1210,1230,1250,1270,1290,1310,1330,1350,1370,1390,1410,1430,1450,1470,1490,1510,1530,1550,1570,1590,1610,
1630,1650,1670,1690,1710,1730,1750,1770,1790,1810,1830,1850,1870,1890,1910,1930,1950,1970,1990],

[11,31,51,71,91,111,131,151,171,191,211,231,251,271,291,311,331,351,371,391,411,431,451,471,491,511,531,551,571,591,6
11,631,651,671,691,711,731,751,771,791,811,831,851,871,891,911,931,951,971,991,1011,1031,1051,1071,1091,1111,1131,115
1,1171,1191,1211,1231,1251,1271,1291,1311,1331,1351,1371,1391,1411,1431,1451,1471,1491,1511,1531,1551,1571,1591,1611,
1631,1651,1671,1691,1711,1731,1751,1771,1791,1811,1831,1851,1871,1891,1911,1931,1951,1971,1991],

[12,32,52,72,92,112,132,152,172,192,212,232,252,272,292,312,332,352,372,392,412,432,452,472,492,512,532,552,572,592,6
12,632,652,672,692,712,732,752,772,792,812,832,852,872,892,912,932,952,972,992,1012,1032,1052,1072,1092,1112,1132,115
2,1172,1192,1212,1232,1252,1272,1292,1312,1332,1352,1372,1392,1412,1432,1452,1472,1492,1512,1532,1552,1572,1592,1612,
1632,1652,1672,1692,1712,1732,1752,1772,1792,1812,1832,1852,1872,1892,1912,1932,1952,1972,1992],

[13,33,53,73,93,113,133,153,173,193,213,233,253,273,293,313,333,353,373,393,413,433,453,473,493,513,533,553,573,593,6
13,633,653,673,693,713,733,753,773,793,813,833,853,873,893,913,933,953,973,993,1013,1033,1053,1073,1093,1113,1133,115
3,1173,1193,1213,1233,1253,1273,1293,1313,1333,1353,1373,1393,1413,1433,1453,1473,1493,1513,1533,1553,1573,1593,1613,
1633,1653,1673,1693,1713,1733,1753,1773,1793,1813,1833,1853,1873,1893,1913,1933,1953,1973,1993],

[14,34,54,74,94,114,134,154,174,194,214,234,254,274,294,314,334,354,374,394,414,434,454,474,494,514,534,554,574,594,6
14,634,654,674,694,714,734,754,774,794,814,834,854,874,894,914,934,954,974,994,1014,1034,1054,1074,1094,1114,1134,115
4,1174,1194,1214,1234,1254,1274,1294,1314,1334,1354,1374,1394,1414,1434,1454,1474,1494,1514,1534,1554,1574,1594,1614,
1634,1654,1674,1694,1714,1734,1754,1774,1794,1814,1834,1854,1874,1894,1914,1934,1954,1974,1994],

[15,35,55,75,95,115,135,155,175,195,215,235,255,275,295,315,335,355,375,395,415,435,455,475,495,515,535,555,575,595,6
15,635,655,675,695,715,735,755,775,795,815,835,855,875,895,915,935,955,975,995,1015,1035,1055,1075,1095,1115,1135,115
5,1175,1195,1215,1235,1255,1275,1295,1315,1335,1355,1375,1395,1415,1435,1455,1475,1495,1515,1535,1555,1575,1595,1615,
1635,1655,1675,1695,1715,1735,1755,1775,1795,1815,1835,1855,1875,1895,1915,1935,1955,1975,1995],

[16,36,56,76,96,116,136,156,176,196,216,236,256,276,296,316,336,356,376,396,416,436,456,476,496,516,536,556,576,596,6
```

```
16,636,656,676,696,716,736,756,776,796,816,836,856,876,896,916,936,956,976,996,1016,1036,1056,1076,1096,1116,1136,115
6,1176,1196,1216,1236,1256,1276,1296,1316,1336,1356,1376,1396,1416,1436,1456,1476,1496,1516,1536,1556,1576,1596,1616,
1636,1656,1676,1696,1716,1736,1756,1776,1796,1816,1836,1856,1876,1896,1916,1936,1956,1976,1996],

[17,37,57,77,97,117,137,157,177,197,217,237,257,277,297,317,337,357,377,397,417,437,457,477,497,517,537,557,577,597,6
17,637,657,677,697,717,737,757,777,797,817,837,857,877,897,917,937,957,977,997,1017,1037,1057,1077,1097,1117,1137,115
7,1177,1197,1217,1237,1257,1277,1297,1317,1337,1357,1377,1397,1417,1437,1457,1477,1497,1517,1537,1557,1577,1597,1617,
1637,1657,1677,1697,1717,1737,1757,1777,1797,1817,1837,1857,1877,1897,1917,1937,1957,1977,1997],

[18,38,58,78,98,118,138,158,178,198,218,238,258,278,298,318,338,358,378,398,418,438,458,478,498,518,538,558,578,598,6
18,638,658,678,698,718,738,758,778,798,818,838,858,878,898,918,938,958,978,998,1018,1038,1058,1078,1098,1118,1138,115
8,1178,1198,1218,1238,1258,1278,1298,1318,1338,1358,1378,1398,1418,1438,1458,1478,1498,1518,1538,1558,1578,1598,1618,
1638,1658,1678,1698,1718,1738,1758,1778,1798,1818,1838,1858,1878,1898,1918,1938,1958,1978,1998],

[19,39,59,79,99,119,139,159,179,199,219,239,259,279,299,319,339,359,379,399,419,439,459,479,499,519,539,559,579,599,6
19,639,659,679,699,719,739,759,779,799,819,839,859,879,899,919,939,959,979,999,1019,1039,1059,1079,1099,1119,1139,115
9,1179,1199,1219,1239,1259,1279,1299,1319,1339,1359,1379,1399,1419,1439,1459,1479,1499,1519,1539,1559,1579,1599,1619,
1639,1659,1679,1699,1719,1739,1759,1779,1799,1819,1839,1859,1879,1899,1919,1939,1959,1979,1999]
        ])

combined_bead_names = ['11-12', '12-13', '13-14', '14-15', '15-16', '11-21', '21-22', '22-23', '23-24', '24-25', '25-
26', '26-27',  '27-28', '21-31', '31-32', '32-33', '33-34', '34-35', '35-36', '36-37']



angle_connections_list = np.array([
        [111,112,113],      [112,113,114],     [113,114,115],     [114,115,116],     [112,111,121],     [111,121,131],
         [111,121,122],     [121,122,123],     [122,123,124],     [123,124,125],     [124,125,126],     [125,126,127],
         [126,127,128],     [122,121,131],     [121,131,132],     [131,132,133],     [132,133,134],     [133,134,135],
         [134,135,136],     [135,136,137],     [211,212,213],     [212,213,214],     [213,214,215],     [214,215,216],
         [212,211,221],     [211,221,231],     [211,221,222],     [221,222,223],     [222,223,224],     [223,224,225],
         [224,225,226],     [225,226,227],     [226,227,228],     [222,221,231],     [221,231,232],     [231,232,233],
         [232,233,234],     [233,234,235],     [234,235,236],     [235,236,237],     [311,312,313],     [312,313,314],
         [313,314,315],     [314,315,316],     [312,311,321],     [311,321,331],     [311,321,322],     [321,322,323],
         [322,323,324],     [323,324,325],     [324,325,326],     [325,326,327],     [326,327,328],     [322,321,331],
         [321,331,332],     [331,332,333],     [332,333,334],     [333,334,335],     [334,335,336],     [335,336,337],
         [411,412,413],     [412,413,414],     [413,414,415],     [414,415,416],     [412,411,421],     [411,421,431],
         [411,421,422],     [421,422,423],     [422,423,424],     [423,424,425],     [424,425,426],     [425,426,427],
         [426,427,428],     [422,421,431],     [421,431,432],     [431,432,433],     [432,433,434],     [433,434,435],
         [434,435,436],     [435,436,437],     [511,512,513],     [512,513,514],     [513,514,515],     [514,515,516],
         [512,511,521],     [511,521,531],     [511,521,522],     [521,522,523],     [522,523,524],     [523,524,525],
         [524,525,526],     [525,526,527],     [526,527,528],     [522,521,531],     [521,531,532],     [531,532,533],
         [532,533,534],     [533,534,535],     [534,535,536],     [535,536,537],     [611,612,613],     [612,613,614],
         [613,614,615],     [614,615,616],     [612,611,621],     [611,621,631],     [611,621,622],     [621,622,623],
         [622,623,624],     [623,624,625],     [624,625,626],     [625,626,627],     [626,627,628],     [622,621,631],
         [621,631,632],     [631,632,633],     [632,633,634],     [633,634,635],     [634,635,636],     [635,636,637],
         [711,712,713],     [712,713,714],     [713,714,715],     [714,715,716],     [712,711,721],     [711,721,731],
         [711,721,722],     [721,722,723],     [722,723,724],     [723,724,725],     [724,725,726],     [725,726,727],
         [726,727,728],     [722,721,731],     [721,731,732],     [731,732,733],     [732,733,734],     [733,734,735],
         [734,735,736],     [735,736,737],     [811,812,813],     [812,813,814],     [813,814,815],     [814,815,816],
         [812,811,821],     [811,821,831],     [811,821,822],     [821,822,823],     [822,823,824],     [823,824,825],
         [824,825,826],     [825,826,827],     [826,827,828],     [822,821,831],     [821,831,832],     [831,832,833],
         [832,833,834],     [833,834,835],     [834,835,836],     [835,836,837],     [911,912,913],     [912,913,914],
         [913,914,915],     [914,915,916],     [912,911,921],     [911,921,931],     [911,921,922],     [921,922,923],
         [922,923,924],     [923,924,925],     [924,925,926],     [925,926,927],     [926,927,928],     [922,921,931],
         [921,931,932],     [931,932,933],     [932,933,934],     [933,934,935],     [934,935,936],     [935,936,937],
         [1011,1012,1013],  [1012,1013,1014],  [1013,1014,1015],  [1014,1015,1016],  [1012,1011,1021],
         [1011,1021,1031],  [1011,1021,1022],  [1021,1022,1023],  [1022,1023,1024],  [1023,1024,1025],
         [1024,1025,1026],  [1025,1026,1027],  [1026,1027,1028],  [1022,1021,1031],  [1021,1031,1032],
         [1031,1032,1033],  [1032,1033,1034],  [1033,1034,1035],  [1034,1035,1036],  [1035,1036,1037],
         [1111,1112,1113],  [1112,1113,1114],  [1113,1114,1115],  [1114,1115,1116],  [1112,1111,1121],
         [1111,1121,1131],  [1111,1121,1122],  [1121,1122,1123],  [1122,1123,1124],  [1123,1124,1125],
         [1124,1125,1126],  [1125,1126,1127],  [1126,1127,1128],  [1122,1121,1131],  [1121,1131,1132],
         [1131,1132,1133],  [1132,1133,1134],  [1133,1134,1135],  [1134,1135,1136],  [1135,1136,1137],
         [1211,1212,1213],  [1212,1213,1214],  [1213,1214,1215],  [1214,1215,1216],  [1212,1211,1221],
         [1211,1221,1231],  [1211,1221,1222],  [1221,1222,1223],  [1222,1223,1224],  [1223,1224,1225],
         [1224,1225,1226],  [1225,1226,1227],  [1226,1227,1228],  [1222,1221,1231],  [1221,1231,1232],
         [1231,1232,1233],  [1232,1233,1234],  [1233,1234,1235],  [1234,1235,1236],  [1235,1236,1237],
         [1311,1312,1313],  [1312,1313,1314],  [1313,1314,1315],  [1314,1315,1316],  [1312,1311,1321],
         [1311,1321,1331],  [1311,1321,1322],  [1321,1322,1323],  [1322,1323,1324],  [1323,1324,1325],
         [1324,1325,1326],  [1325,1326,1327],  [1326,1327,1328],  [1322,1321,1331],  [1321,1331,1332],
         [1331,1332,1333],  [1332,1333,1334],  [1333,1334,1335],  [1334,1335,1336],  [1335,1336,1337],
         [1411,1412,1413],  [1412,1413,1414],  [1413,1414,1415],  [1414,1415,1416],  [1412,1411,1421],
         [1411,1421,1431],  [1411,1421,1422],  [1421,1422,1423],  [1422,1423,1424],  [1423,1424,1425],
         [1424,1425,1426],  [1425,1426,1427],  [1426,1427,1428],  [1422,1421,1431],  [1421,1431,1432],
         [1431,1432,1433],  [1432,1433,1434],  [1433,1434,1435],  [1434,1435,1436],  [1435,1436,1437],
         [1511,1512,1513],  [1512,1513,1514],  [1513,1514,1515],  [1514,1515,1516],  [1512,1511,1521],
         [1511,1521,1531],  [1511,1521,1522],  [1521,1522,1523],  [1522,1523,1524],  [1523,1524,1525],
         [1524,1525,1526],  [1525,1526,1527],  [1526,1527,1528],  [1522,1521,1531],  [1521,1531,1532],
         [1531,1532,1533],  [1532,1533,1534],  [1533,1534,1535],  [1534,1535,1536],  [1535,1536,1537],
         [1611,1612,1613],  [1612,1613,1614],  [1613,1614,1615],  [1614,1615,1616],  [1612,1611,1621],
         [1611,1621,1631],  [1611,1621,1622],  [1621,1622,1623],  [1622,1623,1624],  [1623,1624,1625],
         [1624,1625,1626],  [1625,1626,1627],  [1626,1627,1628],  [1622,1621,1631],  [1621,1631,1632],
         [1631,1632,1633],  [1632,1633,1634],  [1633,1634,1635],  [1634,1635,1636],  [1635,1636,1637],
         [1711,1712,1713],  [1712,1713,1714],  [1713,1714,1715],  [1714,1715,1716],  [1712,1711,1721],
```

[1711,1721,1731], [1711,1721,1722], [1721,1722,1723], [1722,1723,1724], [1723,1724,1725],
[1724,1725,1726], [1725,1726,1727], [1726,1727,1728], [1722,1721,1731], [1721,1731,1732],
[1731,1732,1733], [1732,1733,1734], [1733,1734,1735], [1734,1735,1736], [1735,1736,1737],
[1811,1812,1813], [1812,1813,1814], [1813,1814,1815], [1814,1815,1816], [1812,1811,1821],
[1811,1821,1831], [1811,1821,1822], [1821,1822,1823], [1822,1823,1824], [1823,1824,1825],
[1824,1825,1826], [1825,1826,1827], [1826,1827,1828], [1822,1821,1831], [1821,1831,1832],
[1831,1832,1833], [1832,1833,1834], [1833,1834,1835], [1834,1835,1836], [1835,1836,1837],
[1911,1912,1913], [1912,1913,1914], [1913,1914,1915], [1914,1915,1916], [1912,1911,1921],
[1911,1921,1931], [1911,1921,1922], [1921,1922,1923], [1922,1923,1924], [1923,1924,1925],
[1924,1925,1926], [1925,1926,1927], [1926,1927,1928], [1922,1921,1931], [1921,1931,1932],
[1931,1932,1933], [1932,1933,1934], [1933,1934,1935], [1934,1935,1936], [1935,1936,1937],
[2011,2012,2013], [2012,2013,2014], [2013,2014,2015], [2014,2015,2016], [2012,2011,2021],
[2011,2021,2031], [2011,2021,2022], [2021,2022,2023], [2022,2023,2024], [2023,2024,2025],
[2024,2025,2026], [2025,2026,2027], [2026,2027,2028], [2022,2021,2031], [2021,2031,2032],
[2031,2032,2033], [2032,2033,2034], [2033,2034,2035], [2034,2035,2036], [2035,2036,2037],
[2111,2112,2113], [2112,2113,2114], [2113,2114,2115], [2114,2115,2116], [2112,2111,2121],
[2111,2121,2131], [2111,2121,2122], [2121,2122,2123], [2122,2123,2124], [2123,2124,2125],
[2124,2125,2126], [2125,2126,2127], [2126,2127,2128], [2122,2121,2131], [2121,2131,2132],
[2131,2132,2133], [2132,2133,2134], [2133,2134,2135], [2134,2135,2136], [2135,2136,2137],
[2211,2212,2213], [2212,2213,2214], [2213,2214,2215], [2214,2215,2216], [2212,2211,2221],
[2211,2221,2231], [2211,2221,2222], [2221,2222,2223], [2222,2223,2224], [2223,2224,2225],
[2224,2225,2226], [2225,2226,2227], [2226,2227,2228], [2222,2221,2231], [2221,2231,2232],
[2231,2232,2233], [2232,2233,2234], [2233,2234,2235], [2234,2235,2236], [2235,2236,2237],
[2311,2312,2313], [2312,2313,2314], [2313,2314,2315], [2314,2315,2316], [2312,2311,2321],
[2311,2321,2331], [2311,2321,2322], [2321,2322,2323], [2322,2323,2324], [2323,2324,2325],
[2324,2325,2326], [2325,2326,2327], [2326,2327,2328], [2322,2321,2331], [2321,2331,2332],
[2331,2332,2333], [2332,2333,2334], [2333,2334,2335], [2334,2335,2336], [2335,2336,2337],
[2411,2412,2413], [2412,2413,2414], [2413,2414,2415], [2414,2415,2416], [2412,2411,2421],
[2411,2421,2431], [2411,2421,2422], [2421,2422,2423], [2422,2423,2424], [2423,2424,2425],
[2424,2425,2426], [2425,2426,2427], [2426,2427,2428], [2422,2421,2431], [2421,2431,2432],
[2431,2432,2433], [2432,2433,2434], [2433,2434,2435], [2434,2435,2436], [2435,2436,2437],
[2511,2512,2513], [2512,2513,2514], [2513,2514,2515], [2514,2515,2516], [2512,2511,2521],
[2511,2521,2531], [2511,2521,2522], [2521,2522,2523], [2522,2523,2524], [2523,2524,2525],
[2524,2525,2526], [2525,2526,2527], [2526,2527,2528], [2522,2521,2531], [2521,2531,2532],
[2531,2532,2533], [2532,2533,2534], [2533,2534,2535], [2534,2535,2536], [2535,2536,2537],
[2611,2612,2613], [2612,2613,2614], [2613,2614,2615], [2614,2615,2616], [2612,2611,2621],
[2611,2621,2631], [2611,2621,2622], [2621,2622,2623], [2622,2623,2624], [2623,2624,2625],
[2624,2625,2626], [2625,2626,2627], [2626,2627,2628], [2622,2621,2631], [2621,2631,2632],
[2631,2632,2633], [2632,2633,2634], [2633,2634,2635], [2634,2635,2636], [2635,2636,2637],
[2711,2712,2713], [2712,2713,2714], [2713,2714,2715], [2714,2715,2716], [2712,2711,2721],
[2711,2721,2731], [2711,2721,2722], [2721,2722,2723], [2722,2723,2724], [2723,2724,2725],
[2724,2725,2726], [2725,2726,2727], [2726,2727,2728], [2722,2721,2731], [2721,2731,2732],
[2731,2732,2733], [2732,2733,2734], [2733,2734,2735], [2734,2735,2736], [2735,2736,2737],
[2811,2812,2813], [2812,2813,2814], [2813,2814,2815], [2814,2815,2816], [2812,2811,2821],
[2811,2821,2831], [2811,2821,2822], [2821,2822,2823], [2822,2823,2824], [2823,2824,2825],
[2824,2825,2826], [2825,2826,2827], [2826,2827,2828], [2822,2821,2831], [2821,2831,2832],
[2831,2832,2833], [2832,2833,2834], [2833,2834,2835], [2834,2835,2836], [2835,2836,2837],
[2911,2912,2913], [2912,2913,2914], [2913,2914,2915], [2914,2915,2916], [2912,2911,2921],
[2911,2921,2931], [2911,2921,2922], [2921,2922,2923], [2922,2923,2924], [2923,2924,2925],
[2924,2925,2926], [2925,2926,2927], [2926,2927,2928], [2922,2921,2931], [2921,2931,2932],
[2931,2932,2933], [2932,2933,2934], [2933,2934,2935], [2934,2935,2936], [2935,2936,2937],
[3011,3012,3013], [3012,3013,3014], [3013,3014,3015], [3014,3015,3016], [3012,3011,3021],
[3011,3021,3031], [3011,3021,3022], [3021,3022,3023], [3022,3023,3024], [3023,3024,3025],
[3024,3025,3026], [3025,3026,3027], [3026,3027,3028], [3022,3021,3031], [3021,3031,3032],
[3031,3032,3033], [3032,3033,3034], [3033,3034,3035], [3034,3035,3036], [3035,3036,3037],
[3111,3112,3113], [3112,3113,3114], [3113,3114,3115], [3114,3115,3116], [3112,3111,3121],
[3111,3121,3131], [3111,3121,3122], [3121,3122,3123], [3122,3123,3124], [3123,3124,3125],
[3124,3125,3126], [3125,3126,3127], [3126,3127,3128], [3122,3121,3131], [3121,3131,3132],
[3131,3132,3133], [3132,3133,3134], [3133,3134,3135], [3134,3135,3136], [3135,3136,3137],
[3211,3212,3213], [3212,3213,3214], [3213,3214,3215], [3214,3215,3216], [3212,3211,3221],
[3211,3221,3231], [3211,3221,3222], [3221,3222,3223], [3222,3223,3224], [3223,3224,3225],
[3224,3225,3226], [3225,3226,3227], [3226,3227,3228], [3222,3221,3231], [3221,3231,3232],
[3231,3232,3233], [3232,3233,3234], [3233,3234,3235], [3234,3235,3236], [3235,3236,3237],
[3311,3312,3313], [3312,3313,3314], [3313,3314,3315], [3314,3315,3316], [3312,3311,3321],
[3311,3321,3331], [3311,3321,3322], [3321,3322,3323], [3322,3323,3324], [3323,3324,3325],
[3324,3325,3326], [3325,3326,3327], [3326,3327,3328], [3322,3321,3331], [3321,3331,3332],
[3331,3332,3333], [3332,3333,3334], [3333,3334,3335], [3334,3335,3336], [3335,3336,3337],
[3411,3412,3413], [3412,3413,3414], [3413,3414,3415], [3414,3415,3416], [3412,3411,3421],
[3411,3421,3431], [3411,3421,3422], [3421,3422,3423], [3422,3423,3424], [3423,3424,3425],
[3424,3425,3426], [3425,3426,3427], [3426,3427,3428], [3422,3421,3431], [3421,3431,3432],
[3431,3432,3433], [3432,3433,3434], [3433,3434,3435], [3434,3435,3436], [3435,3436,3437],
[3511,3512,3513], [3512,3513,3514], [3513,3514,3515], [3514,3515,3516], [3512,3511,3521],
[3511,3521,3531], [3511,3521,3522], [3521,3522,3523], [3522,3523,3524], [3523,3524,3525],
[3524,3525,3526], [3525,3526,3527], [3526,3527,3528], [3522,3521,3531], [3521,3531,3532],
[3531,3532,3533], [3532,3533,3534], [3533,3534,3535], [3534,3535,3536], [3535,3536,3537],
[3611,3612,3613], [3612,3613,3614], [3613,3614,3615], [3614,3615,3616], [3612,3611,3621],
[3611,3621,3631], [3611,3621,3622], [3621,3622,3623], [3622,3623,3624], [3623,3624,3625],
[3624,3625,3626], [3625,3626,3627], [3626,3627,3628], [3622,3621,3631], [3621,3631,3632],
[3631,3632,3633], [3632,3633,3634], [3633,3634,3635], [3634,3635,3636], [3635,3636,3637],
[3711,3712,3713], [3712,3713,3714], [3713,3714,3715], [3714,3715,3716], [3712,3711,3721],
[3711,3721,3731], [3711,3721,3722], [3721,3722,3723], [3722,3723,3724], [3723,3724,3725],
[3724,3725,3726], [3725,3726,3727], [3726,3727,3728], [3722,3721,3731], [3721,3731,3732],
[3731,3732,3733], [3732,3733,3734], [3733,3734,3735], [3734,3735,3736], [3735,3736,3737],
[3811,3812,3813], [3812,3813,3814], [3813,3814,3815], [3814,3815,3816], [3812,3811,3821],
[3811,3821,3831], [3811,3821,3822], [3821,3822,3823], [3822,3823,3824], [3823,3824,3825],

[3824,3825,3826], [3825,3826,3827], [3826,3827,3828], [3822,3821,3831], [3821,3831,3832],
[3831,3832,3833], [3832,3833,3834], [3833,3834,3835], [3834,3835,3836], [3835,3836,3837],
[3911,3912,3913], [3912,3913,3914], [3913,3914,3915], [3914,3915,3916], [3912,3911,3921],
[3911,3921,3931], [3911,3921,3922], [3921,3922,3923], [3922,3923,3924], [3923,3924,3925],
[3924,3925,3926], [3925,3926,3927], [3926,3927,3928], [3922,3921,3931], [3921,3931,3932],
[3931,3932,3933], [3932,3933,3934], [3933,3934,3935], [3934,3935,3936], [3935,3936,3937],
[4011,4012,4013], [4012,4013,4014], [4013,4014,4015], [4014,4015,4016], [4012,4011,4021],
[4011,4021,4031], [4011,4021,4022], [4021,4022,4023], [4022,4023,4024], [4023,4024,4025],
[4024,4025,4026], [4025,4026,4027], [4026,4027,4028], [4022,4021,4031], [4021,4031,4032],
[4031,4032,4033], [4032,4033,4034], [4033,4034,4035], [4034,4035,4036], [4035,4036,4037],
[4111,4112,4113], [4112,4113,4114], [4113,4114,4115], [4114,4115,4116], [4112,4111,4121],
[4111,4121,4131], [4111,4121,4122], [4121,4122,4123], [4122,4123,4124], [4123,4124,4125],
[4124,4125,4126], [4125,4126,4127], [4126,4127,4128], [4122,4121,4131], [4121,4131,4132],
[4131,4132,4133], [4132,4133,4134], [4133,4134,4135], [4134,4135,4136], [4135,4136,4137],
[4211,4212,4213], [4212,4213,4214], [4213,4214,4215], [4214,4215,4216], [4212,4211,4221],
[4211,4221,4231], [4211,4221,4222], [4221,4222,4223], [4222,4223,4224], [4223,4224,4225],
[4224,4225,4226], [4225,4226,4227], [4226,4227,4228], [4222,4221,4231], [4221,4231,4232],
[4231,4232,4233], [4232,4233,4234], [4233,4234,4235], [4234,4235,4236], [4235,4236,4237],
[4311,4312,4313], [4312,4313,4314], [4313,4314,4315], [4314,4315,4316], [4312,4311,4321],
[4311,4321,4331], [4311,4321,4322], [4321,4322,4323], [4322,4323,4324], [4323,4324,4325],
[4324,4325,4326], [4325,4326,4327], [4326,4327,4328], [4322,4321,4331], [4321,4331,4332],
[4331,4332,4333], [4332,4333,4334], [4333,4334,4335], [4334,4335,4336], [4335,4336,4337],
[4411,4412,4413], [4412,4413,4414], [4413,4414,4415], [4414,4415,4416], [4412,4411,4421],
[4411,4421,4431], [4411,4421,4422], [4421,4422,4423], [4422,4423,4424], [4423,4424,4425],
[4424,4425,4426], [4425,4426,4427], [4426,4427,4428], [4422,4421,4431], [4421,4431,4432],
[4431,4432,4433], [4432,4433,4434], [4433,4434,4435], [4434,4435,4436], [4435,4436,4437],
[4511,4512,4513], [4512,4513,4514], [4513,4514,4515], [4514,4515,4516], [4512,4511,4521],
[4511,4521,4531], [4511,4521,4522], [4521,4522,4523], [4522,4523,4524], [4523,4524,4525],
[4524,4525,4526], [4525,4526,4527], [4526,4527,4528], [4522,4521,4531], [4521,4531,4532],
[4531,4532,4533], [4532,4533,4534], [4533,4534,4535], [4534,4535,4536], [4535,4536,4537],
[4611,4612,4613], [4612,4613,4614], [4613,4614,4615], [4614,4615,4616], [4612,4611,4621],
[4611,4621,4631], [4611,4621,4622], [4621,4622,4623], [4622,4623,4624], [4623,4624,4625],
[4624,4625,4626], [4625,4626,4627], [4626,4627,4628], [4622,4621,4631], [4621,4631,4632],
[4631,4632,4633], [4632,4633,4634], [4633,4634,4635], [4634,4635,4636], [4635,4636,4637],
[4711,4712,4713], [4712,4713,4714], [4713,4714,4715], [4714,4715,4716], [4712,4711,4721],
[4711,4721,4731], [4711,4721,4722], [4721,4722,4723], [4722,4723,4724], [4723,4724,4725],
[4724,4725,4726], [4725,4726,4727], [4726,4727,4728], [4722,4721,4731], [4721,4731,4732],
[4731,4732,4733], [4732,4733,4734], [4733,4734,4735], [4734,4735,4736], [4735,4736,4737],
[4811,4812,4813], [4812,4813,4814], [4813,4814,4815], [4814,4815,4816], [4812,4811,4821],
[4811,4821,4831], [4811,4821,4822], [4821,4822,4823], [4822,4823,4824], [4823,4824,4825],
[4824,4825,4826], [4825,4826,4827], [4826,4827,4828], [4822,4821,4831], [4821,4831,4832],
[4831,4832,4833], [4832,4833,4834], [4833,4834,4835], [4834,4835,4836], [4835,4836,4837],
[4911,4912,4913], [4912,4913,4914], [4913,4914,4915], [4914,4915,4916], [4912,4911,4921],
[4911,4921,4931], [4911,4921,4922], [4921,4922,4923], [4922,4923,4924], [4923,4924,4925],
[4924,4925,4926], [4925,4926,4927], [4926,4927,4928], [4922,4921,4931], [4921,4931,4932],
[4931,4932,4933], [4932,4933,4934], [4933,4934,4935], [4934,4935,4936], [4935,4936,4937],
[5011,5012,5013], [5012,5013,5014], [5013,5014,5015], [5014,5015,5016], [5012,5011,5021],
[5011,5021,5031], [5011,5021,5022], [5021,5022,5023], [5022,5023,5024], [5023,5024,5025],
[5024,5025,5026], [5025,5026,5027], [5026,5027,5028], [5022,5021,5031], [5021,5031,5032],
[5031,5032,5033], [5032,5033,5034], [5033,5034,5035], [5034,5035,5036], [5035,5036,5037],
[5111,5112,5113], [5112,5113,5114], [5113,5114,5115], [5114,5115,5116], [5112,5111,5121],
[5111,5121,5131], [5111,5121,5122], [5121,5122,5123], [5122,5123,5124], [5123,5124,5125],
[5124,5125,5126], [5125,5126,5127], [5126,5127,5128], [5122,5121,5131], [5121,5131,5132],
[5131,5132,5133], [5132,5133,5134], [5133,5134,5135], [5134,5135,5136], [5135,5136,5137],
[5211,5212,5213], [5212,5213,5214], [5213,5214,5215], [5214,5215,5216], [5212,5211,5221],
[5211,5221,5231], [5211,5221,5222], [5221,5222,5223], [5222,5223,5224], [5223,5224,5225],
[5224,5225,5226], [5225,5226,5227], [5226,5227,5228], [5222,5221,5231], [5221,5231,5232],
[5231,5232,5233], [5232,5233,5234], [5233,5234,5235], [5234,5235,5236], [5235,5236,5237],
[5311,5312,5313], [5312,5313,5314], [5313,5314,5315], [5314,5315,5316], [5312,5311,5321],
[5311,5321,5331], [5311,5321,5322], [5321,5322,5323], [5322,5323,5324], [5323,5324,5325],
[5324,5325,5326], [5325,5326,5327], [5326,5327,5328], [5322,5321,5331], [5321,5331,5332],
[5331,5332,5333], [5332,5333,5334], [5333,5334,5335], [5334,5335,5336], [5335,5336,5337],
[5411,5412,5413], [5412,5413,5414], [5413,5414,5415], [5414,5415,5416], [5412,5411,5421],
[5411,5421,5431], [5411,5421,5422], [5421,5422,5423], [5422,5423,5424], [5423,5424,5425],
[5424,5425,5426], [5425,5426,5427], [5426,5427,5428], [5422,5421,5431], [5421,5431,5432],
[5431,5432,5433], [5432,5433,5434], [5433,5434,5435], [5434,5435,5436], [5435,5436,5437],
[5511,5512,5513], [5512,5513,5514], [5513,5514,5515], [5514,5515,5516], [5512,5511,5521],
[5511,5521,5531], [5511,5521,5522], [5521,5522,5523], [5522,5523,5524], [5523,5524,5525],
[5524,5525,5526], [5525,5526,5527], [5526,5527,5528], [5522,5521,5531], [5521,5531,5532],
[5531,5532,5533], [5532,5533,5534], [5533,5534,5535], [5534,5535,5536], [5535,5536,5537],
[5611,5612,5613], [5612,5613,5614], [5613,5614,5615], [5614,5615,5616], [5612,5611,5621],
[5611,5621,5631], [5611,5621,5622], [5621,5622,5623], [5622,5623,5624], [5623,5624,5625],
[5624,5625,5626], [5625,5626,5627], [5626,5627,5628], [5622,5621,5631], [5621,5631,5632],
[5631,5632,5633], [5632,5633,5634], [5633,5634,5635], [5634,5635,5636], [5635,5636,5637],
[5711,5712,5713], [5712,5713,5714], [5713,5714,5715], [5714,5715,5716], [5712,5711,5721],
[5711,5721,5731], [5711,5721,5722], [5721,5722,5723], [5722,5723,5724], [5723,5724,5725],
[5724,5725,5726], [5725,5726,5727], [5726,5727,5728], [5722,5721,5731], [5721,5731,5732],
[5731,5732,5733], [5732,5733,5734], [5733,5734,5735], [5734,5735,5736], [5735,5736,5737],
[5811,5812,5813], [5812,5813,5814], [5813,5814,5815], [5814,5815,5816], [5812,5811,5821],
[5811,5821,5831], [5811,5821,5822], [5821,5822,5823], [5822,5823,5824], [5823,5824,5825],
[5824,5825,5826], [5825,5826,5827], [5826,5827,5828], [5822,5821,5831], [5821,5831,5832],
[5831,5832,5833], [5832,5833,5834], [5833,5834,5835], [5834,5835,5836], [5835,5836,5837],
[5911,5912,5913], [5912,5913,5914], [5913,5914,5915], [5914,5915,5916], [5912,5911,5921],
[5911,5921,5931], [5911,5921,5922], [5921,5922,5923], [5922,5923,5924], [5923,5924,5925],
[5924,5925,5926], [5925,5926,5927], [5926,5927,5928], [5922,5921,5931], [5921,5931,5932],

[5931,5932,5933], [5932,5933,5934], [5933,5934,5935], [5934,5935,5936], [5935,5936,5937],
[6011,6012,6013], [6012,6013,6014], [6013,6014,6015], [6014,6015,6016], [6012,6011,6021],
[6011,6021,6031], [6011,6021,6022], [6021,6022,6023], [6022,6023,6024], [6023,6024,6025],
[6024,6025,6026], [6025,6026,6027], [6026,6027,6028], [6022,6021,6031], [6021,6031,6032],
[6031,6032,6033], [6032,6033,6034], [6033,6034,6035], [6034,6035,6036], [6035,6036,6037],
[6111,6112,6113], [6112,6113,6114], [6113,6114,6115], [6114,6115,6116], [6112,6111,6121],
[6111,6121,6131], [6111,6121,6122], [6121,6122,6123], [6122,6123,6124], [6123,6124,6125],
[6124,6125,6126], [6125,6126,6127], [6126,6127,6128], [6122,6121,6131], [6121,6131,6132],
[6131,6132,6133], [6132,6133,6134], [6133,6134,6135], [6134,6135,6136], [6135,6136,6137],
[6211,6212,6213], [6212,6213,6214], [6213,6214,6215], [6214,6215,6216], [6212,6211,6221],
[6211,6221,6231], [6211,6221,6222], [6221,6222,6223], [6222,6223,6224], [6223,6224,6225],
[6224,6225,6226], [6225,6226,6227], [6226,6227,6228], [6222,6221,6231], [6221,6231,6232],
[6231,6232,6233], [6232,6233,6234], [6233,6234,6235], [6234,6235,6236], [6235,6236,6237],
[6311,6312,6313], [6312,6313,6314], [6313,6314,6315], [6314,6315,6316], [6312,6311,6321],
[6311,6321,6331], [6311,6321,6322], [6321,6322,6323], [6322,6323,6324], [6323,6324,6325],
[6324,6325,6326], [6325,6326,6327], [6326,6327,6328], [6322,6321,6331], [6321,6331,6332],
[6331,6332,6333], [6332,6333,6334], [6333,6334,6335], [6334,6335,6336], [6335,6336,6337],
[6411,6412,6413], [6412,6413,6414], [6413,6414,6415], [6414,6415,6416], [6412,6411,6421],
[6411,6421,6431], [6411,6421,6422], [6421,6422,6423], [6422,6423,6424], [6423,6424,6425],
[6424,6425,6426], [6425,6426,6427], [6426,6427,6428], [6422,6421,6431], [6421,6431,6432],
[6431,6432,6433], [6432,6433,6434], [6433,6434,6435], [6434,6435,6436], [6435,6436,6437],
[6511,6512,6513], [6512,6513,6514], [6513,6514,6515], [6514,6515,6516], [6512,6511,6521],
[6511,6521,6531], [6511,6521,6522], [6521,6522,6523], [6522,6523,6524], [6523,6524,6525],
[6524,6525,6526], [6525,6526,6527], [6526,6527,6528], [6522,6521,6531], [6521,6531,6532],
[6531,6532,6533], [6532,6533,6534], [6533,6534,6535], [6534,6535,6536], [6535,6536,6537],
[6611,6612,6613], [6612,6613,6614], [6613,6614,6615], [6614,6615,6616], [6612,6611,6621],
[6611,6621,6631], [6611,6621,6622], [6621,6622,6623], [6622,6623,6624], [6623,6624,6625],
[6624,6625,6626], [6625,6626,6627], [6626,6627,6628], [6622,6621,6631], [6621,6631,6632],
[6631,6632,6633], [6632,6633,6634], [6633,6634,6635], [6634,6635,6636], [6635,6636,6637],
[6711,6712,6713], [6712,6713,6714], [6713,6714,6715], [6714,6715,6716], [6712,6711,6721],
[6711,6721,6731], [6711,6721,6722], [6721,6722,6723], [6722,6723,6724], [6723,6724,6725],
[6724,6725,6726], [6725,6726,6727], [6726,6727,6728], [6722,6721,6731], [6721,6731,6732],
[6731,6732,6733], [6732,6733,6734], [6733,6734,6735], [6734,6735,6736], [6735,6736,6737],
[6811,6812,6813], [6812,6813,6814], [6813,6814,6815], [6814,6815,6816], [6812,6811,6821],
[6811,6821,6831], [6811,6821,6822], [6821,6822,6823], [6822,6823,6824], [6823,6824,6825],
[6824,6825,6826], [6825,6826,6827], [6826,6827,6828], [6822,6821,6831], [6821,6831,6832],
[6831,6832,6833], [6832,6833,6834], [6833,6834,6835], [6834,6835,6836], [6835,6836,6837],
[6911,6912,6913], [6912,6913,6914], [6913,6914,6915], [6914,6915,6916], [6912,6911,6921],
[6911,6921,6931], [6911,6921,6922], [6921,6922,6923], [6922,6923,6924], [6923,6924,6925],
[6924,6925,6926], [6925,6926,6927], [6926,6927,6928], [6922,6921,6931], [6921,6931,6932],
[6931,6932,6933], [6932,6933,6934], [6933,6934,6935], [6934,6935,6936], [6935,6936,6937],
[7011,7012,7013], [7012,7013,7014], [7013,7014,7015], [7014,7015,7016], [7012,7011,7021],
[7011,7021,7031], [7011,7021,7022], [7021,7022,7023], [7022,7023,7024], [7023,7024,7025],
[7024,7025,7026], [7025,7026,7027], [7026,7027,7028], [7022,7021,7031], [7021,7031,7032],
[7031,7032,7033], [7032,7033,7034], [7033,7034,7035], [7034,7035,7036], [7035,7036,7037],
[7111,7112,7113], [7112,7113,7114], [7113,7114,7115], [7114,7115,7116], [7112,7111,7121],
[7111,7121,7131], [7111,7121,7122], [7121,7122,7123], [7122,7123,7124], [7123,7124,7125],
[7124,7125,7126], [7125,7126,7127], [7126,7127,7128], [7122,7121,7131], [7121,7131,7132],
[7131,7132,7133], [7132,7133,7134], [7133,7134,7135], [7134,7135,7136], [7135,7136,7137],
[7211,7212,7213], [7212,7213,7214], [7213,7214,7215], [7214,7215,7216], [7212,7211,7221],
[7211,7221,7231], [7211,7221,7222], [7221,7222,7223], [7222,7223,7224], [7223,7224,7225],
[7224,7225,7226], [7225,7226,7227], [7226,7227,7228], [7222,7221,7231], [7221,7231,7232],
[7231,7232,7233], [7232,7233,7234], [7233,7234,7235], [7234,7235,7236], [7235,7236,7237],
[7311,7312,7313], [7312,7313,7314], [7313,7314,7315], [7314,7315,7316], [7312,7311,7321],
[7311,7321,7331], [7311,7321,7322], [7321,7322,7323], [7322,7323,7324], [7323,7324,7325],
[7324,7325,7326], [7325,7326,7327], [7326,7327,7328], [7322,7321,7331], [7321,7331,7332],
[7331,7332,7333], [7332,7333,7334], [7333,7334,7335], [7334,7335,7336], [7335,7336,7337],
[7411,7412,7413], [7412,7413,7414], [7413,7414,7415], [7414,7415,7416], [7412,7411,7421],
[7411,7421,7431], [7411,7421,7422], [7421,7422,7423], [7422,7423,7424], [7423,7424,7425],
[7424,7425,7426], [7425,7426,7427], [7426,7427,7428], [7422,7421,7431], [7421,7431,7432],
[7431,7432,7433], [7432,7433,7434], [7433,7434,7435], [7434,7435,7436], [7435,7436,7437],
[7511,7512,7513], [7512,7513,7514], [7513,7514,7515], [7514,7515,7516], [7512,7511,7521],
[7511,7521,7531], [7511,7521,7522], [7521,7522,7523], [7522,7523,7524], [7523,7524,7525],
[7524,7525,7526], [7525,7526,7527], [7526,7527,7528], [7522,7521,7531], [7521,7531,7532],
[7531,7532,7533], [7532,7533,7534], [7533,7534,7535], [7534,7535,7536], [7535,7536,7537],
[7611,7612,7613], [7612,7613,7614], [7613,7614,7615], [7614,7615,7616], [7612,7611,7621],
[7611,7621,7631], [7611,7621,7622], [7621,7622,7623], [7622,7623,7624], [7623,7624,7625],
[7624,7625,7626], [7625,7626,7627], [7626,7627,7628], [7622,7621,7631], [7621,7631,7632],
[7631,7632,7633], [7632,7633,7634], [7633,7634,7635], [7634,7635,7636], [7635,7636,7637],
[7711,7712,7713], [7712,7713,7714], [7713,7714,7715], [7714,7715,7716], [7712,7711,7721],
[7711,7721,7731], [7711,7721,7722], [7721,7722,7723], [7722,7723,7724], [7723,7724,7725],
[7724,7725,7726], [7725,7726,7727], [7726,7727,7728], [7722,7721,7731], [7721,7731,7732],
[7731,7732,7733], [7732,7733,7734], [7733,7734,7735], [7734,7735,7736], [7735,7736,7737],
[7811,7812,7813], [7812,7813,7814], [7813,7814,7815], [7814,7815,7816], [7812,7811,7821],
[7811,7821,7831], [7811,7821,7822], [7821,7822,7823], [7822,7823,7824], [7823,7824,7825],
[7824,7825,7826], [7825,7826,7827], [7826,7827,7828], [7822,7821,7831], [7821,7831,7832],
[7831,7832,7833], [7832,7833,7834], [7833,7834,7835], [7834,7835,7836], [7835,7836,7837],
[7911,7912,7913], [7912,7913,7914], [7913,7914,7915], [7914,7915,7916], [7912,7911,7921],
[7911,7921,7931], [7911,7921,7922], [7921,7922,7923], [7922,7923,7924], [7923,7924,7925],
[7924,7925,7926], [7925,7926,7927], [7926,7927,7928], [7922,7921,7931], [7921,7931,7932],
[7931,7932,7933], [7932,7933,7934], [7933,7934,7935], [7934,7935,7936], [7935,7936,7937],
[8011,8012,8013], [8012,8013,8014], [8013,8014,8015], [8014,8015,8016], [8012,8011,8021],
[8011,8021,8031], [8011,8021,8022], [8021,8022,8023], [8022,8023,8024], [8023,8024,8025],
[8024,8025,8026], [8025,8026,8027], [8026,8027,8028], [8022,8021,8031], [8021,8031,8032],
[8031,8032,8033], [8032,8033,8034], [8033,8034,8035], [8034,8035,8036], [8035,8036,8037],

```
        [8111,8112,8113], [8112,8113,8114], [8113,8114,8115], [8114,8115,8116], [8112,8111,8121],
        [8111,8121,8131], [8111,8121,8122], [8121,8122,8123], [8122,8123,8124], [8123,8124,8125],
        [8124,8125,8126], [8125,8126,8127], [8126,8127,8128], [8122,8121,8131], [8121,8131,8132],
        [8131,8132,8133], [8132,8133,8134], [8133,8134,8135], [8134,8135,8136], [8135,8136,8137],
        [8211,8212,8213], [8212,8213,8214], [8213,8214,8215], [8214,8215,8216], [8212,8211,8221],
        [8211,8221,8231], [8211,8221,8222], [8221,8222,8223], [8222,8223,8224], [8223,8224,8225],
        [8224,8225,8226], [8225,8226,8227], [8226,8227,8228], [8222,8221,8231], [8221,8231,8232],
        [8231,8232,8233], [8232,8233,8234], [8233,8234,8235], [8234,8235,8236], [8235,8236,8237],
        [8311,8312,8313], [8312,8313,8314], [8313,8314,8315], [8314,8315,8316], [8312,8311,8321],
        [8311,8321,8331], [8311,8321,8322], [8321,8322,8323], [8322,8323,8324], [8323,8324,8325],
        [8324,8325,8326], [8325,8326,8327], [8326,8327,8328], [8322,8321,8331], [8321,8331,8332],
        [8331,8332,8333], [8332,8333,8334], [8333,8334,8335], [8334,8335,8336], [8335,8336,8337],
        [8411,8412,8413], [8412,8413,8414], [8413,8414,8415], [8414,8415,8416], [8412,8411,8421],
        [8411,8421,8431], [8411,8421,8422], [8421,8422,8423], [8422,8423,8424], [8423,8424,8425],
        [8424,8425,8426], [8425,8426,8427], [8426,8427,8428], [8422,8421,8431], [8421,8431,8432],
        [8431,8432,8433], [8432,8433,8434], [8433,8434,8435], [8434,8435,8436], [8435,8436,8437],
        [8511,8512,8513], [8512,8513,8514], [8513,8514,8515], [8514,8515,8516], [8512,8511,8521],
        [8511,8521,8531], [8511,8521,8522], [8521,8522,8523], [8522,8523,8524], [8523,8524,8525],
        [8524,8525,8526], [8525,8526,8527], [8526,8527,8528], [8522,8521,8531], [8521,8531,8532],
        [8531,8532,8533], [8532,8533,8534], [8533,8534,8535], [8534,8535,8536], [8535,8536,8537],
        [8611,8612,8613], [8612,8613,8614], [8613,8614,8615], [8614,8615,8616], [8612,8611,8621],
        [8611,8621,8631], [8611,8621,8622], [8621,8622,8623], [8622,8623,8624], [8623,8624,8625],
        [8624,8625,8626], [8625,8626,8627], [8626,8627,8628], [8622,8621,8631], [8621,8631,8632],
        [8631,8632,8633], [8632,8633,8634], [8633,8634,8635], [8634,8635,8636], [8635,8636,8637],
        [8711,8712,8713], [8712,8713,8714], [8713,8714,8715], [8714,8715,8716], [8712,8711,8721],
        [8711,8721,8731], [8711,8721,8722], [8721,8722,8723], [8722,8723,8724], [8723,8724,8725],
        [8724,8725,8726], [8725,8726,8727], [8726,8727,8728], [8722,8721,8731], [8721,8731,8732],
        [8731,8732,8733], [8732,8733,8734], [8733,8734,8735], [8734,8735,8736], [8735,8736,8737],
        [8811,8812,8813], [8812,8813,8814], [8813,8814,8815], [8814,8815,8816], [8812,8811,8821],
        [8811,8821,8831], [8811,8821,8822], [8821,8822,8823], [8822,8823,8824], [8823,8824,8825],
        [8824,8825,8826], [8825,8826,8827], [8826,8827,8828], [8822,8821,8831], [8821,8831,8832],
        [8831,8832,8833], [8832,8833,8834], [8833,8834,8835], [8834,8835,8836], [8835,8836,8837],
        [8911,8912,8913], [8912,8913,8914], [8913,8914,8915], [8914,8915,8916], [8912,8911,8921],
        [8911,8921,8931], [8911,8921,8922], [8921,8922,8923], [8922,8923,8924], [8923,8924,8925],
        [8924,8925,8926], [8925,8926,8927], [8926,8927,8928], [8922,8921,8931], [8921,8931,8932],
        [8931,8932,8933], [8932,8933,8934], [8933,8934,8935], [8934,8935,8936], [8935,8936,8937],
        [9011,9012,9013], [9012,9013,9014], [9013,9014,9015], [9014,9015,9016], [9012,9011,9021],
        [9011,9021,9031], [9011,9021,9022], [9021,9022,9023], [9022,9023,9024], [9023,9024,9025],
        [9024,9025,9026], [9025,9026,9027], [9026,9027,9028], [9022,9021,9031], [9021,9031,9032],
        [9031,9032,9033], [9032,9033,9034], [9033,9034,9035], [9034,9035,9036], [9035,9036,9037],
        [9111,9112,9113], [9112,9113,9114], [9113,9114,9115], [9114,9115,9116], [9112,9111,9121],
        [9111,9121,9131], [9111,9121,9122], [9121,9122,9123], [9122,9123,9124], [9123,9124,9125],
        [9124,9125,9126], [9125,9126,9127], [9126,9127,9128], [9122,9121,9131], [9121,9131,9132],
        [9131,9132,9133], [9132,9133,9134], [9133,9134,9135], [9134,9135,9136], [9135,9136,9137],
        [9211,9212,9213], [9212,9213,9214], [9213,9214,9215], [9214,9215,9216], [9212,9211,9221],
        [9211,9221,9231], [9211,9221,9222], [9221,9222,9223], [9222,9223,9224], [9223,9224,9225],
        [9224,9225,9226], [9225,9226,9227], [9226,9227,9228], [9222,9221,9231], [9221,9231,9232],
        [9231,9232,9233], [9232,9233,9234], [9233,9234,9235], [9234,9235,9236], [9235,9236,9237],
        [9311,9312,9313], [9312,9313,9314], [9313,9314,9315], [9314,9315,9316], [9312,9311,9321],
        [9311,9321,9331], [9311,9321,9322], [9321,9322,9323], [9322,9323,9324], [9323,9324,9325],
        [9324,9325,9326], [9325,9326,9327], [9326,9327,9328], [9322,9321,9331], [9321,9331,9332],
        [9331,9332,9333], [9332,9333,9334], [9333,9334,9335], [9334,9335,9336], [9335,9336,9337],
        [9411,9412,9413], [9412,9413,9414], [9413,9414,9415], [9414,9415,9416], [9412,9411,9421],
        [9411,9421,9431], [9411,9421,9422], [9421,9422,9423], [9422,9423,9424], [9423,9424,9425],
        [9424,9425,9426], [9425,9426,9427], [9426,9427,9428], [9422,9421,9431], [9421,9431,9432],
        [9431,9432,9433], [9432,9433,9434], [9433,9434,9435], [9434,9435,9436], [9435,9436,9437],
        [9511,9512,9513], [9512,9513,9514], [9513,9514,9515], [9514,9515,9516], [9512,9511,9521],
        [9511,9521,9531], [9511,9521,9522], [9521,9522,9523], [9522,9523,9524], [9523,9524,9525],
        [9524,9525,9526], [9525,9526,9527], [9526,9527,9528], [9522,9521,9531], [9521,9531,9532],
        [9531,9532,9533], [9532,9533,9534], [9533,9534,9535], [9534,9535,9536], [9535,9536,9537],
        [9611,9612,9613], [9612,9613,9614], [9613,9614,9615], [9614,9615,9616], [9612,9611,9621],
        [9611,9621,9631], [9611,9621,9622], [9621,9622,9623], [9622,9623,9624], [9623,9624,9625],
        [9624,9625,9626], [9625,9626,9627], [9626,9627,9628], [9622,9621,9631], [9621,9631,9632],
        [9631,9632,9633], [9632,9633,9634], [9633,9634,9635], [9634,9635,9636], [9635,9636,9637],
        [9711,9712,9713], [9712,9713,9714], [9713,9714,9715], [9714,9715,9716], [9712,9711,9721],
        [9711,9721,9731], [9711,9721,9722], [9721,9722,9723], [9722,9723,9724], [9723,9724,9725],
        [9724,9725,9726], [9725,9726,9727], [9726,9727,9728], [9722,9721,9731], [9721,9731,9732],
        [9731,9732,9733], [9732,9733,9734], [9733,9734,9735], [9734,9735,9736], [9735,9736,9737],
        [9811,9812,9813], [9812,9813,9814], [9813,9814,9815], [9814,9815,9816], [9812,9811,9821],
        [9811,9821,9831], [9811,9821,9822], [9821,9822,9823], [9822,9823,9824], [9823,9824,9825],
        [9824,9825,9826], [9825,9826,9827], [9826,9827,9828], [9822,9821,9831], [9821,9831,9832],
        [9831,9832,9833], [9832,9833,9834], [9833,9834,9835], [9834,9835,9836], [9835,9836,9837],
        [9911,9912,9913], [9912,9913,9914], [9913,9914,9915], [9914,9915,9916], [9912,9911,9921],
        [9911,9921,9931], [9911,9921,9922], [9921,9922,9923], [9922,9923,9924], [9923,9924,9925],
        [9924,9925,9926], [9925,9926,9927], [9926,9927,9928], [9922,9921,9931], [9921,9931,9932],
        [9931,9932,9933], [9932,9933,9934], [9933,9934,9935], [9934,9935,9936], [9935,9936,9937],
        [10011,10012,10013],      [10012,10013,10014],      [10013,10014,10015],      [10014,10015,10016],
        [10012,10011,10021],      [10011,10021,10031],      [10011,10021,10022],      [10021,10022,10023],
        [10022,10023,10024],      [10023,10024,10025],      [10024,10025,10026],      [10025,10026,10027],
        [10026,10027,10028],      [10022,10021,10031],      [10021,10031,10032],      [10031,10032,10033],
        [10032,10033,10034],      [10033,10034,10035],      [10034,10035,10036],      [10035,10036,10037],
         ])

angle_combination_indices = np.array([
```

[0,20,40,60,80,100,120,140,160,180,200,220,240,260,280,300,320,340,360,380,400,420,440,460,480,500,520,540,560,580,60
0,620,640,660,680,700,720,740,760,780,800,820,840,860,880,900,920,940,960,980,1000,1020,1040,1060,1080,1100,1120,1140
,1160,1180,1200,1220,1240,1260,1280,1300,1320,1340,1360,1380,1400,1420,1440,1460,1480,1500,1520,1540,1560,1580,1600,1
620,1640,1660,1680,1700,1720,1740,1760,1780,1800,1820,1840,1860,1880,1900,1920,1940,1960,1980],

[1,21,41,61,81,101,121,141,161,181,201,221,241,261,281,301,321,341,361,381,401,421,441,461,481,501,521,541,561,581,60
1,621,641,661,681,701,721,741,761,781,801,821,841,861,881,901,921,941,961,981,1001,1021,1041,1061,1081,1101,1121,1141
,1161,1181,1201,1221,1241,1261,1281,1301,1321,1341,1361,1381,1401,1421,1441,1461,1481,1501,1521,1541,1561,1581,1601,1
621,1641,1661,1681,1701,1721,1741,1761,1781,1801,1821,1841,1861,1881,1901,1921,1941,1961,1981],

[2,22,42,62,82,102,122,142,162,182,202,222,242,262,282,302,322,342,362,382,402,422,442,462,482,502,522,542,562,582,60
2,622,642,662,682,702,722,742,762,782,802,822,842,862,882,902,922,942,962,982,1002,1022,1042,1062,1082,1102,1122,1142
,1162,1182,1202,1222,1242,1262,1282,1302,1322,1342,1362,1382,1402,1422,1442,1462,1482,1502,1522,1542,1562,1582,1602,1
622,1642,1662,1682,1702,1722,1742,1762,1782,1802,1822,1842,1862,1882,1902,1922,1942,1962,1982],

[3,23,43,63,83,103,123,143,163,183,203,223,243,263,283,303,323,343,363,383,403,423,443,463,483,503,523,543,563,583,60
3,623,643,663,683,703,723,743,763,783,803,823,843,863,883,903,923,943,963,983,1003,1023,1043,1063,1083,1103,1123,1143
,1163,1183,1203,1223,1243,1263,1283,1303,1323,1343,1363,1383,1403,1423,1443,1463,1483,1503,1523,1543,1563,1583,1603,1
623,1643,1663,1683,1703,1723,1743,1763,1783,1803,1823,1843,1863,1883,1903,1923,1943,1963,1983],

[4,24,44,64,84,104,124,144,164,184,204,224,244,264,284,304,324,344,364,384,404,424,444,464,484,504,524,544,564,584,60
4,624,644,664,684,704,724,744,764,784,804,824,844,864,884,904,924,944,964,984,1004,1024,1044,1064,1084,1104,1124,1144
,1164,1184,1204,1224,1244,1264,1284,1304,1324,1344,1364,1384,1404,1424,1444,1464,1484,1504,1524,1544,1564,1584,1604,1
624,1644,1664,1684,1704,1724,1744,1764,1784,1804,1824,1844,1864,1884,1904,1924,1944,1964,1984],

[5,25,45,65,85,105,125,145,165,185,205,225,245,265,285,305,325,345,365,385,405,425,445,465,485,505,525,545,565,585,60
5,625,645,665,685,705,725,745,765,785,805,825,845,865,885,905,925,945,965,985,1005,1025,1045,1065,1085,1105,1125,1145
,1165,1185,1205,1225,1245,1265,1285,1305,1325,1345,1365,1385,1405,1425,1445,1465,1485,1505,1525,1545,1565,1585,1605,1
625,1645,1665,1685,1705,1725,1745,1765,1785,1805,1825,1845,1865,1885,1905,1925,1945,1965,1985],

[6,26,46,66,86,106,126,146,166,186,206,226,246,266,286,306,326,346,366,386,406,426,446,466,486,506,526,546,566,586,60
6,626,646,666,686,706,726,746,766,786,806,826,846,866,886,906,926,946,966,986,1006,1026,1046,1066,1086,1106,1126,1146
,1166,1186,1206,1226,1246,1266,1286,1306,1326,1346,1366,1386,1406,1426,1446,1466,1486,1506,1526,1546,1566,1586,1606,1
626,1646,1666,1686,1706,1726,1746,1766,1786,1806,1826,1846,1866,1886,1906,1926,1946,1966,1986],

[7,27,47,67,87,107,127,147,167,187,207,227,247,267,287,307,327,347,367,387,407,427,447,467,487,507,527,547,567,587,60
7,627,647,667,687,707,727,747,767,787,807,827,847,867,887,907,927,947,967,987,1007,1027,1047,1067,1087,1107,1127,1147
,1167,1187,1207,1227,1247,1267,1287,1307,1327,1347,1367,1387,1407,1427,1447,1467,1487,1507,1527,1547,1567,1587,1607,1
627,1647,1667,1687,1707,1727,1747,1767,1787,1807,1827,1847,1867,1887,1907,1927,1947,1967,1987],

[8,28,48,68,88,108,128,148,168,188,208,228,248,268,288,308,328,348,368,388,408,428,448,468,488,508,528,548,568,588,60
8,628,648,668,688,708,728,748,768,788,808,828,848,868,888,908,928,948,968,988,1008,1028,1048,1068,1088,1108,1128,1148
,1168,1188,1208,1228,1248,1268,1288,1308,1328,1348,1368,1388,1408,1428,1448,1468,1488,1508,1528,1548,1568,1588,1608,1
628,1648,1668,1688,1708,1728,1748,1768,1788,1808,1828,1848,1868,1888,1908,1928,1948,1968,1988],

[9,29,49,69,89,109,129,149,169,189,209,229,249,269,289,309,329,349,369,389,409,429,449,469,489,509,529,549,569,589,60
9,629,649,669,689,709,729,749,769,789,809,829,849,869,889,909,929,949,969,989,1009,1029,1049,1069,1089,1109,1129,1149
,1169,1189,1209,1229,1249,1269,1289,1309,1329,1349,1369,1389,1409,1429,1449,1469,1489,1509,1529,1549,1569,1589,1609,1
629,1649,1669,1689,1709,1729,1749,1769,1789,1809,1829,1849,1869,1889,1909,1929,1949,1969,1989],

[10,30,50,70,90,110,130,150,170,190,210,230,250,270,290,310,330,350,370,390,410,430,450,470,490,510,530,550,570,590,6
10,630,650,670,690,710,730,750,770,790,810,830,850,870,890,910,930,950,970,990,1010,1030,1050,1070,1090,1110,1130,115
0,1170,1190,1210,1230,1250,1270,1290,1310,1330,1350,1370,1390,1410,1430,1450,1470,1490,1510,1530,1550,1570,1590,1610,
1630,1650,1670,1690,1710,1730,1750,1770,1790,1810,1830,1850,1870,1890,1910,1930,1950,1970,1990],

[11,31,51,71,91,111,131,151,171,191,211,231,251,271,291,311,331,351,371,391,411,431,451,471,491,511,531,551,571,591,6
11,631,651,671,691,711,731,751,771,791,811,831,851,871,891,911,931,951,971,991,1011,1031,1051,1071,1091,1111,1131,115
1,1171,1191,1211,1231,1251,1271,1291,1311,1331,1351,1371,1391,1411,1431,1451,1471,1491,1511,1531,1551,1571,1591,1611,
1631,1651,1671,1691,1711,1731,1751,1771,1791,1811,1831,1851,1871,1891,1911,1931,1951,1971,1991],

[12,32,52,72,92,112,132,152,172,192,212,232,252,272,292,312,332,352,372,392,412,432,452,472,492,512,532,552,572,592,6
12,632,652,672,692,712,732,752,772,792,812,832,852,872,892,912,932,952,972,992,1012,1032,1052,1072,1092,1112,1132,115
2,1172,1192,1212,1232,1252,1272,1292,1312,1332,1352,1372,1392,1412,1432,1452,1472,1492,1512,1532,1552,1572,1592,1612,
1632,1652,1672,1692,1712,1732,1752,1772,1792,1812,1832,1852,1872,1892,1912,1932,1952,1972,1992],

[13,33,53,73,93,113,133,153,173,193,213,233,253,273,293,313,333,353,373,393,413,433,453,473,493,513,533,553,573,593,6
13,633,653,673,693,713,733,753,773,793,813,833,853,873,893,913,933,953,973,993,1013,1033,1053,1073,1093,1113,1133,115
3,1173,1193,1213,1233,1253,1273,1293,1313,1333,1353,1373,1393,1413,1433,1453,1473,1493,1513,1533,1553,1573,1593,1613,
1633,1653,1673,1693,1713,1733,1753,1773,1793,1813,1833,1853,1873,1893,1913,1933,1953,1973,1993],

[14,34,54,74,94,114,134,154,174,194,214,234,254,274,294,314,334,354,374,394,414,434,454,474,494,514,534,554,574,594,6
14,634,654,674,694,714,734,754,774,794,814,834,854,874,894,914,934,954,974,994,1014,1034,1054,1074,1094,1114,1134,115
4,1174,1194,1214,1234,1254,1274,1294,1314,1334,1354,1374,1394,1414,1434,1454,1474,1494,1514,1534,1554,1574,1594,1614,
1634,1654,1674,1694,1714,1734,1754,1774,1794,1814,1834,1854,1874,1894,1914,1934,1954,1974,1994],

[15,35,55,75,95,115,135,155,175,195,215,235,255,275,295,315,335,355,375,395,415,435,455,475,495,515,535,555,575,595,6
15,635,655,675,695,715,735,755,775,795,815,835,855,875,895,915,935,955,975,995,1015,1035,1055,1075,1095,1115,1135,115
5,1175,1195,1215,1235,1255,1275,1295,1315,1335,1355,1375,1395,1415,1435,1455,1475,1495,1515,1535,1555,1575,1595,1615,
1635,1655,1675,1695,1715,1735,1755,1775,1795,1815,1835,1855,1875,1895,1915,1935,1955,1975,1995],

[16,36,56,76,96,116,136,156,176,196,216,236,256,276,296,316,336,356,376,396,416,436,456,476,496,516,536,556,576,596,6
16,636,656,676,696,716,736,756,776,796,816,836,856,876,896,916,936,956,976,996,1016,1036,1056,1076,1096,1116,1136,115
6,1176,1196,1216,1236,1256,1276,1296,1316,1336,1356,1376,1396,1416,1436,1456,1476,1496,1516,1536,1556,1576,1596,1616,
1636,1656,1676,1696,1716,1736,1756,1776,1796,1816,1836,1856,1876,1896,1916,1936,1956,1976,1996],

```
[17,37,57,77,97,117,137,157,177,197,217,237,257,277,297,317,337,357,377,397,417,437,457,477,497,517,537,557,577,597,6
17,637,657,677,697,717,737,757,777,797,817,837,857,877,897,917,937,957,977,997,1017,1037,1057,1077,1097,1117,1137,115
7,1177,1197,1217,1237,1257,1277,1297,1317,1337,1357,1377,1397,1417,1437,1457,1477,1497,1517,1537,1557,1577,1597,1617,
1637,1657,1677,1697,1717,1737,1757,1777,1797,1817,1837,1857,1877,1897,1917,1937,1957,1977,1997],

[18,38,58,78,98,118,138,158,178,198,218,238,258,278,298,318,338,358,378,398,418,438,458,478,498,518,538,558,578,598,6
18,638,658,678,698,718,738,758,778,798,818,838,858,878,898,918,938,958,978,998,1018,1038,1058,1078,1098,1118,1138,115
8,1178,1198,1218,1238,1258,1278,1298,1318,1338,1358,1378,1398,1418,1438,1458,1478,1498,1518,1538,1558,1578,1598,1618,
1638,1658,1678,1698,1718,1738,1758,1778,1798,1818,1838,1858,1878,1898,1918,1938,1958,1978,1998],

[19,39,59,79,99,119,139,159,179,199,219,239,259,279,299,319,339,359,379,399,419,439,459,479,499,519,539,559,579,599,6
19,639,659,679,699,719,739,759,779,799,819,839,859,879,899,919,939,959,979,999,1019,1039,1059,1079,1099,1119,1139,115
9,1179,1199,1219,1239,1259,1279,1299,1319,1339,1359,1379,1399,1419,1439,1459,1479,1499,1519,1539,1559,1579,1599,1619,
1639,1659,1679,1699,1719,1739,1759,1779,1799,1819,1839,1859,1879,1899,1919,1939,1959,1979,1999]  ])

combined_angle_names = ['11-12-13', '12-13-14', '13-14-15', '14-15-16', '12-11-21', '11-21-31', '11-21-22', '21-22-
23', '22-23-24', '23-24-25', '24-25-26', '25-26-27', '26-27-28', '22-21-31', '21-31-32', '31-32-33', '32-33-34', '33-
34-35', '34-35-36', '35-36-37'  ]



dihedral_connections_list = np.array([
       [113,114,115,116],  [112,113,114,115], [111,112,113,114], [121,111,112,113], [122,121,111,112],
        [131,121,111,112], [111,121,131,132], [111,121,122,123], [121,122,123,124], [122,123,124,125],
        [123,124,125,126], [124,125,126,127], [125,126,127,128], [131,121,122,123], [122,121,131,132],
        [121,131,132,133], [131,132,133,134], [132,133,134,135], [133,134,135,136], [134,135,136,137],
        [213,214,215,216], [212,213,214,215], [211,212,213,214], [221,211,212,213], [222,221,211,212],
        [231,221,211,212], [211,221,231,232], [211,221,222,223], [221,222,223,224], [222,223,224,225],
        [223,224,225,226], [224,225,226,227], [225,226,227,228], [231,221,222,223], [222,221,231,232],
        [221,231,232,233], [231,232,233,234], [232,233,234,235], [233,234,235,236], [234,235,236,237],
        [313,314,315,316], [312,313,314,315], [311,312,313,314], [321,311,312,313], [322,321,311,312],
        [331,321,311,312], [311,321,331,332], [311,321,322,323], [321,322,323,324], [322,323,324,325],
        [323,324,325,326], [324,325,326,327], [325,326,327,328], [331,321,322,323], [322,321,331,332],
        [321,331,332,333], [331,332,333,334], [332,333,334,335], [333,334,335,336], [334,335,336,337],
        [413,414,415,416], [412,413,414,415], [411,412,413,414], [421,411,412,413], [422,421,411,412],
        [431,421,411,412], [411,421,431,432], [411,421,422,423], [421,422,423,424], [422,423,424,425],
        [423,424,425,426], [424,425,426,427], [425,426,427,428], [431,421,422,423], [422,421,431,432],
        [421,431,432,433], [431,432,433,434], [432,433,434,435], [433,434,435,436], [434,435,436,437],
        [513,514,515,516], [512,513,514,515], [511,512,513,514], [521,511,512,513], [522,521,511,512],
        [531,521,511,512], [511,521,531,532], [511,521,522,523], [521,522,523,524], [522,523,524,525],
        [523,524,525,526], [524,525,526,527], [525,526,527,528], [531,521,522,523], [522,521,531,532],
        [521,531,532,533], [531,532,533,534], [532,533,534,535], [533,534,535,536], [534,535,536,537],
        [613,614,615,616], [612,613,614,615], [611,612,613,614], [621,611,612,613], [622,621,611,612],
        [631,621,611,612], [611,621,631,632], [611,621,622,623], [621,622,623,624], [622,623,624,625],
        [623,624,625,626], [624,625,626,627], [625,626,627,628], [631,621,622,623], [622,621,631,632],
        [621,631,632,633], [631,632,633,634], [632,633,634,635], [633,634,635,636], [634,635,636,637],
        [713,714,715,716], [712,713,714,715], [711,712,713,714], [721,711,712,713], [722,721,711,712],
        [731,721,711,712], [711,721,731,732], [711,721,722,723], [721,722,723,724], [722,723,724,725],
        [723,724,725,726], [724,725,726,727], [725,726,727,728], [731,721,722,723], [722,721,731,732],
        [721,731,732,733], [731,732,733,734], [732,733,734,735], [733,734,735,736], [734,735,736,737],
        [813,814,815,816], [812,813,814,815], [811,812,813,814], [821,811,812,813], [822,821,811,812],
        [831,821,811,812], [811,821,831,832], [811,821,822,823], [821,822,823,824], [822,823,824,825],
        [823,824,825,826], [824,825,826,827], [825,826,827,828], [831,821,822,823], [822,821,831,832],
        [821,831,832,833], [831,832,833,834], [832,833,834,835], [833,834,835,836], [834,835,836,837],
        [913,914,915,916], [912,913,914,915], [911,912,913,914], [921,911,912,913], [922,921,911,912],
        [931,921,911,912], [911,921,931,932], [911,921,922,923], [921,922,923,924], [922,923,924,925],
        [923,924,925,926], [924,925,926,927], [925,926,927,928], [931,921,922,923], [922,921,931,932],
        [921,931,932,933], [931,932,933,934], [932,933,934,935], [933,934,935,936], [934,935,936,937],
        [1013,1014,1015,1016],      [1012,1013,1014,1015],      [1011,1012,1013,1014],      [1021,1011,1012,1013],
        [1022,1021,1011,1012],      [1031,1021,1011,1012],      [1011,1021,1031,1032],      [1011,1021,1022,1023],
        [1021,1022,1023,1024],      [1022,1023,1024,1025],      [1023,1024,1025,1026],      [1024,1025,1026,1027],
        [1025,1026,1027,1028],      [1031,1021,1022,1023],      [1022,1021,1031,1032],      [1021,1031,1032,1033],
        [1031,1032,1033,1034],      [1032,1033,1034,1035],      [1033,1034,1035,1036],      [1034,1035,1036,1037],
        [1113,1114,1115,1116],      [1112,1113,1114,1115],      [1111,1112,1113,1114],      [1121,1111,1112,1113],
        [1122,1121,1111,1112],      [1131,1121,1111,1112],      [1111,1121,1131,1132],      [1111,1121,1122,1123],
        [1121,1122,1123,1124],      [1122,1123,1124,1125],      [1123,1124,1125,1126],      [1124,1125,1126,1127],
        [1125,1126,1127,1128],      [1131,1121,1122,1123],      [1122,1121,1131,1132],      [1121,1131,1132,1133],
        [1131,1132,1133,1134],      [1132,1133,1134,1135],      [1133,1134,1135,1136],      [1134,1135,1136,1137],
        [1213,1214,1215,1216],      [1212,1213,1214,1215],      [1211,1212,1213,1214],      [1221,1211,1212,1213],
        [1222,1221,1211,1212],      [1231,1221,1211,1212],      [1211,1221,1231,1232],      [1211,1221,1222,1223],
        [1221,1222,1223,1224],      [1222,1223,1224,1225],      [1223,1224,1225,1226],      [1224,1225,1226,1227],
        [1225,1226,1227,1228],      [1231,1221,1222,1223],      [1222,1221,1231,1232],      [1221,1231,1232,1233],
        [1231,1232,1233,1234],      [1232,1233,1234,1235],      [1233,1234,1235,1236],      [1234,1235,1236,1237],
        [1313,1314,1315,1316],      [1312,1313,1314,1315],      [1311,1312,1313,1314],      [1321,1311,1312,1313],
        [1322,1321,1311,1312],      [1331,1321,1311,1312],      [1311,1321,1331,1332],      [1311,1321,1322,1323],
        [1321,1322,1323,1324],      [1322,1323,1324,1325],      [1323,1324,1325,1326],      [1324,1325,1326,1327],
        [1325,1326,1327,1328],      [1331,1321,1322,1323],      [1322,1321,1331,1332],      [1321,1331,1332,1333],
        [1331,1332,1333,1334],      [1332,1333,1334,1335],      [1333,1334,1335,1336],      [1334,1335,1336,1337],
        [1413,1414,1415,1416],      [1412,1413,1414,1415],      [1411,1412,1413,1414],      [1421,1411,1412,1413],
        [1422,1421,1411,1412],      [1431,1421,1411,1412],      [1411,1421,1431,1432],      [1411,1421,1422,1423],
        [1421,1422,1423,1424],      [1422,1423,1424,1425],      [1423,1424,1425,1426],      [1424,1425,1426,1427],
        [1425,1426,1427,1428],      [1431,1421,1422,1423],      [1422,1421,1431,1432],      [1421,1431,1432,1433],
        [1431,1432,1433,1434],      [1432,1433,1434,1435],      [1433,1434,1435,1436],      [1434,1435,1436,1437],
        [1513,1514,1515,1516],      [1512,1513,1514,1515],      [1511,1512,1513,1514],      [1521,1511,1512,1513],
```

```
[1522,1521,1511,1512],
[1521,1522,1523,1524],
[1525,1526,1527,1528],
[1531,1532,1533,1534],
[1613,1614,1615,1616],
[1622,1621,1611,1612],
[1621,1622,1623,1624],
[1625,1626,1627,1628],
[1631,1632,1633,1634],
[1713,1714,1715,1716],
[1722,1721,1711,1712],
[1721,1722,1723,1724],
[1725,1726,1727,1728],
[1731,1732,1733,1734],
[1813,1814,1815,1816],
[1822,1821,1811,1812],
[1821,1822,1823,1824],
[1825,1826,1827,1828],
[1831,1832,1833,1834],
[1913,1914,1915,1916],
[1922,1921,1911,1912],
[1921,1922,1923,1924],
[1925,1926,1927,1928],
[1931,1932,1933,1934],
[2013,2014,2015,2016],
[2022,2021,2011,2012],
[2021,2022,2023,2024],
[2025,2026,2027,2028],
[2031,2032,2033,2034],
[2113,2114,2115,2116],
[2122,2121,2111,2112],
[2121,2122,2123,2124],
[2125,2126,2127,2128],
[2131,2132,2133,2134],
[2213,2214,2215,2216],
[2222,2221,2211,2212],
[2221,2222,2223,2224],
[2225,2226,2227,2228],
[2231,2232,2233,2234],
[2313,2314,2315,2316],
[2322,2321,2311,2312],
[2321,2322,2323,2324],
[2325,2326,2327,2328],
[2331,2332,2333,2334],
[2413,2414,2415,2416],
[2422,2421,2411,2412],
[2421,2422,2423,2424],
[2425,2426,2427,2428],
[2431,2432,2433,2434],
[2513,2514,2515,2516],
[2522,2521,2511,2512],
[2521,2522,2523,2524],
[2525,2526,2527,2528],
[2531,2532,2533,2534],
[2613,2614,2615,2616],
[2622,2621,2611,2612],
[2621,2622,2623,2624],
[2625,2626,2627,2628],
[2631,2632,2633,2634],
[2713,2714,2715,2716],
[2722,2721,2711,2712],
[2721,2722,2723,2724],
[2725,2726,2727,2728],
[2731,2732,2733,2734],
[2813,2814,2815,2816],
[2822,2821,2811,2812],
[2821,2822,2823,2824],
[2825,2826,2827,2828],
[2831,2832,2833,2834],
[2913,2914,2915,2916],
[2922,2921,2911,2912],
[2921,2922,2923,2924],
[2925,2926,2927,2928],
[2931,2932,2933,2934],
[3013,3014,3015,3016],
[3022,3021,3011,3012],
[3021,3022,3023,3024],
[3025,3026,3027,3028],
[3031,3032,3033,3034],
[3113,3114,3115,3116],
[3122,3121,3111,3112],
[3121,3122,3123,3124],
[3125,3126,3127,3128],
[3131,3132,3133,3134],
[3213,3214,3215,3216],
[1531,1521,1511,1512],
[1522,1523,1524,1525],
[1531,1521,1522,1523],
[1532,1533,1534,1535],
[1612,1613,1614,1615],
[1631,1621,1611,1612],
[1622,1623,1624,1625],
[1631,1621,1622,1623],
[1632,1633,1634,1635],
[1712,1713,1714,1715],
[1731,1721,1711,1712],
[1722,1723,1724,1725],
[1731,1721,1722,1723],
[1732,1733,1734,1735],
[1812,1813,1814,1815],
[1831,1821,1811,1812],
[1822,1823,1824,1825],
[1831,1821,1822,1823],
[1832,1833,1834,1835],
[1912,1913,1914,1915],
[1931,1921,1911,1912],
[1922,1923,1924,1925],
[1931,1921,1922,1923],
[1932,1933,1934,1935],
[2012,2013,2014,2015],
[2031,2021,2011,2012],
[2022,2023,2024,2025],
[2031,2021,2022,2023],
[2032,2033,2034,2035],
[2112,2113,2114,2115],
[2131,2121,2111,2112],
[2122,2123,2124,2125],
[2131,2121,2122,2123],
[2132,2133,2134,2135],
[2212,2213,2214,2215],
[2231,2221,2211,2212],
[2222,2223,2224,2225],
[2231,2221,2222,2223],
[2232,2233,2234,2235],
[2312,2313,2314,2315],
[2331,2321,2311,2312],
[2322,2323,2324,2325],
[2331,2321,2322,2323],
[2332,2333,2334,2335],
[2412,2413,2414,2415],
[2431,2421,2411,2412],
[2422,2423,2424,2425],
[2431,2421,2422,2423],
[2432,2433,2434,2435],
[2512,2513,2514,2515],
[2531,2521,2511,2512],
[2522,2523,2524,2525],
[2531,2521,2522,2523],
[2532,2533,2534,2535],
[2612,2613,2614,2615],
[2631,2621,2611,2612],
[2622,2623,2624,2625],
[2631,2621,2622,2623],
[2632,2633,2634,2635],
[2712,2713,2714,2715],
[2731,2721,2711,2712],
[2722,2723,2724,2725],
[2731,2721,2722,2723],
[2732,2733,2734,2735],
[2812,2813,2814,2815],
[2831,2821,2811,2812],
[2822,2823,2824,2825],
[2831,2821,2822,2823],
[2832,2833,2834,2835],
[2912,2913,2914,2915],
[2931,2921,2911,2912],
[2922,2923,2924,2925],
[2931,2921,2922,2923],
[2932,2933,2934,2935],
[3012,3013,3014,3015],
[3031,3021,3011,3012],
[3022,3023,3024,3025],
[3031,3021,3022,3023],
[3032,3033,3034,3035],
[3112,3113,3114,3115],
[3131,3121,3111,3112],
[3122,3123,3124,3125],
[3131,3121,3122,3123],
[3132,3133,3134,3135],
[3212,3213,3214,3215],
[1511,1521,1531,1532],
[1523,1524,1525,1526],
[1522,1521,1531,1532],
[1533,1534,1535,1536],
[1611,1612,1613,1614],
[1611,1621,1631,1632],
[1623,1624,1625,1626],
[1622,1621,1631,1632],
[1633,1634,1635,1636],
[1711,1712,1713,1714],
[1711,1721,1731,1732],
[1723,1724,1725,1726],
[1722,1721,1731,1732],
[1733,1734,1735,1736],
[1811,1812,1813,1814],
[1811,1821,1831,1832],
[1823,1824,1825,1826],
[1822,1821,1831,1832],
[1833,1834,1835,1836],
[1911,1912,1913,1914],
[1911,1921,1931,1932],
[1923,1924,1925,1926],
[1922,1921,1931,1932],
[1933,1934,1935,1936],
[2011,2012,2013,2014],
[2011,2021,2031,2032],
[2023,2024,2025,2026],
[2022,2021,2031,2032],
[2033,2034,2035,2036],
[2111,2112,2113,2114],
[2111,2121,2131,2132],
[2123,2124,2125,2126],
[2122,2121,2131,2132],
[2133,2134,2135,2136],
[2211,2212,2213,2214],
[2211,2221,2231,2232],
[2223,2224,2225,2226],
[2222,2221,2231,2232],
[2233,2234,2235,2236],
[2311,2312,2313,2314],
[2311,2321,2331,2332],
[2323,2324,2325,2326],
[2322,2321,2331,2332],
[2333,2334,2335,2336],
[2411,2412,2413,2414],
[2411,2421,2431,2432],
[2423,2424,2425,2426],
[2422,2421,2431,2432],
[2433,2434,2435,2436],
[2511,2512,2513,2514],
[2511,2521,2531,2532],
[2523,2524,2525,2526],
[2522,2521,2531,2532],
[2533,2534,2535,2536],
[2611,2612,2613,2614],
[2611,2621,2631,2632],
[2623,2624,2625,2626],
[2622,2621,2631,2632],
[2633,2634,2635,2636],
[2711,2712,2713,2714],
[2711,2721,2731,2732],
[2723,2724,2725,2726],
[2722,2721,2731,2732],
[2733,2734,2735,2736],
[2811,2812,2813,2814],
[2811,2821,2831,2832],
[2823,2824,2825,2826],
[2822,2821,2831,2832],
[2833,2834,2835,2836],
[2911,2912,2913,2914],
[2911,2921,2931,2932],
[2923,2924,2925,2926],
[2922,2921,2931,2932],
[2933,2934,2935,2936],
[3011,3012,3013,3014],
[3011,3021,3031,3032],
[3023,3024,3025,3026],
[3022,3021,3031,3032],
[3033,3034,3035,3036],
[3111,3112,3113,3114],
[3111,3121,3131,3132],
[3123,3124,3125,3126],
[3122,3121,3131,3132],
[3133,3134,3135,3136],
[3211,3212,3213,3214],
[1511,1521,1522,1523],
[1524,1525,1526,1527],
[1521,1531,1532,1533],
[1534,1535,1536,1537],
[1621,1611,1612,1613],
[1611,1621,1622,1623],
[1624,1625,1626,1627],
[1621,1631,1632,1633],
[1634,1635,1636,1637],
[1721,1711,1712,1713],
[1711,1721,1722,1723],
[1724,1725,1726,1727],
[1721,1731,1732,1733],
[1734,1735,1736,1737],
[1821,1811,1812,1813],
[1811,1821,1822,1823],
[1824,1825,1826,1827],
[1821,1831,1832,1833],
[1834,1835,1836,1837],
[1921,1911,1912,1913],
[1911,1921,1922,1923],
[1924,1925,1926,1927],
[1921,1931,1932,1933],
[1934,1935,1936,1937],
[2021,2011,2012,2013],
[2011,2021,2022,2023],
[2024,2025,2026,2027],
[2021,2031,2032,2033],
[2034,2035,2036,2037],
[2121,2111,2112,2113],
[2111,2121,2122,2123],
[2124,2125,2126,2127],
[2121,2131,2132,2133],
[2134,2135,2136,2137],
[2221,2211,2212,2213],
[2211,2221,2222,2223],
[2224,2225,2226,2227],
[2221,2231,2232,2233],
[2234,2235,2236,2237],
[2321,2311,2312,2313],
[2311,2321,2322,2323],
[2324,2325,2326,2327],
[2321,2331,2332,2333],
[2334,2335,2336,2337],
[2421,2411,2412,2413],
[2411,2421,2422,2423],
[2424,2425,2426,2427],
[2421,2431,2432,2433],
[2434,2435,2436,2437],
[2521,2511,2512,2513],
[2511,2521,2522,2523],
[2524,2525,2526,2527],
[2521,2531,2532,2533],
[2534,2535,2536,2537],
[2621,2611,2612,2613],
[2611,2621,2622,2623],
[2624,2625,2626,2627],
[2621,2631,2632,2633],
[2634,2635,2636,2637],
[2721,2711,2712,2713],
[2711,2721,2722,2723],
[2724,2725,2726,2727],
[2721,2731,2732,2733],
[2734,2735,2736,2737],
[2821,2811,2812,2813],
[2811,2821,2822,2823],
[2824,2825,2826,2827],
[2821,2831,2832,2833],
[2834,2835,2836,2837],
[2921,2911,2912,2913],
[2911,2921,2922,2923],
[2924,2925,2926,2927],
[2921,2931,2932,2933],
[2934,2935,2936,2937],
[3021,3011,3012,3013],
[3011,3021,3022,3023],
[3024,3025,3026,3027],
[3021,3031,3032,3033],
[3034,3035,3036,3037],
[3121,3111,3112,3113],
[3111,3121,3122,3123],
[3124,3125,3126,3127],
[3121,3131,3132,3133],
[3134,3135,3136,3137],
[3221,3211,3212,3213],
```

```
[3222,3221,3211,3212],
[3221,3222,3223,3224],
[3225,3226,3227,3228],
[3231,3232,3233,3234],
[3313,3314,3315,3316],
[3322,3321,3311,3312],
[3321,3322,3323,3324],
[3325,3326,3327,3328],
[3331,3332,3333,3334],
[3413,3414,3415,3416],
[3422,3421,3411,3412],
[3421,3422,3423,3424],
[3425,3426,3427,3428],
[3431,3432,3433,3434],
[3513,3514,3515,3516],
[3522,3521,3511,3512],
[3521,3522,3523,3524],
[3525,3526,3527,3528],
[3531,3532,3533,3534],
[3613,3614,3615,3616],
[3622,3621,3611,3612],
[3621,3622,3623,3624],
[3625,3626,3627,3628],
[3631,3632,3633,3634],
[3713,3714,3715,3716],
[3722,3721,3711,3712],
[3721,3722,3723,3724],
[3725,3726,3727,3728],
[3731,3732,3733,3734],
[3813,3814,3815,3816],
[3822,3821,3811,3812],
[3821,3822,3823,3824],
[3825,3826,3827,3828],
[3831,3832,3833,3834],
[3913,3914,3915,3916],
[3922,3921,3911,3912],
[3921,3922,3923,3924],
[3925,3926,3927,3928],
[3931,3932,3933,3934],
[4013,4014,4015,4016],
[4022,4021,4011,4012],
[4021,4022,4023,4024],
[4025,4026,4027,4028],
[4031,4032,4033,4034],
[4113,4114,4115,4116],
[4122,4121,4111,4112],
[4121,4122,4123,4124],
[4125,4126,4127,4128],
[4131,4132,4133,4134],
[4213,4214,4215,4216],
[4222,4221,4211,4212],
[4221,4222,4223,4224],
[4225,4226,4227,4228],
[4231,4232,4233,4234],
[4313,4314,4315,4316],
[4322,4321,4311,4312],
[4321,4322,4323,4324],
[4325,4326,4327,4328],
[4331,4332,4333,4334],
[4413,4414,4415,4416],
[4422,4421,4411,4412],
[4421,4422,4423,4424],
[4425,4426,4427,4428],
[4431,4432,4433,4434],
[4513,4514,4515,4516],
[4522,4521,4511,4512],
[4521,4522,4523,4524],
[4525,4526,4527,4528],
[4531,4532,4533,4534],
[4613,4614,4615,4616],
[4622,4621,4611,4612],
[4621,4622,4623,4624],
[4625,4626,4627,4628],
[4631,4632,4633,4634],
[4713,4714,4715,4716],
[4722,4721,4711,4712],
[4721,4722,4723,4724],
[4725,4726,4727,4728],
[4731,4732,4733,4734],
[4813,4814,4815,4816],
[4822,4821,4811,4812],
[4821,4822,4823,4824],
[4825,4826,4827,4828],
[4831,4832,4833,4834],
[4913,4914,4915,4916],
[3231,3221,3211,3212],
[3222,3223,3224,3225],
[3231,3221,3222,3223],
[3232,3233,3234,3235],
[3312,3313,3314,3315],
[3331,3321,3311,3312],
[3322,3323,3324,3325],
[3331,3321,3322,3323],
[3332,3333,3334,3335],
[3412,3413,3414,3415],
[3431,3421,3411,3412],
[3422,3423,3424,3425],
[3431,3421,3422,3423],
[3432,3433,3434,3435],
[3512,3513,3514,3515],
[3531,3521,3511,3512],
[3522,3523,3524,3525],
[3531,3521,3522,3523],
[3532,3533,3534,3535],
[3612,3613,3614,3615],
[3631,3621,3611,3612],
[3622,3623,3624,3625],
[3631,3621,3622,3623],
[3632,3633,3634,3635],
[3712,3713,3714,3715],
[3731,3721,3711,3712],
[3722,3723,3724,3725],
[3731,3721,3722,3723],
[3732,3733,3734,3735],
[3812,3813,3814,3815],
[3831,3821,3811,3812],
[3822,3823,3824,3825],
[3831,3821,3822,3823],
[3832,3833,3834,3835],
[3912,3913,3914,3915],
[3931,3921,3911,3912],
[3922,3923,3924,3925],
[3931,3921,3922,3923],
[3932,3933,3934,3935],
[4012,4013,4014,4015],
[4031,4021,4011,4012],
[4022,4023,4024,4025],
[4031,4021,4022,4023],
[4032,4033,4034,4035],
[4112,4113,4114,4115],
[4131,4121,4111,4112],
[4122,4123,4124,4125],
[4131,4121,4122,4123],
[4132,4133,4134,4135],
[4212,4213,4214,4215],
[4231,4221,4211,4212],
[4222,4223,4224,4225],
[4231,4221,4222,4223],
[4232,4233,4234,4235],
[4312,4313,4314,4315],
[4331,4321,4311,4312],
[4322,4323,4324,4325],
[4331,4321,4322,4323],
[4332,4333,4334,4335],
[4412,4413,4414,4415],
[4431,4421,4411,4412],
[4422,4423,4424,4425],
[4431,4421,4422,4423],
[4432,4433,4434,4435],
[4512,4513,4514,4515],
[4531,4521,4511,4512],
[4522,4523,4524,4525],
[4531,4521,4522,4523],
[4532,4533,4534,4535],
[4612,4613,4614,4615],
[4631,4621,4611,4612],
[4622,4623,4624,4625],
[4631,4621,4622,4623],
[4632,4633,4634,4635],
[4712,4713,4714,4715],
[4731,4721,4711,4712],
[4722,4723,4724,4725],
[4731,4721,4722,4723],
[4732,4733,4734,4735],
[4812,4813,4814,4815],
[4831,4821,4811,4812],
[4822,4823,4824,4825],
[4831,4821,4822,4823],
[4832,4833,4834,4835],
[4912,4913,4914,4915],
[3211,3221,3231,3232],
[3223,3224,3225,3226],
[3222,3221,3231,3232],
[3233,3234,3235,3236],
[3311,3312,3313,3314],
[3311,3321,3331,3332],
[3323,3324,3325,3326],
[3322,3321,3331,3332],
[3333,3334,3335,3336],
[3411,3412,3413,3414],
[3411,3421,3431,3432],
[3423,3424,3425,3426],
[3422,3421,3431,3432],
[3433,3434,3435,3436],
[3511,3512,3513,3514],
[3511,3521,3531,3532],
[3523,3524,3525,3526],
[3522,3521,3531,3532],
[3533,3534,3535,3536],
[3611,3612,3613,3614],
[3611,3621,3631,3632],
[3623,3624,3625,3626],
[3622,3621,3631,3632],
[3633,3634,3635,3636],
[3711,3712,3713,3714],
[3711,3721,3731,3732],
[3723,3724,3725,3726],
[3722,3721,3731,3732],
[3733,3734,3735,3736],
[3811,3812,3813,3814],
[3811,3821,3831,3832],
[3823,3824,3825,3826],
[3822,3821,3831,3832],
[3833,3834,3835,3836],
[3911,3912,3913,3914],
[3911,3921,3931,3932],
[3923,3924,3925,3926],
[3922,3921,3931,3932],
[3933,3934,3935,3936],
[4011,4012,4013,4014],
[4011,4021,4031,4032],
[4023,4024,4025,4026],
[4022,4021,4031,4032],
[4033,4034,4035,4036],
[4111,4112,4113,4114],
[4111,4121,4131,4132],
[4123,4124,4125,4126],
[4122,4121,4131,4132],
[4133,4134,4135,4136],
[4211,4212,4213,4214],
[4211,4221,4231,4232],
[4223,4224,4225,4226],
[4222,4221,4231,4232],
[4233,4234,4235,4236],
[4311,4312,4313,4314],
[4311,4321,4331,4332],
[4323,4324,4325,4326],
[4322,4321,4331,4332],
[4333,4334,4335,4336],
[4411,4412,4413,4414],
[4411,4421,4431,4432],
[4423,4424,4425,4426],
[4422,4421,4431,4432],
[4433,4434,4435,4436],
[4511,4512,4513,4514],
[4511,4521,4531,4532],
[4523,4524,4525,4526],
[4522,4521,4531,4532],
[4533,4534,4535,4536],
[4611,4612,4613,4614],
[4611,4621,4631,4632],
[4623,4624,4625,4626],
[4622,4621,4631,4632],
[4633,4634,4635,4636],
[4711,4712,4713,4714],
[4711,4721,4731,4732],
[4723,4724,4725,4726],
[4722,4721,4731,4732],
[4733,4734,4735,4736],
[4811,4812,4813,4814],
[4811,4821,4831,4832],
[4823,4824,4825,4826],
[4822,4821,4831,4832],
[4833,4834,4835,4836],
[4911,4912,4913,4914],
[3211,3221,3222,3223],
[3224,3225,3226,3227],
[3221,3231,3232,3233],
[3234,3235,3236,3237],
[3321,3311,3312,3313],
[3311,3321,3322,3323],
[3324,3325,3326,3327],
[3321,3331,3332,3333],
[3334,3335,3336,3337],
[3421,3411,3412,3413],
[3411,3421,3422,3423],
[3424,3425,3426,3427],
[3421,3431,3432,3433],
[3434,3435,3436,3437],
[3521,3511,3512,3513],
[3511,3521,3522,3523],
[3524,3525,3526,3527],
[3521,3531,3532,3533],
[3534,3535,3536,3537],
[3621,3611,3612,3613],
[3611,3621,3622,3623],
[3624,3625,3626,3627],
[3621,3631,3632,3633],
[3634,3635,3636,3637],
[3721,3711,3712,3713],
[3711,3721,3722,3723],
[3724,3725,3726,3727],
[3721,3731,3732,3733],
[3734,3735,3736,3737],
[3821,3811,3812,3813],
[3811,3821,3822,3823],
[3824,3825,3826,3827],
[3821,3831,3832,3833],
[3834,3835,3836,3837],
[3921,3911,3912,3913],
[3911,3921,3922,3923],
[3924,3925,3926,3927],
[3921,3931,3932,3933],
[3934,3935,3936,3937],
[4021,4011,4012,4013],
[4011,4021,4022,4023],
[4024,4025,4026,4027],
[4021,4031,4032,4033],
[4034,4035,4036,4037],
[4121,4111,4112,4113],
[4111,4121,4122,4123],
[4124,4125,4126,4127],
[4121,4131,4132,4133],
[4134,4135,4136,4137],
[4221,4211,4212,4213],
[4211,4221,4222,4223],
[4224,4225,4226,4227],
[4221,4231,4232,4233],
[4234,4235,4236,4237],
[4321,4311,4312,4313],
[4311,4321,4322,4323],
[4324,4325,4326,4327],
[4321,4331,4332,4333],
[4334,4335,4336,4337],
[4421,4411,4412,4413],
[4411,4421,4422,4423],
[4424,4425,4426,4427],
[4421,4431,4432,4433],
[4434,4435,4436,4437],
[4521,4511,4512,4513],
[4511,4521,4522,4523],
[4524,4525,4526,4527],
[4521,4531,4532,4533],
[4534,4535,4536,4537],
[4621,4611,4612,4613],
[4611,4621,4622,4623],
[4624,4625,4626,4627],
[4621,4631,4632,4633],
[4634,4635,4636,4637],
[4721,4711,4712,4713],
[4711,4721,4722,4723],
[4724,4725,4726,4727],
[4721,4731,4732,4733],
[4734,4735,4736,4737],
[4821,4811,4812,4813],
[4811,4821,4822,4823],
[4824,4825,4826,4827],
[4821,4831,4832,4833],
[4834,4835,4836,4837],
[4921,4911,4912,4913],
```

[4922,4921,4911,4912], [4931,4921,4911,4912], [4911,4921,4931,4932], [4911,4921,4922,4923],
[4921,4922,4923,4924], [4922,4923,4924,4925], [4923,4924,4925,4926], [4924,4925,4926,4927],
[4925,4926,4927,4928], [4931,4921,4922,4923], [4922,4921,4931,4932], [4921,4931,4932,4933],
[4931,4932,4933,4934], [4932,4933,4934,4935], [4933,4934,4935,4936], [4934,4935,4936,4937],
[5013,5014,5015,5016], [5012,5013,5014,5015], [5011,5012,5013,5014], [5021,5011,5012,5013],
[5022,5021,5011,5012], [5031,5021,5011,5012], [5011,5021,5031,5032], [5011,5021,5022,5023],
[5021,5022,5023,5024], [5022,5023,5024,5025], [5023,5024,5025,5026], [5024,5025,5026,5027],
[5025,5026,5027,5028], [5031,5021,5022,5023], [5022,5021,5031,5032], [5021,5031,5032,5033],
[5031,5032,5033,5034], [5032,5033,5034,5035], [5033,5034,5035,5036], [5034,5035,5036,5037],
[5113,5114,5115,5116], [5112,5113,5114,5115], [5111,5112,5113,5114], [5121,5111,5112,5113],
[5122,5121,5111,5112], [5131,5121,5111,5112], [5111,5121,5131,5132], [5111,5121,5122,5123],
[5121,5122,5123,5124], [5122,5123,5124,5125], [5123,5124,5125,5126], [5124,5125,5126,5127],
[5125,5126,5127,5128], [5131,5121,5122,5123], [5122,5121,5131,5132], [5121,5131,5132,5133],
[5131,5132,5133,5134], [5132,5133,5134,5135], [5133,5134,5135,5136], [5134,5135,5136,5137],
[5213,5214,5215,5216], [5212,5213,5214,5215], [5211,5212,5213,5214], [5221,5211,5212,5213],
[5222,5221,5211,5212], [5231,5221,5211,5212], [5211,5221,5231,5232], [5211,5221,5222,5223],
[5221,5222,5223,5224], [5222,5223,5224,5225], [5223,5224,5225,5226], [5224,5225,5226,5227],
[5225,5226,5227,5228], [5231,5221,5222,5223], [5222,5221,5231,5232], [5221,5231,5232,5233],
[5231,5232,5233,5234], [5232,5233,5234,5235], [5233,5234,5235,5236], [5234,5235,5236,5237],
[5313,5314,5315,5316], [5312,5313,5314,5315], [5311,5312,5313,5314], [5321,5311,5312,5313],
[5322,5321,5311,5312], [5331,5321,5311,5312], [5311,5321,5331,5332], [5311,5321,5322,5323],
[5321,5322,5323,5324], [5322,5323,5324,5325], [5323,5324,5325,5326], [5324,5325,5326,5327],
[5325,5326,5327,5328], [5331,5321,5322,5323], [5322,5321,5331,5332], [5321,5331,5332,5333],
[5331,5332,5333,5334], [5332,5333,5334,5335], [5333,5334,5335,5336], [5334,5335,5336,5337],
[5413,5414,5415,5416], [5412,5413,5414,5415], [5411,5412,5413,5414], [5421,5411,5412,5413],
[5422,5421,5411,5412], [5431,5421,5411,5412], [5411,5421,5431,5432], [5411,5421,5422,5423],
[5421,5422,5423,5424], [5422,5423,5424,5425], [5423,5424,5425,5426], [5424,5425,5426,5427],
[5425,5426,5427,5428], [5431,5421,5422,5423], [5422,5421,5431,5432], [5421,5431,5432,5433],
[5431,5432,5433,5434], [5432,5433,5434,5435], [5433,5434,5435,5436], [5434,5435,5436,5437],
[5513,5514,5515,5516], [5512,5513,5514,5515], [5511,5512,5513,5514], [5521,5511,5512,5513],
[5522,5521,5511,5512], [5531,5521,5511,5512], [5511,5521,5531,5532], [5511,5521,5522,5523],
[5521,5522,5523,5524], [5522,5523,5524,5525], [5523,5524,5525,5526], [5524,5525,5526,5527],
[5525,5526,5527,5528], [5531,5521,5522,5523], [5522,5521,5531,5532], [5521,5531,5532,5533],
[5531,5532,5533,5534], [5532,5533,5534,5535], [5533,5534,5535,5536], [5534,5535,5536,5537],
[5613,5614,5615,5616], [5612,5613,5614,5615], [5611,5612,5613,5614], [5621,5611,5612,5613],
[5622,5621,5611,5612], [5631,5621,5611,5612], [5611,5621,5631,5632], [5611,5621,5622,5623],
[5621,5622,5623,5624], [5622,5623,5624,5625], [5623,5624,5625,5626], [5624,5625,5626,5627],
[5625,5626,5627,5628], [5631,5621,5622,5623], [5622,5621,5631,5632], [5621,5631,5632,5633],
[5631,5632,5633,5634], [5632,5633,5634,5635], [5633,5634,5635,5636], [5634,5635,5636,5637],
[5713,5714,5715,5716], [5712,5713,5714,5715], [5711,5712,5713,5714], [5721,5711,5712,5713],
[5722,5721,5711,5712], [5731,5721,5711,5712], [5711,5721,5731,5732], [5711,5721,5722,5723],
[5721,5722,5723,5724], [5722,5723,5724,5725], [5723,5724,5725,5726], [5724,5725,5726,5727],
[5725,5726,5727,5728], [5731,5721,5722,5723], [5722,5721,5731,5732], [5721,5731,5732,5733],
[5731,5732,5733,5734], [5732,5733,5734,5735], [5733,5734,5735,5736], [5734,5735,5736,5737],
[5813,5814,5815,5816], [5812,5813,5814,5815], [5811,5812,5813,5814], [5821,5811,5812,5813],
[5822,5821,5811,5812], [5831,5821,5811,5812], [5811,5821,5831,5832], [5811,5821,5822,5823],
[5821,5822,5823,5824], [5822,5823,5824,5825], [5823,5824,5825,5826], [5824,5825,5826,5827],
[5825,5826,5827,5828], [5831,5821,5822,5823], [5822,5821,5831,5832], [5821,5831,5832,5833],
[5831,5832,5833,5834], [5832,5833,5834,5835], [5833,5834,5835,5836], [5834,5835,5836,5837],
[5913,5914,5915,5916], [5912,5913,5914,5915], [5911,5912,5913,5914], [5921,5911,5912,5913],
[5922,5921,5911,5912], [5931,5921,5911,5912], [5911,5921,5931,5932], [5911,5921,5922,5923],
[5921,5922,5923,5924], [5922,5923,5924,5925], [5923,5924,5925,5926], [5924,5925,5926,5927],
[5925,5926,5927,5928], [5931,5921,5922,5923], [5922,5921,5931,5932], [5921,5931,5932,5933],
[5931,5932,5933,5934], [5932,5933,5934,5935], [5933,5934,5935,5936], [5934,5935,5936,5937],
[6013,6014,6015,6016], [6012,6013,6014,6015], [6011,6012,6013,6014], [6021,6011,6012,6013],
[6022,6021,6011,6012], [6031,6021,6011,6012], [6011,6021,6031,6032], [6011,6021,6022,6023],
[6021,6022,6023,6024], [6022,6023,6024,6025], [6023,6024,6025,6026], [6024,6025,6026,6027],
[6025,6026,6027,6028], [6031,6021,6022,6023], [6022,6021,6031,6032], [6021,6031,6032,6033],
[6031,6032,6033,6034], [6032,6033,6034,6035], [6033,6034,6035,6036], [6034,6035,6036,6037],
[6113,6114,6115,6116], [6112,6113,6114,6115], [6111,6112,6113,6114], [6121,6111,6112,6113],
[6122,6121,6111,6112], [6131,6121,6111,6112], [6111,6121,6131,6132], [6111,6121,6122,6123],
[6121,6122,6123,6124], [6122,6123,6124,6125], [6123,6124,6125,6126], [6124,6125,6126,6127],
[6125,6126,6127,6128], [6131,6121,6122,6123], [6122,6121,6131,6132], [6121,6131,6132,6133],
[6131,6132,6133,6134], [6132,6133,6134,6135], [6133,6134,6135,6136], [6134,6135,6136,6137],
[6213,6214,6215,6216], [6212,6213,6214,6215], [6211,6212,6213,6214], [6221,6211,6212,6213],
[6222,6221,6211,6212], [6231,6221,6211,6212], [6211,6221,6231,6232], [6211,6221,6222,6223],
[6221,6222,6223,6224], [6222,6223,6224,6225], [6223,6224,6225,6226], [6224,6225,6226,6227],
[6225,6226,6227,6228], [6231,6221,6222,6223], [6222,6221,6231,6232], [6221,6231,6232,6233],
[6231,6232,6233,6234], [6232,6233,6234,6235], [6233,6234,6235,6236], [6234,6235,6236,6237],
[6313,6314,6315,6316], [6312,6313,6314,6315], [6311,6312,6313,6314], [6321,6311,6312,6313],
[6322,6321,6311,6312], [6331,6321,6311,6312], [6311,6321,6331,6332], [6311,6321,6322,6323],
[6321,6322,6323,6324], [6322,6323,6324,6325], [6323,6324,6325,6326], [6324,6325,6326,6327],
[6325,6326,6327,6328], [6331,6321,6322,6323], [6322,6321,6331,6332], [6321,6331,6332,6333],
[6331,6332,6333,6334], [6332,6333,6334,6335], [6333,6334,6335,6336], [6334,6335,6336,6337],
[6413,6414,6415,6416], [6412,6413,6414,6415], [6411,6412,6413,6414], [6421,6411,6412,6413],
[6422,6421,6411,6412], [6431,6421,6411,6412], [6411,6421,6431,6432], [6411,6421,6422,6423],
[6421,6422,6423,6424], [6422,6423,6424,6425], [6423,6424,6425,6426], [6424,6425,6426,6427],
[6425,6426,6427,6428], [6431,6421,6422,6423], [6422,6421,6431,6432], [6421,6431,6432,6433],
[6431,6432,6433,6434], [6432,6433,6434,6435], [6433,6434,6435,6436], [6434,6435,6436,6437],
[6513,6514,6515,6516], [6512,6513,6514,6515], [6511,6512,6513,6514], [6521,6511,6512,6513],
[6522,6521,6511,6512], [6531,6521,6511,6512], [6511,6521,6531,6532], [6511,6521,6522,6523],
[6521,6522,6523,6524], [6522,6523,6524,6525], [6523,6524,6525,6526], [6524,6525,6526,6527],
[6525,6526,6527,6528], [6531,6521,6522,6523], [6522,6521,6531,6532], [6521,6531,6532,6533],
[6531,6532,6533,6534], [6532,6533,6534,6535], [6533,6534,6535,6536], [6534,6535,6536,6537],
[6613,6614,6615,6616], [6612,6613,6614,6615], [6611,6612,6613,6614], [6621,6611,6612,6613],

[6622,6621,6611,6612],
[6621,6622,6623,6624],
[6625,6626,6627,6628],
[6631,6632,6633,6634],
[6713,6714,6715,6716],
[6722,6721,6711,6712],
[6721,6722,6723,6724],
[6725,6726,6727,6728],
[6731,6732,6733,6734],
[6813,6814,6815,6816],
[6822,6821,6811,6812],
[6821,6822,6823,6824],
[6825,6826,6827,6828],
[6831,6832,6833,6834],
[6913,6914,6915,6916],
[6922,6921,6911,6912],
[6921,6922,6923,6924],
[6925,6926,6927,6928],
[6931,6932,6933,6934],
[7013,7014,7015,7016],
[7022,7021,7011,7012],
[7021,7022,7023,7024],
[7025,7026,7027,7028],
[7031,7032,7033,7034],
[7113,7114,7115,7116],
[7122,7121,7111,7112],
[7121,7122,7123,7124],
[7125,7126,7127,7128],
[7131,7132,7133,7134],
[7213,7214,7215,7216],
[7222,7221,7211,7212],
[7221,7222,7223,7224],
[7225,7226,7227,7228],
[7231,7232,7233,7234],
[7313,7314,7315,7316],
[7322,7321,7311,7312],
[7321,7322,7323,7324],
[7325,7326,7327,7328],
[7331,7332,7333,7334],
[7413,7414,7415,7416],
[7422,7421,7411,7412],
[7421,7422,7423,7424],
[7425,7426,7427,7428],
[7431,7432,7433,7434],
[7513,7514,7515,7516],
[7522,7521,7511,7512],
[7521,7522,7523,7524],
[7525,7526,7527,7528],
[7531,7532,7533,7534],
[7613,7614,7615,7616],
[7622,7621,7611,7612],
[7621,7622,7623,7624],
[7625,7626,7627,7628],
[7631,7632,7633,7634],
[7713,7714,7715,7716],
[7722,7721,7711,7712],
[7721,7722,7723,7724],
[7725,7726,7727,7728],
[7731,7732,7733,7734],
[7813,7814,7815,7816],
[7822,7821,7811,7812],
[7821,7822,7823,7824],
[7825,7826,7827,7828],
[7831,7832,7833,7834],
[7913,7914,7915,7916],
[7922,7921,7911,7912],
[7921,7922,7923,7924],
[7925,7926,7927,7928],
[7931,7932,7933,7934],
[8013,8014,8015,8016],
[8022,8021,8011,8012],
[8021,8022,8023,8024],
[8025,8026,8027,8028],
[8031,8032,8033,8034],
[8113,8114,8115,8116],
[8122,8121,8111,8112],
[8121,8122,8123,8124],
[8125,8126,8127,8128],
[8131,8132,8133,8134],
[8213,8214,8215,8216],
[8222,8221,8211,8212],
[8221,8222,8223,8224],
[8225,8226,8227,8228],
[8231,8232,8233,8234],
[8313,8314,8315,8316],
[6631,6621,6611,6612],
[6622,6623,6624,6625],
[6631,6621,6622,6623],
[6632,6633,6634,6635],
[6712,6713,6714,6715],
[6731,6721,6711,6712],
[6722,6723,6724,6725],
[6731,6721,6722,6723],
[6732,6733,6734,6735],
[6812,6813,6814,6815],
[6831,6821,6811,6812],
[6822,6823,6824,6825],
[6831,6821,6822,6823],
[6832,6833,6834,6835],
[6912,6913,6914,6915],
[6931,6921,6911,6912],
[6922,6923,6924,6925],
[6931,6921,6922,6923],
[6932,6933,6934,6935],
[7012,7013,7014,7015],
[7031,7021,7011,7012],
[7022,7023,7024,7025],
[7031,7021,7022,7023],
[7032,7033,7034,7035],
[7112,7113,7114,7115],
[7131,7121,7111,7112],
[7122,7123,7124,7125],
[7131,7121,7122,7123],
[7132,7133,7134,7135],
[7212,7213,7214,7215],
[7231,7221,7211,7212],
[7222,7223,7224,7225],
[7231,7221,7222,7223],
[7232,7233,7234,7235],
[7312,7313,7314,7315],
[7331,7321,7311,7312],
[7322,7323,7324,7325],
[7331,7321,7322,7323],
[7332,7333,7334,7335],
[7412,7413,7414,7415],
[7431,7421,7411,7412],
[7422,7423,7424,7425],
[7431,7421,7422,7423],
[7432,7433,7434,7435],
[7512,7513,7514,7515],
[7531,7521,7511,7512],
[7522,7523,7524,7525],
[7531,7521,7522,7523],
[7532,7533,7534,7535],
[7612,7613,7614,7615],
[7631,7621,7611,7612],
[7622,7623,7624,7625],
[7631,7621,7622,7623],
[7632,7633,7634,7635],
[7712,7713,7714,7715],
[7731,7721,7711,7712],
[7722,7723,7724,7725],
[7731,7721,7722,7723],
[7732,7733,7734,7735],
[7812,7813,7814,7815],
[7831,7821,7811,7812],
[7822,7823,7824,7825],
[7831,7821,7822,7823],
[7832,7833,7834,7835],
[7912,7913,7914,7915],
[7931,7921,7911,7912],
[7922,7923,7924,7925],
[7931,7921,7922,7923],
[7932,7933,7934,7935],
[8012,8013,8014,8015],
[8031,8021,8011,8012],
[8022,8023,8024,8025],
[8031,8021,8022,8023],
[8032,8033,8034,8035],
[8112,8113,8114,8115],
[8131,8121,8111,8112],
[8122,8123,8124,8125],
[8131,8121,8122,8123],
[8132,8133,8134,8135],
[8212,8213,8214,8215],
[8231,8221,8211,8212],
[8222,8223,8224,8225],
[8231,8221,8222,8223],
[8232,8233,8234,8235],
[8312,8313,8314,8315],
[6611,6621,6631,6632],
[6623,6624,6625,6626],
[6622,6621,6631,6632],
[6633,6634,6635,6636],
[6711,6712,6713,6714],
[6711,6721,6731,6732],
[6723,6724,6725,6726],
[6722,6721,6731,6732],
[6733,6734,6735,6736],
[6811,6812,6813,6814],
[6811,6821,6831,6832],
[6823,6824,6825,6826],
[6822,6821,6831,6832],
[6833,6834,6835,6836],
[6911,6912,6913,6914],
[6911,6921,6931,6932],
[6923,6924,6925,6926],
[6922,6921,6931,6932],
[6933,6934,6935,6936],
[7011,7012,7013,7014],
[7011,7021,7031,7032],
[7023,7024,7025,7026],
[7022,7021,7031,7032],
[7033,7034,7035,7036],
[7111,7112,7113,7114],
[7111,7121,7131,7132],
[7123,7124,7125,7126],
[7122,7121,7131,7132],
[7133,7134,7135,7136],
[7211,7212,7213,7214],
[7211,7221,7231,7232],
[7223,7224,7225,7226],
[7222,7221,7231,7232],
[7233,7234,7235,7236],
[7311,7312,7313,7314],
[7311,7321,7331,7332],
[7323,7324,7325,7326],
[7322,7321,7331,7332],
[7333,7334,7335,7336],
[7411,7412,7413,7414],
[7411,7421,7431,7432],
[7423,7424,7425,7426],
[7422,7421,7431,7432],
[7433,7434,7435,7436],
[7511,7512,7513,7514],
[7511,7521,7531,7532],
[7523,7524,7525,7526],
[7522,7521,7531,7532],
[7533,7534,7535,7536],
[7611,7612,7613,7614],
[7611,7621,7631,7632],
[7623,7624,7625,7626],
[7622,7621,7631,7632],
[7633,7634,7635,7636],
[7711,7712,7713,7714],
[7711,7721,7731,7732],
[7723,7724,7725,7726],
[7722,7721,7731,7732],
[7733,7734,7735,7736],
[7811,7812,7813,7814],
[7811,7821,7831,7832],
[7823,7824,7825,7826],
[7822,7821,7831,7832],
[7833,7834,7835,7836],
[7911,7912,7913,7914],
[7911,7921,7931,7932],
[7923,7924,7925,7926],
[7922,7921,7931,7932],
[7933,7934,7935,7936],
[8011,8012,8013,8014],
[8011,8021,8031,8032],
[8023,8024,8025,8026],
[8022,8021,8031,8032],
[8033,8034,8035,8036],
[8111,8112,8113,8114],
[8111,8121,8131,8132],
[8123,8124,8125,8126],
[8122,8121,8131,8132],
[8133,8134,8135,8136],
[8211,8212,8213,8214],
[8211,8221,8231,8232],
[8223,8224,8225,8226],
[8222,8221,8231,8232],
[8233,8234,8235,8236],
[8311,8312,8313,8314],
[6611,6621,6622,6623],
[6624,6625,6626,6627],
[6621,6631,6632,6633],
[6634,6635,6636,6637],
[6721,6711,6712,6713],
[6711,6721,6722,6723],
[6724,6725,6726,6727],
[6721,6731,6732,6733],
[6734,6735,6736,6737],
[6821,6811,6812,6813],
[6811,6821,6822,6823],
[6824,6825,6826,6827],
[6821,6831,6832,6833],
[6834,6835,6836,6837],
[6921,6911,6912,6913],
[6911,6921,6922,6923],
[6924,6925,6926,6927],
[6921,6931,6932,6933],
[6934,6935,6936,6937],
[7021,7011,7012,7013],
[7011,7021,7022,7023],
[7024,7025,7026,7027],
[7021,7031,7032,7033],
[7034,7035,7036,7037],
[7121,7111,7112,7113],
[7111,7121,7122,7123],
[7124,7125,7126,7127],
[7121,7131,7132,7133],
[7134,7135,7136,7137],
[7221,7211,7212,7213],
[7211,7221,7222,7223],
[7224,7225,7226,7227],
[7221,7231,7232,7233],
[7234,7235,7236,7237],
[7321,7311,7312,7313],
[7311,7321,7322,7323],
[7324,7325,7326,7327],
[7321,7331,7332,7333],
[7334,7335,7336,7337],
[7421,7411,7412,7413],
[7411,7421,7422,7423],
[7424,7425,7426,7427],
[7421,7431,7432,7433],
[7434,7435,7436,7437],
[7521,7511,7512,7513],
[7511,7521,7522,7523],
[7524,7525,7526,7527],
[7521,7531,7532,7533],
[7534,7535,7536,7537],
[7621,7611,7612,7613],
[7611,7621,7622,7623],
[7624,7625,7626,7627],
[7621,7631,7632,7633],
[7634,7635,7636,7637],
[7721,7711,7712,7713],
[7711,7721,7722,7723],
[7724,7725,7726,7727],
[7721,7731,7732,7733],
[7734,7735,7736,7737],
[7821,7811,7812,7813],
[7811,7821,7822,7823],
[7824,7825,7826,7827],
[7821,7831,7832,7833],
[7834,7835,7836,7837],
[7921,7911,7912,7913],
[7911,7921,7922,7923],
[7924,7925,7926,7927],
[7921,7931,7932,7933],
[7934,7935,7936,7937],
[8021,8011,8012,8013],
[8011,8021,8022,8023],
[8024,8025,8026,8027],
[8021,8031,8032,8033],
[8034,8035,8036,8037],
[8121,8111,8112,8113],
[8111,8121,8122,8123],
[8124,8125,8126,8127],
[8121,8131,8132,8133],
[8134,8135,8136,8137],
[8221,8211,8212,8213],
[8211,8221,8222,8223],
[8224,8225,8226,8227],
[8221,8231,8232,8233],
[8234,8235,8236,8237],
[8321,8311,8312,8313],

[8322,8321,8311,8312],
[8321,8322,8323,8324],
[8325,8326,8327,8328],
[8331,8332,8333,8334],
[8413,8414,8415,8416],
[8422,8421,8411,8412],
[8421,8422,8423,8424],
[8425,8426,8427,8428],
[8431,8432,8433,8434],
[8513,8514,8515,8516],
[8522,8521,8511,8512],
[8521,8522,8523,8524],
[8525,8526,8527,8528],
[8531,8532,8533,8534],
[8613,8614,8615,8616],
[8622,8621,8611,8612],
[8621,8622,8623,8624],
[8625,8626,8627,8628],
[8631,8632,8633,8634],
[8713,8714,8715,8716],
[8722,8721,8711,8712],
[8721,8722,8723,8724],
[8725,8726,8727,8728],
[8731,8732,8733,8734],
[8813,8814,8815,8816],
[8822,8821,8811,8812],
[8821,8822,8823,8824],
[8825,8826,8827,8828],
[8831,8832,8833,8834],
[8913,8914,8915,8916],
[8922,8921,8911,8912],
[8921,8922,8923,8924],
[8925,8926,8927,8928],
[8931,8932,8933,8934],
[9013,9014,9015,9016],
[9022,9021,9011,9012],
[9021,9022,9023,9024],
[9025,9026,9027,9028],
[9031,9032,9033,9034],
[9113,9114,9115,9116],
[9122,9121,9111,9112],
[9121,9122,9123,9124],
[9125,9126,9127,9128],
[9131,9132,9133,9134],
[9213,9214,9215,9216],
[9222,9221,9211,9212],
[9221,9222,9223,9224],
[9225,9226,9227,9228],
[9231,9232,9233,9234],
[9313,9314,9315,9316],
[9322,9321,9311,9312],
[9321,9322,9323,9324],
[9325,9326,9327,9328],
[9331,9332,9333,9334],
[9413,9414,9415,9416],
[9422,9421,9411,9412],
[9421,9422,9423,9424],
[9425,9426,9427,9428],
[9431,9432,9433,9434],
[9513,9514,9515,9516],
[9522,9521,9511,9512],
[9521,9522,9523,9524],
[9525,9526,9527,9528],
[9531,9532,9533,9534],
[9613,9614,9615,9616],
[9622,9621,9611,9612],
[9621,9622,9623,9624],
[9625,9626,9627,9628],
[9631,9632,9633,9634],
[9713,9714,9715,9716],
[9722,9721,9711,9712],
[9721,9722,9723,9724],
[9725,9726,9727,9728],
[9731,9732,9733,9734],
[9813,9814,9815,9816],
[9822,9821,9811,9812],
[9821,9822,9823,9824],
[9825,9826,9827,9828],
[9831,9832,9833,9834],
[9913,9914,9915,9916],
[9922,9921,9911,9912],
[9921,9922,9923,9924],
[9925,9926,9927,9928],
[9931,9932,9933,9934],
[10013,10014,10015,10016],
[8331,8321,8311,8312],
[8322,8323,8324,8325],
[8331,8321,8322,8323],
[8332,8333,8334,8335],
[8412,8413,8414,8415],
[8431,8421,8411,8412],
[8422,8423,8424,8425],
[8431,8421,8422,8423],
[8432,8433,8434,8435],
[8512,8513,8514,8515],
[8531,8521,8511,8512],
[8522,8523,8524,8525],
[8531,8521,8522,8523],
[8532,8533,8534,8535],
[8612,8613,8614,8615],
[8631,8621,8611,8612],
[8622,8623,8624,8625],
[8631,8621,8622,8623],
[8632,8633,8634,8635],
[8712,8713,8714,8715],
[8731,8721,8711,8712],
[8722,8723,8724,8725],
[8731,8721,8722,8723],
[8732,8733,8734,8735],
[8812,8813,8814,8815],
[8831,8821,8811,8812],
[8822,8823,8824,8825],
[8831,8821,8822,8823],
[8832,8833,8834,8835],
[8912,8913,8914,8915],
[8931,8921,8911,8912],
[8922,8923,8924,8925],
[8931,8921,8922,8923],
[8932,8933,8934,8935],
[9012,9013,9014,9015],
[9031,9021,9011,9012],
[9022,9023,9024,9025],
[9031,9021,9022,9023],
[9032,9033,9034,9035],
[9112,9113,9114,9115],
[9131,9121,9111,9112],
[9122,9123,9124,9125],
[9131,9121,9122,9123],
[9132,9133,9134,9135],
[9212,9213,9214,9215],
[9231,9221,9211,9212],
[9222,9223,9224,9225],
[9231,9221,9222,9223],
[9232,9233,9234,9235],
[9312,9313,9314,9315],
[9331,9321,9311,9312],
[9322,9323,9324,9325],
[9331,9321,9322,9323],
[9332,9333,9334,9335],
[9412,9413,9414,9415],
[9431,9421,9411,9412],
[9422,9423,9424,9425],
[9431,9421,9422,9423],
[9432,9433,9434,9435],
[9512,9513,9514,9515],
[9531,9521,9511,9512],
[9522,9523,9524,9525],
[9531,9521,9522,9523],
[9532,9533,9534,9535],
[9612,9613,9614,9615],
[9631,9621,9611,9612],
[9622,9623,9624,9625],
[9631,9621,9622,9623],
[9632,9633,9634,9635],
[9712,9713,9714,9715],
[9731,9721,9711,9712],
[9722,9723,9724,9725],
[9731,9721,9722,9723],
[9732,9733,9734,9735],
[9812,9813,9814,9815],
[9831,9821,9811,9812],
[9822,9823,9824,9825],
[9831,9821,9822,9823],
[9832,9833,9834,9835],
[9912,9913,9914,9915],
[9931,9921,9911,9912],
[9922,9923,9924,9925],
[9931,9921,9922,9923],
[9932,9933,9934,9935],
[10012,10013,10014,10015],
[8311,8321,8331,8332],
[8323,8324,8325,8326],
[8322,8321,8331,8332],
[8333,8334,8335,8336],
[8411,8412,8413,8414],
[8411,8421,8431,8432],
[8423,8424,8425,8426],
[8422,8421,8431,8432],
[8433,8434,8435,8436],
[8511,8512,8513,8514],
[8511,8521,8531,8532],
[8523,8524,8525,8526],
[8522,8521,8531,8532],
[8533,8534,8535,8536],
[8611,8612,8613,8614],
[8611,8621,8631,8632],
[8623,8624,8625,8626],
[8622,8621,8631,8632],
[8633,8634,8635,8636],
[8711,8712,8713,8714],
[8711,8721,8731,8732],
[8723,8724,8725,8726],
[8722,8721,8731,8732],
[8733,8734,8735,8736],
[8811,8812,8813,8814],
[8811,8821,8831,8832],
[8823,8824,8825,8826],
[8822,8821,8831,8832],
[8833,8834,8835,8836],
[8911,8912,8913,8914],
[8911,8921,8931,8932],
[8923,8924,8925,8926],
[8922,8921,8931,8932],
[8933,8934,8935,8936],
[9011,9012,9013,9014],
[9011,9021,9031,9032],
[9023,9024,9025,9026],
[9022,9021,9031,9032],
[9033,9034,9035,9036],
[9111,9112,9113,9114],
[9111,9121,9131,9132],
[9123,9124,9125,9126],
[9122,9121,9131,9132],
[9133,9134,9135,9136],
[9211,9212,9213,9214],
[9211,9221,9231,9232],
[9223,9224,9225,9226],
[9222,9221,9231,9232],
[9233,9234,9235,9236],
[9311,9312,9313,9314],
[9311,9321,9331,9332],
[9323,9324,9325,9326],
[9322,9321,9331,9332],
[9333,9334,9335,9336],
[9411,9412,9413,9414],
[9411,9421,9431,9432],
[9423,9424,9425,9426],
[9422,9421,9431,9432],
[9433,9434,9435,9436],
[9511,9512,9513,9514],
[9511,9521,9531,9532],
[9523,9524,9525,9526],
[9522,9521,9531,9532],
[9533,9534,9535,9536],
[9611,9612,9613,9614],
[9611,9621,9631,9632],
[9623,9624,9625,9626],
[9622,9621,9631,9632],
[9633,9634,9635,9636],
[9711,9712,9713,9714],
[9711,9721,9731,9732],
[9723,9724,9725,9726],
[9722,9721,9731,9732],
[9733,9734,9735,9736],
[9811,9812,9813,9814],
[9811,9821,9831,9832],
[9823,9824,9825,9826],
[9822,9821,9831,9832],
[9833,9834,9835,9836],
[9911,9912,9913,9914],
[9911,9921,9931,9932],
[9923,9924,9925,9926],
[9922,9921,9931,9932],
[9933,9934,9935,9936],
[10011,10012,10013,10014],
[8311,8321,8322,8323],
[8324,8325,8326,8327],
[8321,8331,8332,8333],
[8334,8335,8336,8337],
[8421,8411,8412,8413],
[8411,8421,8422,8423],
[8424,8425,8426,8427],
[8421,8431,8432,8433],
[8434,8435,8436,8437],
[8521,8511,8512,8513],
[8511,8521,8522,8523],
[8524,8525,8526,8527],
[8521,8531,8532,8533],
[8534,8535,8536,8537],
[8621,8611,8612,8613],
[8611,8621,8622,8623],
[8624,8625,8626,8627],
[8621,8631,8632,8633],
[8634,8635,8636,8637],
[8721,8711,8712,8713],
[8711,8721,8722,8723],
[8724,8725,8726,8727],
[8721,8731,8732,8733],
[8734,8735,8736,8737],
[8821,8811,8812,8813],
[8811,8821,8822,8823],
[8824,8825,8826,8827],
[8821,8831,8832,8833],
[8834,8835,8836,8837],
[8921,8911,8912,8913],
[8911,8921,8922,8923],
[8924,8925,8926,8927],
[8921,8931,8932,8933],
[8934,8935,8936,8937],
[9021,9011,9012,9013],
[9011,9021,9022,9023],
[9024,9025,9026,9027],
[9021,9031,9032,9033],
[9034,9035,9036,9037],
[9121,9111,9112,9113],
[9111,9121,9122,9123],
[9124,9125,9126,9127],
[9121,9131,9132,9133],
[9134,9135,9136,9137],
[9221,9211,9212,9213],
[9211,9221,9222,9223],
[9224,9225,9226,9227],
[9221,9231,9232,9233],
[9234,9235,9236,9237],
[9321,9311,9312,9313],
[9311,9321,9322,9323],
[9324,9325,9326,9327],
[9321,9331,9332,9333],
[9334,9335,9336,9337],
[9421,9411,9412,9413],
[9411,9421,9422,9423],
[9424,9425,9426,9427],
[9421,9431,9432,9433],
[9434,9435,9436,9437],
[9521,9511,9512,9513],
[9511,9521,9522,9523],
[9524,9525,9526,9527],
[9521,9531,9532,9533],
[9534,9535,9536,9537],
[9621,9611,9612,9613],
[9611,9621,9622,9623],
[9624,9625,9626,9627],
[9621,9631,9632,9633],
[9634,9635,9636,9637],
[9721,9711,9712,9713],
[9711,9721,9722,9723],
[9724,9725,9726,9727],
[9721,9731,9732,9733],
[9734,9735,9736,9737],
[9821,9811,9812,9813],
[9811,9821,9822,9823],
[9824,9825,9826,9827],
[9821,9831,9832,9833],
[9834,9835,9836,9837],
[9921,9911,9912,9913],
[9911,9921,9922,9923],
[9924,9925,9926,9927],
[9921,9931,9932,9933],
[9934,9935,9936,9937],

```
        [10021,10011,10012,10013],  [10022,10021,10011,10012],  [10031,10021,10011,10012],
        [10011,10021,10031,10032],  [10011,10021,10022,10023],  [10021,10022,10023,10024],
        [10022,10023,10024,10025],  [10023,10024,10025,10026],  [10024,10025,10026,10027],
        [10025,10026,10027,10028],  [10031,10021,10022,10023],  [10022,10021,10031,10032],
        [10021,10031,10032,10033],  [10031,10032,10033,10034],  [10032,10033,10034,10035],
        [10033,10034,10035,10036],  [10034,10035,10036,10037]   ])


dihedral_combination_indices = np.array([

[0,20,40,60,80,100,120,140,160,180,200,220,240,260,280,300,320,340,360,380,400,420,440,460,480,500,520,540,560,580,60
0,620,640,660,680,700,720,740,760,780,800,820,840,860,880,900,920,940,960,980,1000,1020,1040,1060,1080,1100,1120,1140
,1160,1180,1200,1220,1240,1260,1280,1300,1320,1340,1360,1380,1400,1420,1440,1460,1480,1500,1520,1540,1560,1580,1600,1
620,1640,1660,1680,1700,1720,1740,1760,1780,1800,1820,1840,1860,1880,1900,1920,1940,1960,1980],

[1,21,41,61,81,101,121,141,161,181,201,221,241,261,281,301,321,341,361,381,401,421,441,461,481,501,521,541,561,581,60
1,621,641,661,681,701,721,741,761,781,801,821,841,861,881,901,921,941,961,981,1001,1021,1041,1061,1081,1101,1121,1141
,1161,1181,1201,1221,1241,1261,1281,1301,1321,1341,1361,1381,1401,1421,1441,1461,1481,1501,1521,1541,1561,1581,1601,1
621,1641,1661,1681,1701,1721,1741,1761,1781,1801,1821,1841,1861,1881,1901,1921,1941,1961,1981],

[2,22,42,62,82,102,122,142,162,182,202,222,242,262,282,302,322,342,362,382,402,422,442,462,482,502,522,542,562,582,60
2,622,642,662,682,702,722,742,762,782,802,822,842,862,882,902,922,942,962,982,1002,1022,1042,1062,1082,1102,1122,1142
,1162,1182,1202,1222,1242,1262,1282,1302,1322,1342,1362,1382,1402,1422,1442,1462,1482,1502,1522,1542,1562,1582,1602,1
622,1642,1662,1682,1702,1722,1742,1762,1782,1802,1822,1842,1862,1882,1902,1922,1942,1962,1982],

[3,23,43,63,83,103,123,143,163,183,203,223,243,263,283,303,323,343,363,383,403,423,443,463,483,503,523,543,563,583,60
3,623,643,663,683,703,723,743,763,783,803,823,843,863,883,903,923,943,963,983,1003,1023,1043,1063,1083,1103,1123,1143
,1163,1183,1203,1223,1243,1263,1283,1303,1323,1343,1363,1383,1403,1423,1443,1463,1483,1503,1523,1543,1563,1583,1603,1
623,1643,1663,1683,1703,1723,1743,1763,1783,1803,1823,1843,1863,1883,1903,1923,1943,1963,1983],

[4,24,44,64,84,104,124,144,164,184,204,224,244,264,284,304,324,344,364,384,404,424,444,464,484,504,524,544,564,584,60
4,624,644,664,684,704,724,744,764,784,804,824,844,864,884,904,924,944,964,984,1004,1024,1044,1064,1084,1104,1124,1144
,1164,1184,1204,1224,1244,1264,1284,1304,1324,1344,1364,1384,1404,1424,1444,1464,1484,1504,1524,1544,1564,1584,1604,1
624,1644,1664,1684,1704,1724,1744,1764,1784,1804,1824,1844,1864,1884,1904,1924,1944,1964,1984],

[5,25,45,65,85,105,125,145,165,185,205,225,245,265,285,305,325,345,365,385,405,425,445,465,485,505,525,545,565,585,60
5,625,645,665,685,705,725,745,765,785,805,825,845,865,885,905,925,945,965,985,1005,1025,1045,1065,1085,1105,1125,1145
,1165,1185,1205,1225,1245,1265,1285,1305,1325,1345,1365,1385,1405,1425,1445,1465,1485,1505,1525,1545,1565,1585,1605,1
625,1645,1665,1685,1705,1725,1745,1765,1785,1805,1825,1845,1865,1885,1905,1925,1945,1965,1985],

[6,26,46,66,86,106,126,146,166,186,206,226,246,266,286,306,326,346,366,386,406,426,446,466,486,506,526,546,566,586,60
6,626,646,666,686,706,726,746,766,786,806,826,846,866,886,906,926,946,966,986,1006,1026,1046,1066,1086,1106,1126,1146
,1166,1186,1206,1226,1246,1266,1286,1306,1326,1346,1366,1386,1406,1426,1446,1466,1486,1506,1526,1546,1566,1586,1606,1
626,1646,1666,1686,1706,1726,1746,1766,1786,1806,1826,1846,1866,1886,1906,1926,1946,1966,1986],

[7,27,47,67,87,107,127,147,167,187,207,227,247,267,287,307,327,347,367,387,407,427,447,467,487,507,527,547,567,587,60
7,627,647,667,687,707,727,747,767,787,807,827,847,867,887,907,927,947,967,987,1007,1027,1047,1067,1087,1107,1127,1147
,1167,1187,1207,1227,1247,1267,1287,1307,1327,1347,1367,1387,1407,1427,1447,1467,1487,1507,1527,1547,1567,1587,1607,1
627,1647,1667,1687,1707,1727,1747,1767,1787,1807,1827,1847,1867,1887,1907,1927,1947,1967,1987],

[8,28,48,68,88,108,128,148,168,188,208,228,248,268,288,308,328,348,368,388,408,428,448,468,488,508,528,548,568,588,60
8,628,648,668,688,708,728,748,768,788,808,828,848,868,888,908,928,948,968,988,1008,1028,1048,1068,1088,1108,1128,1148
,1168,1188,1208,1228,1248,1268,1288,1308,1328,1348,1368,1388,1408,1428,1448,1468,1488,1508,1528,1548,1568,1588,1608,1
628,1648,1668,1688,1708,1728,1748,1768,1788,1808,1828,1848,1868,1888,1908,1928,1948,1968,1988],

[9,29,49,69,89,109,129,149,169,189,209,229,249,269,289,309,329,349,369,389,409,429,449,469,489,509,529,549,569,589,60
9,629,649,669,689,709,729,749,769,789,809,829,849,869,889,909,929,949,969,989,1009,1029,1049,1069,1089,1109,1129,1149
,1169,1189,1209,1229,1249,1269,1289,1309,1329,1349,1369,1389,1409,1429,1449,1469,1489,1509,1529,1549,1569,1589,1609,1
629,1649,1669,1689,1709,1729,1749,1769,1789,1809,1829,1849,1869,1889,1909,1929,1949,1969,1989],

[10,30,50,70,90,110,130,150,170,190,210,230,250,270,290,310,330,350,370,390,410,430,450,470,490,510,530,550,570,590,6
10,630,650,670,690,710,730,750,770,790,810,830,850,870,890,910,930,950,970,990,1010,1030,1050,1070,1090,1110,1130,115
0,1170,1190,1210,1230,1250,1270,1290,1310,1330,1350,1370,1390,1410,1430,1450,1470,1490,1510,1530,1550,1570,1590,1610,
1630,1650,1670,1690,1710,1730,1750,1770,1790,1810,1830,1850,1870,1890,1910,1930,1950,1970,1990],

[11,31,51,71,91,111,131,151,171,191,211,231,251,271,291,311,331,351,371,391,411,431,451,471,491,511,531,551,571,591,6
11,631,651,671,691,711,731,751,771,791,811,831,851,871,891,911,931,951,971,991,1011,1031,1051,1071,1091,1111,1131,115
1,1171,1191,1211,1231,1251,1271,1291,1311,1331,1351,1371,1391,1411,1431,1451,1471,1491,1511,1531,1551,1571,1591,1611,
1631,1651,1671,1691,1711,1731,1751,1771,1791,1811,1831,1851,1871,1891,1911,1931,1951,1971,1991],

[12,32,52,72,92,112,132,152,172,192,212,232,252,272,292,312,332,352,372,392,412,432,452,472,492,512,532,552,572,592,6
12,632,652,672,692,712,732,752,772,792,812,832,852,872,892,912,932,952,972,992,1012,1032,1052,1072,1092,1112,1132,115
2,1172,1192,1212,1232,1252,1272,1292,1312,1332,1352,1372,1392,1412,1432,1452,1472,1492,1512,1532,1552,1572,1592,1612,
1632,1652,1672,1692,1712,1732,1752,1772,1792,1812,1832,1852,1872,1892,1912,1932,1952,1972,1992],

[13,33,53,73,93,113,133,153,173,193,213,233,253,273,293,313,333,353,373,393,413,433,453,473,493,513,533,553,573,593,6
13,633,653,673,693,713,733,753,773,793,813,833,853,873,893,913,933,953,973,993,1013,1033,1053,1073,1093,1113,1133,115
3,1173,1193,1213,1233,1253,1273,1293,1313,1333,1353,1373,1393,1413,1433,1453,1473,1493,1513,1533,1553,1573,1593,1613,
1633,1653,1673,1693,1713,1733,1753,1773,1793,1813,1833,1853,1873,1893,1913,1933,1953,1973,1993],

[14,34,54,74,94,114,134,154,174,194,214,234,254,274,294,314,334,354,374,394,414,434,454,474,494,514,534,554,574,594,6
14,634,654,674,694,714,734,754,774,794,814,834,854,874,894,914,934,954,974,994,1014,1034,1054,1074,1094,1114,1134,115
4,1174,1194,1214,1234,1254,1274,1294,1314,1334,1354,1374,1394,1414,1434,1454,1474,1494,1514,1534,1554,1574,1594,1614,
1634,1654,1674,1694,1714,1734,1754,1774,1794,1814,1834,1854,1874,1894,1914,1934,1954,1974,1994],
```

```
[15,35,55,75,95,115,135,155,175,195,215,235,255,275,295,315,335,355,375,395,415,435,455,475,495,515,535,555,575,595,6
15,635,655,675,695,715,735,755,775,795,815,835,855,875,895,915,935,955,975,995,1015,1035,1055,1075,1095,1115,1135,115
5,1175,1195,1215,1235,1255,1275,1295,1315,1335,1355,1375,1395,1415,1435,1455,1475,1495,1515,1535,1555,1575,1595,1615,
1635,1655,1675,1695,1715,1735,1755,1775,1795,1815,1835,1855,1875,1895,1915,1935,1955,1975,1995],

[16,36,56,76,96,116,136,156,176,196,216,236,256,276,296,316,336,356,376,396,416,436,456,476,496,516,536,556,576,596,6
16,636,656,676,696,716,736,756,776,796,816,836,856,876,896,916,936,956,976,996,1016,1036,1056,1076,1096,1116,1136,115
6,1176,1196,1216,1236,1256,1276,1296,1316,1336,1356,1376,1396,1416,1436,1456,1476,1496,1516,1536,1556,1576,1596,1616,
1636,1656,1676,1696,1716,1736,1756,1776,1796,1816,1836,1856,1876,1896,1916,1936,1956,1976,1996],

[17,37,57,77,97,117,137,157,177,197,217,237,257,277,297,317,337,357,377,397,417,437,457,477,497,517,537,557,577,597,6
17,637,657,677,697,717,737,757,777,797,817,837,857,877,897,917,937,957,977,997,1017,1037,1057,1077,1097,1117,1137,115
7,1177,1197,1217,1237,1257,1277,1297,1317,1337,1357,1377,1397,1417,1437,1457,1477,1497,1517,1537,1557,1577,1597,1617,
1637,1657,1677,1697,1717,1737,1757,1777,1797,1817,1837,1857,1877,1897,1917,1937,1957,1977,1997],

[18,38,58,78,98,118,138,158,178,198,218,238,258,278,298,318,338,358,378,398,418,438,458,478,498,518,538,558,578,598,6
18,638,658,678,698,718,738,758,778,798,818,838,858,878,898,918,938,958,978,998,1018,1038,1058,1078,1098,1118,1138,115
8,1178,1198,1218,1238,1258,1278,1298,1318,1338,1358,1378,1398,1418,1438,1458,1478,1498,1518,1538,1558,1578,1598,1618,
1638,1658,1678,1698,1718,1738,1758,1778,1798,1818,1838,1858,1878,1898,1918,1938,1958,1978,1998],

[19,39,59,79,99,119,139,159,179,199,219,239,259,279,299,319,339,359,379,399,419,439,459,479,499,519,539,559,579,599,6
19,639,659,679,699,719,739,759,779,799,819,839,859,879,899,919,939,959,979,999,1019,1039,1059,1079,1099,1119,1139,115
9,1179,1199,1219,1239,1259,1279,1299,1319,1339,1359,1379,1399,1419,1439,1459,1479,1499,1519,1539,1559,1579,1599,1619,
1639,1659,1679,1699,1719,1739,1759,1779,1799,1819,1839,1859,1879,1899,1919,1939,1959,1979,1999] ])

combined_dihedral_names = ['13-14-15-16',     '12-13-14-15',     '11-12-13-14',     '21-11-12-13',     '22-21-11-12',
         '31-21-11-12',     '11-21-31-32',     '11-21-22-23',     '21-22-23-24',     '22-23-24-25',     '23-24-25-26',
         '24-25-26-27',     '25-26-27-28',     '31-21-22-23',     '22-21-31-32',     '21-31-32-33',     '31-32-33-34',
         '32-33-34-35',     '33-34-35-36',     '34-35-36-37' ]



for struct in range(len(structure)):

    bead_ = {}
    bead_cog_ = {}

    bead_distance_  = {}
    combined_bead_distance = {}
    combi_bead_array = {}
    average_dist    = np.zeros(shape=(len(distance_combination_indices),(len(temperatures))))
    stdev_dist      = np.zeros(shape=(len(distance_combination_indices),(len(temperatures))))
    histo_dist      = np.zeros(shape=(len(distance_combination_indices),30,(len(temperatures))),dtype=int)
    histo_temp_bond = {}

    bead_angle_ = {}
    combined_bead_angle = {}
    combi_angle_array = {}
    average_angle    = np.ndarray(shape=(len(angle_combination_indices),(len(temperatures))))
    stdev_angle      = np.ndarray(shape=(len(angle_combination_indices),(len(temperatures))))
    histo_angle      = np.ndarray(shape=(len(angle_combination_indices),26,(len(temperatures))),dtype=int)
    histo_temp_angle = {}

    bead_dihedral_ = {}
    combined_bead_dihedral = {}
    combi_dihedral_array = {}
    average_dihedral    = np.ndarray(shape=(len(dihedral_combination_indices),(len(temperatures))))
    stdev_dihedral      = np.ndarray(shape=(len(dihedral_combination_indices),(len(temperatures))))
    histo_dihedral      = np.ndarray(shape=(len(dihedral_combination_indices),36,(len(temperatures))),dtype=int)
    histo_temp_dihedral = {}


    print(str("Starting ") + str(structure[struct]))

    for temp in range(len(temperatures)):

        print(str("\t") + str("Starting ") + str(structure[struct]) + " @ " + str(temperatures[temp]))

        wdir = str('/path/to/folder/' + TAG + '/' + structure[struct] + '/' + temperatures[temp] + '/')
        traj = md.load_xtc(wdir + 'traj.xtc', top = wdir +'box.pdb')

        coordinates_ = {}

        for i in range(num_atoms): # number of atoms

############################
## COORDINATES EXTRACTION ##
############################

# extract the xyz coordinates of each atom from frame 4000 (number of frames = 1001) - resulting array is of the shape
[frames, atom, xyz]
            # coordinates_[i] = the xyz coordinates for each atom for each frame (3D); results in num_atoms arrays
            coordinates_[i] = (traj.xyz[from_frame:,[i],:])
            # reshape the array from 3D to 2D i.e. remove the redundant atom dimension which is just 1
```

```
            coordinates_[i] = np.reshape(coordinates_[i],(num_frames,3))  # array size is 1001 (num_frames) x 3 (xyz)

        print(str("\t")*2 + str("Coordinates extraction complete"))


##################################################
## CALCULATING CENTRE OF GEOMETRY OF EACH BEAD ##
##################################################


### 4 ATOM BEADS - CREATION OF ARRAYS WITH CALCULATED CENTRE OF GEOMETRY
        # create 3D arrays for each bead - each array is of the shape and size [num_frames, num_atoms, xyz]
        for a in range(len(bead_nums_4)):
            # creates an empty 3D array of shape [num_frames(1001), number of atoms(4), xyz(3)] for each bead listed
in bead_nums_atoms_4
            bead_[bead_nums_4[a]] = np.empty([num_frames,4,3])

            # populate the empty arrays from the coordinates extracted above
            for j in range(num_frames): # number of frames
                for k in range(4): # number of atoms per bead
                    for l in range(3): # x, y and z
                        # used to determine the Python atom index number which is then used to refer to that coordinates
array
                        atnum = bead_nums_atoms_4[a,k]
                        # fill in array with coordinates of the atoms
                        bead_[bead_nums_4[a]][j,k,l] = coordinates_[atnum][j,l]

            # creates empty array which will hold centre of geometry coordinates (xyz) of the bead for num_frames
frames
            bead_cog_[bead_nums_4[a]] = np.empty([num_frames,3])
            for m in range(num_frames):  # number of frames
                for n in range(3): # number of coordinates
                    # calculate centre of geometry
                    bead_cog_[bead_nums_4[a]][m,n] = np.mean(bead_[bead_nums_4[a]][m,:,n])

        print(str("\t")*2 + str("4-atom centre of geometry calculations complete"))


## 3 ATOM BEADS - CREATION OF ARRAYS WITH CALCULATED CENTRE OF GEOMETRY
        for a in range(len(bead_nums_3)):
            # creates an empty 3D array of shape [num_frames(1001), number of atoms(3), xyz(3)] for each bead listed
in bead_nums_4
            bead_[bead_nums_3[a]] = np.empty([num_frames,3,3])

            # populate the empty arrays from the coordinates extracted above
            for j in range(num_frames): # number of frames
                for k in range(3): # number of atoms per bead
                    for l in range(3): # x, y and z
                        # used to determine the atom number which is then used to refer to that coordinates array
                        atnum = bead_nums_atoms_3[a,k]
                        bead_[bead_nums_3[a]][j,k,l] = coordinates_[atnum][j,l]

            # creates empty array which will hold centre of geometry coordinates (xyz) of the bead for num_frames
frames
            bead_cog_[bead_nums_3[a]] = np.empty([num_frames,3])
            for m in range(num_frames):  # number of frames
                for n in range(3): # number of coordinates
                    # calculate centre of geometry
                    bead_cog_[bead_nums_3[a]][m,n] = np.mean(bead_[bead_nums_3[a]][m,:,n])

        print(str("\t")*2 + str("3-atom centre of geometry calculations complete"))


### 2 ATOM BEADS - CREATION OF ARRAYS WITH CALCULATED CENTRE OF GEOMETRY
        for a in range(len(bead_nums_2)):
            # creates an empty 3D array of shape [num_frames(1001), number of atoms(2), xyz(3)] for each bead listed
in bead_nums_4
            bead_[bead_nums_2[a]] = np.empty([num_frames,2,3])

            # populate the empty arrays from the coordinates extracted above
            for j in range(num_frames): # number of frames
                for k in range(2): # number of atoms per bead
                    for l in range(3): # x, y and z
                        # used to determine the atom number which is then used to refer to that coordinates array
                        atnum = bead_nums_atoms_2[a,k]
                        bead_[bead_nums_2[a]][j,k,l] = coordinates_[atnum][j,l]

            # creates empty array which will hold centre of geometry coordinates (xyz) of the bead for num_frames
frames
            bead_cog_[bead_nums_2[a]] = np.empty([num_frames,3])
            for m in range(num_frames):  # number of frames
                for n in range(3): # number of coordinates
                    # calculate centre of geometry
                    bead_cog_[bead_nums_2[a]][m,n] = np.mean(bead_[bead_nums_2[a]][m,:,n])
```

```
        print(str("\t")*2 + str("2-atom centre of geometry calculations complete"))


##############################################
## CALCULATING BEAD DISTANCES BETWEEN CoGs AND STATISTICS ##
##############################################

        for i in range(len(bead_connections_list)): # all bead connections for all molecules
            active_bead_1 = bead_connections_list[i,0]
            active_bead_2 = bead_connections_list[i,1]
            bead_distance_[i] = np.ndarray(shape=(num_frames))
            for j in range(num_frames):
                bead_distance_[i][j] = ((bead_cog_[active_bead_1][j,0] - bead_cog_[active_bead_2][j,0])**2 +
                                        (bead_cog_[active_bead_1][j,1] - bead_cog_[active_bead_2][j,1])**2 +
                                        (bead_cog_[active_bead_1][j,2] - bead_cog_[active_bead_2][j,2])**2)**0.5

        # combining the different distances for similar bead connections together (e.g. 111-112 + 211-212 + 311-312 +
... + 10011-10012)
        for combi in range(len(distance_combination_indices)):
            combined_bead_distance[combi] = np.array([])
            for numbeads in range(num_mols):
                combi_bead_array[numbeads] = bead_distance_[distance_combination_indices[combi,numbeads]]
                combined_bead_distance[combi]                                                                  =
np.concatenate([combined_bead_distance[combi],combi_bead_array[numbeads]], axis=None)

            average_dist[combi,temp] = np.mean(combined_bead_distance[combi])
            stdev_dist[combi,temp]   = np.std(combined_bead_distance[combi])
            histo_temp_bond          = np.histogram(combined_bead_distance[combi], bins=30, range=[0.2,0.5])
            histo_dist[combi,:,temp] = histo_temp_bond[0]


#######################################
## CALCULATING ANGLES AND STATISTICS ##
#######################################

        for i in range(len(angle_connections_list)):
            active_bead_1 = angle_connections_list[i,0]
            active_bead_2 = angle_connections_list[i,1]
            active_bead_3 = angle_connections_list[i,2]
            bead_angle_[i] = np.ndarray(shape=(num_frames))
            for j in range(num_frames):
                ba = bead_cog_[active_bead_1][j]-bead_cog_[active_bead_2][j]
                bc = bead_cog_[active_bead_3][j]-bead_cog_[active_bead_2][j]
                bead_angle_[i][j] = np.degrees(
                                        np.arccos(
                                            np.dot(ba,bc)/(np.linalg.norm(ba) * np.linalg.norm(bc))))

        for combi in range(len(angle_combination_indices)):
            combined_bead_angle[combi] = np.ndarray([])
            for numbeads in range(num_mols):
                combi_angle_array[numbeads] = bead_angle_[angle_combination_indices[combi,numbeads]]
                combined_bead_angle[combi] = np.concatenate([combined_bead_angle[combi],combi_angle_array[numbeads]],
axis=None)

            average_angle[combi,temp]    = np.mean(combined_bead_angle[combi])
            stdev_angle[combi,temp]      = np.std(combined_bead_angle[combi])
            histo_temp_angle             = np.histogram(combined_bead_angle[combi], bins=26, range=[50,180])
            histo_angle[combi,:,temp]    = histo_temp_angle[0]


##########################################
## CALCULATING DIHEDRALS AND STATISTICS ##
##########################################

        for i in range(len(dihedral_connections_list)):
            active_bead_1 = dihedral_connections_list[i,0]
            active_bead_2 = dihedral_connections_list[i,1]
            active_bead_3 = dihedral_connections_list[i,2]
            active_bead_4 = dihedral_connections_list[i,3]
            bead_dihedral_[i] = np.ndarray(shape=(num_frames))
            for j in range(num_frames):
                a1 = -1.0*(bead_cog_[active_bead_2][j]-bead_cog_[active_bead_1][j])
                a2 = bead_cog_[active_bead_3][j]-bead_cog_[active_bead_2][j]
                a3 = bead_cog_[active_bead_4][j]-bead_cog_[active_bead_3][j]

                a2 /= np.linalg.norm(a2)
                v = a1 - np.dot(a1, a2) * a2
                w = a3 - np.dot(a3, a2) * a2
                x = np.dot(v, w)
                y = np.dot(np.cross(a2 , v) ,w)
                bead_dihedral_[i][j] = np.degrees(np.arctan2(y, x)) + 180

        for combi in range(len(dihedral_combination_indices)):
            combined_bead_dihedral[combi] = np.array([])
            for numbeads in range(num_mols):
```

```
                combi_dihedral_array[numbeads] = bead_dihedral_[dihedral_combination_indices[combi,numbeads]]
                combined_bead_dihedral[combi]                                                                         =
np.concatenate([combined_bead_dihedral[combi],combi_dihedral_array[numbeads]], axis=None)

            average_dihedral[combi,temp]    = np.mean(combined_bead_dihedral[combi])
            stdev_dihedral[combi,temp]      = np.std(combined_bead_dihedral[combi])
            histo_temp_dihedral             = np.histogram(combined_bead_dihedral[combi], bins=36, range=[0,360])
            histo_dihedral[combi,:,temp]    = histo_temp_dihedral[0]

        print(str("\t") + str(structure[struct]) + " @ " + str(temperatures[temp]) + str(" is done!"))
        print("\n")

    output_file = wdir + TAG + "-" + structure[struct]  + "-output.ods"
    with open(output_file, "w") as outfile:
        outfile.write("Distances")
        outfile.write("\n")
        outfile.write(str("\t")*2 + str("Averages") + (str("_") + str(" "))*7 + str("Standard_Deviations"))
        outfile.write("\n")
        outfile.write(str("Bead_names") + str(temperatures) + str("\t") + str(temperatures))
        outfile.write("\n")

        for j in range(len(combined_bead_names)):
            active_beads = combined_bead_names[j] # bead names for bead distances
            outfile.write(str(active_beads)  +  str("\t")  +  str(average_dist[j,:]).replace('\n',  '')  +  str("\t")  +
str(stdev_dist[j,:]).replace('\n', ''))
            outfile.write("\n")
        outfile.write("\n")

        for j in range(len(combined_bead_names)):
            active_beads = combined_bead_names[j] # bead names for bead distances
            for temp in range(len(temperatures)):
                outfile.write(str(active_beads)      +      str("      ")      +      str(temperatures[temp])      +
str(histo_dist[j,:,temp]).replace('\n', ''))
                outfile.write("\n")
            outfile.write("\n")
        outfile.write("\n")
        outfile.write("\n")


        outfile.write("Angles")
        outfile.write("\n")
        outfile.write(str("\t")*2 + str("Averages") + (str("_") + str(" "))*7 + str("Standard_Deviations"))
        outfile.write("\n")
        outfile.write(str("Bead_names") + str(temperatures) + str("\t") + str(temperatures))
        outfile.write("\n")

        for j in range(len(combined_angle_names)):
            active_beads = combined_angle_names[j] # bead names for bead distances
            outfile.write(str(active_beads)  +  str("\t")  +  str(average_angle[j,:]).replace('\n',  '')  +  str("\t")  +
str(stdev_angle[j,:]).replace('\n', ''))
            outfile.write("\n")
        outfile.write("\n")

        for j in range(len(combined_angle_names)):
            active_beads = combined_angle_names[j] # bead names for bead distances
            for temp in range(len(temperatures)):
                outfile.write(str(active_beads)      +      str("      ")      +      str(temperatures[temp])      +
str(histo_angle[j,:,temp]).replace('\n', ''))
                outfile.write("\n")
            outfile.write("\n")
        outfile.write("\n")
        outfile.write("\n")


        outfile.write("Dihedrals")
        outfile.write("\n")
        outfile.write(str("\t")*2 + str("Averages") + (str("_") + str(" "))*7 + str("Standard_Deviations"))
        outfile.write("\n")
        outfile.write(str("Bead_names") + str(temperatures) + str("\t") + str(temperatures))
        outfile.write("\n")

        for j in range(len(combined_dihedral_names)):
            active_beads = combined_dihedral_names[j] # bead names for bead distances
            outfile.write(str(active_beads) + str("\t") + str(average_dihedral[j,:]).replace('\n', '') + str("\t") +
str(stdev_dihedral[j,:]).replace('\n', ''))
            outfile.write("\n")
        outfile.write("\n")

        for j in range(len(combined_dihedral_names)):
            active_beads = combined_dihedral_names[j] # bead names for bead distances
            for temp in range(len(temperatures)):
                outfile.write(str(active_beads)      +      str("      ")      +      str(temperatures[temp])      +
str(histo_dihedral[j,:,temp]).replace('\n', ''))
                outfile.write("\n")
            outfile.write("\n")
```

```
        outfile.write("\n")
        outfile.write("\n")

    print(str(output_file) + str(" is printed"))

duration = (time.time() - start_time)/60
print(" ")
print(TAG + str(" data analysis took ") + str("%.2f" % duration) + str(" minutes"))
```

SI.2 Plots of potential energy (kJ/mol) versus torsion angle for 4-body bead systems, using Centre of Geometries determined from the UA simulations.

In the cases where the same legend label appears more than once in the same plot, this is due to that dihedral system being present in that molecule more than once.

All data was collected from 0 to 360° in bins of 10°.

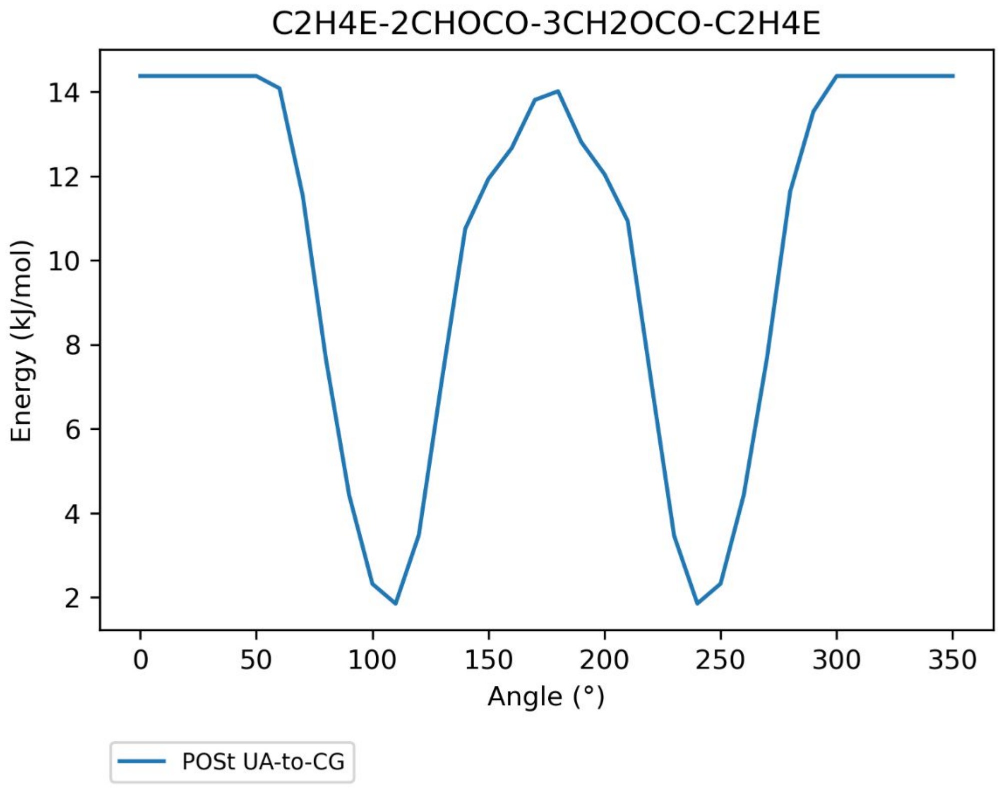

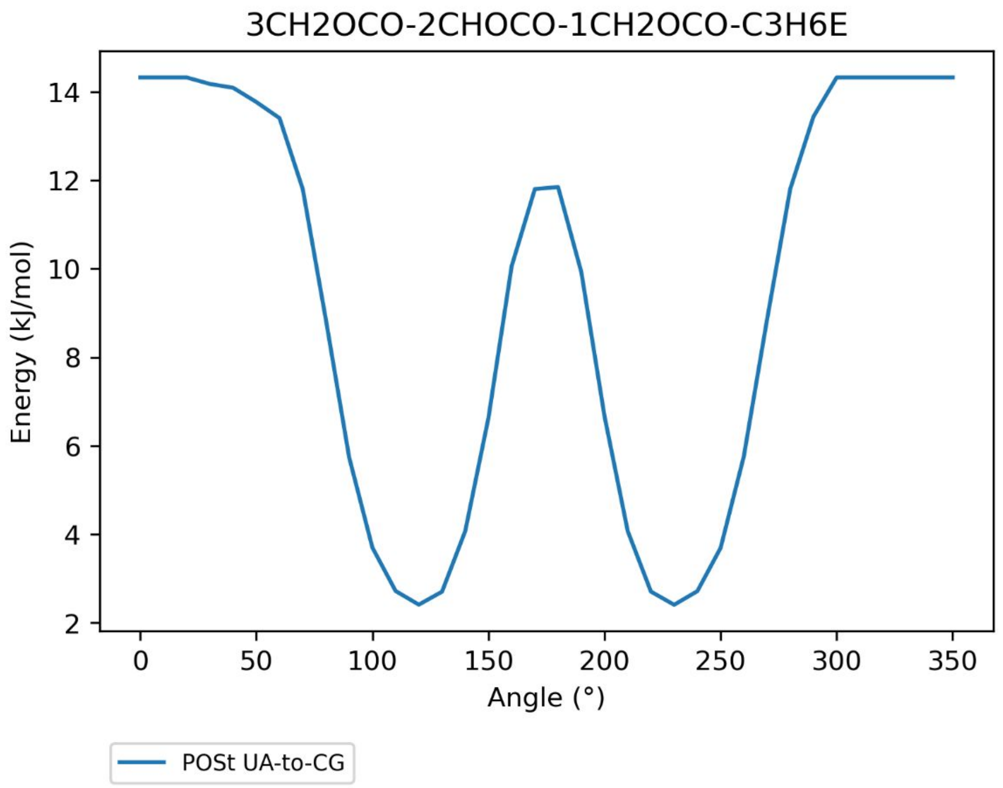
3CH2OCO-2CHOCO-1CH2OCO-C3H6E
Energy (kJ/mol)
Angle (°)
POSt UA-to-CG

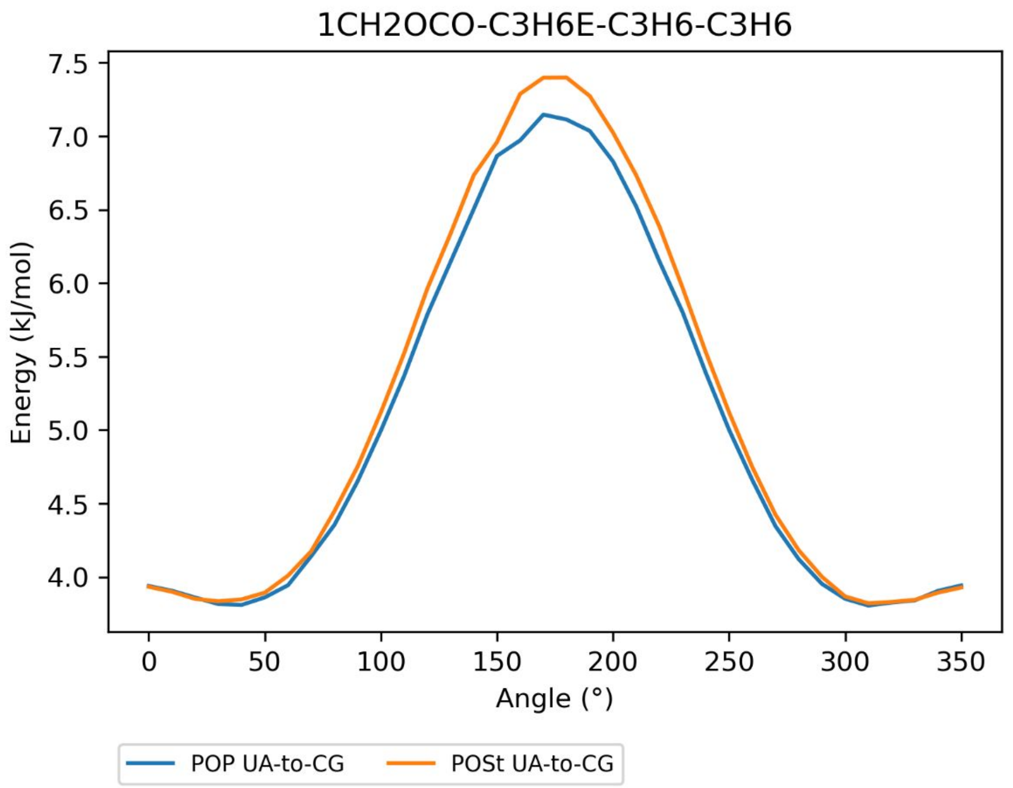
1CH2OCO-C3H6E-C3H6-C3H6
Energy (kJ/mol)
Angle (°)
POP UA-to-CG
POSt UA-to-CG

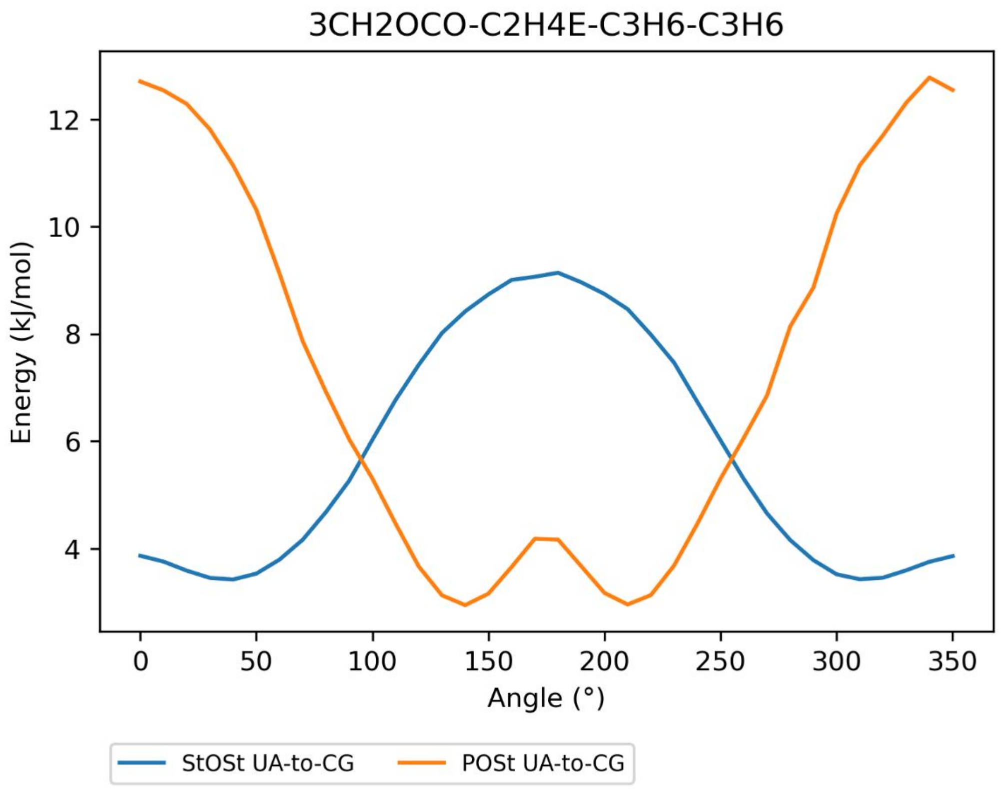
3CH2OCO-C2H4E-C3H6-C3H6
Energy (kJ/mol)
Angle (°)
StOSt UA-to-CG
POSt UA-to-CG

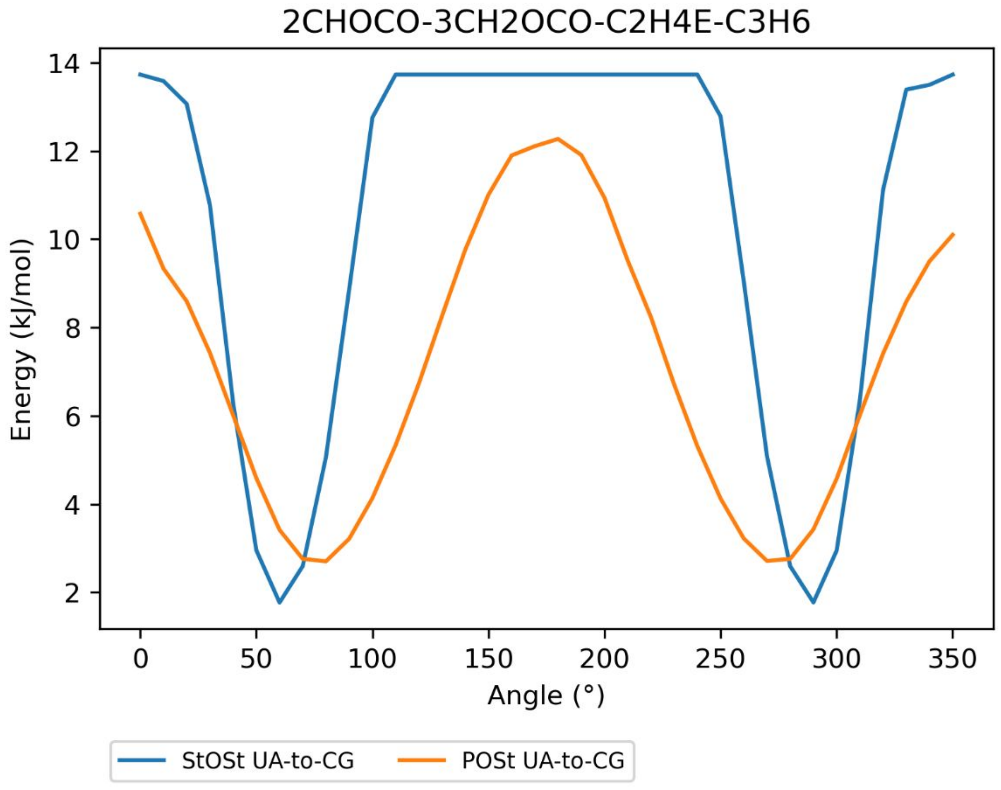
2CHOCO-3CH2OCO-C2H4E-C3H6
Energy (kJ/mol)
Angle (°)
StOSt UA-to-CG
POSt UA-to-CG

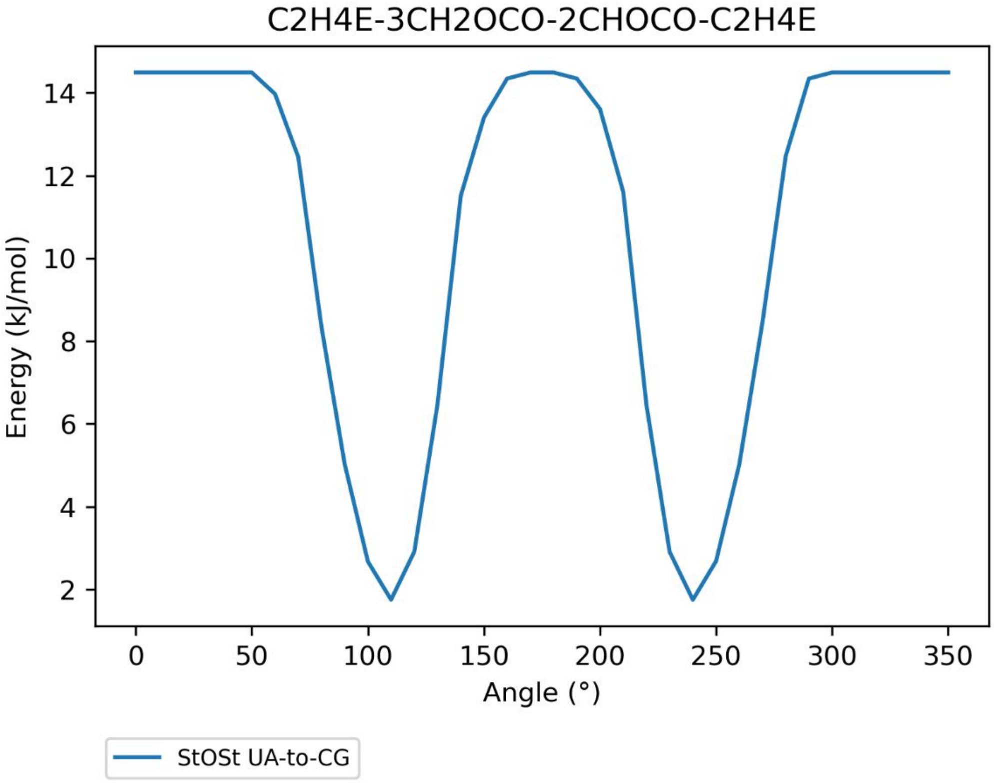

C2H4E-3CH2OCO-2CHOCO-C2H4E
Energy (kJ/mol)
Angle (°)
StOSt UA-to-CG

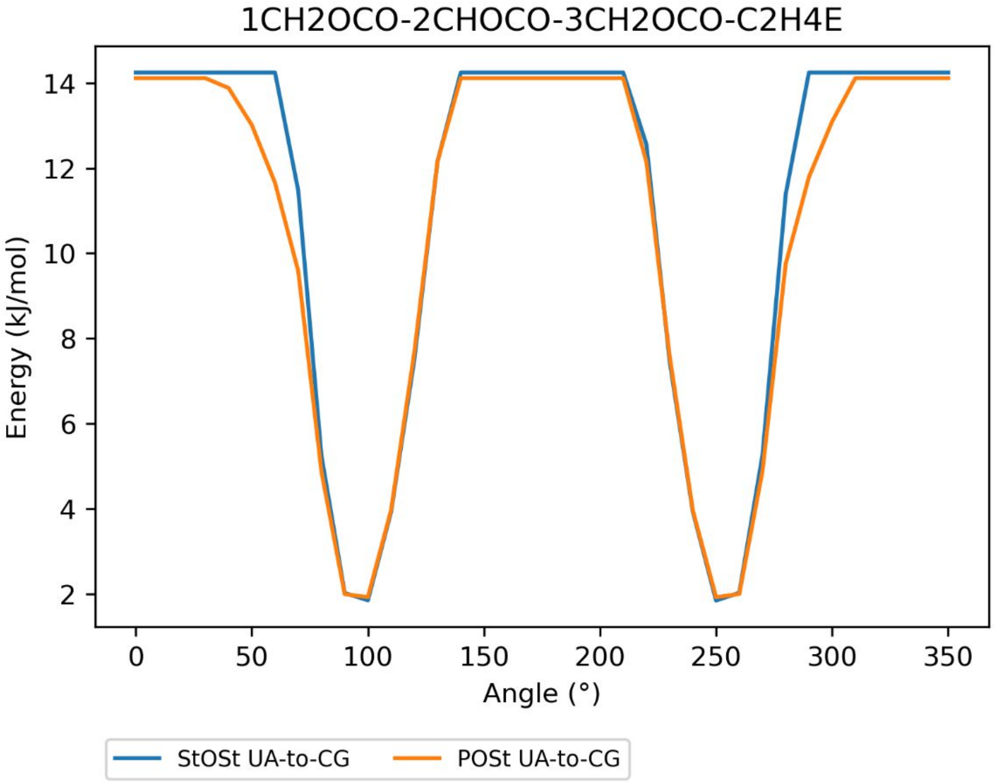

1CH2OCO-2CHOCO-3CH2OCO-C2H4E
Energy (kJ/mol)
Angle (°)
StOSt UA-to-CG
POSt UA-to-CG

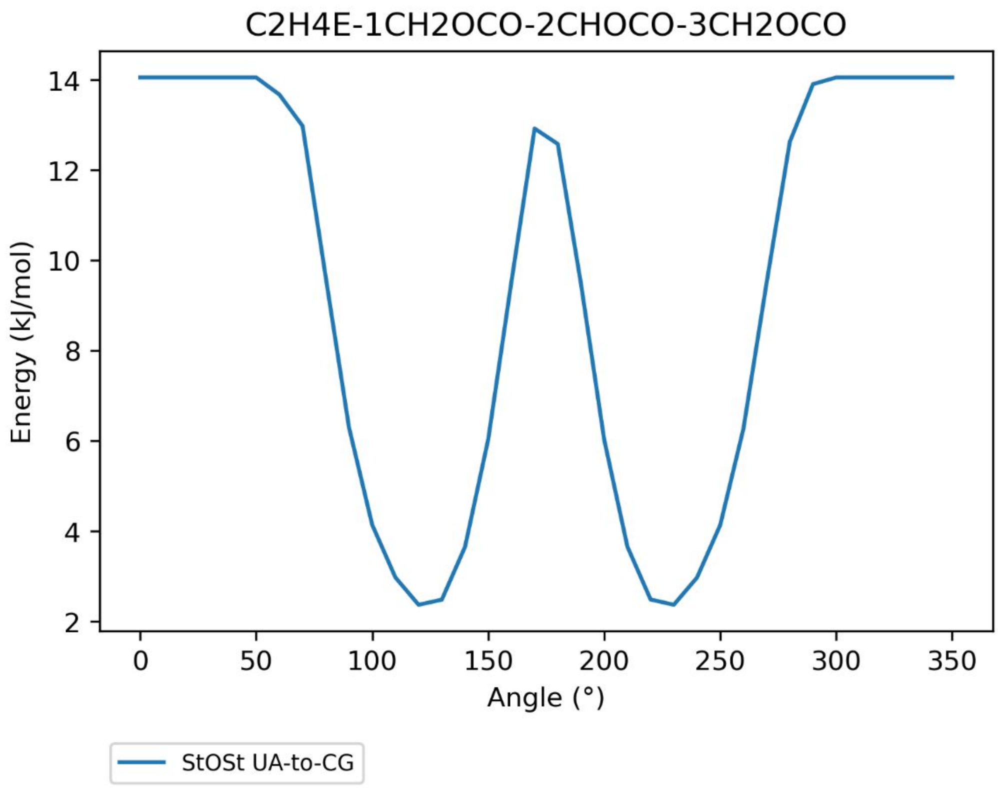
C2H4E-1CH2OCO-2CHOCO-3CH2OCO
Energy (kJ/mol)
Angle (°)
StOSt UA-to-CG

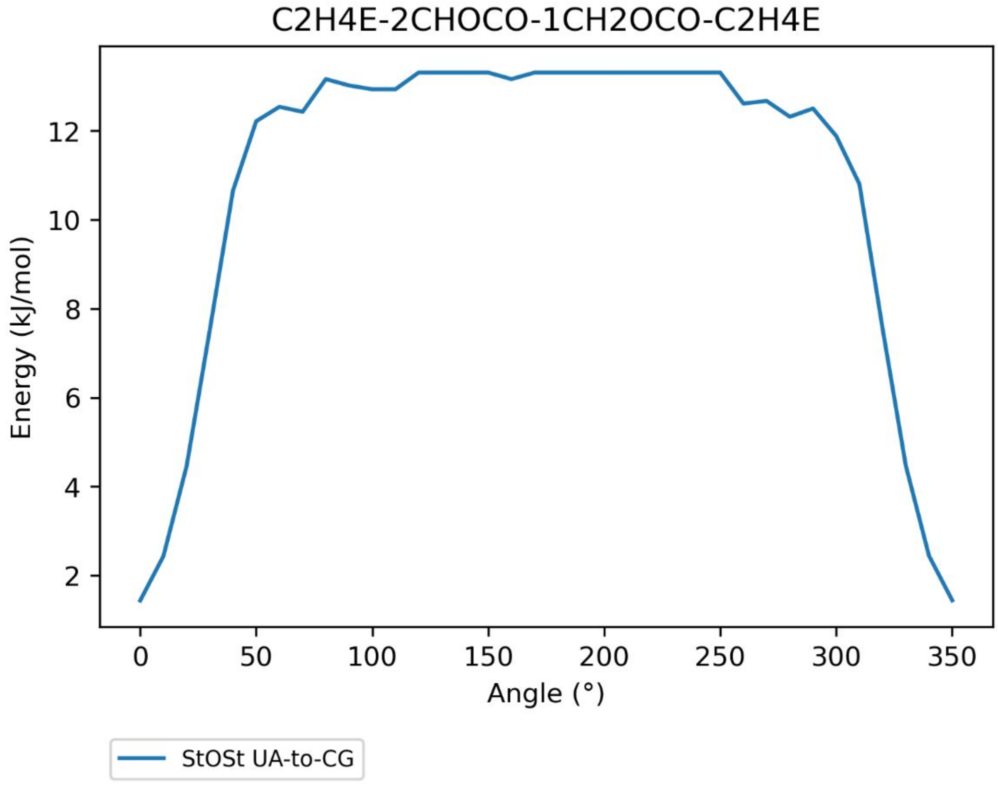
C2H4E-2CHOCO-1CH2OCO-C2H4E
Energy (kJ/mol)
Angle (°)
StOSt UA-to-CG

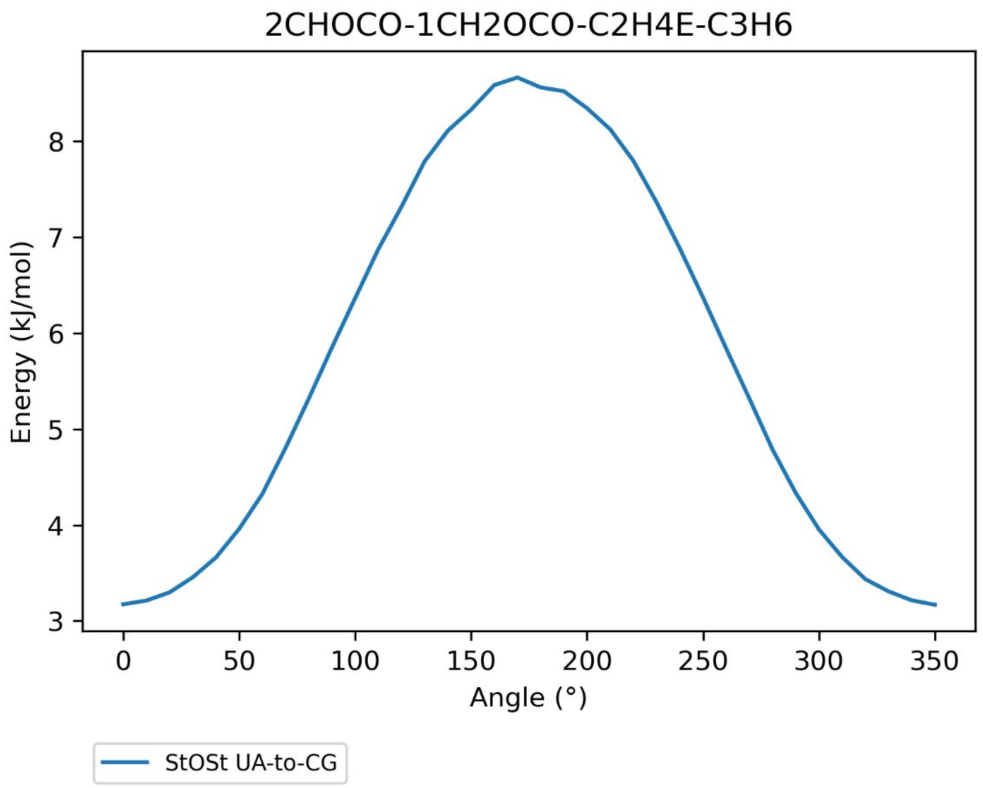

2CHOCO-1CH2OCO-C2H4E-C3H6
Energy (kJ/mol)
3
4
5
6
7
8
0
50
100
150
200
250
300
350
Angle (°)
StOSt UA-to-CG

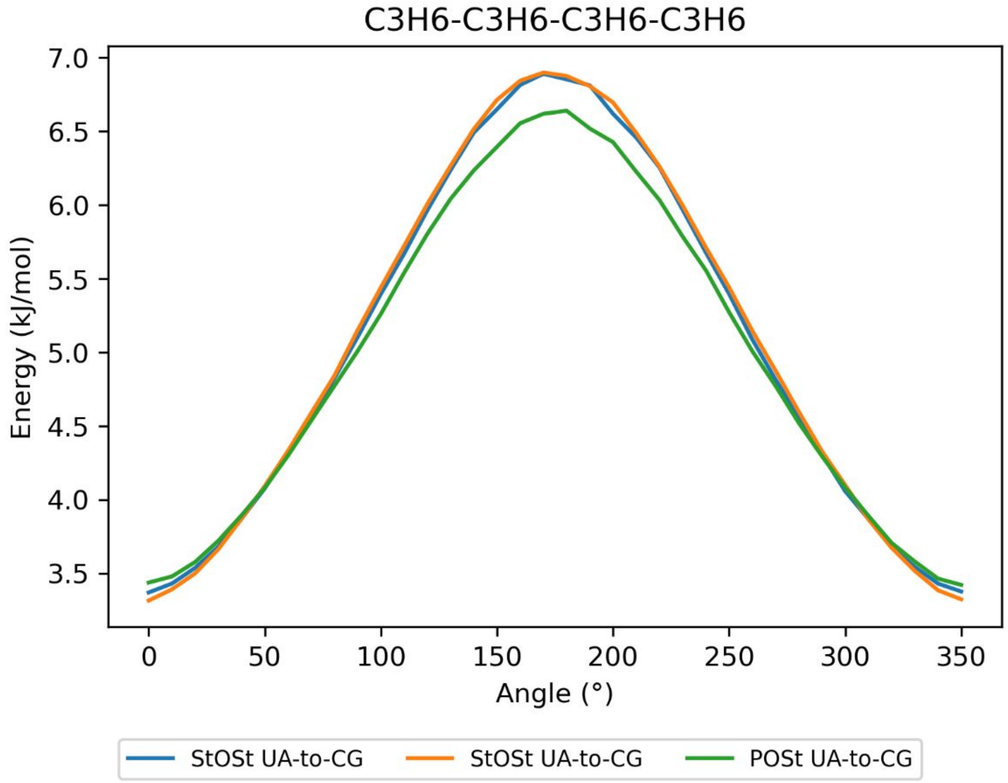

C3H6-C3H6-C3H6-C3H6
Energy (kJ/mol)
3.5
4.0
4.5
5.0
5.5
6.0
6.5
7.0
0
50
100
150
200
250
300
350
Angle (°)
StOSt UA-to-CG
StOSt UA-to-CG
POSt UA-to-CG

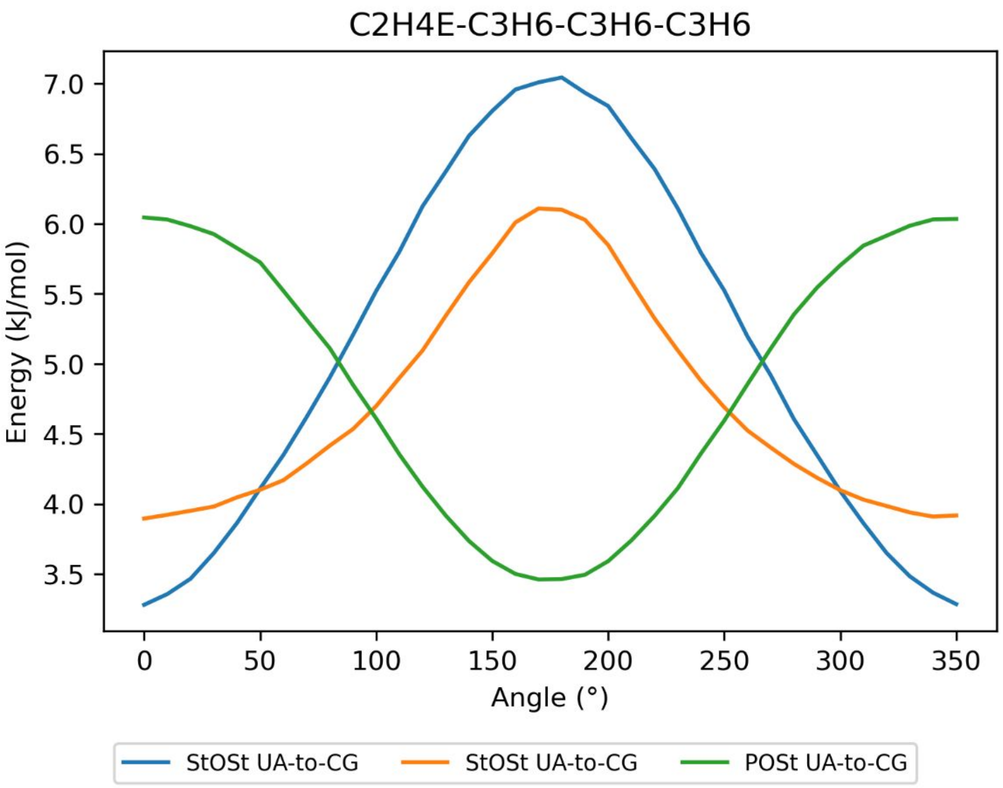
C2H4E-C3H6-C3H6-C3H6
Energy (kJ/mol)
Angle (°)
StOSt UA-to-CG
StOSt UA-to-CG
POSt UA-to-CG

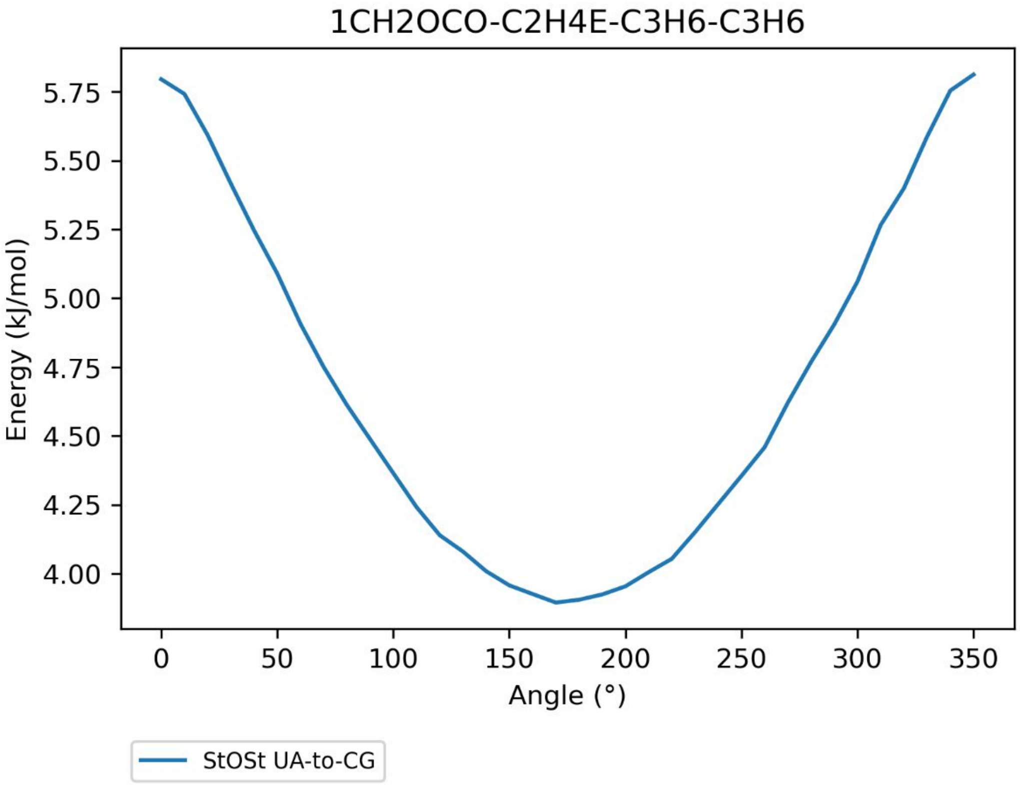
1CH2OCO-C2H4E-C3H6-C3H6
Energy (kJ/mol)
Angle (°)
StOSt UA-to-CG

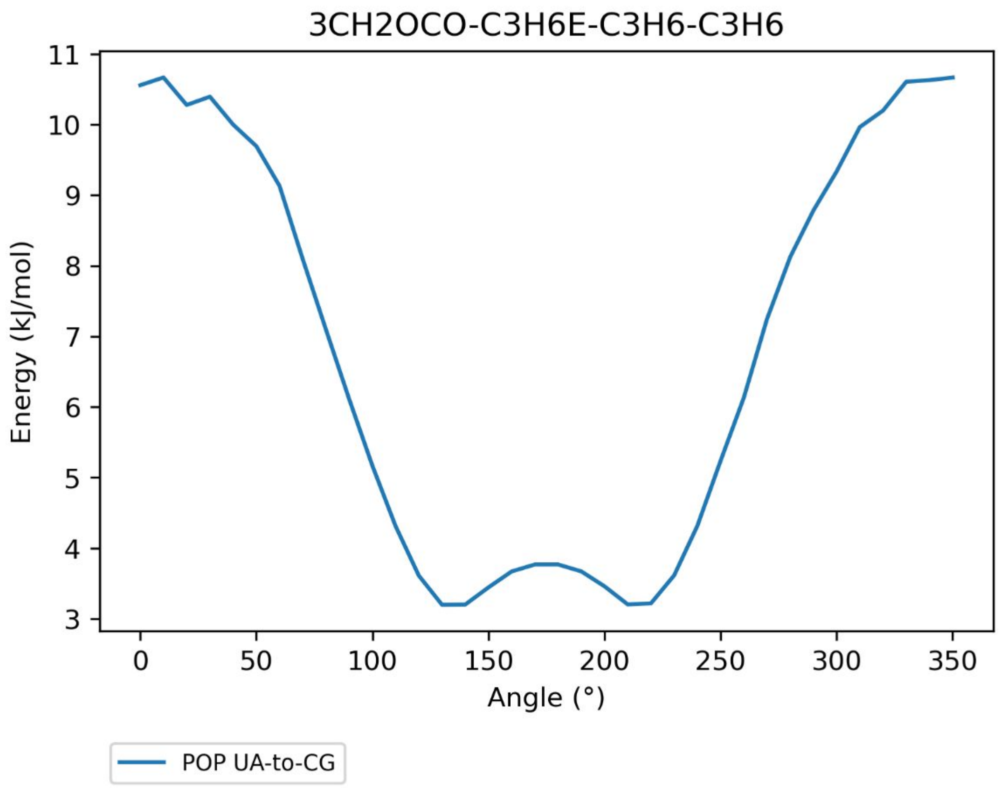
3CH2OCO-C3H6E-C3H6-C3H6
Energy (kJ/mol)
11
10
9
8
7
6
5
4
3
0
50
100
150
200
250
300
350
Angle (°)
POP UA-to-CG

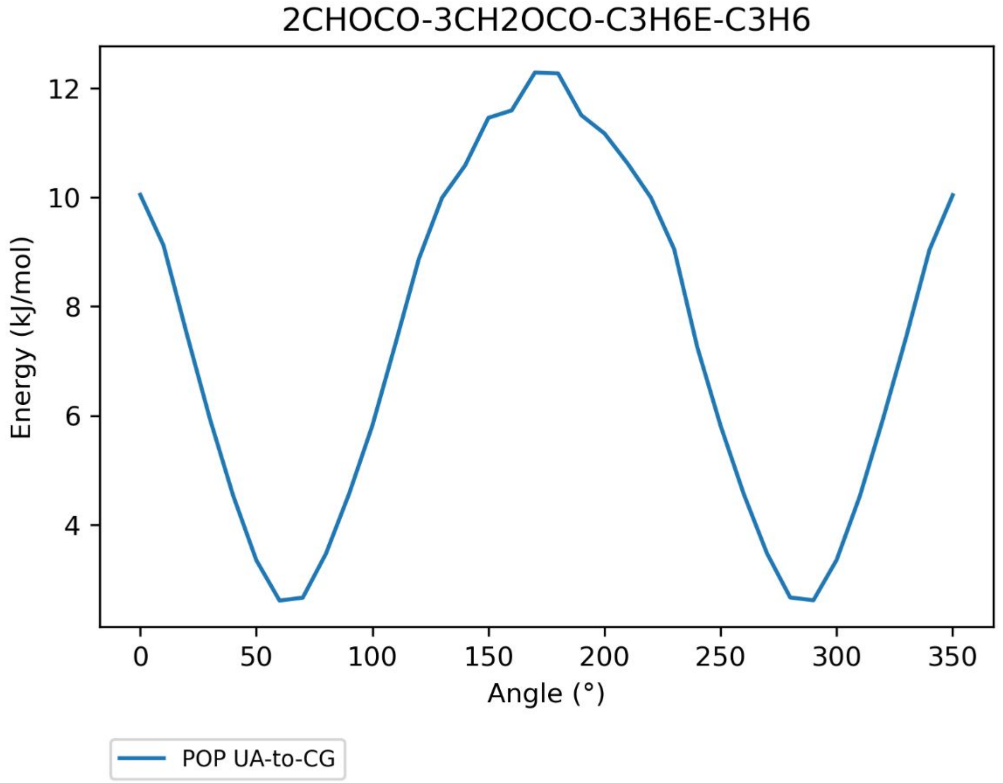
2CHOCO-3CH2OCO-C3H6E-C3H6
Energy (kJ/mol)
12
10
8
6
4
0
50
100
150
200
250
300
350
Angle (°)
POP UA-to-CG

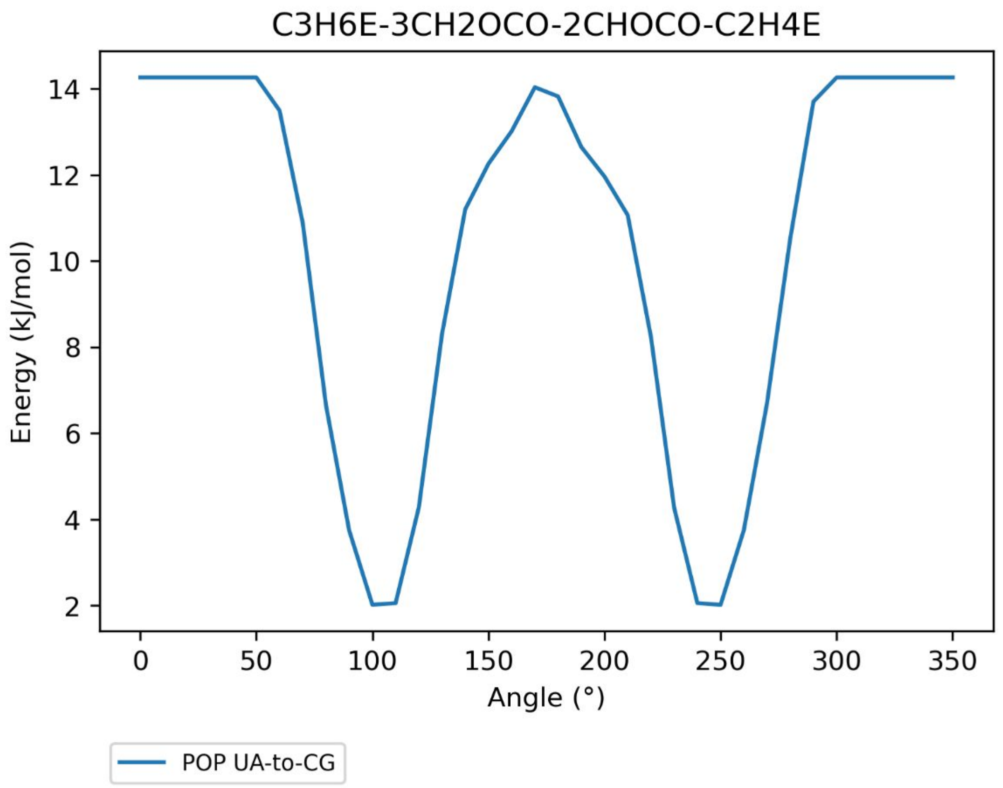
C3H6E-3CH2OCO-2CHOCO-C2H4E
Energy (kJ/mol)
Angle (°)
POP UA-to-CG

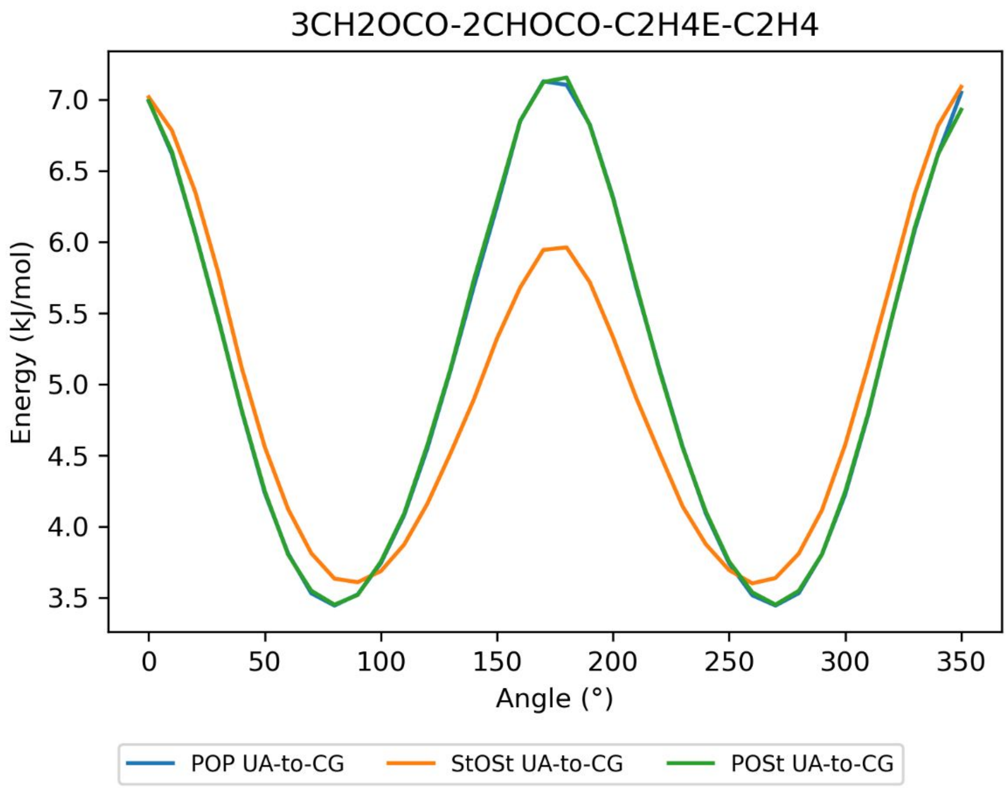
3CH2OCO-2CHOCO-C2H4E-C2H4
Energy (kJ/mol)
Angle (°)
POP UA-to-CG
StOSt UA-to-CG
POSt UA-to-CG

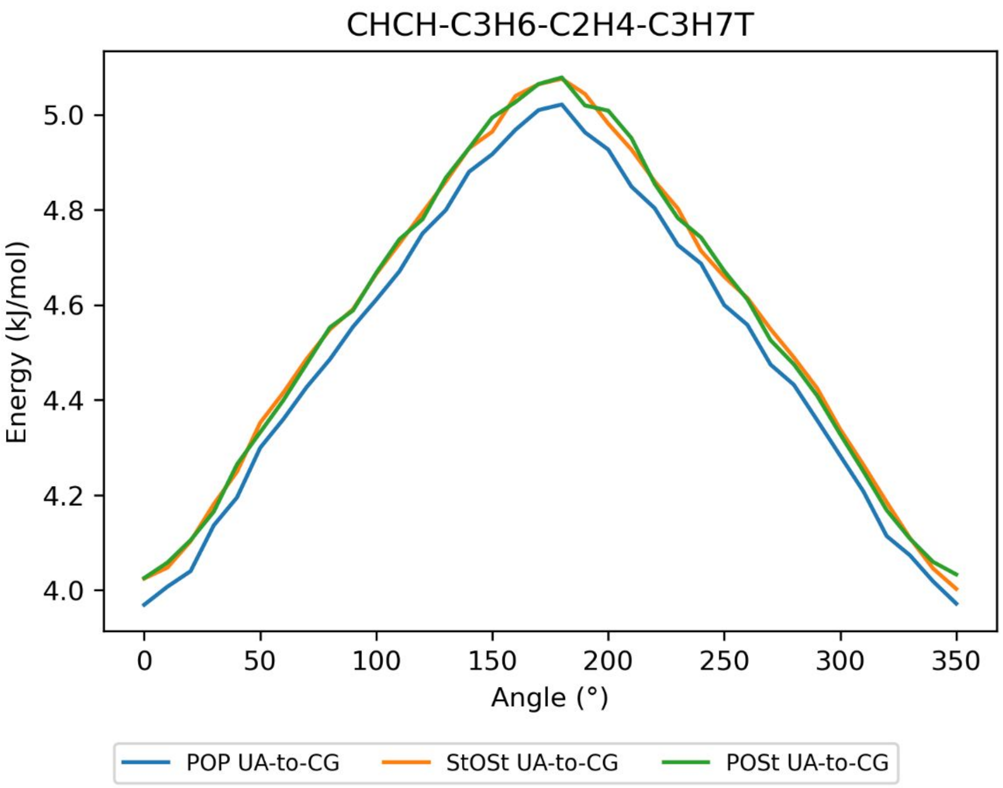
CHCH-C3H6-C2H4-C3H7T
Energy (kJ/mol)
Angle (°)
5.0
4.8
4.6
4.4
4.2
4.0
0
50
100
150
200
250
300
350
POP UA-to-CG
StOSt UA-to-CG
POSt UA-to-CG

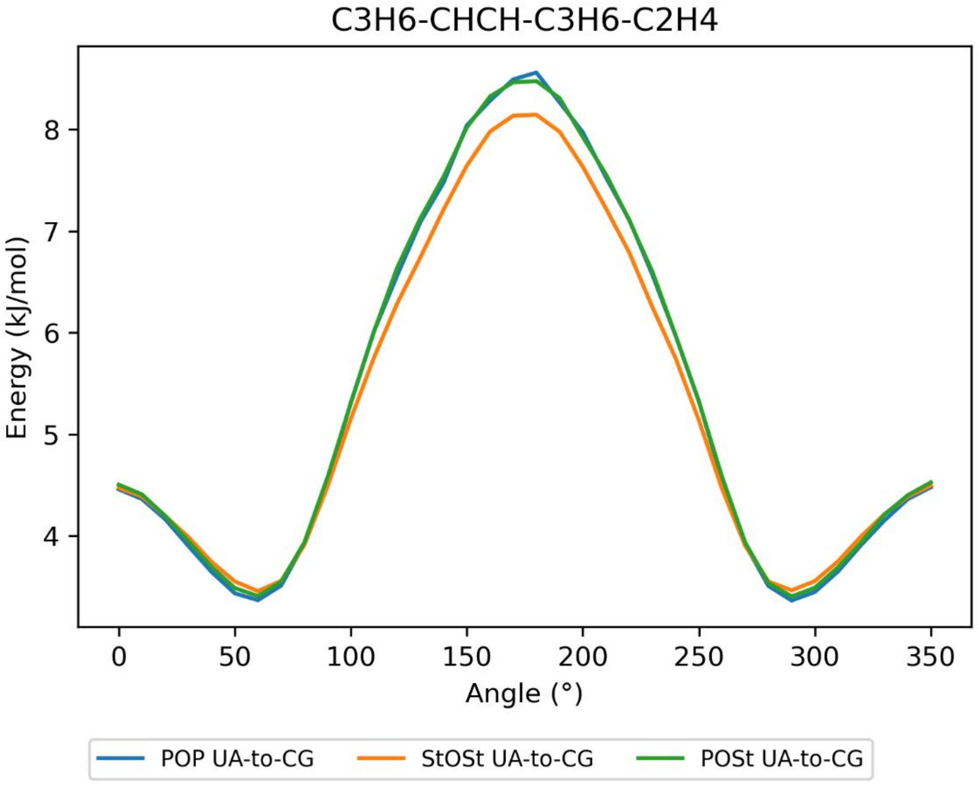
C3H6-CHCH-C3H6-C2H4
Energy (kJ/mol)
Angle (°)
8
7
6
5
4
0
50
100
150
200
250
300
350
POP UA-to-CG
StOSt UA-to-CG
POSt UA-to-CG

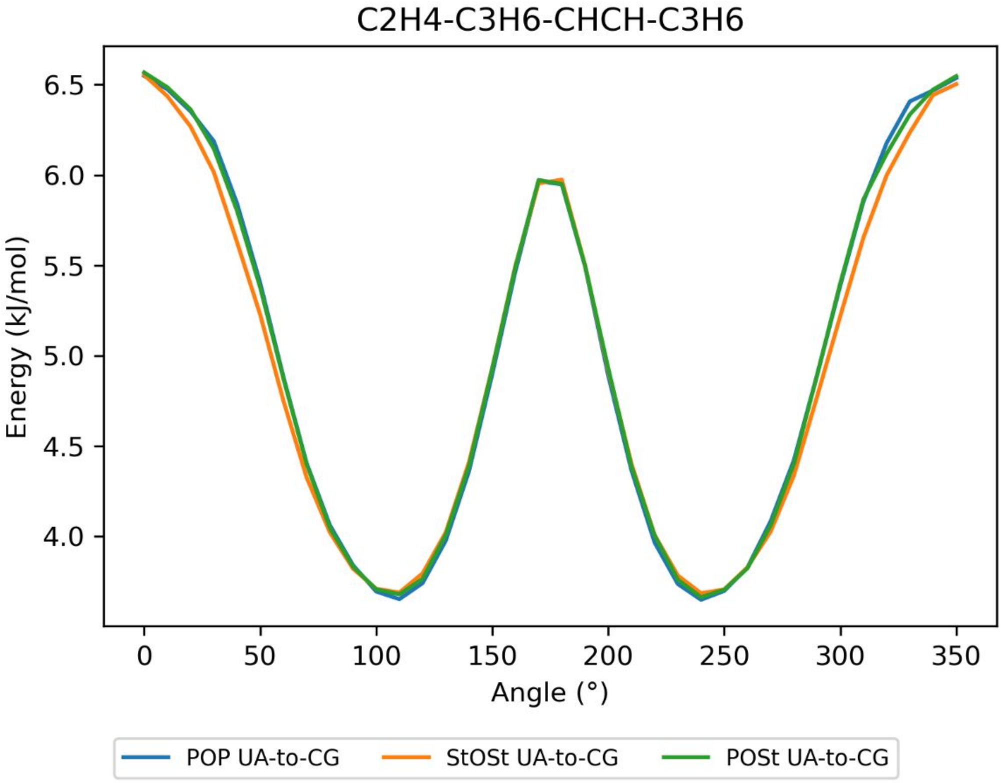

C2H4-C3H6-CHCH-C3H6
Energy (kJ/mol)
Angle (°)
POP UA-to-CG
StOSt UA-to-CG
POSt UA-to-CG

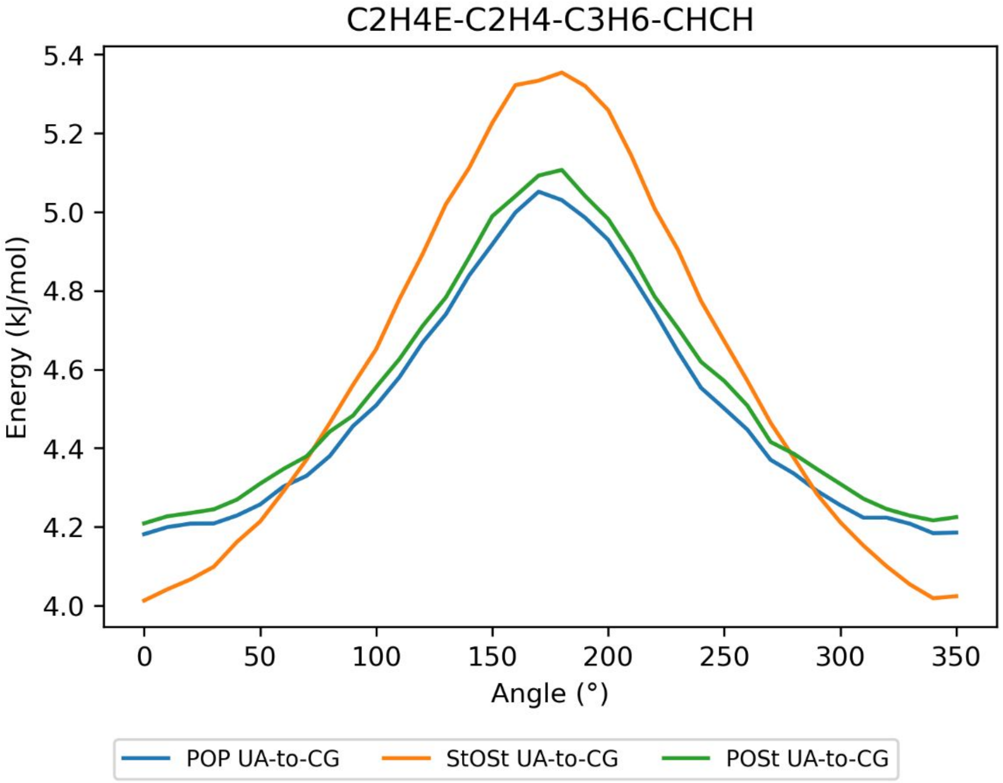

C2H4E-C2H4-C3H6-CHCH
Energy (kJ/mol)
Angle (°)
POP UA-to-CG
StOSt UA-to-CG
POSt UA-to-CG

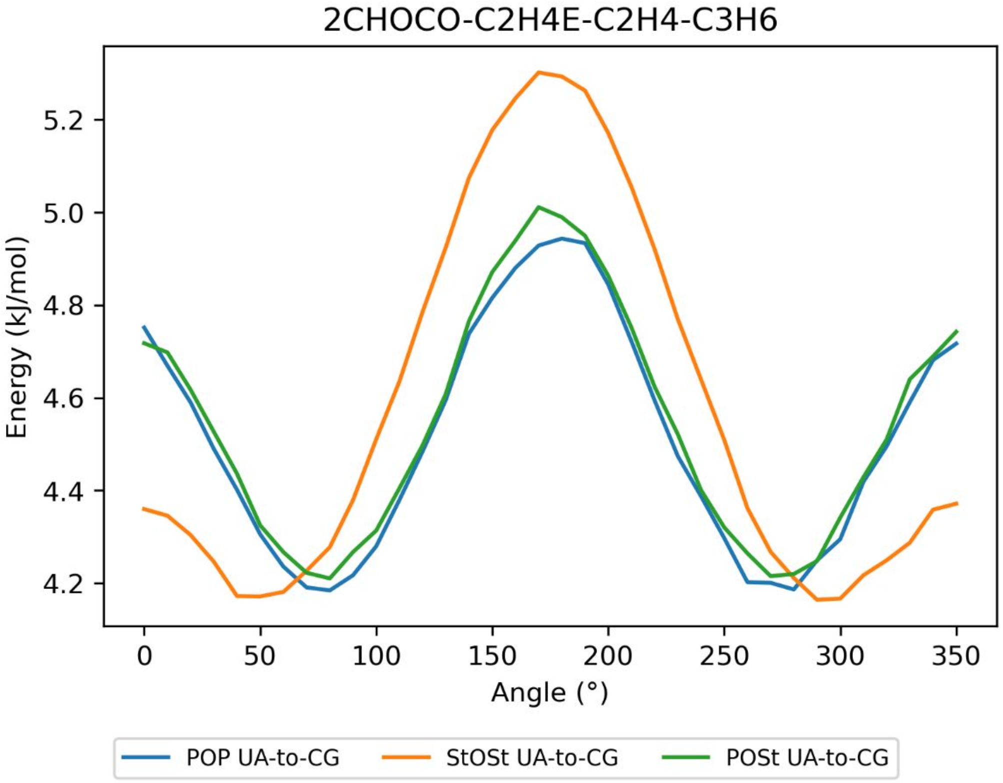
2CHOCO-C2H4E-C2H4-C3H6
Energy (kJ/mol)
Angle (°)
5.2
5.0
4.8
4.6
4.4
4.2
0
50
100
150
200
250
300
350
POP UA-to-CG
StOSt UA-to-CG
POSt UA-to-CG

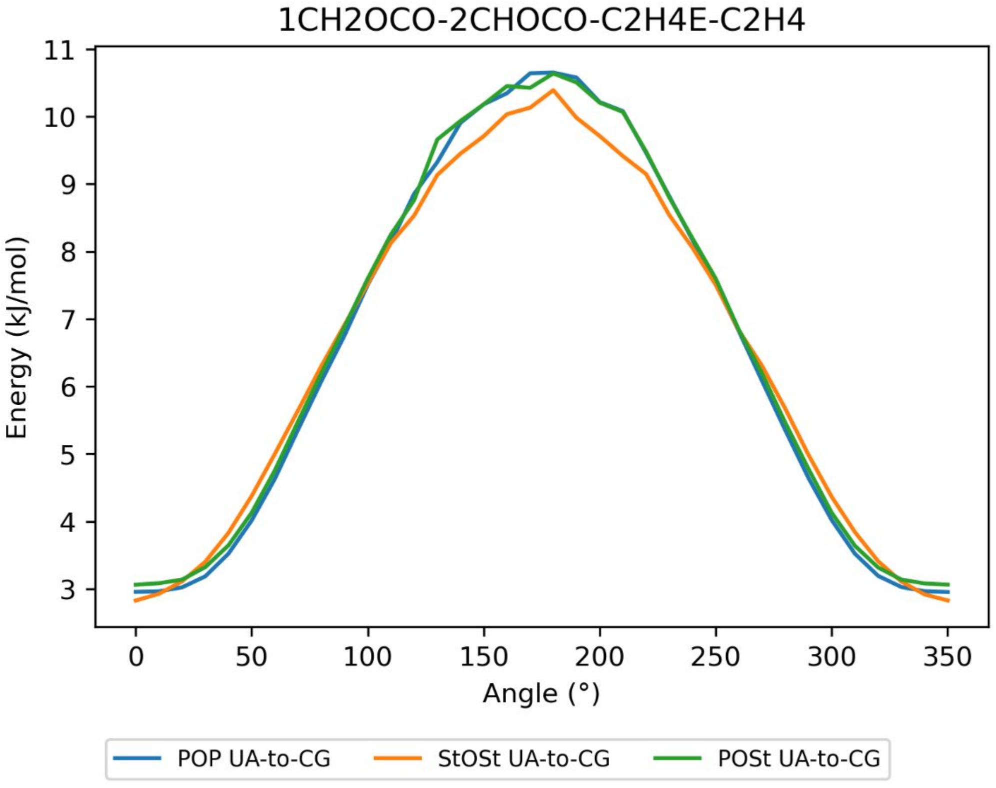
1CH2OCO-2CHOCO-C2H4E-C2H4
Energy (kJ/mol)
Angle (°)
11
10
9
8
7
6
5
4
3
0
50
100
150
200
250
300
350
POP UA-to-CG
StOSt UA-to-CG
POSt UA-to-CG

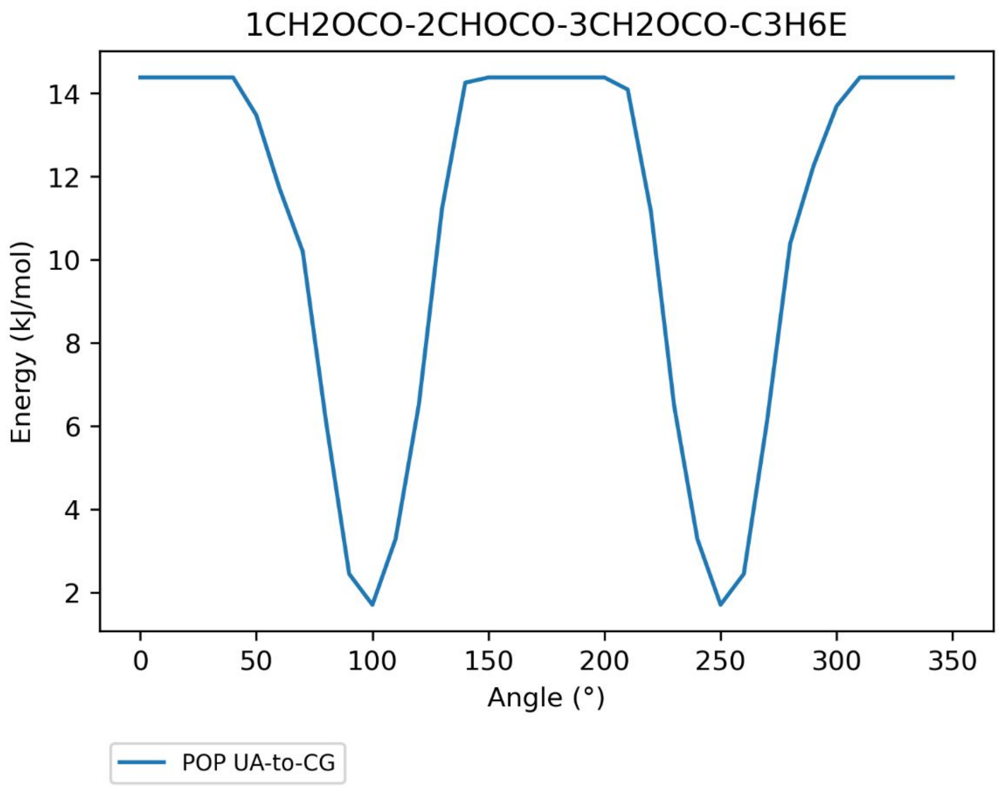
1CH2OCO-2CHOCO-3CH2OCO-C3H6E
Energy (kJ/mol)
Angle (°)
POP UA-to-CG

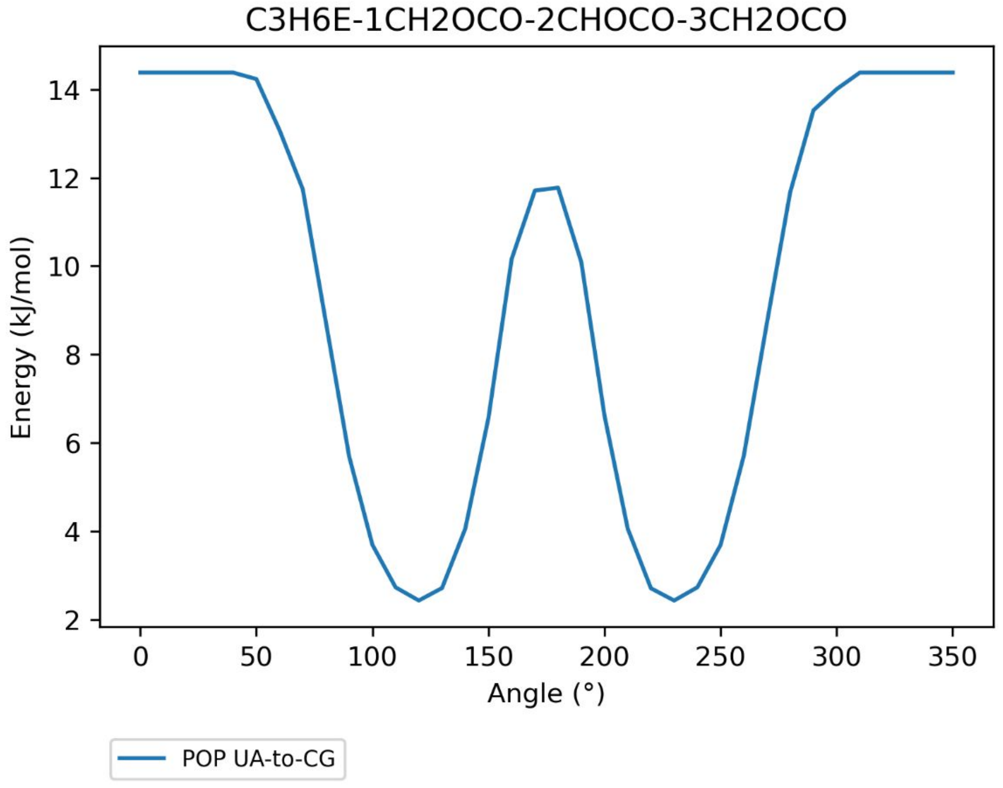
C3H6E-1CH2OCO-2CHOCO-3CH2OCO
Energy (kJ/mol)
Angle (°)
POP UA-to-CG

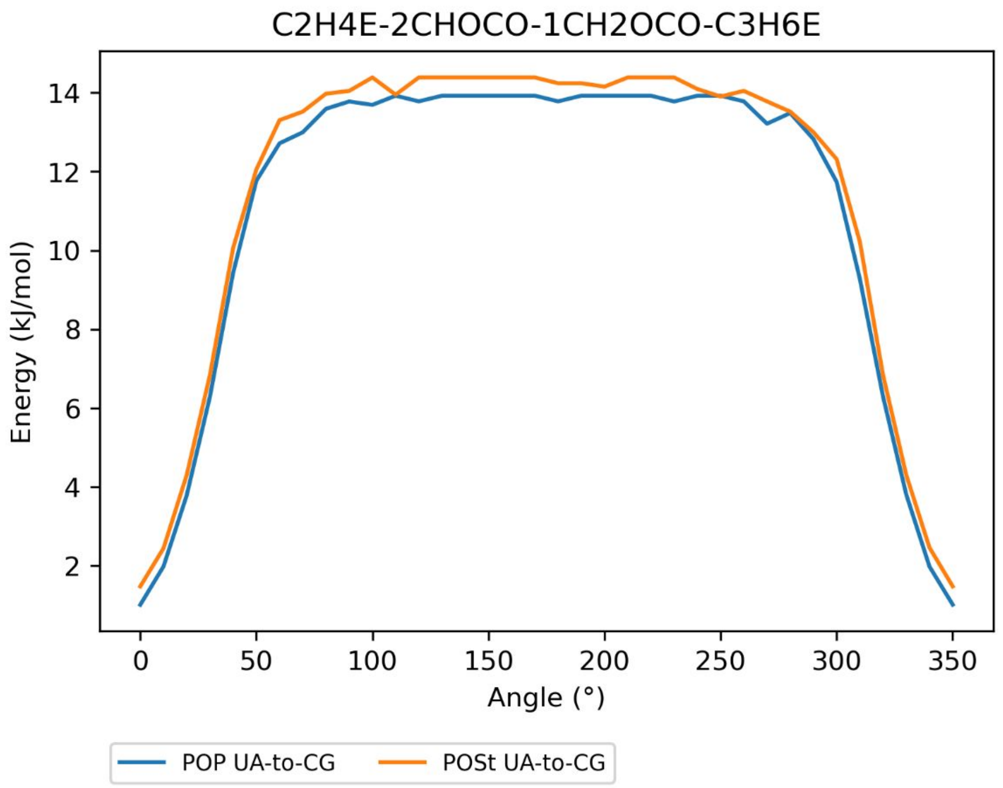
C2H4E-2CHOCO-1CH2OCO-C3H6E
Energy (kJ/mol)
Angle (°)
0
50
100
150
200
250
300
350
2
4
6
8
10
12
14
POP UA-to-CG
POSt UA-to-CG

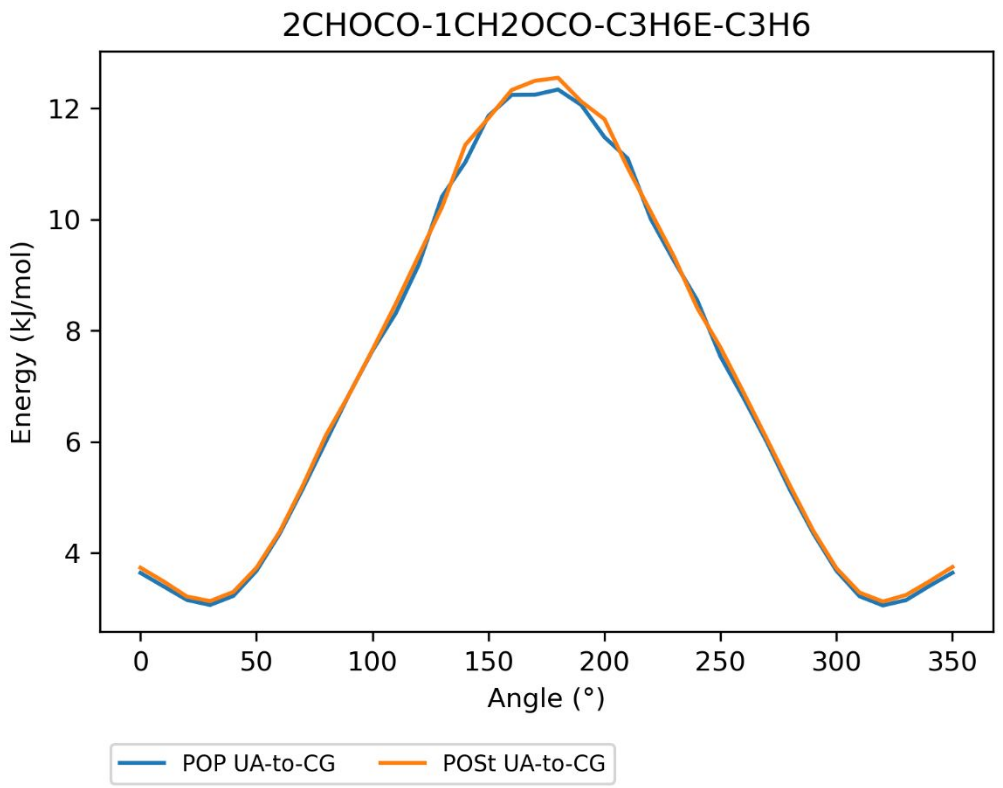
2CHOCO-1CH2OCO-C3H6E-C3H6
Energy (kJ/mol)
Angle (°)
0
50
100
150
200
250
300
350
4
6
8
10
12
POP UA-to-CG
POSt UA-to-CG

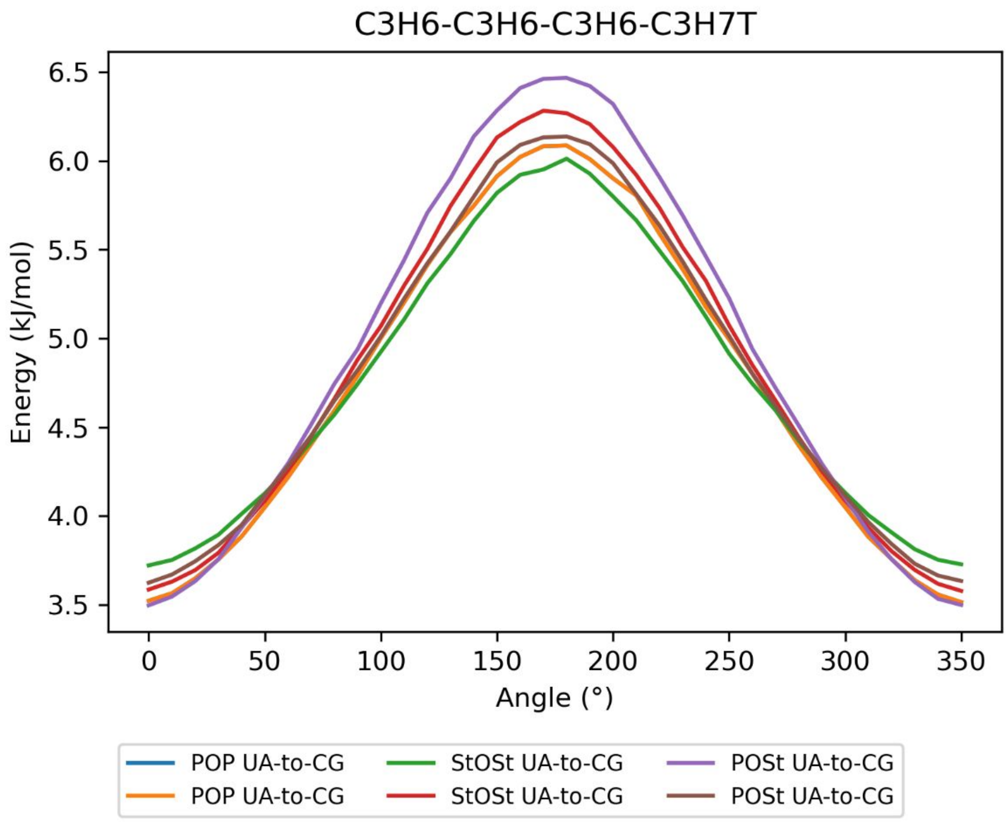
C3H6-C3H6-C3H6-C3H7T
Energy (kJ/mol)
Angle (°)
POP UA-to-CG
POP UA-to-CG
StOSt UA-to-CG
StOSt UA-to-CG
POSt UA-to-CG
POSt UA-to-CG

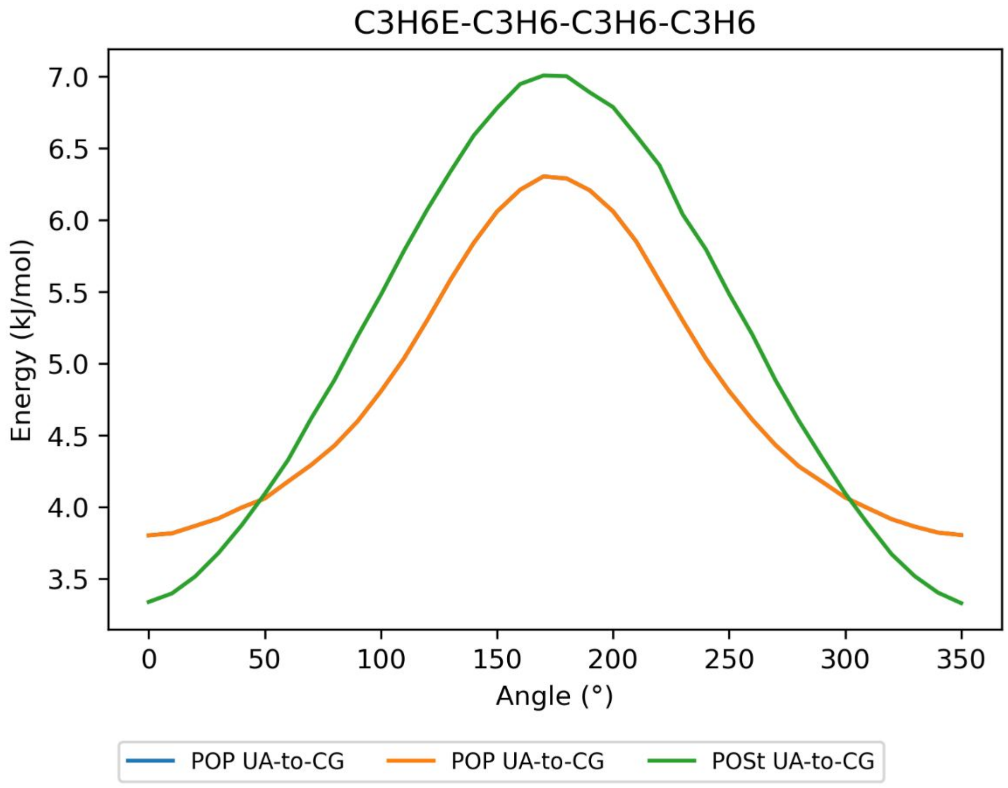
C3H6E-C3H6-C3H6-C3H6
Energy (kJ/mol)
Angle (°)
POP UA-to-CG
POP UA-to-CG
POSt UA-to-CG

SI.3 Plots comparing UA (CG-mapped) bond distances with CG bond distances

Plots labelled as "UA-to-CG" show the distance distribution of the centre-of-geometry distances of the atoms in the UA simulations which were mapped as CG beads. All other plots are for the CG simulations. In the cases were the same legend label appears more than once in one plot, this is due to that bead type pair being present in that molecule more than once (e.g. the C3H6-C3H6 bead pair is present three times in a stearic acid chain, and with two stearic acid chains in StOSt, this bead pair is found a total of six times).

All data was collected from 0.2 to 0.5 nm in 30 bins. All plots have been normalized.

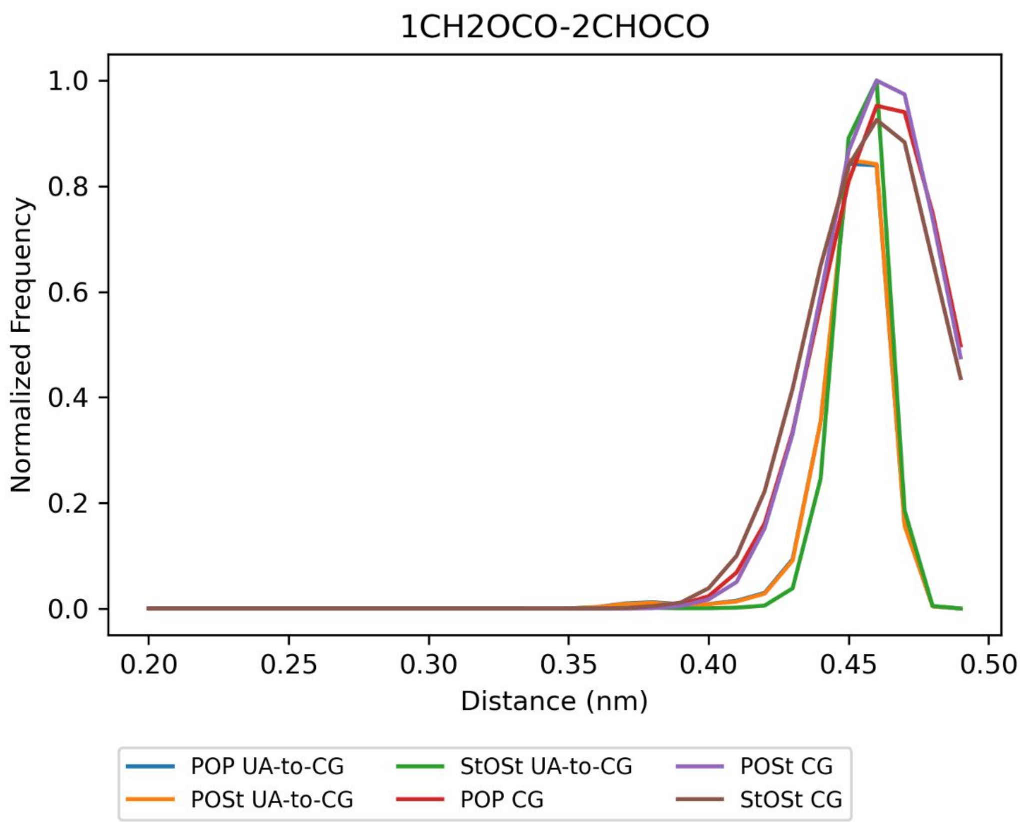

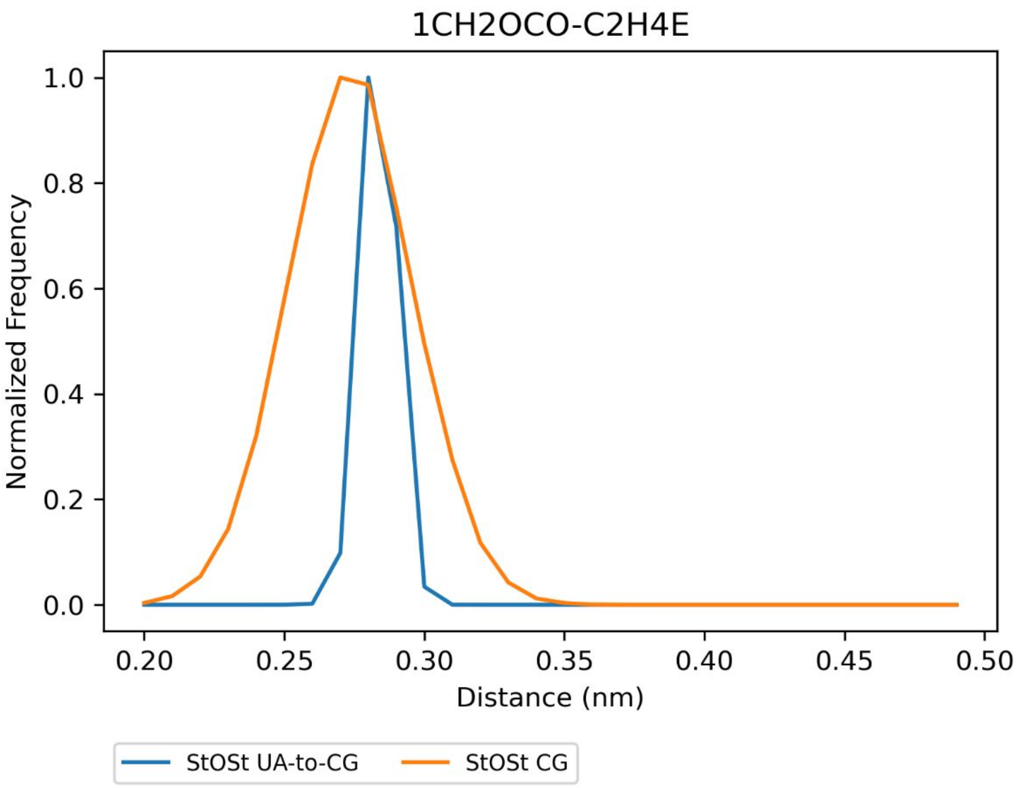
1CH2OCO-C2H4E
Normalized Frequency
Distance (nm)
StOSt UA-to-CG
StOSt CG

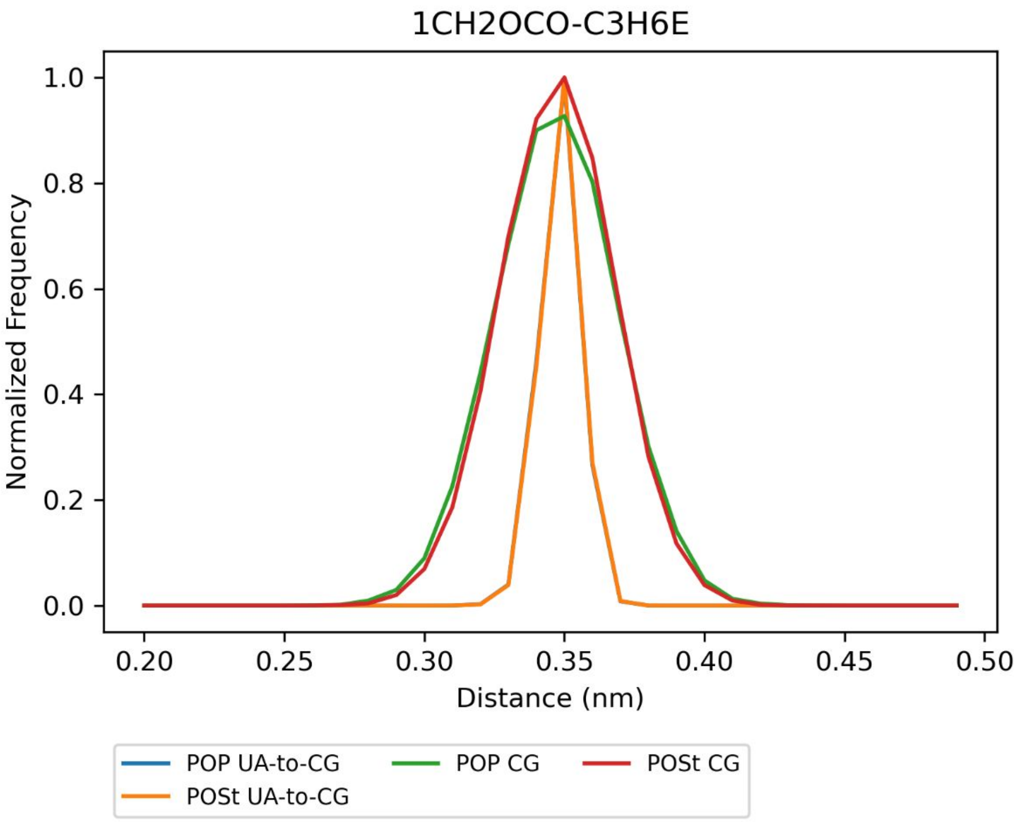
1CH2OCO-C3H6E
Normalized Frequency
Distance (nm)
POP UA-to-CG
POSt UA-to-CG
POP CG
POSt CG

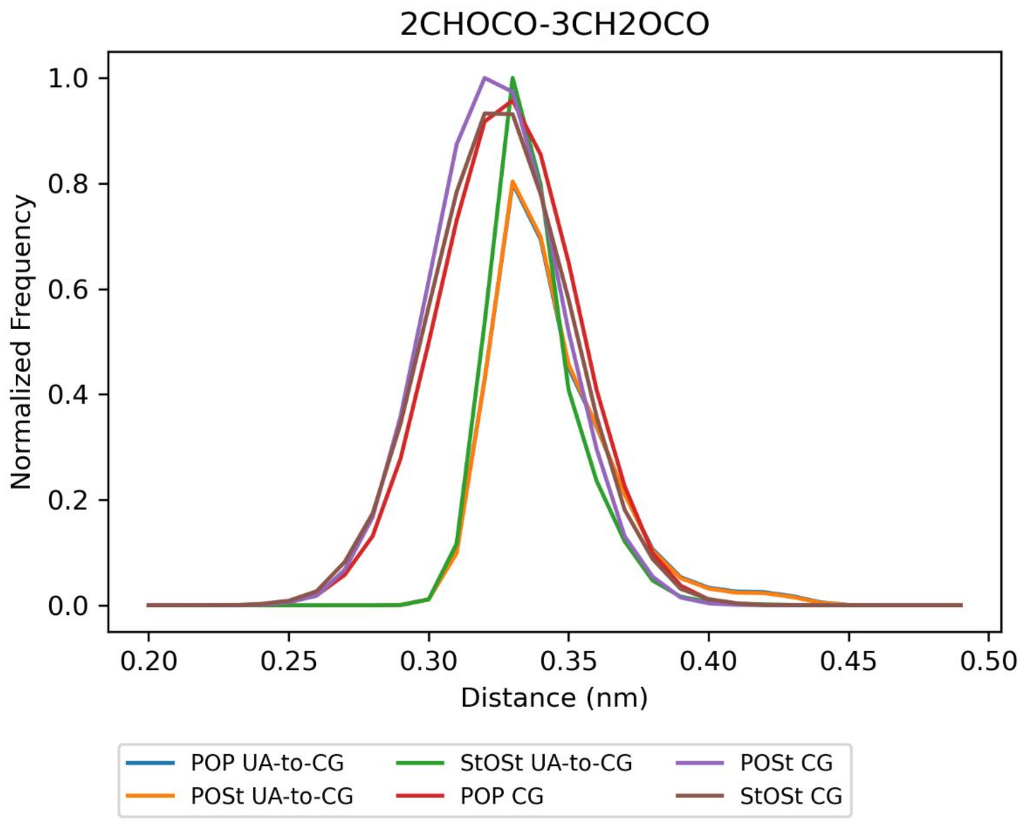
2CHOCO-3CH2OCO
Normalized Frequency
Distance (nm)
0.0
0.2
0.4
0.6
0.8
1.0
0.20
0.25
0.30
0.35
0.40
0.45
0.50
POP UA-to-CG
POSt UA-to-CG
StOSt UA-to-CG
POP CG
POSt CG
StOSt CG

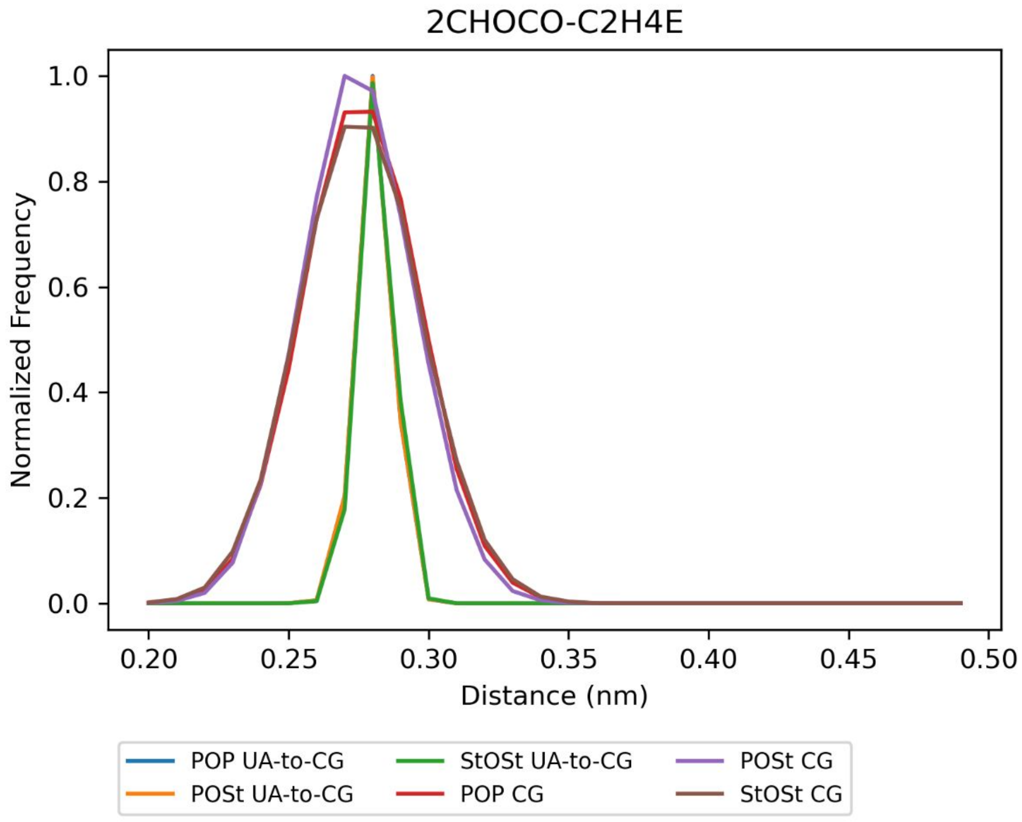
2CHOCO-C2H4E
Normalized Frequency
Distance (nm)
0.0
0.2
0.4
0.6
0.8
1.0
0.20
0.25
0.30
0.35
0.40
0.45
0.50
POP UA-to-CG
POSt UA-to-CG
StOSt UA-to-CG
POP CG
POSt CG
StOSt CG

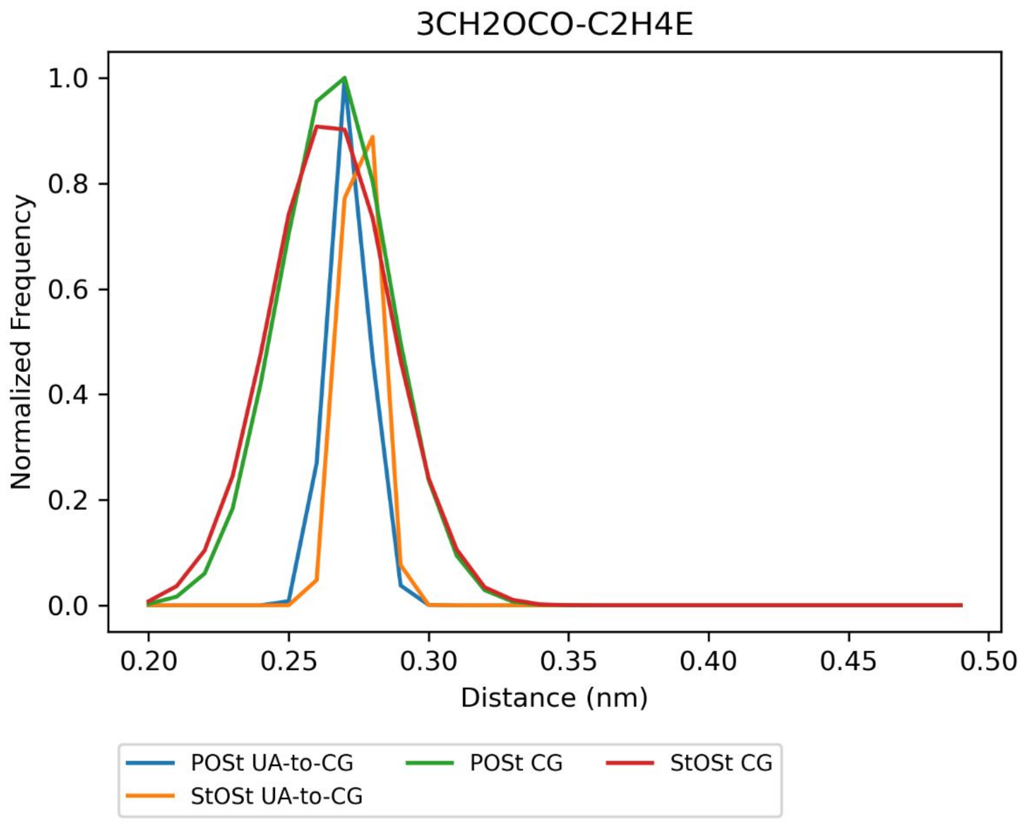

3CH2OCO-C2H4E
Normalized Frequency
Distance (nm)
0.0
0.2
0.4
0.6
0.8
1.0
0.20
0.25
0.30
0.35
0.40
0.45
0.50
POSt UA-to-CG
POSt CG
StOSt CG
StOSt UA-to-CG

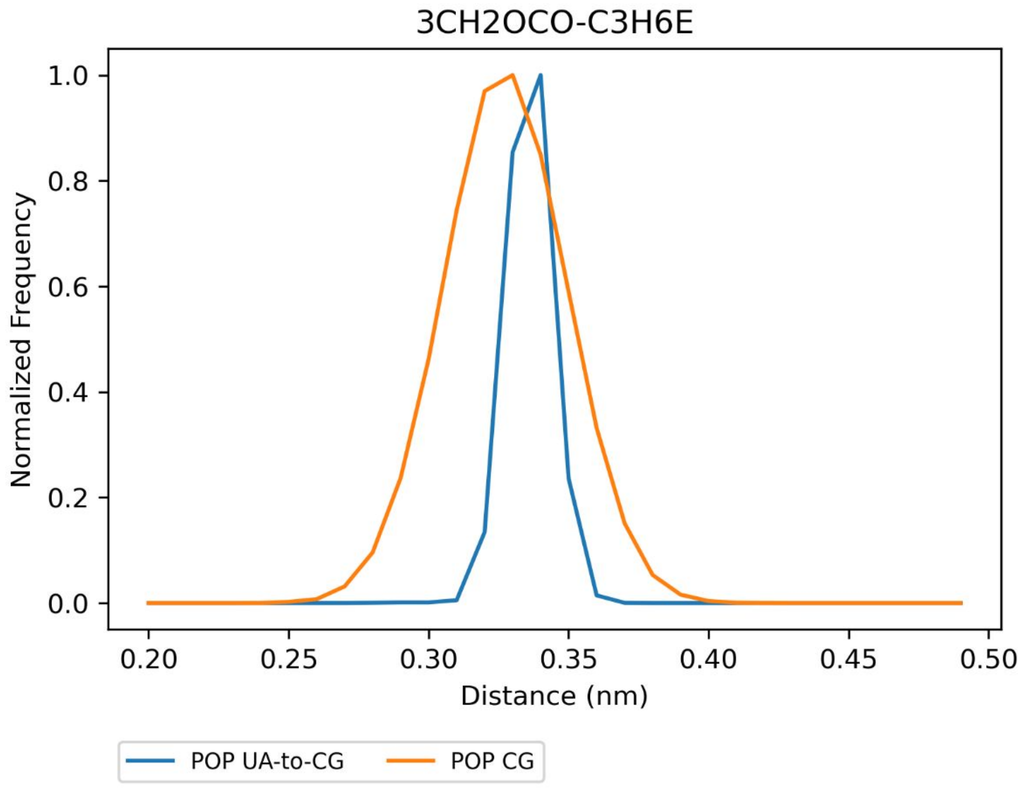

3CH2OCO-C3H6E
Normalized Frequency
Distance (nm)
0.0
0.2
0.4
0.6
0.8
1.0
0.20
0.25
0.30
0.35
0.40
0.45
0.50
POP UA-to-CG
POP CG

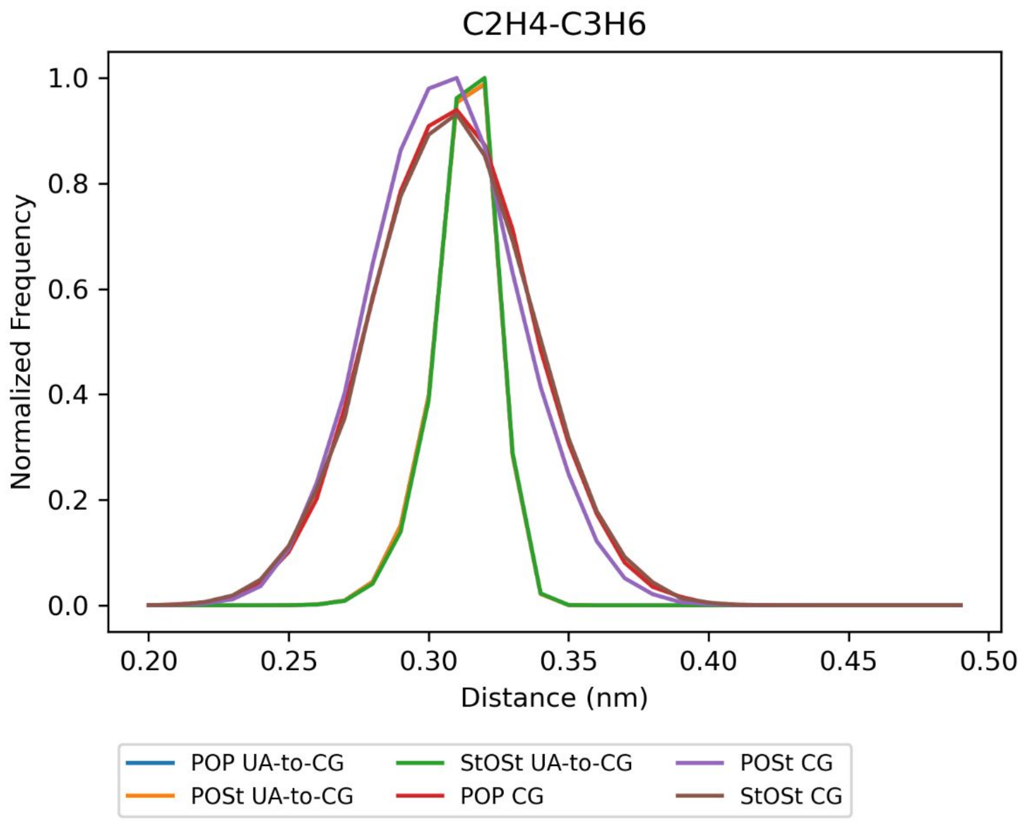

C2H4-C3H6
Normalized Frequency
Distance (nm)
POP UA-to-CG
POSt UA-to-CG
StOSt UA-to-CG
POP CG
POSt CG
StOSt CG

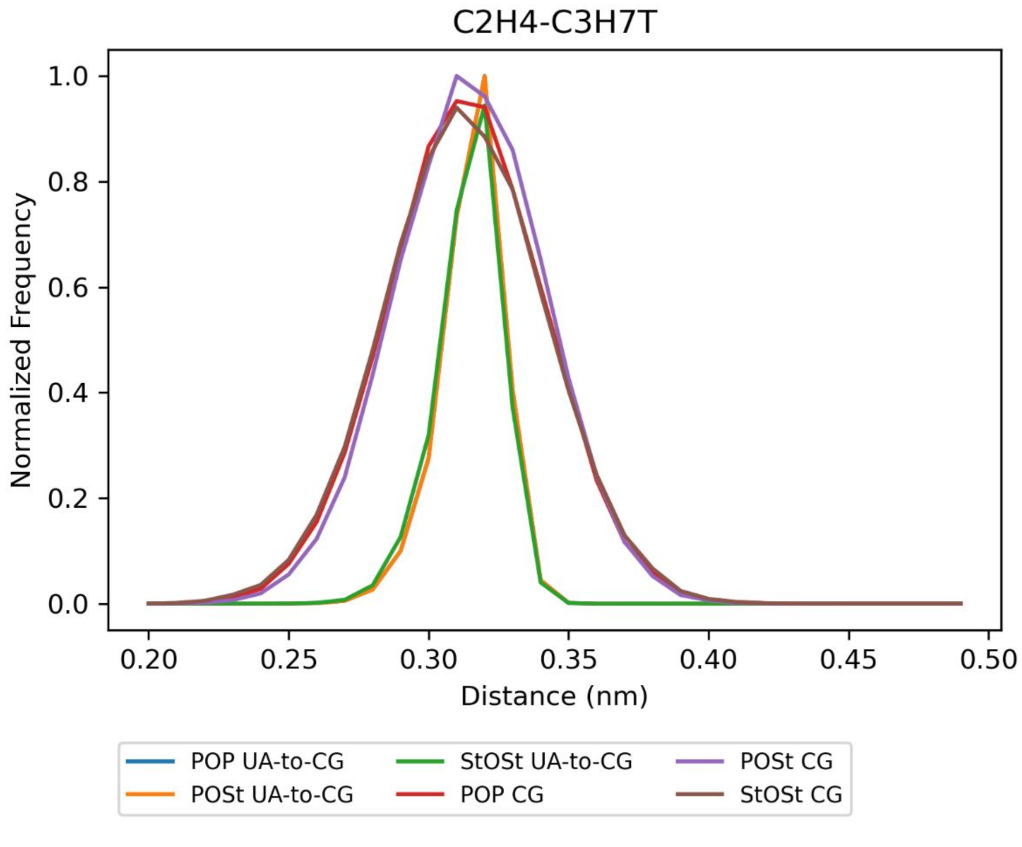

C2H4-C3H7T
Normalized Frequency
Distance (nm)
POP UA-to-CG
POSt UA-to-CG
StOSt UA-to-CG
POP CG
POSt CG
StOSt CG

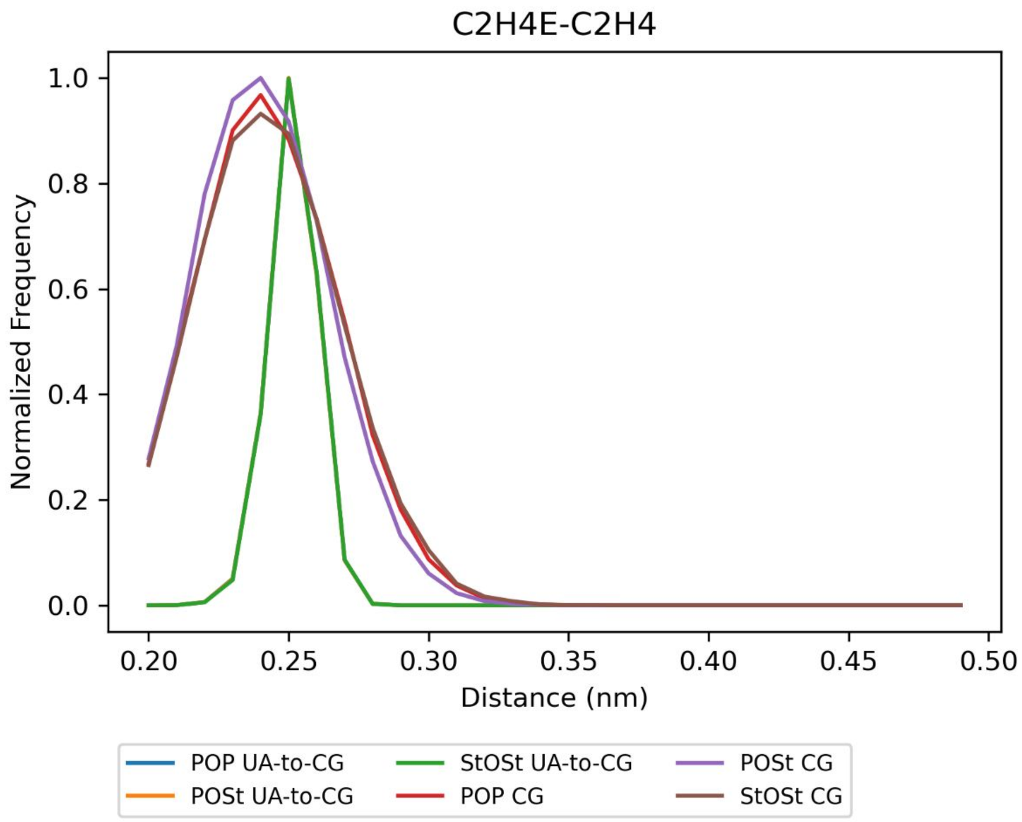
C2H4E-C2H4
Normalized Frequency
Distance (nm)
0.0
0.2
0.4
0.6
0.8
1.0
0.20
0.25
0.30
0.35
0.40
0.45
0.50
POP UA-to-CG
POSt UA-to-CG
StOSt UA-to-CG
POP CG
POSt CG
StOSt CG

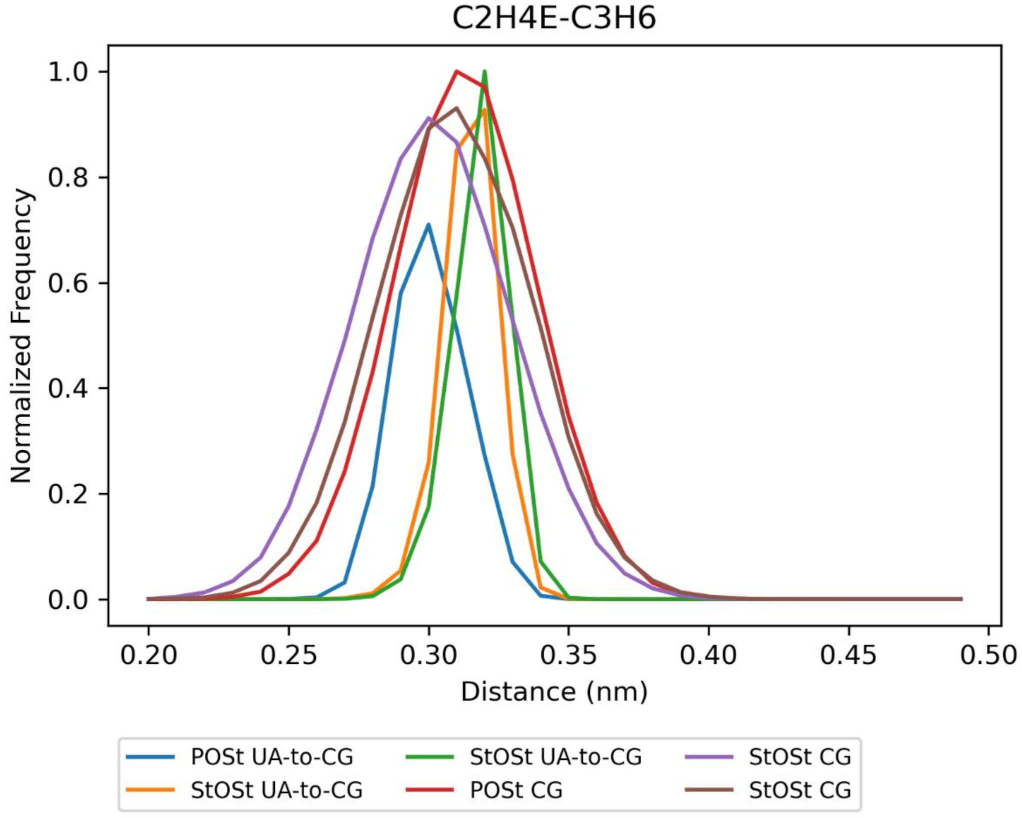
C2H4E-C3H6
Normalized Frequency
Distance (nm)
0.0
0.2
0.4
0.6
0.8
1.0
0.20
0.25
0.30
0.35
0.40
0.45
0.50
POSt UA-to-CG
StOSt UA-to-CG
StOSt UA-to-CG
POSt CG
StOSt CG
StOSt CG

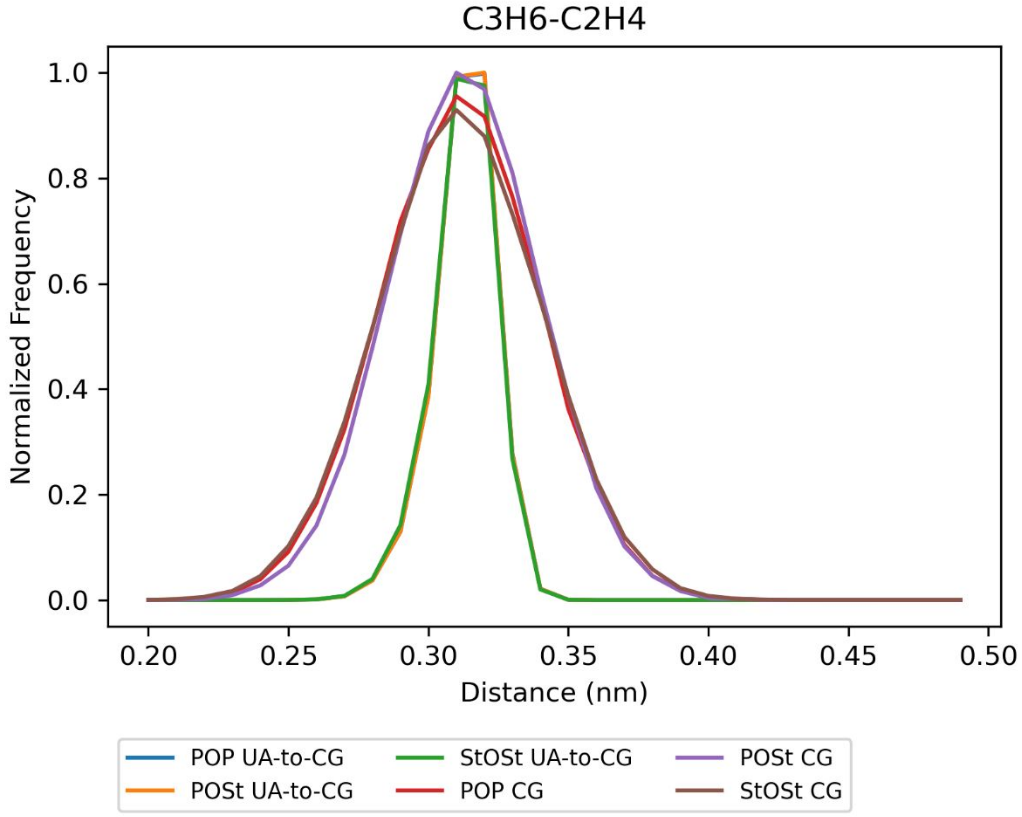
C3H6-C2H4
Normalized Frequency
Distance (nm)
0.0
0.2
0.4
0.6
0.8
1.0
0.20
0.25
0.30
0.35
0.40
0.45
0.50
POP UA-to-CG
POSt UA-to-CG
StOSt UA-to-CG
POP CG
POSt CG
StOSt CG

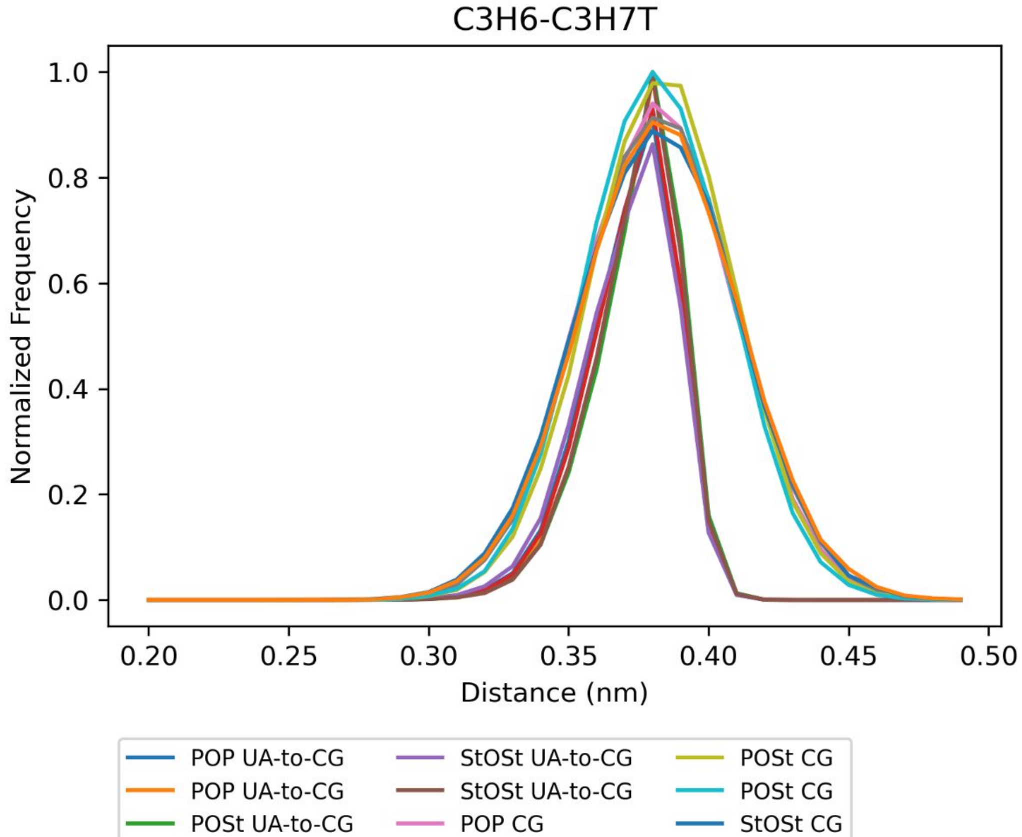
C3H6-C3H7T
Normalized Frequency
Distance (nm)
0.0
0.2
0.4
0.6
0.8
1.0
0.20
0.25
0.30
0.35
0.40
0.45
0.50
POP UA-to-CG
POP UA-to-CG
POSt UA-to-CG
POSt UA-to-CG
StOSt UA-to-CG
StOSt UA-to-CG
POP CG
POP CG
POSt CG
POSt CG
StOSt CG
StOSt CG

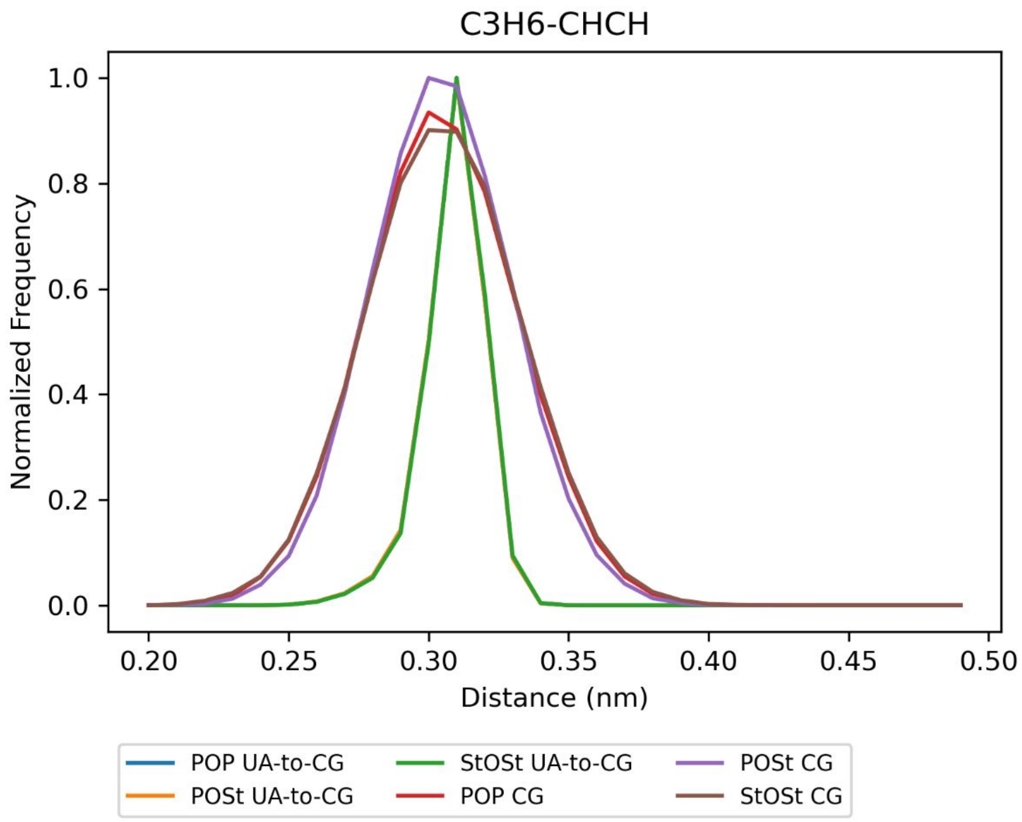
C3H6-CHCH
Normalized Frequency
1.0
0.8
0.6
0.4
0.2
0.0
0.20
0.25
0.30
0.35
0.40
0.45
0.50
Distance (nm)
POP UA-to-CG
POSt UA-to-CG
StOSt UA-to-CG
POP CG
POSt CG
StOSt CG

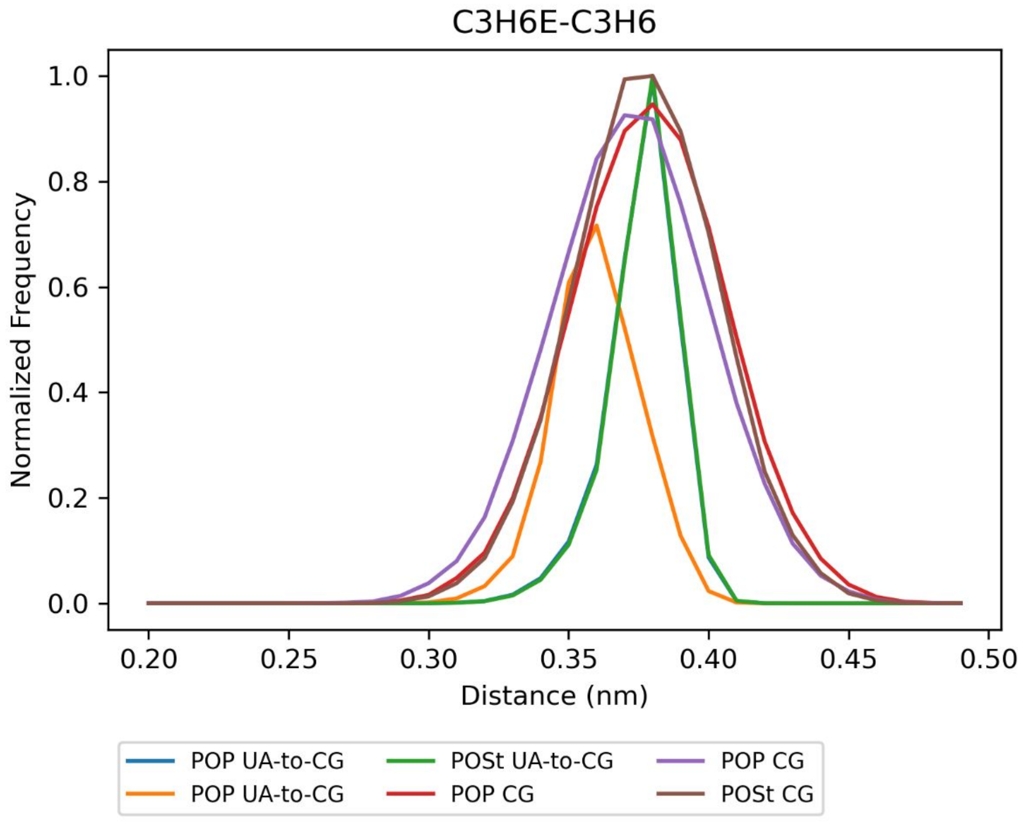
C3H6E-C3H6
Normalized Frequency
1.0
0.8
0.6
0.4
0.2
0.0
0.20
0.25
0.30
0.35
0.40
0.45
0.50
Distance (nm)
POP UA-to-CG
POP UA-to-CG
POSt UA-to-CG
POP CG
POP CG
POSt CG

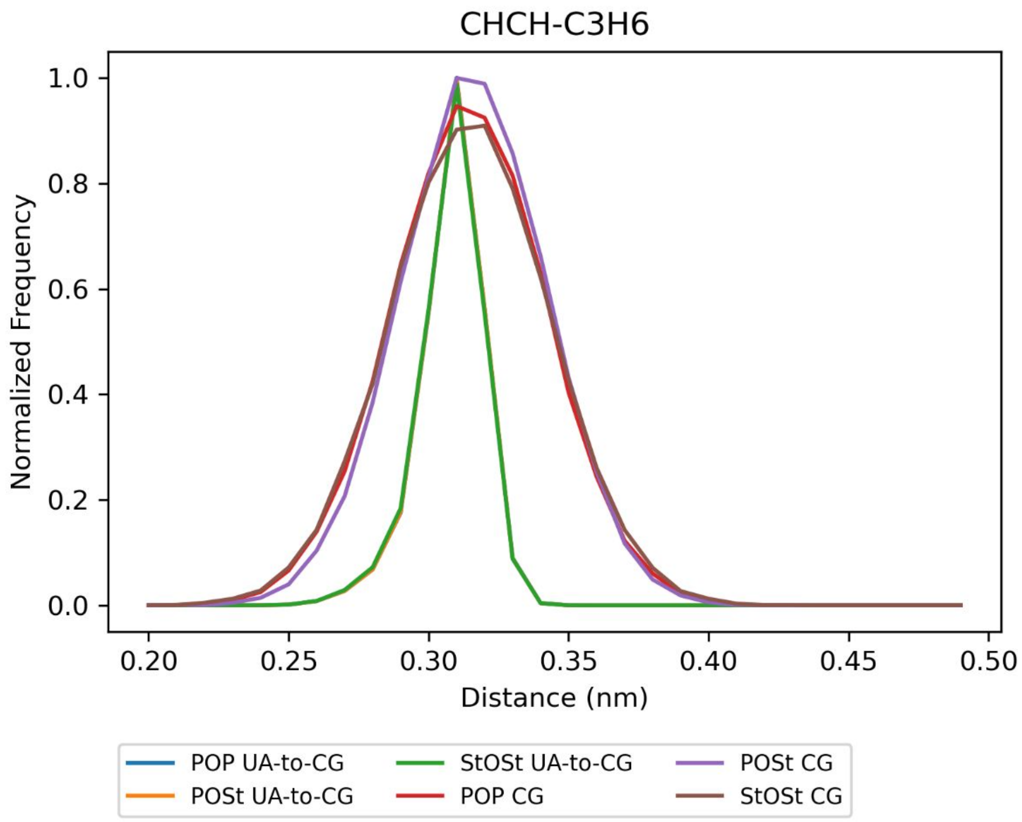

CHCH-C3H6
Normalized Frequency
Distance (nm)
0.0
0.2
0.4
0.6
0.8
1.0
0.20
0.25
0.30
0.35
0.40
0.45
0.50
POP UA-to-CG
POSt UA-to-CG
StOSt UA-to-CG
POP CG
POSt CG
StOSt CG

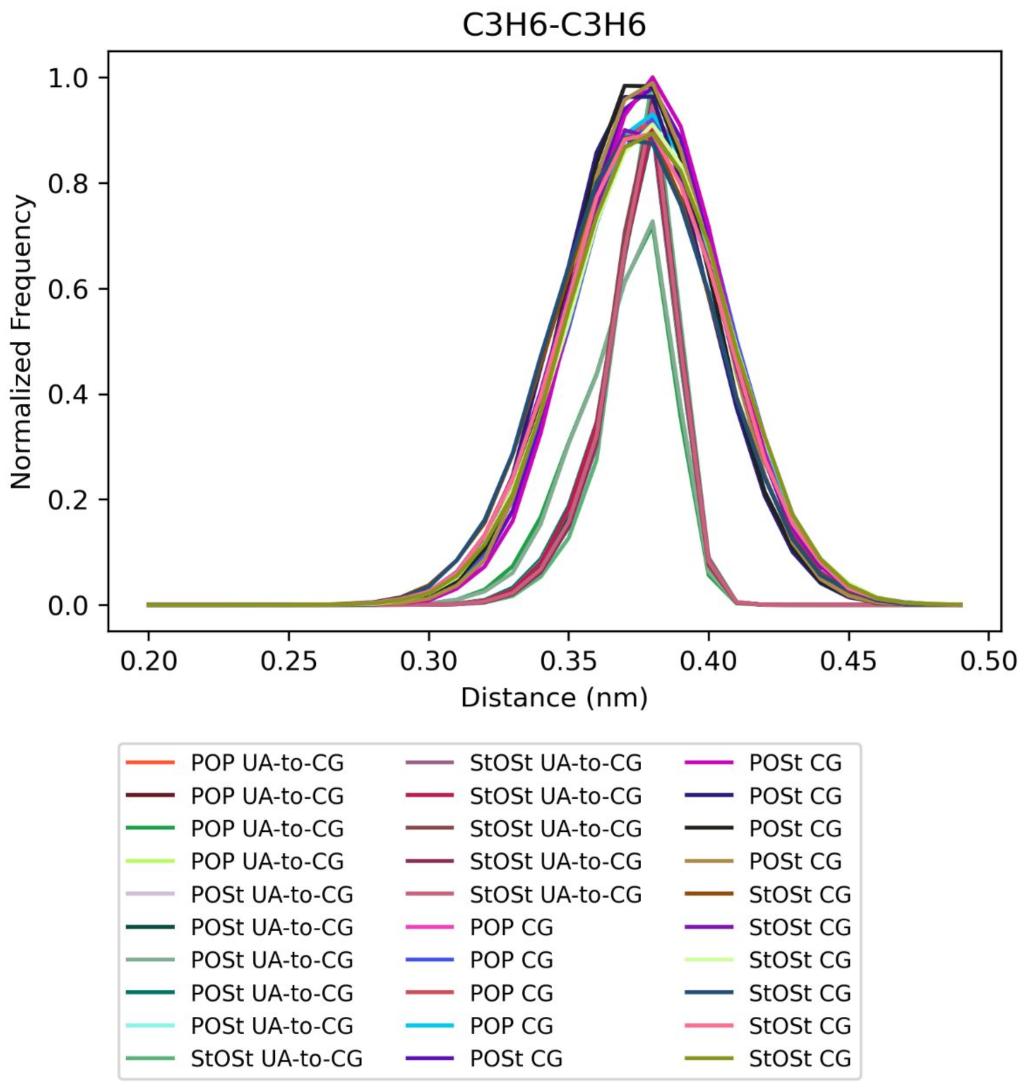

C3H6-C3H6
Normalized Frequency
1.0
0.8
0.6
0.4
0.2
0.0
0.20
0.25
0.30
0.35
0.40
0.45
0.50
Distance (nm)
POP UA-to-CG
POP UA-to-CG
POP UA-to-CG
POP UA-to-CG
POSt UA-to-CG
POSt UA-to-CG
POSt UA-to-CG
POSt UA-to-CG
POSt UA-to-CG
StOSt UA-to-CG
StOSt UA-to-CG
StOSt UA-to-CG
StOSt UA-to-CG
StOSt UA-to-CG
StOSt UA-to-CG
POP CG
POP CG
POP CG
POP CG
POSt CG
POSt CG
POSt CG
POSt CG
POSt CG
StOSt CG
StOSt CG
StOSt CG
StOSt CG
StOSt CG
StOSt CG

SI.4 Bayesian Optimization Metric Reference Values, Weightings and Equilibration Temperatures

Each iteration of the Bayesian Optimization consisted of nine different simulations, namely an equilibration of β_1 and β_2 crystals, and a melt, of each of *sn*-POP, *sn*-POSt and *sn*-StOSt. Each of these simulations was carried out at the respective temperature shown in the table below.

After equilibration, the respective metrics from each of the simulations (lattice dimensions and angle, density, and various conformations) were extracted as listed below (the metrics extracted from the respective simulations are highlighted in the same colour for easy reference). A cost was then calculated (using Equation 2 – see main text) by comparing the extracted data with the reference values listed below, and multiplying each by the respective weighting (also given below). The Scaled Weighting is given only for easy comparison (the sum of the scaled weightings = 1).

Metric	*Crystal/ Melt*	*TAG*	*Reference Value*	*Weighting*	*Scaled Weighting*	*Units*	*Comment*	*Simulation temperature (K)*
a_dimension	Beta_1	*sn*-POSt	2.7225	0.333	0.003581	nm	Crystalline box dimensions and angles	295
b_dimension			12.5976	0.333	0.003581	nm		
c_dimension			4.0968	0.333	0.003581	nm		
beta_angle			88.79	0.5	0.005377	nm		
a_dimension	Beta_2		2.71215	0.333	0.003581	nm		250
b_dimension			12.6529	0.333	0.003581	nm		
c_dimension			4.05895	0.333	0.003581	nm		
beta_angle			88.51	0.5	0.005377	nm		
a_dimension	Beta_1	*sn*-POP	2.725	0.333	0.003581	nm		295
b_dimension			12.1319	0.333	0.003581	nm		
c_dimension			4.104477	0.333	0.003581	nm		
beta_angle			88.85	0.5	0.005377	nm		
a_dimension	Beta_2		2.72355	0.333	0.003581	nm		280
b_dimension			12.1622	0.333	0.003581	nm		
c_dimension			4.1097815	0.333	0.003581	nm		
beta_angle			88.78	0.5	0.005377	nm		
a_dimension	Beta_1	*sn*-StOSt	2.72115	0.333	0.003581	nm		295
b_dimension			12.9903	0.333	0.003581	nm		

Metric	*Crystal/ Melt*	*TAG*	*Reference Value*	*Weighting*	*Scaled Weighting*	*Units*	*Comment*	*Simulation temperature (K)*
c_dimension			4.0918365	0.333	0.003581	nm		
beta_angle			88.71	0.5	0.005377	nm		
a_dimension	Beta_2		2.72005	0.333	0.003581	nm		298
b_dimension			13.0295	0.333	0.003581	nm		
c_dimension			4.110428	0.333	0.003581	nm		
beta_angle			88.75	0.5	0.005377	nm		
density	Beta_1	*sn*-POSt	1018.028931	2	0.021507	kg/m^3	-	295
density	Beta_2		1026.935303	2	0.021507	kg/m^3		250
density	melt		888.7	2	0.021507	kg/m^3		313.15
density	Beta_1	*sn*-POP	1020	2	0.021507	kg/m^3		295
density	Beta_2		1017	2	0.021507	kg/m^3		280
density	melt		884.9	2	0.021507	kg/m^3		313.15
density	Beta_1	*sn*-StOSt	1021	2	0.021507	kg/m^3		295
density	Beta_2		1014	2	0.021507	kg/m^3		298
density	melt		885.3	2	0.021507	kg/m^3		313.15
Average_O-P/S_rotation_final_frame	Beta_1	*sn*-POSt	360	3	0.03226	°	(1) Determining the rotation between the *sn*-1 and *sn*-3 plane and the *sn*-2 plane at the end of equilibration	295
Average_O(final)-O(initial)_rotation			360	1.5	0.01613	°	(2) Determining the change in orientation in space of the *sn*-2 plane between the start and end of equilibration	
Average_P/S(final)-P/S(initial)_rotation			360	1.5	0.01613	°	(3) Determining the change in orientation in space of the *sn*-1/*sn*-3 plane between the start and end of equilibration	
Average_O-P/S_rotation_final_frame	Beta_2		360	3	0.03226	°	(1)	250
Average_O(final)-O(initial)_rotation			360	1.5	0.01613	°	(2)	

Metric	*Crystal/ Melt*	*TAG*	*Reference Value*	*Weighting*	*Scaled Weighting*	*Units*	*Comment*	*Simulation temperature (K)*
Average_P/S(final)-P/S(initial)_rotation			360	1.5	0.01613	°	(3)	
Average_O-P/P_rotation_final_frame	Beta_1	*sn*-POP	360	3	0.03226	°	(1)	295
Average_O(final)-O(initial)_rotation			360	1.5	0.01613	°	(2)	
Average_P/P(final)-P/P(initial)_rotation			360	1.5	0.01613	°	(3)	
Average_O-P/P_rotation_final_frame	Beta_2		360	3	0.03226	°	(1)	280
Average_O(final)-O(initial)_rotation			360	1.5	0.01613	°	(2)	
Average_P/P(final)-P/P(initial)_rotation			360	1.5	0.01613	°	(3)	
Average_O-S/S_rotation_final_frame	Beta_1	*sn*-StOSt	360	3	0.03226	°	(1)	295
Average_O(final)-O(initial)_rotation			360	1.5	0.01613	°	(2)	
Average_S/S(final)-S/S(initial)_rotation			360	1.5	0.01613	°	(3)	
Average_O-S/S_rotation_final_frame	Beta_2		360	3	0.03226	°	(1)	298
Average_O(final)-O(initial)_rotation			360	1.5	0.01613	°	(2)	
Average_S/S(final)-S/S(initial)_rotation			360	1.5	0.01613	°	(3)	
C3H7T-2CHOCO-CHCH_(P6-O1-O5)	Beta_1	*sn*-POSt	177.22	5	0.053767	°	(4) "Artificial" long-range bonded angle to determine the linearity of the *sn*-1 and *sn*-2 chains	295
C3H7T-2CHOCO-CHCH_(P6-O1-O5)	Beta_2		176.51	5	0.053767	°	(4)	250
C3H7T-2CHOCO-CHCH_(P6-O1-O5)	Beta_1	*sn*-POP	177.06	5	0.053767	°	(4)	295
C3H7T-2CHOCO-CHCH_(P6-O1-O5)	Beta_2		179.5	5	0.053767	°	(4)	280

Metric	*Crystal/ Melt*	*TAG*	*Reference Value*	*Weighting*	*Scaled Weighting*	*Units*	*Comment*	*Simulation temperature (K)*
C3H7T-2CHOCO-CHCH_(S7-O1-O5)	Beta_1	*sn*-StOSt	177.08	5	0.053767	°	(4)	295
C3H7T-2CHOCO-CHCH_(S7-O1-O5)	Beta_2		174.87	5	0.053767	°	(4)	298

SI.5 Chosen Lennard-Jones parameter ranges for the Bayesian Optimization

Parameter	*Bead*	*Minimum*	*Maximum*
	1CH2OCO	0.3	0.6
	2CHOCO	0.3	0.6
	3CH2OCO	0.3	0.6
	CHCH	0.2	0.5
σ (nm)	C2H4E	0.2	0.5
	C2H4	0.2	0.5
	C3H6E	0.25	0.55
	C3H6	0.25	0.55
	C3H7T	0.25	0.55
	1CH2OCO	2	6
	2CHOCO	3	8
	3CH2OCO	2	6
	CHCH	1	4
ε (kJ/mol)	C2H4E	1	4
	C2H4	1	4
	C3H6E	1	4
	C3H6	1	4
	C3H7T	1	4

Ranges for σ were chosen based on previous work by Braseillo *et al.*[1] as well as size of beads.

Ranges for ε were chosen based on previous work by Braseillo *et al.*[1]

SI.6 Full set of simulated macroscopic properties of all TAGs. All reported results are the average of three runs.

SI.6.1 Empirical and simulated crystalline densities

TAG	*Polymorph*	*Temperature (K)*	*Empirical Density (g/cm³)*	*Simulated Density (g/cm³)*	*Simulated Density Range (g/cm³)*	*% Difference*
sn-MMM	β	293	1.038	0.998	0.994-1.003	-3.85
sn-SSS	β	293	1.028	0.940	0.938-0.945	-8.56
sn-POSt	β_1	295	1.018	0.968	0.965-0.969	-4.95
sn-POSt	β_2	250	1.026	0.984	0.984-0.985	-4.05
sn-StOSt	β_1	295	1.021	0.968	0.968-0.968	-5.17
sn-StOSt	β_2	298	1.014	0.963	0.956-0.967	-5.02
sn-POP	β_1	295	1.020	0.977	0.972-0.979	-4.26
sn-POP	β_2	280	1.017	0.984	0.977-0.987	-3.28
sn-MOM	β_1	295	1.025	1.004	1.000-1.007	-2.01
sn-PPP	β	243	1.039	0.975	0.973-0.976	-6.13
sn-StOA	β_1	294	1.015	0.967	0.962-0.969	-4.73
sn-StOA	β_2	298	1.015	0.960	0.960-0.961	-5.38

SI.6.2 Empirical and simulated crystalline unit cell angles

TAG	*Phase*	*Temperature (K)*	*Empirical α (°)*	*Simulated α (°)*	*% Difference (α)*	*Empirical β (°)*	*Simulated β (°)*	*% Difference (β)*	*Empirical γ (°)*	*Simulated γ (°)*	*% Difference (γ)*
sn-MMM	β	293	73.39	72.97	-0.57	100.41	100.56	+0.15	118.27	117.83	-0.37
sn-SSS	β	293	73.75	74.18	+0.58	100.26	99.79	-0.47	117.69	116.95	-0.63
sn-POSt	$β_1$	295	90.00	90.00	< 0.01	88.79	88.86	+0.08	90.00	90.00	< 0.01
sn-POSt	$β_2$	250	90.00	90.00	< 0.01	88.51	88.56	+0.05	90.00	90.00	< 0.01
sn-StOSt	$β_1$	295	90.00	90.00	< 0.01	88.71	88.83	+0.14	90.00	90.00	< 0.01
sn-StOSt	$β_2$	298	90.00	90.00	< 0.01	88.75	88.83	+0.09	90.00	90.00	< 0.01
sn-POP	$β_1$	295	90.00	90.00	< 0.01	88.85	88.93	+0.09	90.00	90.00	< 0.01
sn-POP	$β_2$	280	90.00	90.00	< 0.01	88.78	88.87	+0.10	90.00	90.00	< 0.01
sn-MOM	$β_1$	295	90.00	90.00	< 0.01	88.84	88.93	+0.10	90.00	90.00	< 0.01
sn-PPP	β	243	84.66	84.37	-0.34	86.97	86.95	-0.02	79.77	80.30	+0.66
sn-StOA	$β_1$	294	90.00	90.00	< 0.01	88.73	88.82	+0.10	90.00	90.00	< 0.01

sn-StOA	β_2	298	90.00	90.00	< 0.01	88.64	88.66	+0.03	90.00	90.00	< 0.01

SI.6.3 Simulated, interpolated and empirical melted densities (all values are ± 0.0005 g/cm^3)

TAG	*Simulated Density (g/cm^3) at 280 K*	*Simulated Density (g/cm^3) at 290 K*	*Simulated Density (g/cm^3) at 300 K*	*Simulated Density (g/cm^3) at 310 K*	*Simulated Density (g/cm^3) at 320 K*	*Simulated Density (g/cm^3) at 330 K*
sn-POSt	0.925	0.919	0.914	0.909	0.903	0.898
sn-StOSt	0.918	0.913	0.908	0.903	0.897	0.892
sn-POP	0.936	0.931	0.925	0.919	0.914	0.908
sn-OOO	0.958	0.952	0.947	0.942	0.936	0.931

TAG	*sn*-POSt	*sn*-StOSt	*sn*-POP	*sn*-OOO
Empirical density (g/cm³) *	0.902	0.906	0.904	0.916
Interpolated density (g/cm³) *	0.918	0.911	0.929	0.953
% Difference density (g/cm³) *	+1.72	+0.54	+2.76	+4.06
Empirical density (g/cm³) †	0.889	0.885	0.885	0.908
Interpolated density (g/cm³) †	0.907	0.901	0.918	0.948
% Difference density (g/cm³) †	+2.06	+1.76	+3.72	+4.43

* at 293.15 K for *sn*-POSt, *sn*-StOSt and *sn*-POP; at 288.15 K for *sn*-OOO
† at 313.15 K for *sn*-POSt, *sn*-StOSt and *sn*-POP; at 298.15 K for *sn*-OOO

SI.7 Radial Distribution Functions for all possible bead type combinations for the melt and crystal of *sn*-POSt

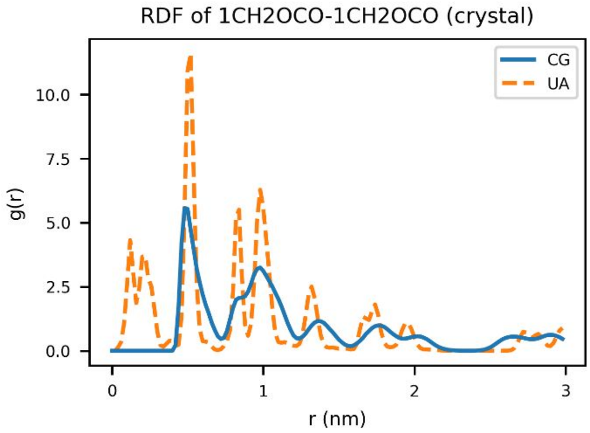

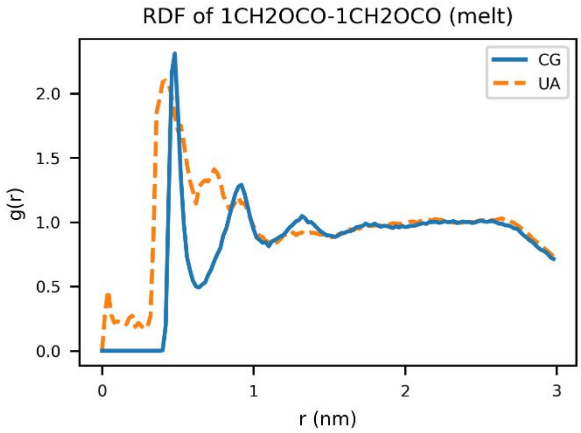

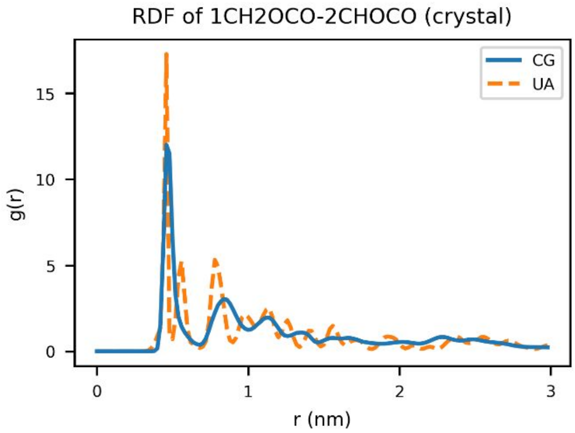

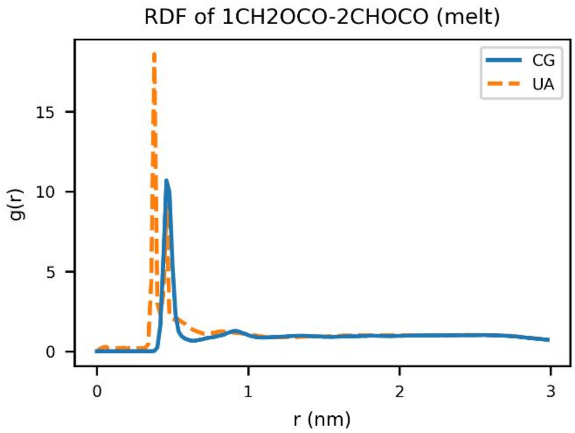

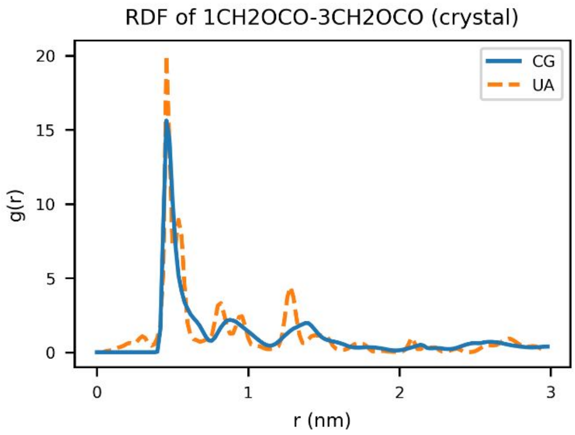

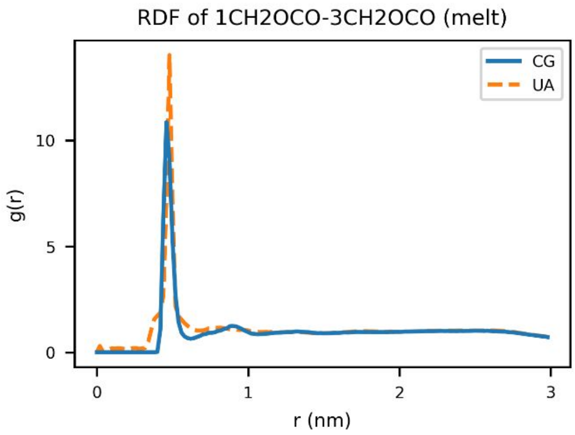

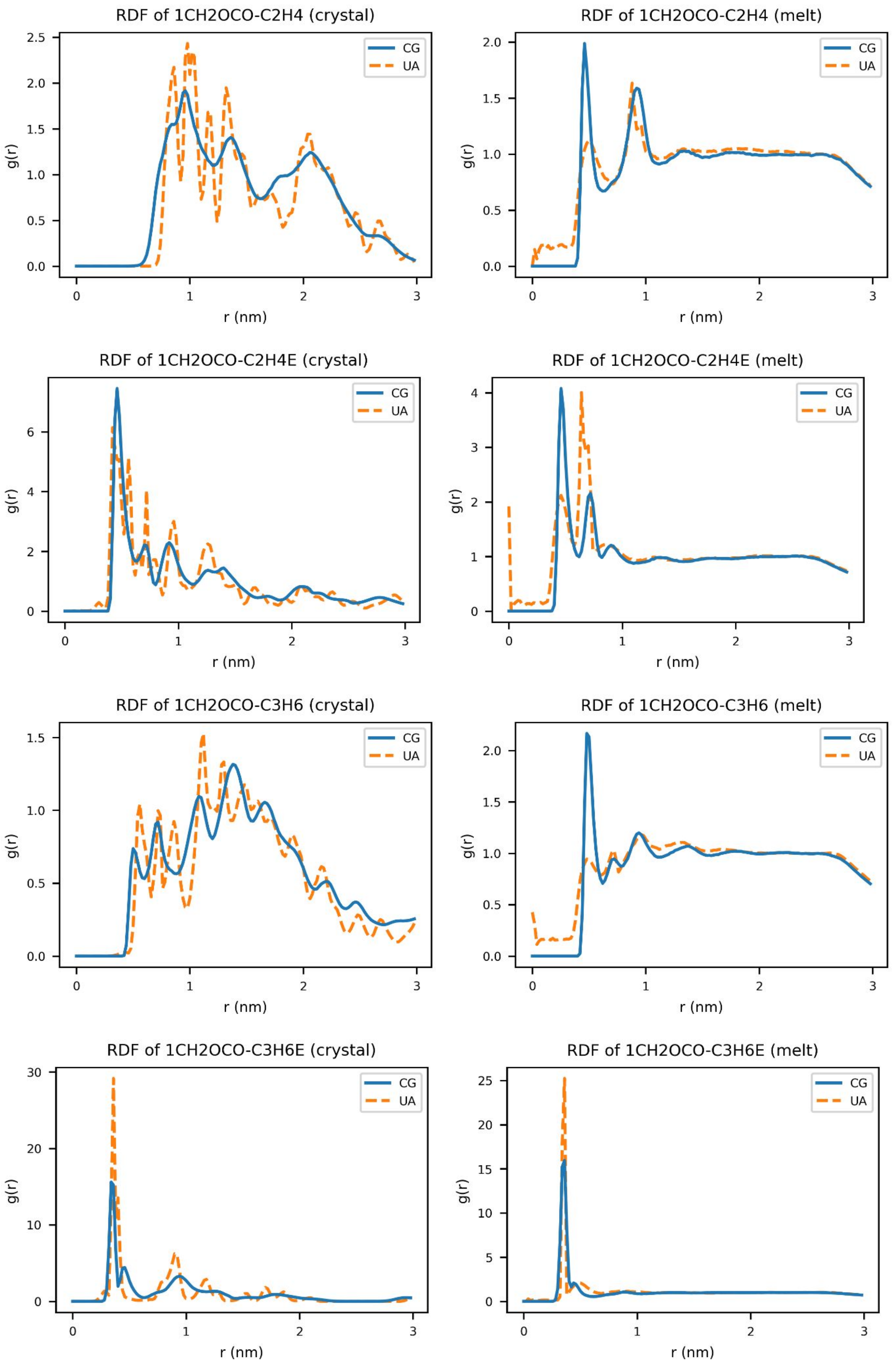
RDF of 1CH2OCO-C2H4 (crystal)
RDF of 1CH2OCO-C2H4 (melt)
RDF of 1CH2OCO-C2H4E (crystal)
RDF of 1CH2OCO-C2H4E (melt)
RDF of 1CH2OCO-C3H6 (crystal)
RDF of 1CH2OCO-C3H6 (melt)
RDF of 1CH2OCO-C3H6E (crystal)
RDF of 1CH2OCO-C3H6E (melt)
g(r)
r (nm)
CG
UA

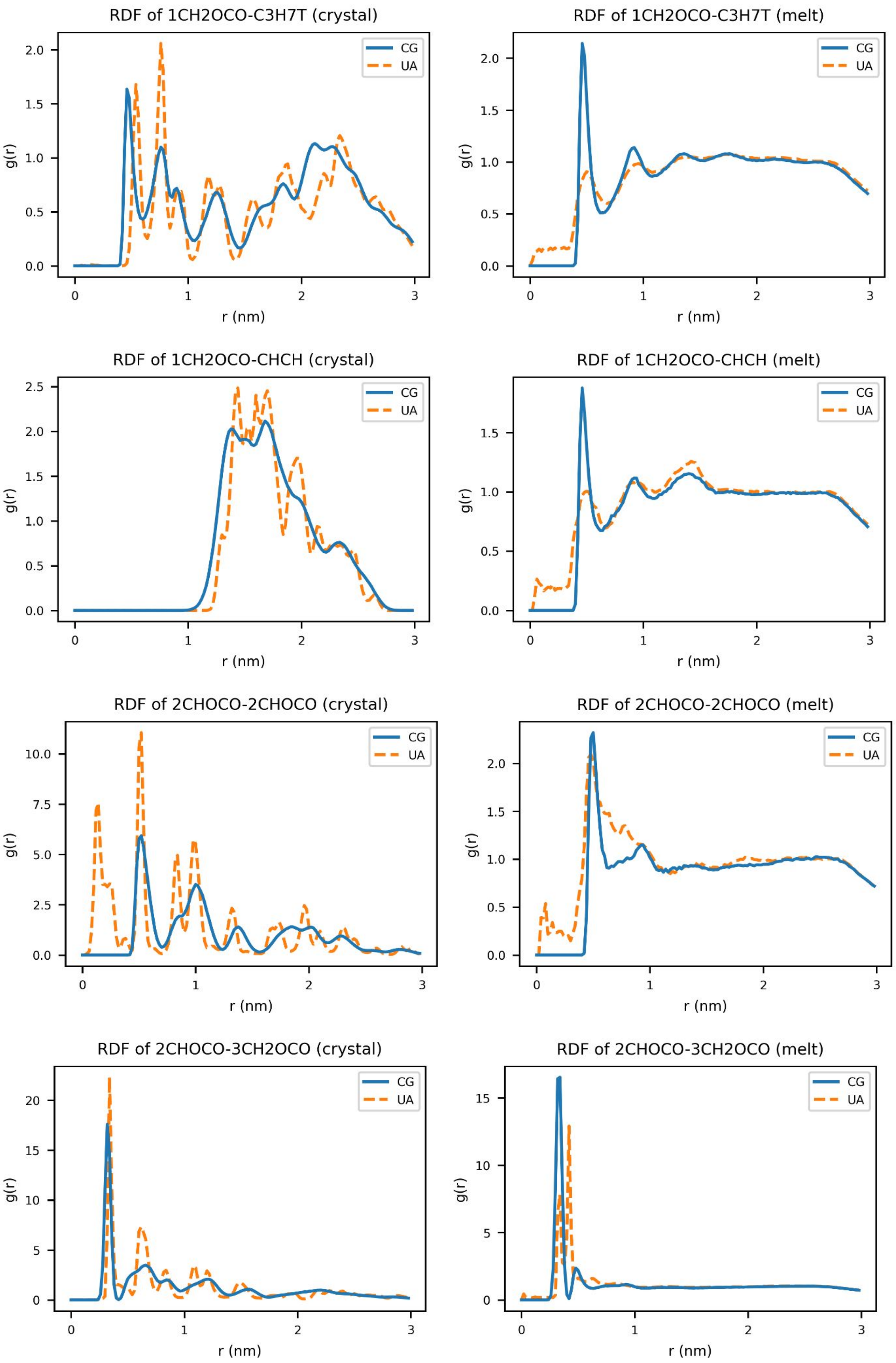
RDF of 1CH2OCO-C3H7T (crystal)
RDF of 1CH2OCO-C3H7T (melt)
RDF of 1CH2OCO-CHCH (crystal)
RDF of 1CH2OCO-CHCH (melt)
RDF of 2CHOCO-2CHOCO (crystal)
RDF of 2CHOCO-2CHOCO (melt)
RDF of 2CHOCO-3CH2OCO (crystal)
RDF of 2CHOCO-3CH2OCO (melt)
CG
UA
g(r)
r (nm)

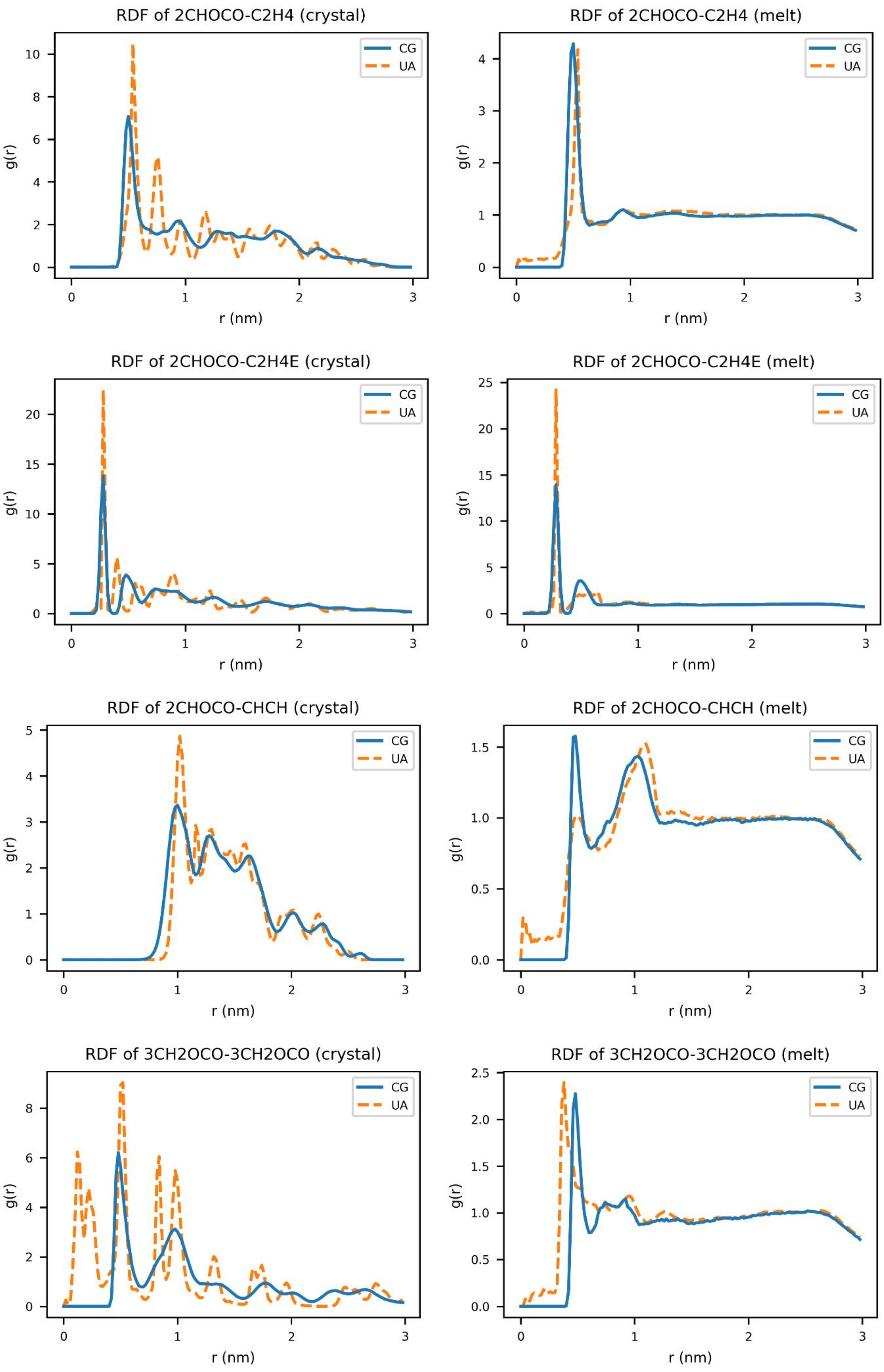
RDF of 2CHOCO-C2H4 (crystal)
RDF of 2CHOCO-C2H4 (melt)
RDF of 2CHOCO-C2H4E (crystal)
RDF of 2CHOCO-C2H4E (melt)
RDF of 2CHOCO-CHCH (crystal)
RDF of 2CHOCO-CHCH (melt)
RDF of 3CH2OCO-3CH2OCO (crystal)
RDF of 3CH2OCO-3CH2OCO (melt)
g(r)
r (nm)
CG
UA

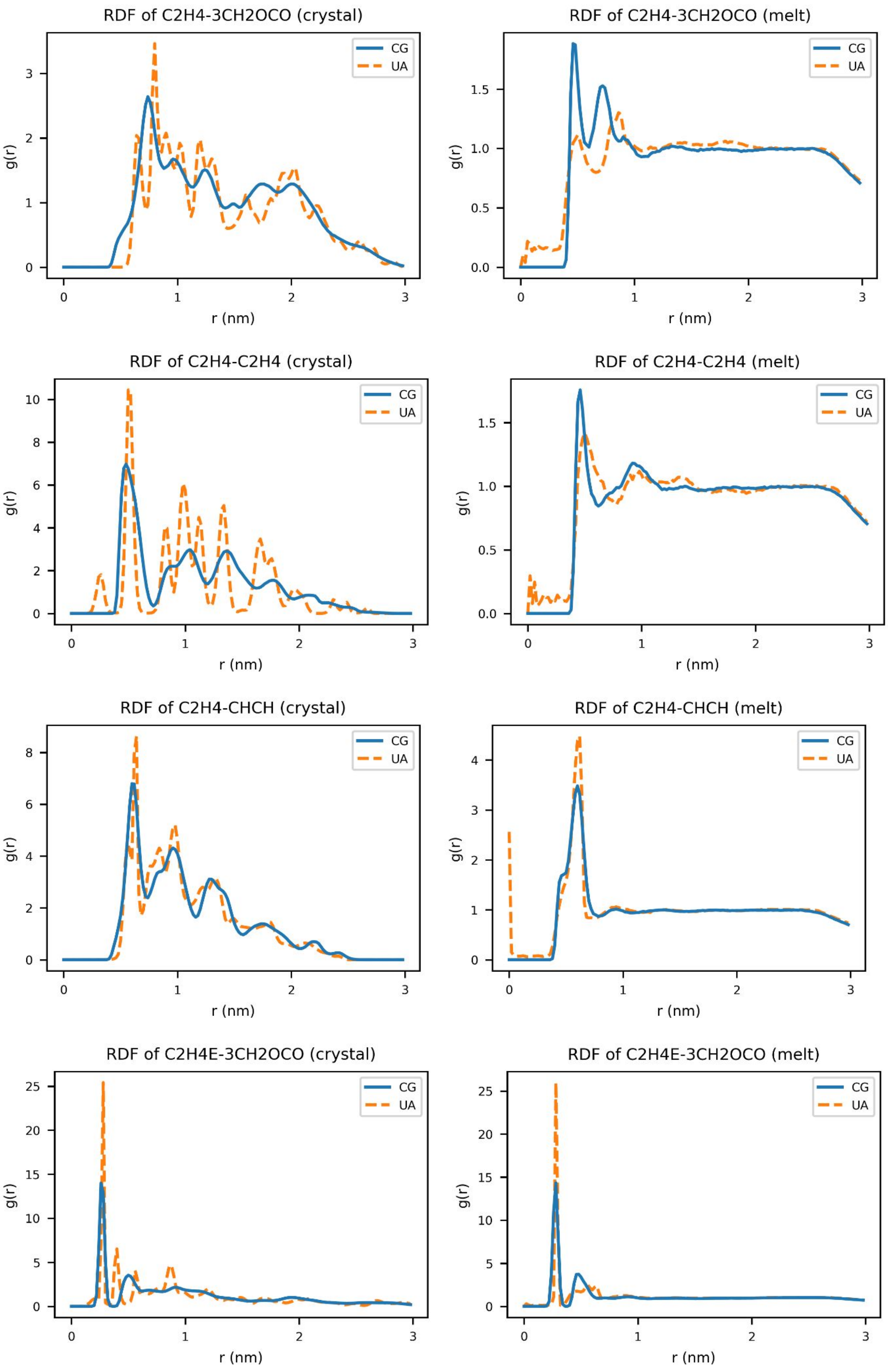

RDF of C2H4-3CH2OCO (crystal)
RDF of C2H4-3CH2OCO (melt)
RDF of C2H4-C2H4 (crystal)
RDF of C2H4-C2H4 (melt)
RDF of C2H4-CHCH (crystal)
RDF of C2H4-CHCH (melt)
RDF of C2H4E-3CH2OCO (crystal)
RDF of C2H4E-3CH2OCO (melt)
CG
UA
g(r)
r (nm)

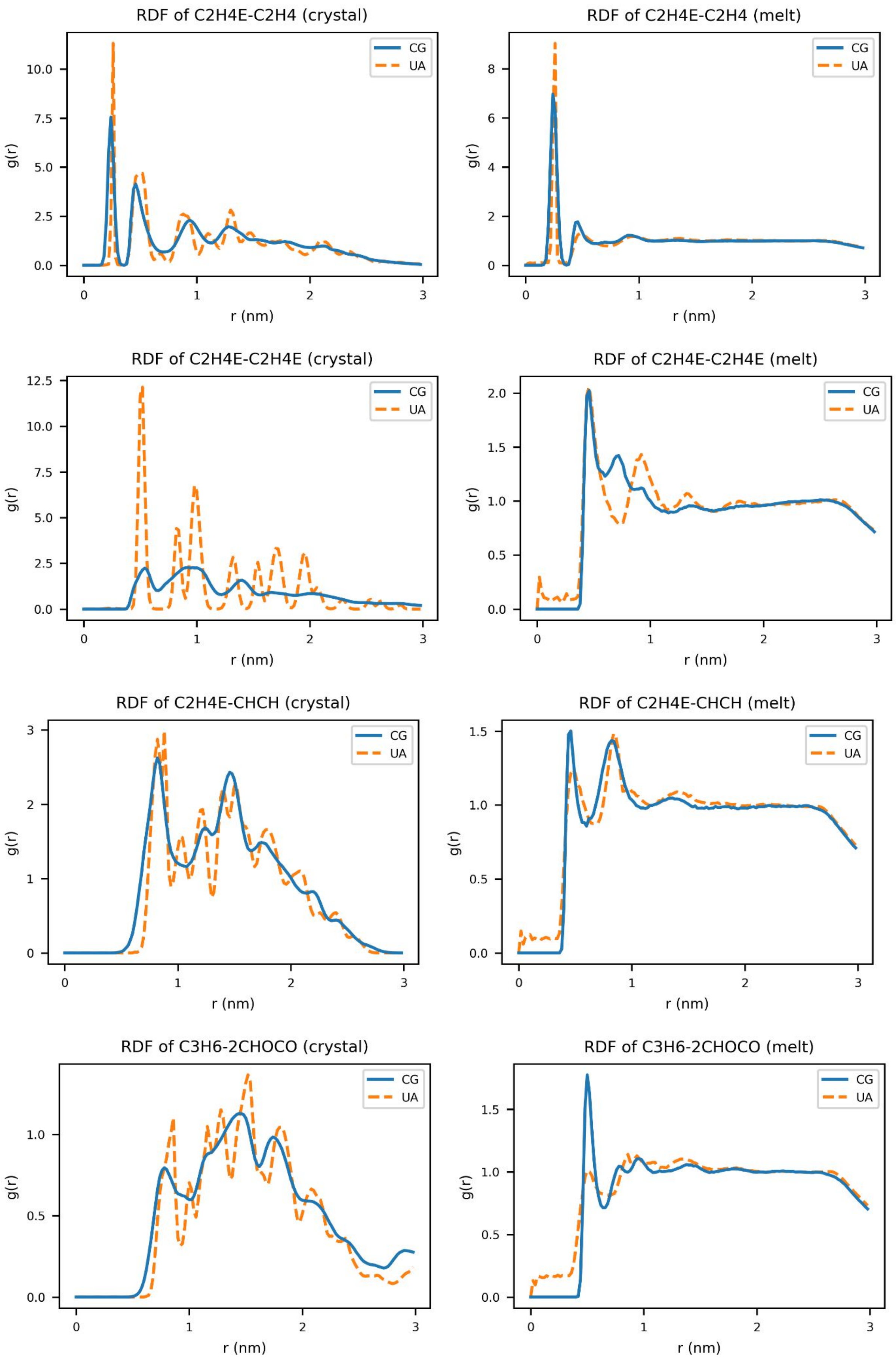
RDF of C2H4E-C2H4 (crystal)
RDF of C2H4E-C2H4 (melt)
RDF of C2H4E-C2H4E (crystal)
RDF of C2H4E-C2H4E (melt)
RDF of C2H4E-CHCH (crystal)
RDF of C2H4E-CHCH (melt)
RDF of C3H6-2CHOCO (crystal)
RDF of C3H6-2CHOCO (melt)
CG
UA
g(r)
r (nm)

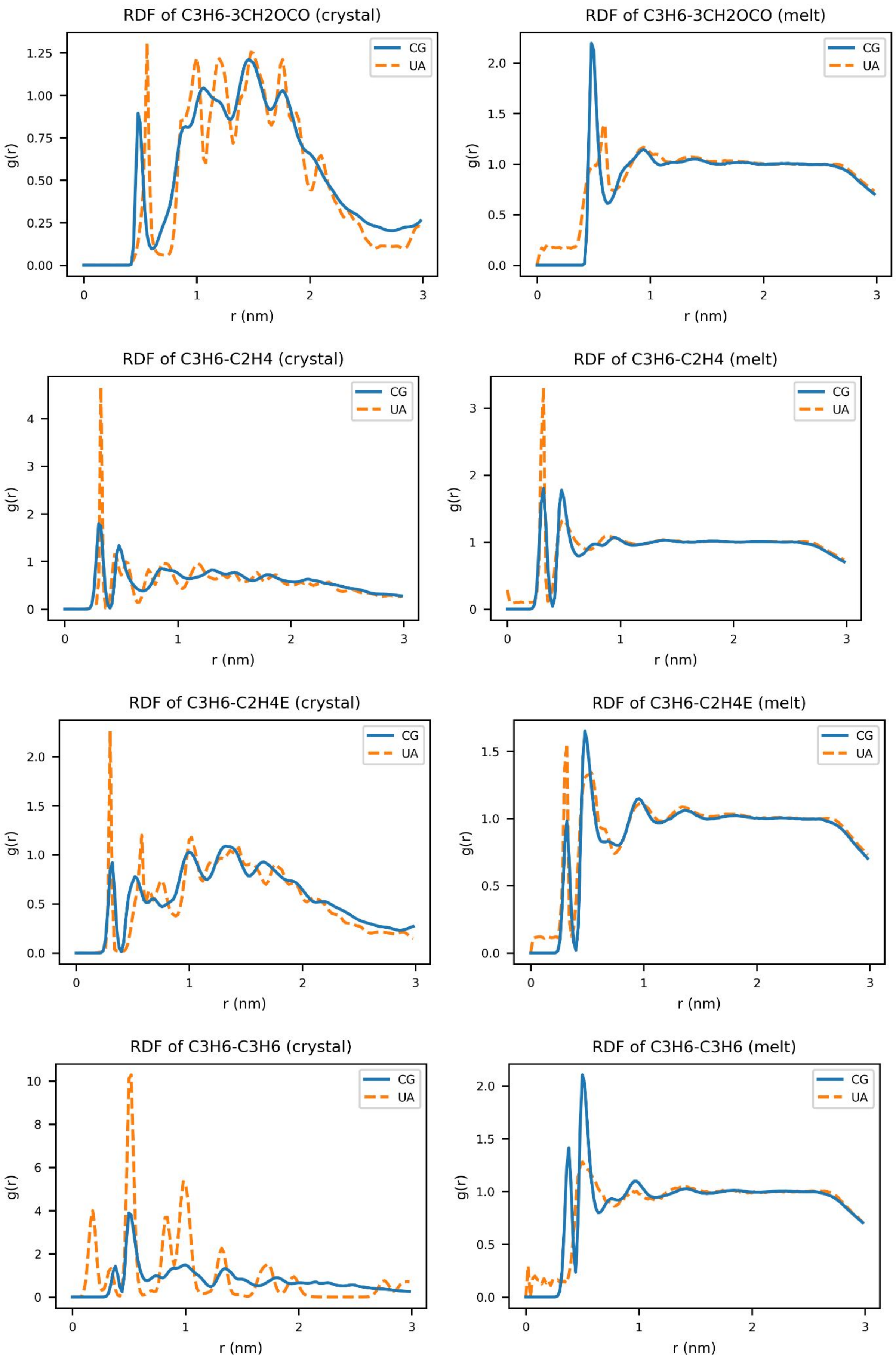

RDF of C3H6-3CH2OCO (crystal)
RDF of C3H6-3CH2OCO (melt)
RDF of C3H6-C2H4 (crystal)
RDF of C3H6-C2H4 (melt)
RDF of C3H6-C2H4E (crystal)
RDF of C3H6-C2H4E (melt)
RDF of C3H6-C3H6 (crystal)
RDF of C3H6-C3H6 (melt)
g(r)
r (nm)
CG
UA

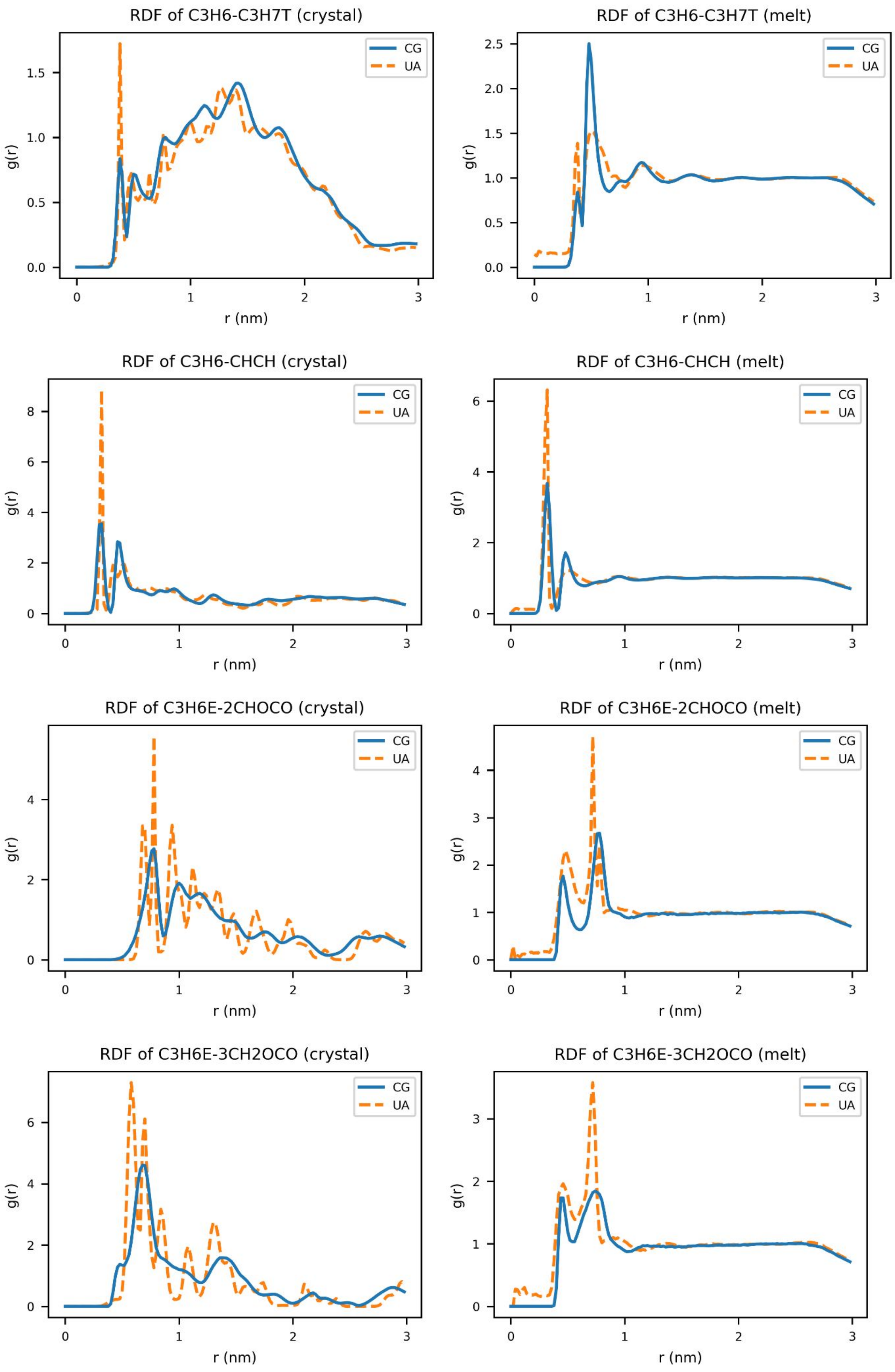
RDF of C3H6-C3H7T (crystal)
RDF of C3H6-C3H7T (melt)
RDF of C3H6-CHCH (crystal)
RDF of C3H6-CHCH (melt)
RDF of C3H6E-2CHOCO (crystal)
RDF of C3H6E-2CHOCO (melt)
RDF of C3H6E-3CH2OCO (crystal)
RDF of C3H6E-3CH2OCO (melt)
CG
UA
g(r)
r (nm)

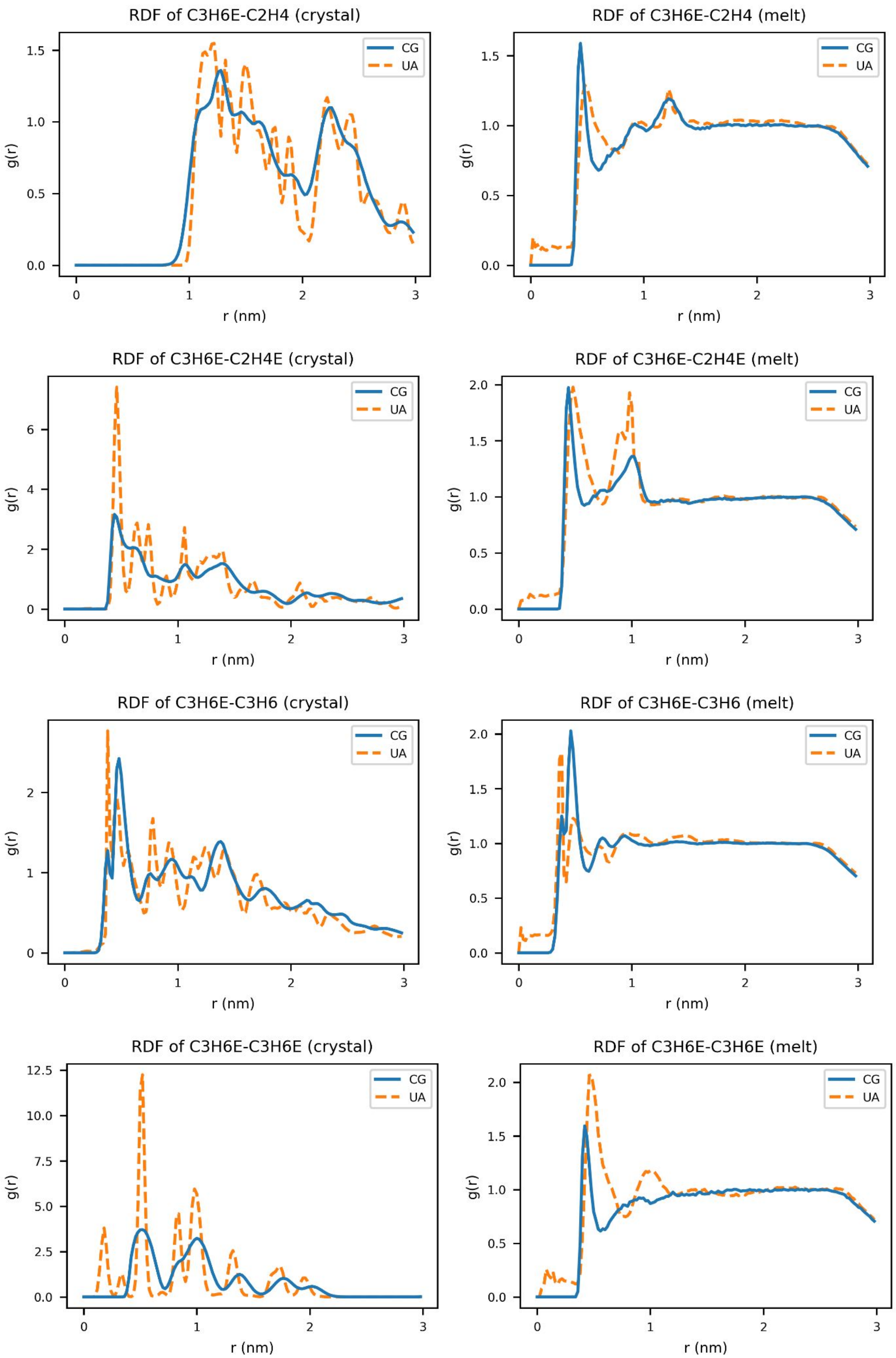
RDF of C3H6E-C2H4 (crystal)
RDF of C3H6E-C2H4 (melt)
RDF of C3H6E-C2H4E (crystal)
RDF of C3H6E-C2H4E (melt)
RDF of C3H6E-C3H6 (crystal)
RDF of C3H6E-C3H6 (melt)
RDF of C3H6E-C3H6E (crystal)
RDF of C3H6E-C3H6E (melt)
CG
UA
g(r)
r (nm)

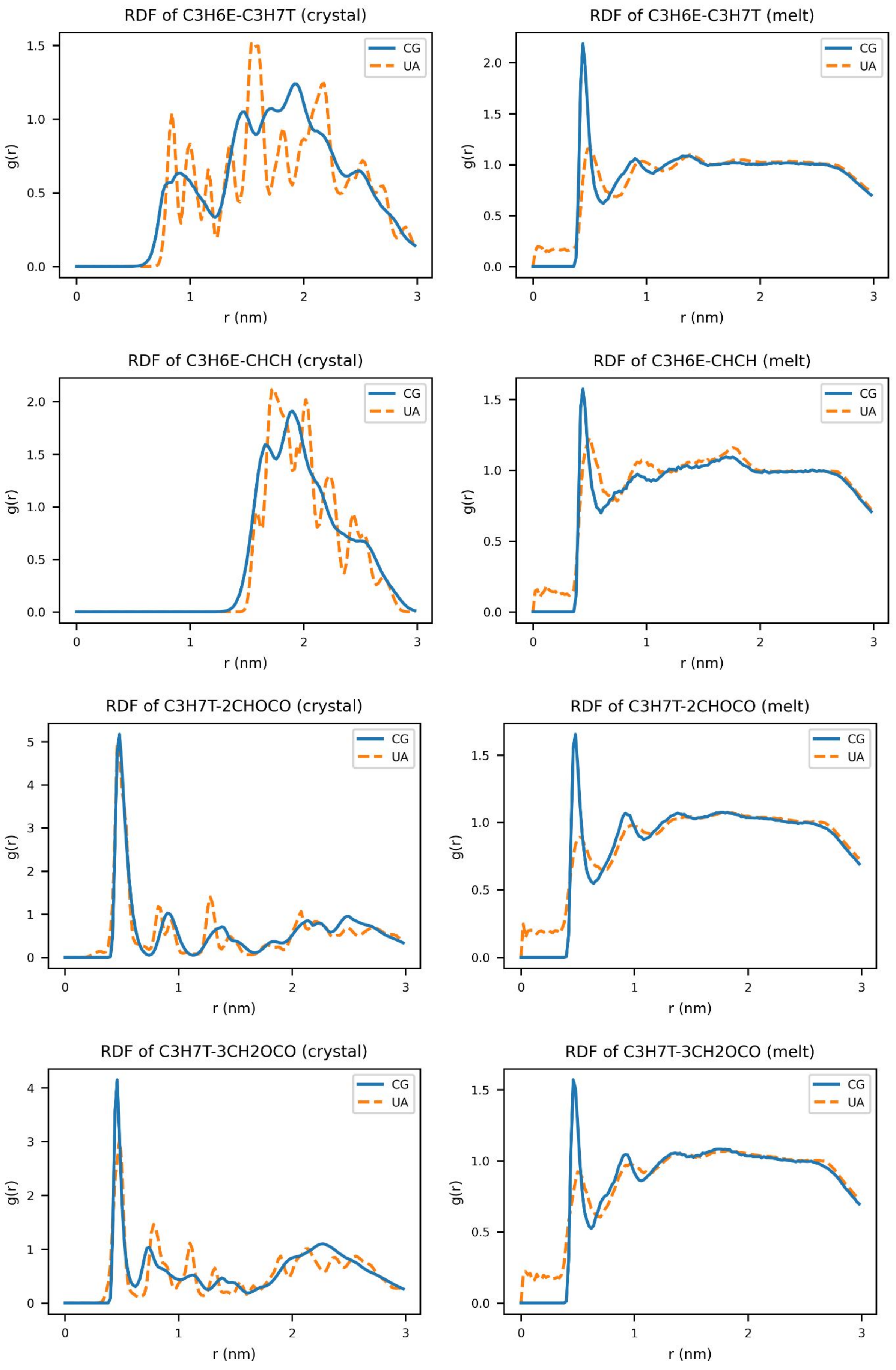
RDF of C3H6E-C3H7T (crystal)
RDF of C3H6E-C3H7T (melt)
RDF of C3H6E-CHCH (crystal)
RDF of C3H6E-CHCH (melt)
RDF of C3H7T-2CHOCO (crystal)
RDF of C3H7T-2CHOCO (melt)
RDF of C3H7T-3CH2OCO (crystal)
RDF of C3H7T-3CH2OCO (melt)
CG
UA
g(r)
r (nm)

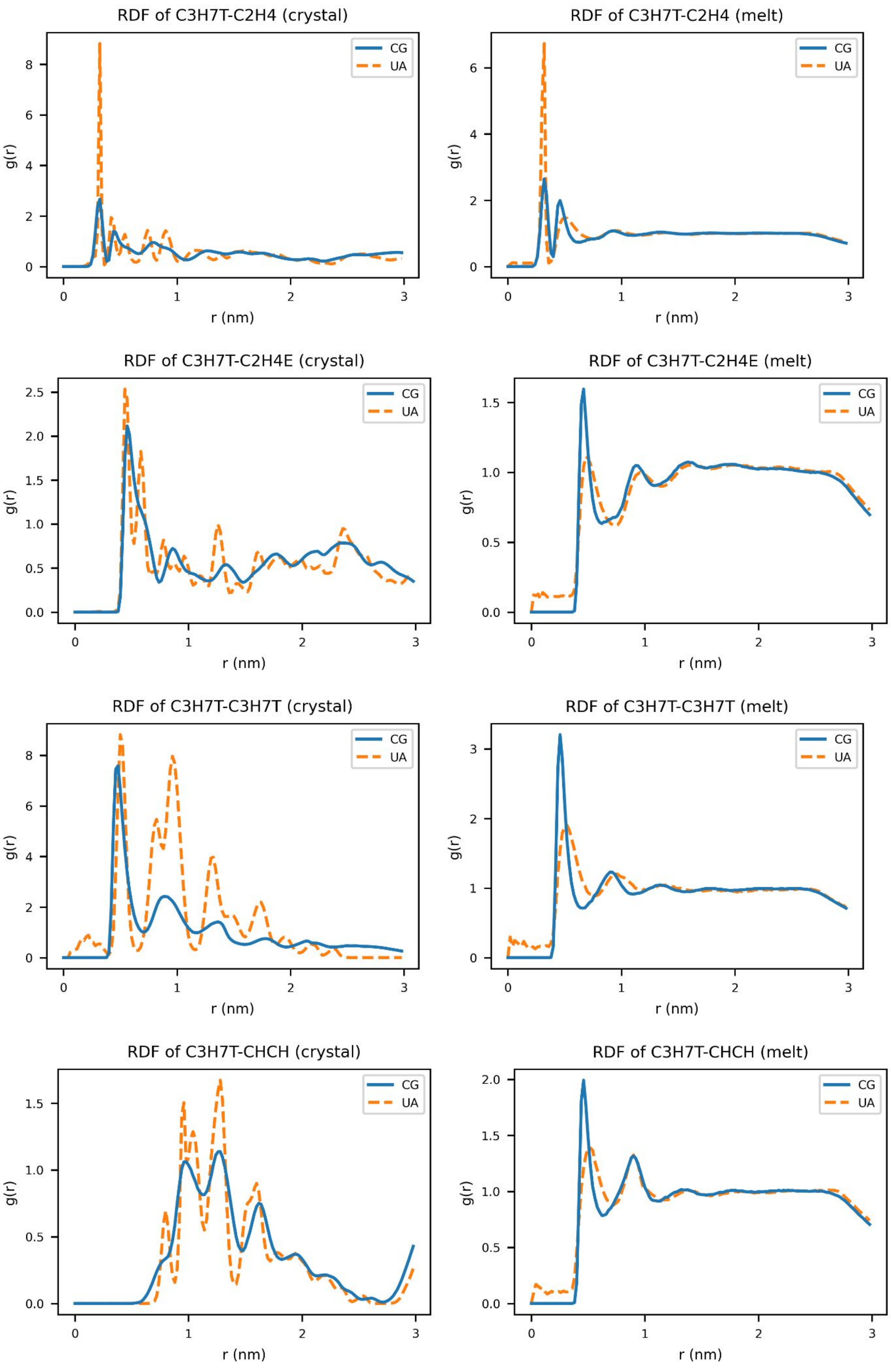
RDF of C3H7T-C2H4 (crystal)
RDF of C3H7T-C2H4 (melt)
RDF of C3H7T-C2H4E (crystal)
RDF of C3H7T-C2H4E (melt)
RDF of C3H7T-C3H7T (crystal)
RDF of C3H7T-C3H7T (melt)
RDF of C3H7T-CHCH (crystal)
RDF of C3H7T-CHCH (melt)
CG
UA
g(r)
r (nm)

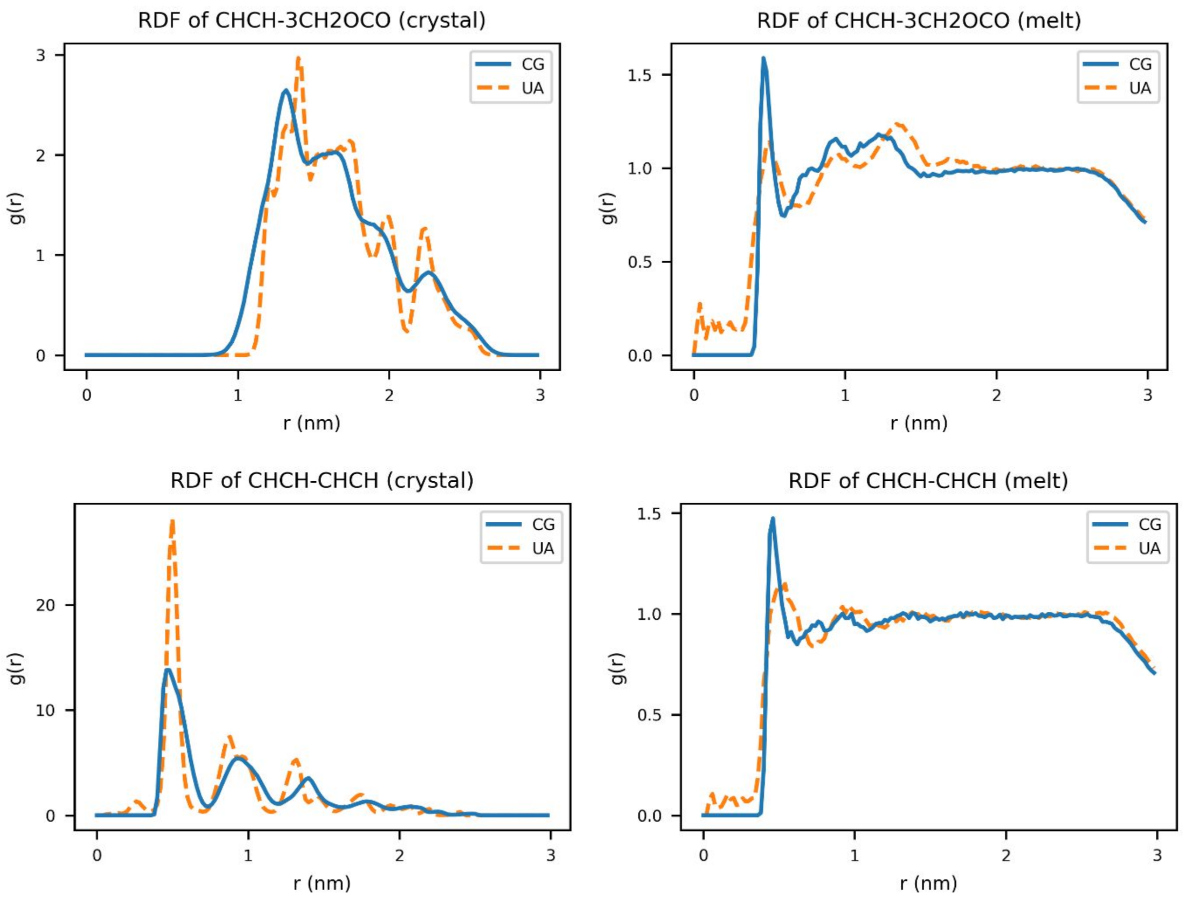

RDF of CHCH-3CH2OCO (crystal)
RDF of CHCH-3CH2OCO (melt)
RDF of CHCH-CHCH (crystal)
RDF of CHCH-CHCH (melt)
g(r)
r (nm)
CG
UA

SI.8 Simulated densities (g/cm^3) of *sn*-POSt using different equilibration temperatures and time-steps for robustness testing of the COGITO FF. *Note: a density of 0 means that that simulation crashed.*

		Melt density (g/cm^3)				Calculated for a 25 fs time-step or less	
Temperature (K)	Time-step (fs)	Run 1	Run 2	Run 3	Average	Average	StDev
280	2	0.925	0.925	0.925	0.925	0.925	< 0.001
280	10	0.924	0.925	0.924	0.924		
280	20	0.925	0.925	0.925	0.925		
280	25	0.925	0.925	0.925	0.925		
280	30	0.925	0.926	0.926	0.925		
280	40	0	0	0	-		
280	50	0	0	0	-		
300	2	0.914	0.914	0.914	0.914	0.914	< 0.001
300	10	0.914	0.914	0.914	0.914		
300	20	0.914	0.914	0.914	0.914		
300	25	0.914	0.914	0.915	0.914		
300	30	0.915	0.914	0.915	0.915		
300	40	0	0	0	-		
300	50	0	0	0	-		
325	2	0.900	0.900	0.900	0.900	0.900	< 0.001
325	10	0.900	0.900	0.900	0.900		
325	20	0.901	0.901	0.901	0.901		
325	25	0.901	0.901	0.901	0.901		
325	30	0.901	0	0.901	-		
325	40	0	0	0	-		
325	50	0	0	0	-		
350	2	0.887	0.887	0.887	0.887	0.887	< 0.001

350	10	0.887	0.887	0.887	0.887
350	20	0.887	0.887	0.887	0.887
350	25	0.887	0.887	0.887	0.887
350	30	0.888	0.888	0.888	0.888
350	40	0	0	0	-
350	50	0	0	0	-

		β_2 density (g/cm^3)				Calculated for a 25 fs time-step or less	
Temperature (K)	Time-step (fs)	Run 1	Run 2	Run 3	Average	Average	StDev
280	2	0.973	0.97	0.971	0.971	0.972	0.001
280	10	0.972	0.973	0.971	0.972		
280	20	0.973	0.973	0.971	0.972		
280	25	0.973	0.971	0.97	0.971		
280	30	0.972	0.972	0.971	0.972		
280	40	0	0	0	-		
280	50	0	0	0	-		
300	2	0.965	0.963	0.962	0.963	0.964	0.002
300	10	0.962	0.966	0.961	0.963		
300	20	0.962	0.965	0.964	0.964		
300	25	0.963	0.968	0.964	0.965		
300	30	0.963	0.963	0.963	0.963		
300	40	0	0	0	-		
300	50	0	0	0	-		
325	2	0.95	0.949	0.949	0.949	0.951	0.002
325	10	0.948	0.952	0.953	0.951		
325	20	0.953	0.953	0.952	0.953		
325	25	0.95	0.951	0.952	0.951		

325	30	0	0	0	-		
325	40	0	0	0	-		
325	50	0	0	0	-		
350	2	0.94	0.936	0.94	0.939	0.940	0.002
350	10	0.94	0.939	0.94	0.940		
350	20	0.941	0.94	0.94	0.940		
350	25	0.941	0.941	0.937	0.940		
350	30	0	0	0	-		
350	40	0	0	0	-		
350	50	0	0	0	-		

SI.9.1 Simulated enthalpies and enthalpies of fusion of TAGs

sn-POP (empirical melting temperature = simulation temperature = 310 K)

Run	Crystalline enthalpy (kJ/mol)	95% CI	Melt enthalpy (kJ/mol)	95% CI	ΔH_{fus} (kJ/mol)	95% CI	ΔH_{fus} * 3.5 (kJ/mol)	Data points
1	-290.5	0.12	-250.77	0.13	39.73	0.18	139.06	1000
2	-290.34	0.13	-250.85	0.13	39.49	0.18	138.22	1000
3	-290.21	0.12	-250.92	0.13	39.29	0.18	137.52	1000
4	-290.12	0.12	-250.69	0.13	39.43	0.18	138.01	1000
5	-290.33	0.12	-250.84	0.14	39.49	0.18	138.22	1000
6	-290.14	0.12	-250.76	0.13	39.38	0.18	137.83	1000
7	-290.14	0.12	-250.71	0.13	39.43	0.18	138.01	1000
8	-290.28	0.13	-250.9	0.14	39.38	0.19	137.83	1000
9	-290.25	0.12	-250.63	0.13	39.62	0.18	138.67	1000
10	-290.31	0.13	-250.84	0.13	39.47	0.18	138.15	1000
Overall	-290.26	0.04	-250.79	0.04	39.47	0.06	138.15	10000

sn-POSt (empirical melting temperature = simulation temperature = 308 K)

Run	Crystalline enthalpy (kJ/mol)	95% CI	Melt enthalpy (kJ/mol)	95% CI	ΔH_{fus} (kJ/mol)	95% CI	ΔH_{fus} * 3.5 (kJ/mol)	Data points
1	-314.6	0.13	-273.72	0.14	40.88	0.19	143.08	1000
2	-314.24	0.13	-273.61	0.14	40.63	0.19	142.21	1000
3	-315.8	0.13	-273.51	0.13	42.29	0.18	148.02	1000
4	-315	0.13	-273.5	0.14	41.5	0.19	145.25	1000
5	-314.38	0.12	-273.59	0.14	40.79	0.18	142.77	1000
6	-314.08	0.14	-273.64	0.14	40.44	0.2	141.54	1000
7	-315.14	0.12	-273.7	0.13	41.44	0.18	145.04	1000
8	-314.55	0.13	-273.63	0.14	40.92	0.19	143.22	1000
9	-314.17	0.13	-273.63	0.13	40.54	0.18	141.89	1000
10	-314.05	0.13	-273.67	0.14	40.38	0.19	141.33	1000
Overall	-314.6	0.04	-273.62	0.04	40.98	0.06	143.43	10000

sn-StOSt (empirical melting temperature = simulation temperature = 317 K)

Run	Crystalline enthalpy (kJ/mol)	95% CI	Melt enthalpy (kJ/mol)	95% CI	ΔH_{fus} (kJ/mol)	95% CI	ΔH_{fus} * 3.5 (kJ/mol)	Data points
1	-340.43	0.14	-288.18	0.14	52.25	0.2	182.88	1000
2	-331.13	0.14	-288.56	0.14	42.57	0.2	149.00	1000
3	-340.43	0.13	-288.22	0.14	52.21	0.19	182.74	1000
4	-340.41	0.14	-288	0.14	52.41	0.2	183.44	1000
5	-329.62	0.14	-288.25	0.14	41.37	0.2	144.80	1000
6	-340.58	0.13	-288.12	0.14	52.46	0.19	183.61	1000
7	-329.49	0.14	-288.21	0.14	41.28	0.2	144.48	1000
8	-340.46	0.13	-288.42	0.14	52.04	0.19	182.14	1000
9	-340.45	0.13	-288.47	0.14	51.98	0.19	181.93	1000
10	-329.96	0.14	-288.18	0.14	41.78	0.2	146.23	1000
Overall	-336.3	0.11	-288.26	0.04	48.04	0.12	168.14	10000

sn-StStSt (empirical melting temperature = simulation temperature = 346.25 K)

Run	Crystalline enthalpy (kJ/mol)	95% CI	Melt enthalpy (kJ/mol)	95% CI	ΔH_{fus} (kJ/mol)	95% CI	ΔH_{fus} * 3.5 (kJ/mol)	Data points

1	-306.93	0.14	-250.69	0.15	56.24	0.21	196.84	1000
2	-303.46	0.14	-250.36	0.15	53.1	0.21	185.85	1000
3	-305.72	0.14	-250.79	0.15	54.93	0.21	192.26	1000
4	-306.8	0.14	-250.46	0.15	56.34	0.21	197.19	1000
5	-303.48	0.15	-250.4	0.15	53.08	0.21	185.78	1000
6	-303.37	0.14	-250.55	0.15	52.82	0.21	184.87	1000
7	-306.74	0.14	-250.38	0.15	56.36	0.21	197.26	1000
8	-301.04	0.14	-250.71	0.14	50.33	0.2	176.16	1000
9	-304.25	0.14	-250.39	0.15	53.86	0.21	188.51	1000
10	-304.97	0.14	-250.43	0.15	54.54	0.21	190.89	1000
Overall	-304.68	0.06	-250.51	0.05	54.17	0.08	189.60	10000

sn-MMM (empirical melting temperature = simulation temperature = 330.15 K)

Run	Crystalline enthalpy (kJ/mol)	95% CI	Melt enthalpy (kJ/mol)	95% CI	ΔH_{fus} (kJ/mol)	95% CI	ΔH_{fus} * 3.5 (kJ/mol)	Data points
1	-277.95	0.13	-235.71	0.14	42.24	0.19	147.84	1000
2	-281.98	0.12	-235.24	0.13	46.74	0.18	163.59	1000
3	-280.09	0.12	-235.29	0.14	44.8	0.18	156.80	1000
4	-276.51	0.13	-235.29	0.14	41.22	0.19	144.27	1000
5	-275.92	0.12	-235.56	0.13	40.36	0.18	141.26	1000
6	-275.09	0.12	-235.13	0.13	39.96	0.18	139.86	1000
7	-275.99	0.12	-235.71	0.14	40.28	0.18	140.98	1000
8	-282.5	0.12	-235.27	0.13	47.23	0.18	165.31	1000
9	-274.76	0.13	-235.57	0.13	39.19	0.18	137.17	1000
10	-278.31	0.12	-235.19	0.14	43.12	0.18	150.92	1000
Overall	-277.91	0.07	-235.4	0.04	42.51	0.08	148.79	10000

SI.9.2 Simulated energies and enthalpies of vaporization of TAGs

sn-StStSt (simulation temperature = 506 K)

Run	Melt energy (kJ/mol)	95% CI	Gas energy (kJ/mol)	95% CI	ΔH_{vap} (kJ/mol)	95% CI	ΔH_{vap} / 1.5 (kJ/mol)	Data points
1	114.22	2.17	-133.39	0.21	251.82	2.18	167.88	1000
2	115.12	2.11	-133.68	0.21	253.01	2.12	168.6733	1000

3	114.96	2.16	-133.78	0.21	252.95	2.17	168.6333	1000
4	115.15	2.09	-133.46	0.21	252.82	2.1	168.5467	1000
5	117.14	2.16	-133.43	0.21	254.78	2.17	169.8533	1000
6	112.71	2.06	-133.45	0.21	250.37	2.07	166.9133	1000
7	116.96	2.13	-133.3	0.21	254.47	2.14	169.6467	1000
8	114.73	2.13	-133.49	0.21	252.43	2.14	168.2867	1000
9	115.91	2.15	-133.28	0.2	253.4	2.16	168.9333	1000
10	114.53	2.1	-133.42	0.2	252.16	2.11	168.1067	1000
Overall	115.14	0.66	-133.47	0.07	252.82	0.66	168.5467	10000

sn-MMM (simulation temperature = 469 K)

Run	Melt energy (kJ/mol)	95% CI	Gas energy (kJ/mol)	95% CI	ΔH_{vap} (kJ/mol)	95% CI	ΔH_{vap} / 1.5 (kJ/mol)	Data points
1	75.65	1.79	-148.15	0.18	227.7	1.8	151.8	1000
2	77.42	1.8	-147.84	0.18	229.16	1.81	152.7733	1000
3	77.24	1.78	-148.29	0.18	229.43	1.79	152.9533	1000
4	81.33	1.84	-148.22	0.18	233.45	1.85	155.6333	1000
5	76.49	1.83	-147.95	0.17	228.34	1.84	152.2267	1000
6	79.85	1.86	-148.19	0.17	231.94	1.87	154.6267	1000
7	75.82	1.76	-147.91	0.18	227.63	1.77	151.7533	1000
8	75.23	1.8	-148.3	0.17	227.43	1.81	151.62	1000
9	76.88	1.85	-148.25	0.18	229.03	1.86	152.6867	1000
10	80.28	3.66	-148.12	0.18	232.3	3.66	154.8667	1000
Overall	77.5	0.64	-148.12	0.06	229.52	0.64	153.0133	10000
